# Transcriptomic and Gene Expression Analysis of Chemosensory Genes from White Grubs of *Hylamorpha elegans* (Coleoptera: Scarabaeidae), a Subterranean Pest in South America

**DOI:** 10.3390/insects15090660

**Published:** 2024-08-30

**Authors:** Paula Lizana, Ana Mutis, Rubén Palma-Millanao, Giovanni Larama, Binu Antony, Andrés Quiroz, Herbert Venthur

**Affiliations:** 1 Programa de Doctorado en Ciencias de Recursos Naturales, Universidad de La Frontera, Temuco 4811230, Chile; p.lizana02@ufromail.cl; 2 Laboratorio de Química Ecológica, Departamento de Ciencias Químicas y Recursos Naturales, Facultad de Ingeniería y Ciencias, Universidad de La Frontera, Temuco 4811230, Chile; ana.mutis@ufrontera.cl (A.M.); andres.quiroz@ufrontera.cl (A.Q.); 3 Centro de Investigación Biotecnológica Aplicada al Medio Ambiente (CIBAMA), Universidad de La Frontera, Temuco 4811230, Chile; 4 Vicerrectoría de Investigación y Postgrado, Universidad de La Frontera, Temuco 4811230, Chile; 5 Biocontrol Research Laboratory and Scientific and Technological Bioresource Nucleus, Universidad de La Frontera, Temuco 4811230, Chile; giovanni.larama@ufrontera.cl; 6 Chair of Date Palm Research, Center for Chemical Ecology and Functional Genomics, College of Food and Agricultural Sciences, King Saud University, Riyadh 11451, Saudi Arabia; bantony@ksu.edu.sa

**Keywords:** insect olfaction, chemosensation, RNA-seq, Coleoptera, white grubs, *Hylamorpha elegans*

## Abstract

**Simple Summary:**

*Hylamorpha elegans*, a native Chilean scarab beetle regarded as a significant pest, feeds on economically important crops, such as red clover, ryegrass, and wheat, in its larval stage (i.e., white grubs). Traditionally, chemical control through insecticides has been used against *H. elegans* without success, and alternative control strategies based on ethology and the use of semiochemicals have remained elusive. Thus, chemical communication through chemosensory genes in *H. elegans* could represent an advance towards integrated pest management. In this study, the repertoire of chemosensory gene candidates from white grubs of *H. elegans*, their phylogenetic relationships, and relative gene expression have been reported for the first time.

**Abstract:**

Olfaction and gustation processes play key roles in the life cycle of insects, such as finding and accepting food sources, oviposition sites, and mates, among other fundamental aspects of insect development. In this context, chemosensory genes found in sensory organs (e.g., antennae and maxillary palps) are crucial for understanding insect behaviour, particularly the phytophagous behaviour of insect pests that attack economically important crops. An example is the scarab beetle *Hylamorpha elegans*, which feeds on the roots of several crops important for livestock in its larval stage. In this study, chemosensory gene candidates of *H. elegans* white grubs identified through the head transcriptome and phylogenetic and tissue-biased gene expression (antennae, head without antennae, and legs) have been reported. Overall, 47 chemosensory genes were identified (2 ORs, 1 GR, 11 IRs, 9 CSPs, and 24 OBPs). Gene expression analysis revealed the predominant presence of IRs in the legs, whereas ORs and the GR were present in the heads and/or antennae. Particularly, HeleOBP9 and HeleCSP2 were significantly expressed in the head but not in the antennae or legs; these and other genes are discussed as potential targets in the context of *H. elegans* management.

## 1. Introduction

Olfaction is an important sensory system for insects and is involved in vital processes such as mate and host finding, oviposition, and food-related cues, particularly in species with nocturnal or subterranean habits [1,2]. Thus, a well-tuned olfactory system has evolved mainly in the antennae of insects, particularly inside small hair-like structures called sensilla. In the olfactory system, five protein families are involved in the perception of chemicals from the environment, and this interplay has been a focus of interest for pest control [3]. Odorant binding proteins (OBPs) and chemosensory proteins (CSPs) are two of the most notable. These proteins bind hydrophobic volatiles (e.g., pheromones and plant volatiles) and transport these compounds through aqueous sensillar lymph to facilitate their solubilization [4,5]. The role of OBPs as odorant transporters and the first filter of olfactory information make OBPs and CSPs the most studied proteins in insect chemosensation [6,7,8,9,10]. In addition to OBPs and CSPs, three families of chemoreceptors, namely odorant receptors (ORs), gustatory receptors (GRs), and ionotropic receptors (IRs), have been widely identified in insects. ORs have been extensively studied for their role as ligand-gated ion channels due to their clear function in odorant recognition, whether emitted by host plants or conspecific insects [11,12]. IRs and GRs have been the subject of interest in recent years due to their involvement in hygroreception and sugar/bitter perception, respectively [13,14,15,16,17,18]. Most of the research on insect chemosensation has been performed on adults due to their notorious olfaction-driven behaviors. Consequently, the search for an olfactory system in larvae has received less attention despite aggregation, food, or host-related behaviours having been described in the literature [19,20,21]. Remarkably, some hints of an olfactory system in larvae have been reported for fly larvae of *Procecidochares utilis*, *Drosophila* flies, and malaria-vector mosquitoes *Anopheles gambiae* [22,23,24,25]. Likewise, chemosensory genes have been identified for the larval stages of the moths *Spodoptera littoralis*, *S. exigua*, *Helicoverpa armigera*, and *H. assulta* [26,27,28,29]. More recently, the repertoire of chemosensory genes in the larvae of the leaf beetle *Plagiodera versicolora* (29 OBPs, 6 CSPs, 14 ORs, 13 GRs, and 8 IRs) was reported [30]. Regarding how this underground chemosensory system works, in the larvae of *Diabrotica virgifera virgifera,* it has been shown that CO_2_ can serve as a long-distance host location signal, with the GR2 receptor of *D. virgifera virgifera* being responsible for mediating this interaction [31]. Similarly, in the larvae of the bee *Melolontha hippocastani*, volatiles from plant roots are likely to be perceived by this chemosensory system, thus allowing the larvae to reach their host plants [32]. However, chemosensory genes in larvae with subterranean habits have not been studied extensively.

The scarab beetle *Hylamorpha elegans* is a pest that exhibits great subterranean phytophagous behaviour during its larval stage (i.e., white grubs), where it attacks the roots of economically important crops in Chile, such as wheat (*Triticum aestivum*), red clover (*Trifolium pratense*), white clover (*T. repens*), ryegrass (*Lolium perenne*), alfalfa (*Medicago sativa*), and oat (*Avena sativa*) [33,34,35]. This Coleopteran species, commonly called San Juan Verde, is endemic to South America and is distributed in central and southern Chile and southwestern Argentina [36,37]. During summer (December–February), *H. elegans* adults feed mainly on the leaves of the Patagonian oak *Nothofagus obliqua*, conjugating the presence of host plant volatiles and proposed sex pheromone components to mate [38,39,40]. Then, gravid females deposit their fertilised eggs 2–3 cm below the soil surface of crop fields, where they spend all their egg, larval, and pupa stages underground, and as they progress through their larval stage, they approach the surface [41]. White grubs begin to emerge in February, and three described instar larvae develop in March and October; the first to develop is L1, the second is L2, and the third is the prepupal stage L3. The pupal stage begins in November and ends in December. In the latter month, the adults emerge and group together around *Nothofagus* forests, starting the cycle again [42,43]. Although the impact of forestry is low, severe damage occurs in agriculture, where white grubs feed on organic matter (OM), forage plant roots, and annual crops (with wheat and red clover being the most affected), decreasing the amount of forage for cattle during winter [34,35]. In that sense, understanding the life cycle of *H. elegans* is fundamental to elucidating the damage it causes to different crops. Current evidence suggests that L2 can cause plant losses and a decrease in leaf weight, as evidenced in *L. perenne*. However, L3 causes the most damage to the previously mentioned plant [44]. Generally, scarabaeids at the larval stage can feed on OM, and it has been proposed that they can decrease the intensity of larval herbivory in soils with high OM content [45]. Interestingly, it has been shown that in soils with a high particulate organic matter (POM) content, root damage caused by *H. elegans* decreases, possibly because POM acts as an alternative food source [46,47]. There is evidence that semiochemicals such as 1,4-hydroquinone, 1,4-benzoquinone, β-ionone, and essential oil from *N. obliqua* affect the behaviour of adult *H. elegans* [38,39,40]. However, whether other chemicals in a subterranean environment have semiochemical activity on white grubs is still unknown. In this context, future studies on the behaviour of these white grubs, as well as their behaviour towards the host plant and even studies of volatiles from the roots of these plants, can contribute to obtaining a clearer understanding of the behaviour of this pest. Similarly, white grubs of *H. elegans* appear to make crucial behavioural decisions for feeding, where a chemosensory system may be key to the host search.

Considering that *H. elegans* is a subterranean pest at the larval stage with particular feeding behaviours and that a chemosensory system can provide key information about their chemical ecology in a dark environment, the aim of this study was to examine the chemosensory genes of *H. elegans* white grubs as well as their tissue-biased gene expression. First, head transcriptomes from two larval stages were obtained, and the repertoire of chemosensory genes (ORs, GRs, IRs, OBPs, and CSPs) was analysed. The relative gene expression according to the tissues (antennae, head without antennae, and legs) was evaluated to determine putative functions. Overall, this study provides a new set of chemosensory genes that, in the absence of genomic data, expands the repertoire of *H. elegans*. Furthermore, the results obtained in this study lay the groundwork for future functional studies targeting chemosensory genes to control white grubs in soil.

## 2. Materials and Methods

### 2.1. White Grub Collection and RNA Extraction

To obtain white grubs and subsequently total RNA, groups of *H. elegans* larvae were collected at the L2 and L3 stages in May and July, respectively, at the Maquehue Experimental Station of the Universidad de La Frontera, Temuco, Chile. On the same collection day, 25 heads of each larval stage (i.e., L2 and L3) were carefully dissected per replicate, and this was performed in triplicate; thus, a total of 75 white grub heads were dissected for L2, and 75 heads were dissected for L3. Subsequently, RNA extraction was performed with TRIzol reagent (Invitrogen, Carlsbad, CA, USA). Concentrations (ng/μL) were measured using a Quantus Fluorometer (Promega, Madison, WI, USA). Total RNA integrity was checked by 1% agarose gel electrophoresis. Once integrity and optimal concentrations were obtained, samples were precipitated with ethanol and subsequently sent to Macrogen (Seoul, Republic of Korea) for next-generation sequencing (NGS). Three head-derived RNA replicates for L2 and L3 were considered for NGS.

### 2.2. Sequencing and De Novo Transcriptome Assembly

The RNA samples were subjected to library preparation with the TruSeq mRNA Sample Prep Kit (Macrogen, Republic of Korea) and subsequently sequenced on a NovaSeq 6000 platform for 150 cycles in paired-end mode. Following sequencing, the resulting FASTQ files containing both the sequences and their corresponding quality scores were subjected to preprocessing using Trimmomatic v0.38 [48] to eliminate residual adapters and low-quality sequences. The retained high-quality reads were subsequently utilised for the de novo transcriptome assembly of *H. elegans* through Trinity v2.12 [49].

### 2.3. Transcriptome Assessment, Abundance Estimation, and Differential Expression

The initial assembly was subjected to clustering with a 95% similarity threshold using CD-HIT [50] to reduce redundancy. To evaluate the completeness of the assembly, BUSCO v5 [51] was used to compare the assembled transcripts to a reference collection of proteins (Embryophyta odb10) based on similarity. The relative abundance was estimated using RSEM v1.2.26 [52]. Differences in relative RNA abundance were assessed using the Bioconductor package DESeq2 [53] within the R statistics environment. The significance of these changes in gene expression was determined based on a false discovery rate (FDR) threshold of less than 0.05 and a minimum fold change (FC) of 4.

### 2.4. Transcriptome Annotation and Candidate Gene Identification

The methodology proposed by Gu et al. (2015) [54] was followed with some modifications to identify and annotate chemosensory genes in white grubs. A database for each of the protein families (ORs, GRs, IRs, OBPs, and CSPs) was manually constructed using amino acid sequences in FASTA format from another Coleopteran, such as *Tribolium castaneum, Dendroctonus ponderosae, Ips typographus, Megacyllene caryae, Holitrichia oblita, H. paralella, Anoplophora glabripennis, Anomala corpulenta, Rhynchophorus ferrugineus, Anoplophora chinensis, Tenebrio molitor*, or *Agrilus planipennis*. Similarly, sequences from two widely studied model insects, *Drosophila melanogaster* and *Bombyx mori*, were added. To identify chemosensory genes, an in-house database of Coleopteran chemosensory genes was created through local BLAST, and then with the resulting files, transcripts were identified by local searches using the NCBI BLASTx and BLASTn tools with an e-value of 1 × 10^−5^ as the threshold [55]. Subsequently, a second search round with local BLAST was performed with sequences from *H. elegans* adults and larvae in order to increase the number of identified transcripts. The ORF FINDER tool was subsequently used to determine the open reading frame of each unigene (ORF) (https://www.ncbi.nlm.nih.gov/orffinder accessed on 1 September 2022). An annotation table with the resulting parameters was prepared with the obtained data (Tables S1–S5). Finally, the transcripts per kilobase million (TPM) values from the head transcriptomes of plants in each larval stage (L2 and L3) were calculated based on the formula log_10_TPM to generate heatmaps with R and R Studio software version 4.1.3.

### 2.5. Phylogenetic Analysis

Evolutionary relationships were performed for the five families of chemosensory genes (i.e., OBPs, CSPs, ORs, IRs, and GRs). The amino acid sequences of the chemosensory genes were extracted from the literature and databases such as NCBI. The ORFs of *H. elegans* chemosensory genes were used for phylogenetic tree construction. All the sequences were aligned using the MAFFT server [56]. The best substitution models were identified via an automatic model search by ModelFinder [57], which revealed JTT + F + G4 for ORs, LG + F + G4 for IRs and GRs, LG + I + G4 for CSPs, and WAG + I + G4 for OBPs. The maximum likelihood analysis was performed with default settings and ultrafast bootstrap support with RAxML software v. 8 [58]. FigTree software (http://tree.bio.ed.ac.uk/software/figtree/ accessed on 1 September 2022) and the image editor Inkscape 0.48 were used to visualise and edit each phylogenetic tree.

### 2.6. Tissue-Based Relative Expression Determined by qRT–PCR

First-strand cDNA was synthesised from 1 μg of total RNA using the PrimeScript™ cDNA synthesis kit (Takara Bio, Inc., San Jose, CA, USA), with DNase I pretreatment for each tissue (antennae, head without antennae, and legs). All qRT–PCR analyses were carried out using specific primers designed in PRIMER3 v. 0.4.0 (http://bioinfo.ut.ee/primer3-0.4.0/ accessed on 1 September 2022) for all identified chemosensory genes, including the ribosomal protein RPL18, which was the best internal control compared with β-actin, α-tubulin, glyceraldehyde-3-phosphate dehydrogenase (GAPDH), and elongation Factor 1-alpha (EF1α) (Figure S1); moreover, the efficiencies were measured using melting curves. The qRT-PCR experiments were carried out using a qPCR Brilliant II SYBR Master Mix on an AriaMx real-time PCR system. The following conditions were used: 95 °C for 10 min; 40 cycles of 95 °C for 15 s, 60 °C for 15 s, and 72 °C for 20 s; and melting curve analysis (95 °C for 15 s, 60 °C for 1 min, and 95 °C for 15 s). All the reactions were performed using three biological replicates, each with three technical replicates. Moreover, the relative differences between proteins were analysed using the 2^−ΔΔCt^ method [59,60], and statistical significance was evaluated by one-way ANOVA; all the results were integrated into the R package qPCRTools [61] in R Studio.

## 3. Results

### 3.1. Sequencing and De Novo Assembly

Transcriptome data were obtained using an Illumina NovaSeq6000 platform. Both samples (L2 and L3) were merged for assembly, which produced 48,112 transcripts and 28,125 genes with a contig length N50 of 2305 bp (Table 1). After assembly, 19,457,430; 20,719,633; and 28,865,158 high-quality reads were obtained for the L2 samples. Similarly, 29,700,456; 22,681,291; and 20,635,375 high-quality reads were obtained for the L3 samples (Table S7). Statistical analysis revealed 834 differentially expressed genes (DEGs) (Figure S3). Overall, 47 chemosensing-related transcripts were identified for *H. elegans* white grubs, hereafter referred to as HeleORs, HeleGRs, HeleIRs, HeleOBPs, and HeleCSPs.

### 3.2. Transcript Annotation and Abundance Estimation of Chemoreceptors

**Figure 1:**
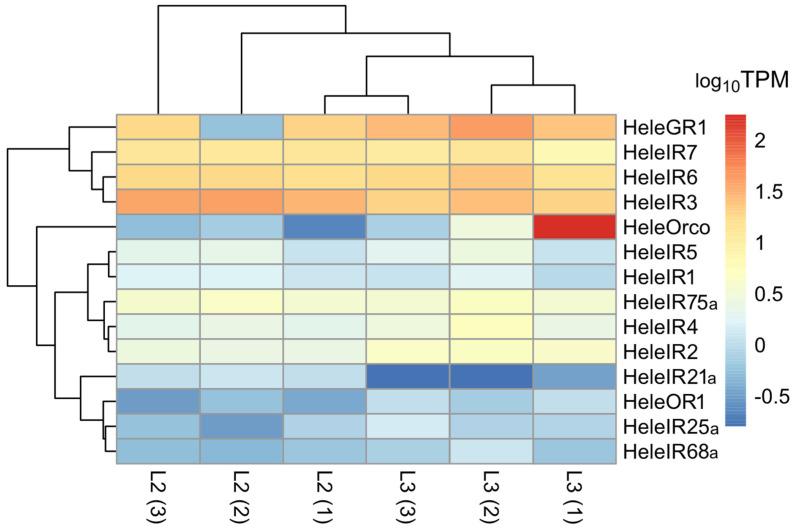
Heatmap of transcript abundance (log_10_TPM) for chemoreceptors (ORs, GRs, and IRs) calculated from the assembled head transcriptome of *H. elegans* white grubs. The numbers in parentheses indicate the biological replicates of each larval stage.

Regarding the ORs, two full-length ORs were identified that corresponded to Orco and OR1. HeleOrco was scored as complete because it presented seven transmembrane domains, while HeleOR1 was scored as partial because of its five transmembrane domains (Table S1). The two ORs identified were more abundant in L3 than in L2, with TPM values of 0.22, 0.72, and 0.55 in L2, and values of 177.43, 2.86, and 0.79 in L3 for each replicate of HeleOrco, whereas for HeleOR1, the TPM values were 0.38, 0.59, and 0.32 in L2 and 1.12, 0.7, and 1.13 in L3 (Figure 1).

Among the GRs, one was identified that was annotated as complete by both length and the seven transmembrane domains that it contained (Table S2). GR1 was more abundant in L3, with values of 24.9, 43.36, and 28.02, while in L2, it presented abundance values of 19.01, 0.59, and 18.65 (Figure 1).

For the IRs, 11 IRs were identified, where HeleIR2, 3, 4, 5, and 7 were annotated as complete because they presented three transmembrane domains, while HeleIR1, 21a, 25a, 68a, and 75a were annotated as partial because they presented only one transmembrane domain (Table S3). In terms of abundance, HeleIR1, 2, and 4 showed the highest levels among the HeleIRs. Among the two larval stages, HeleIR3 showed a greater abundance in L2, with TPM values of 30.09, 39.59, and 37.55, while in L3, the values were lower, with 19.06, 25.68, and 19.04, respectively (Figure 1).

### 3.3. Chemoreceptor–Phylogenetic Relationships

**Figure 2:**
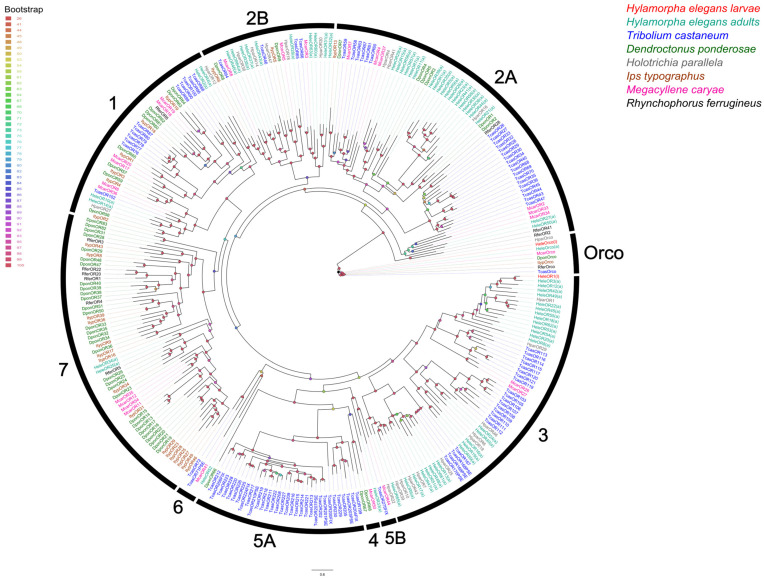
Phylogenetic relationships of odorant receptors (ORs) from H. elegans white grubs with ORs from other Coleopterans. Different species are highlighted with different colours and are indicated in the upper right. White grubs of *H. elegans* were annotated with (l), while adults of *H. elegans* are annotated with (a). Each clade is highlighted in black arrows and named according to Mitchell et al. (2020) [62]. The bootstrap data are indicated by node spheres and coloured according to the percentage of confidence. The Orco clade was used to root the tree.

In terms of phylogenetic relationships, HeleOrco clustered in a single clade together with other beetle Orcos, such as *H. paralella*, *T. castaneum*, *D. ponderosae*, *M. caryae*, *R. ferrugineus*, *I. typographus,* and adult *H. elegans*. In comparison, HeleOR1 clustered in clade 3 together with other receptors of *T. castaneum*, *M. caryae*, *H. paralella,* and adults of *H. elegans*, where it showed a close evolutionary relationship with the paralogue gene HeleOR3(a) (with “(a)” indicating “adults”) (Figure 2).

**Figure 3:**
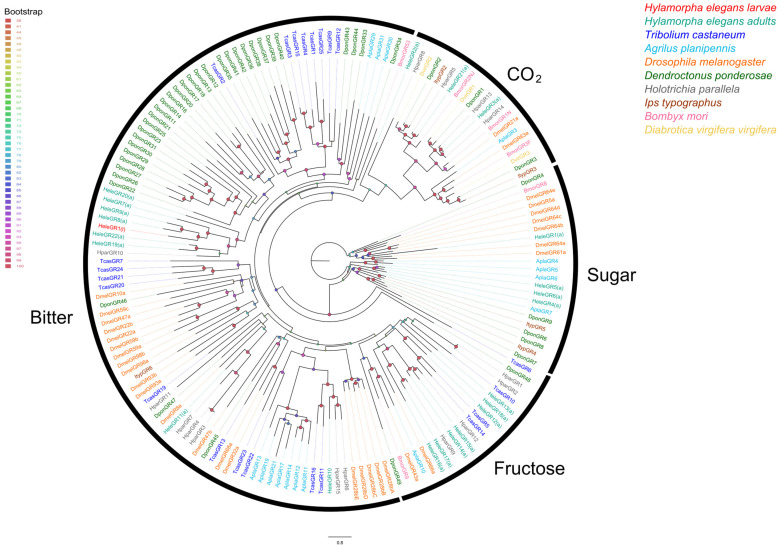
Phylogenetic relationships of candidate GRs of H. elegans white grubs with other GRs of Coleoptera, Diptera, and Lepidoptera of known function. White grubs of *H. elegans* are annotated with (l)**,** while adults of *H. elegans* are annotated with (a). The bootstrap data are indicated by node spheres and coloured according to the percentage of confidence.

With respect to the phylogenetic relationships of HeleGR1, it clustered in a bitter-related clade together with HeleGR8(a), HeleGR9(a), and a large expansion of DponGRs (Figure 3).

**Figure 4:**
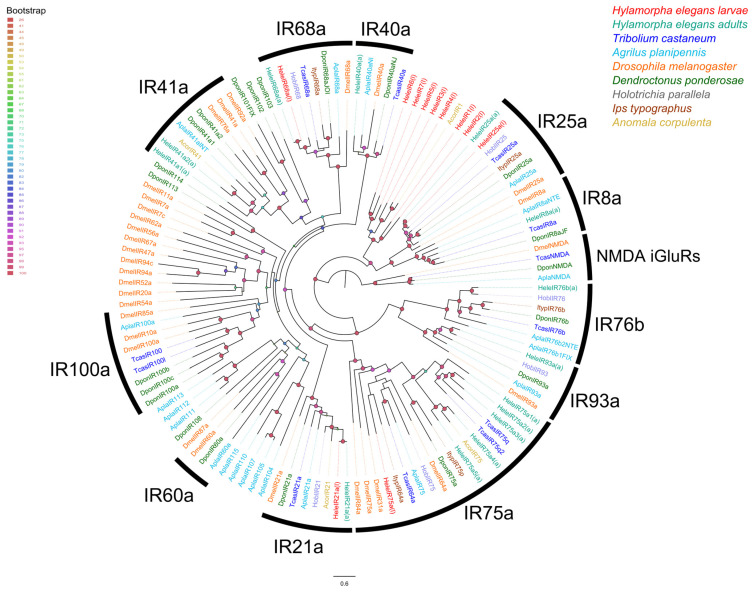
Phylogenetic relationships of candidate IRs of *H. elegans* white grubs with other IRs of Coleoptera, Diptera, and Lepidoptera of known function. White grubs of *H. elegans* are annotated with (l), while adults of *H. elegans* are annotated with (a). The bootstrap data are indicated by node spheres and colored according to the percentage of confidence.

For IRs, the phylogenetic tree showed that only four HeleIRs clustered into known clades. Thus, HeleIR68a clustered into clade IR68a, HeleIR75a into clade IR75a, HeleIR25a into clade IR25a, and HeleIR21a into clade IR21a. The remaining HeleIRs were distributed in clades of unknown function (Figure 4).

### 3.4. Differential Expression of Chemoreceptors

**Figure 5:**
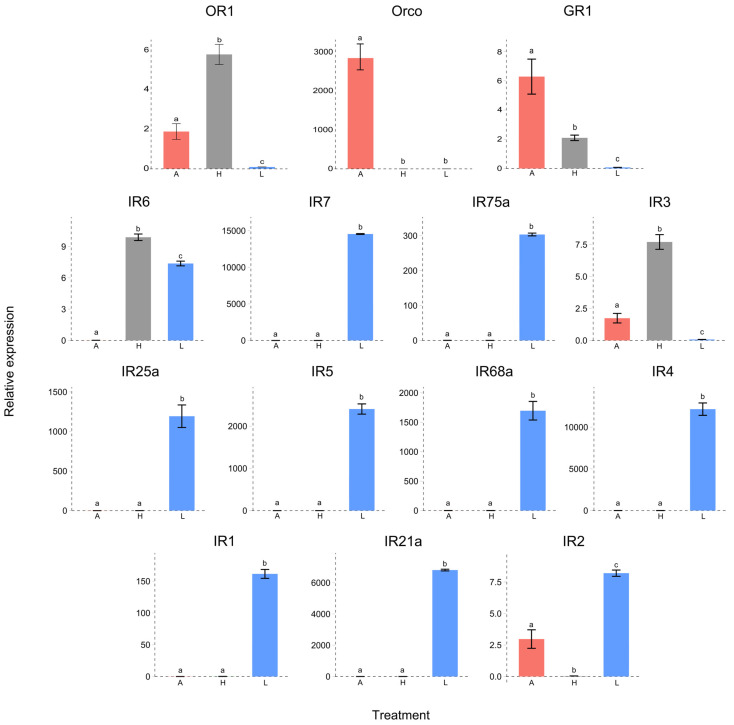
Expression levels of chemoreceptors (GRs, ORs, and IRs) in L3 of *H. elegans* white grubs. *H. elegans* with A, antennae; H, heads (without antennae); and L, legs. Different lowercase letters indicate significant differences (one-way ANOVA with Tukey’s test, *p* < 0.05).

To evaluate the tissue-biased expression of the chemoreceptors of *H. elegans*, white grubs, antennae, heads without antennae, and legs were evaluated (Figure 5). Our results revealed that HeleOR1 was more highly expressed in the heads without antennae than in the antennae and that its expression was almost null in the legs. HeleOrco was expressed only in the antennae. HeleGR1 was threefold more highly expressed in the antennae than in the heads. Among the IRs, only HeleIR6 and HeleIR3 exhibited significantly greater expression in the head than in the other tissues. HeleIR1, 2, 4, 5, 7, 21a, 25a, 68a, and 75a were more highly expressed in the legs than in the heads without antennae or in the antennae.

### 3.5. Transcript Annotation and Abundance Estimation of Soluble Proteins

**Figure 6:**
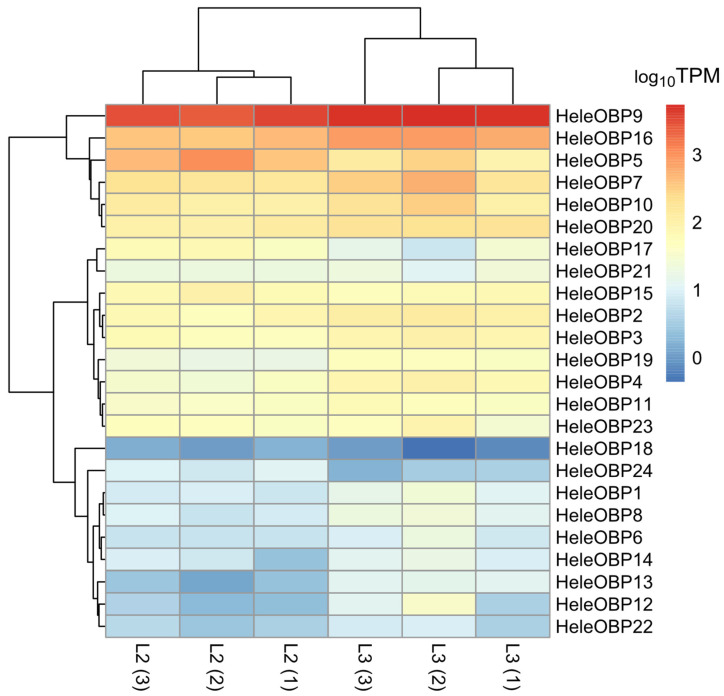
Heatmap of transcript abundance (TPM) for OBPs, calculated from the assembled head transcriptome of *H. elegans* white grubs. The numbers in parentheses indicate the biological replicates of each larval stage.

Among the OBPs, 24 HeleOBPs were identified from the transcriptome, where only HeleOBP14 was considered partial because it did not present a signal peptide, while the remaining 23 HeleOBPs were considered complete (Table S4). Among the HeleOBPs, HeleOBP9 had TPM values of 4958.16, 5475.18, and 4931.94 at the L3 stage, which were greater than those of L2, with values of 3497.28, 2530.27, and 2861.15, respectively (Figure 6).

**Figure 7:**
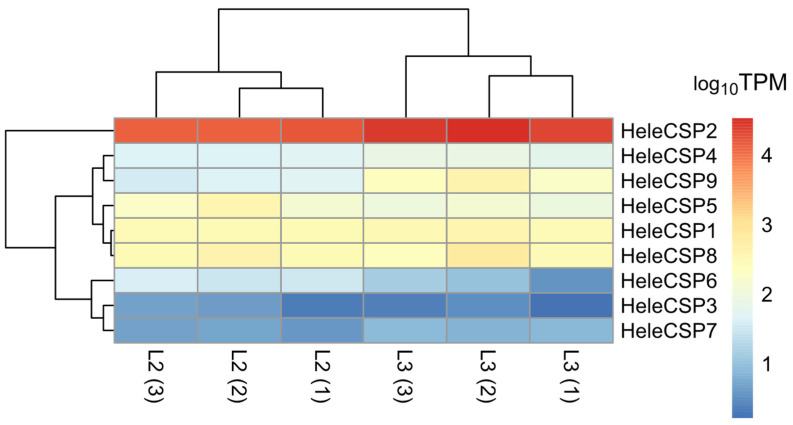
Heatmap of transcript abundance (TPM) for CSPs, calculated from the assembled head transcriptome of white grubs of *H. elegans*. The numbers in parentheses indicate the biological replicates of each larval stage.

For the second family of soluble proteins, nine CSPs were identified, all of which were considered complete because they presented a signal peptide (Table S5). Among the HeleCSPs, HeleCSP2 had the highest abundance. Particularly, in stage L3, it presented TPM values of 22,310.99, 33,795.75, and 25,738.09; and in stage L2, it had a lower abundance with values of 16,255.04, 12,655.54, and 13,599.63, respectively (Figure 7).

### 3.6. Phylogenetic Relationships of Soluble Proteins

**Figure 8:**
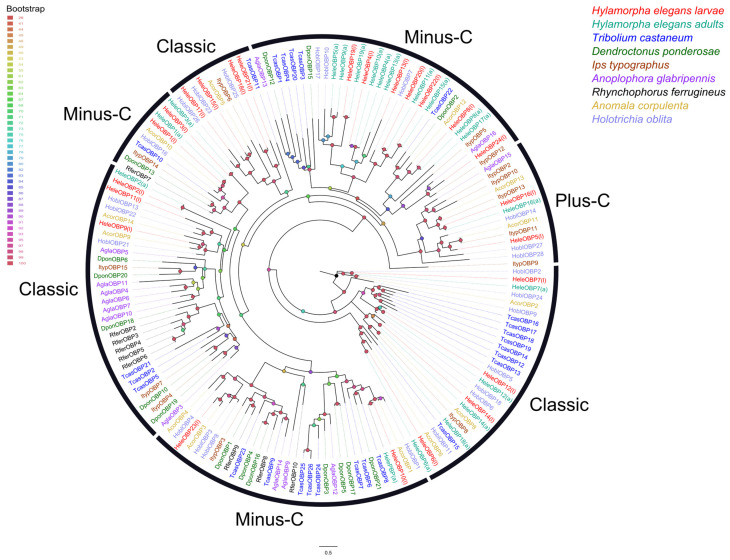
Phylogenetic relationships of odorant-binding proteins (OBPs) from *H. elegans* white grubs and OBPs from other Coleoptera species White grubs of *H. elegans* were annotated with (l), while adults of *H. elegans* are annotated with (a). The bootstrap data are indicated by node spheres and coloured according to the percentage of confidence.

**Figure 9:**
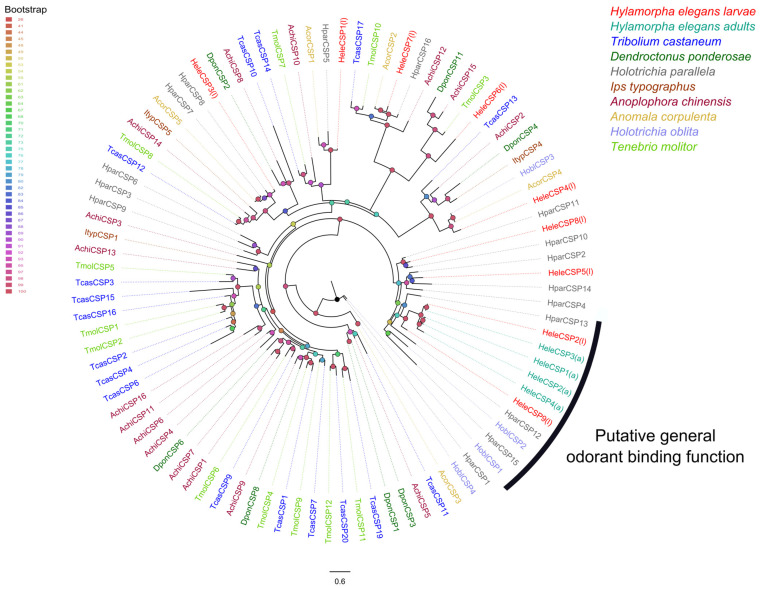
Phylogenetic relationships of chemosensory proteins (CSPs) from *H. elegans* white grubs and CSPs from other Coleoptera. White grubs of *H. elegans* were annotated with (l), while adults of *H. elegans* are annotated with (a). The bootstrap data are indicated by node spheres and coloured according to the percentage of confidence.

In terms of phylogenetic relationships, HeleOBPs are grouped according to the number of cysteine (Cys) residues, for instance, following the Cys pattern of classic OBPs (C1-X_25–30_-C2-X_3_-C3-X_36–42_-C4-X_8–14_-C5-X_8_-C6) [63]. Thus, HeleOBP4, 10, 13, 19, 20, 22, and 23 contain the six conserved Cys residues and are therefore grouped into two clades containing classic OBPs. HeleOBP5, 8, 16, and 24 contained additional Cys and a conserved proline and were grouped with other OBPs classified as Plus-C. Finally, the HeleOBP1, 2, 3, 6, 7, 9, 11, 12, 14, 15, 17, 18, and 21 contained fewer than the other six Cys residues. Therefore, these HeleOBPs were grouped into two clades classified as Minus-C OBPs along with other Coleopteran OBPs (Figure 8). For the CSPs, phylogenetic analysis showed that only HeleCSP2 and 9 clustered in the clade with a likely general odorant binding function (Figure 9).

### 3.7. Differential Expression of Soluble Proteins

**Figure 10:**
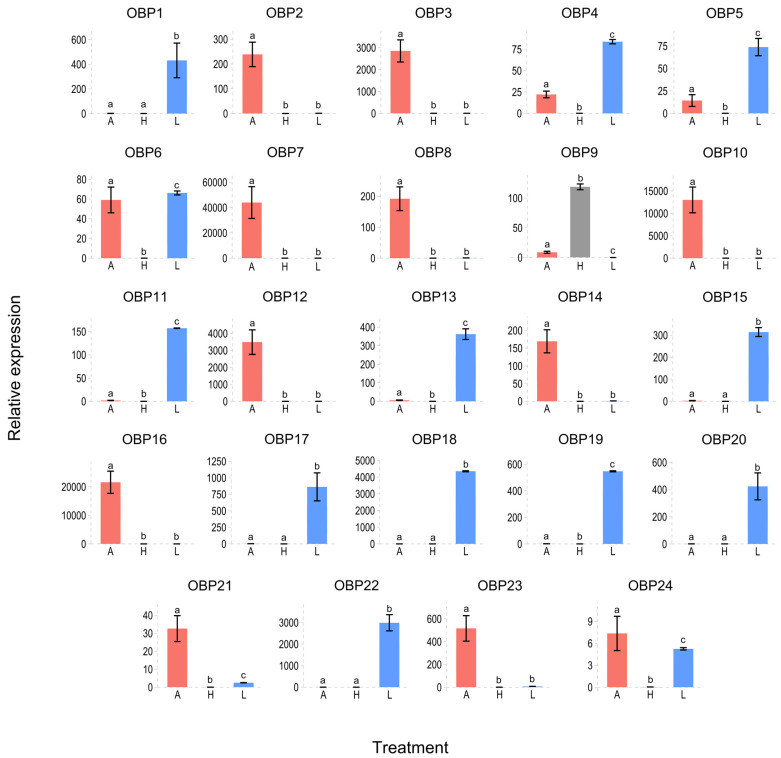
Expression levels of the OBPs in L3 of *H. elegans* white grubs. *H. elegans* with A, antennae; H, heads (without antennae); and L, legs were considered to be expressed. Different lowercase letters indicate significant differences (one-way ANOVA with Tukey’s test, *p* < 0.05).

Considering the differential tissue-biased expression (Figure 10), HeleOBP9 was expressed in the head (without the antennae), whereas the remaining HeleOBPs were not expressed in this tissue. HeleOBP1, 4, 5, 9, 11, 13, 15, 17, 18, 19, 20, and 22 exhibited significantly greater expression in the legs of H. elegans white grubs than in the heads and antennae. Finally, HeleOBP2, 3, 7, 8, 10, 12, 14, 16, 21, 23, and 24 exhibited significantly greater expression in the antennae than in the heads and legs.

**Figure 11:**
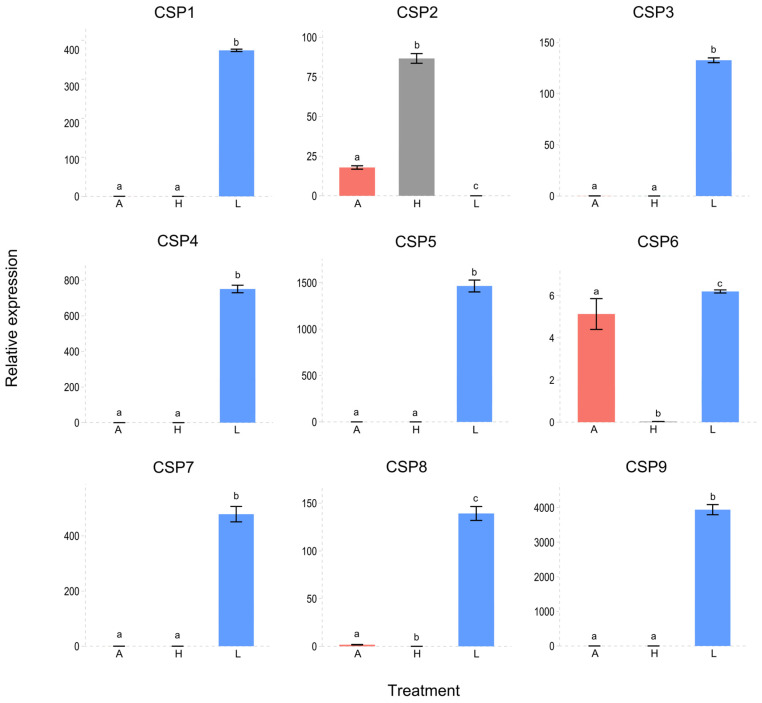
Expression levels of the CSPs in L3 of *H. elegans* white grubs. *H. elegans* with A, antennae; H, heads (without antennae); and L, legs were considered to be expressed. Different lowercase letters indicate significant differences (one-way ANOVA with Tukey’s test, *p* < 0.05).

In terms of differential tissue-biased expression, HeleCSP1, 3, 4, 5, 6, 7, 8, and 9 exhibited significantly greater expression in the legs than in the head and antennae. Only HeleCSP2 was significantly expressed in the head (Figure 11).

## 4. Discussion

The superfamily Scarabaeoidea comprises a large, diverse group of insect species, with approximately 31,000 species worldwide that exhibit common characteristics, namely lamellate antennae in adulthood and C-shaped larvae with developed antennae and legs [64]. Interestingly, it has been suggested that the ancestral larval habitat of scarabaeoid beetles is soil, adapted from a fungus-rich, humus-based diet to other food sources, such as necrophagy, coprophagy, fungivory, carnivory, herbivory, and saprophagy [64,65]. Particularly in the Scarabaeidae, a more general soil-based diet coevolved with a saprophagous/herbivorous diet with the rise of angiosperms [66,67]. Many of these scarab beetles are agricultural pests in both the adult and larval stages due to phytophagy [68]. In Chile, the scarab beetle *H. elegans* is considered an economically important pest of cereal, ryegrass, and red clover crops during the larval stage (also called white grubs), adversely affecting the production and food of cattle [43].

We believe that a developed chemosensory system plays a key role in *H. elegans* white grub behaviour, as has been proven for larvae of other insect species [26,28,69], especially in subterranean environments. For instance, it has been reported that the roots of red clover *T. pratense*, a host plant of *H. elegans* white grubs, emit volatiles such as ethanol, (*E*)-2-hexenal, hexanal, 3-octanone, limonene, and α-pinene [70,71] and other secondary metabolites (e.g., isoflavones) [72]. Furthermore, it has been reported that root exudates from wheat and other 100 plants (including ryegrass) can consistently emit jasmonic acid, salicylic acid, (-)-loliolide, and luteolin for signalling purposes [73]. Therefore, this study focused on identifying chemosensory genes in *H. elegans* white grubs. To date, 24 OBPs, 4 CSPs, 22 GRs, 14 IRs, and 66 ORs have been described in *H. elegans* adults via analysis of their antennal transcriptomes [74,75]. This study revealed 24 OBPs, 9 CSPs, 1 GR, 11 IRs, and 2 ORs in the head transcriptome of *H. elegans* white grubs at two larval stages (L2 and L3). Interestingly, the number of OBPs was similar in both the adult and larval stages; however, not all OBPs identified in larvae were the same as those in adults. This can be seen since 10 HeleOBPs are likely specific to white grubs because they were identified for the first time in this study. Similarly, the remaining 14 HeleOBPs identified in this study were also described in the adult stage, so these 14 OBPs can be considered to be involved in both stages. Similarly, the remaining 10 HeleOBPs previously identified in adults have not been identified in white grubs, so they could be specific to the adult stage of *H. elegans*. In the case of CSPs, of the nine HeleCSPs identified, eight are probably specific to white grubs, as they have been reported for the first time in this study, while one HeleCSP we identified has also been reported in adults. Notably, three CSPs previously reported in adults were not identified in this study; therefore, these CSPs could be considered adult-specific. There is a great difference in the expression of chemoreceptors between the receptors identified in adults and those identified in this research. The HeleGR1 gene identified in this study was not detected in adults, so it could be considered specific to the larval stage. For the ORs, HeleOrco was identified both in adults and in this study, while HeleOR1 is described for the first time here; thus, HeleOR1 could be considered larval-specific. Finally, considering the IRs, although the difference between adults and larvae is not as significant as that for the other receptors, of the 11 IRs described in this study, 3 IRs were identified in both larvae and adults. Therefore, the 8 HeleIRs identified in this study could be considered larval-specific, while the 11 IRs previously identified in adults could be considered specific to that stage.

Although the number of soluble proteins is similar in larvae and adults, the difference between the chemoreceptors of both stages could be key in the chemosensory system of this insect. Other authors have also reported a wide difference in the chemoreceptor profile depending on the stage of the insect. For example, in the moth *S. littoralis*, 51 of the 57 identified OBPs were expressed in adults and larvae, while 22 of the 47 identified ORs were found in both adults and larvae, and 25 ORs were adult-specific [26]. Similarly, in the *Aedes aegypti* mosquito, 123 ORs were identified, of which only 15 were larvae-specific, 9 ORs were found in both larvae and adults, and the remaining 99 ORs were adult-specific [76]. Although there is evidence that chemosensory genes are expressed in larvae and adults, a differential expression profile has also been shown according to the larval stage of insects such as *Plagiodera versicolora* and *Solenopsis invicta*, where in the initial larval stages, the expression levels of soluble proteins and chemoreceptors are lower than those in the last larval stage [30,77]. Considering this, our abundance data for the L2 and L3 stages of *H. elegans* revealed a greater abundance of HeleGR1, HeleOBP9, and HeleCSP2 in L3; this may imply that insects such as *H. elegans* need a more complete chemosensory system at higher larval stages (such as L3) because of feeding, pest status, and/or proximity to the adult stage of the insect. In that sense, antennae and legs are observable in *H. elegans* white grubs, and in accordance with developed sensory appendages in the larvae of coleopterans [78], this guided the relative expression experiments.

In the case of HeleOrco and HeleOR1 expression, as the life cycle of *H. elegans* progresses, its olfactory system begins to develop, and because Orco is a highly conserved ancestral receptor [68], it may be the first olfactory receptor to be expressed, followed by OR1. Considering the greater number of OBPs compared with ORs, it is possible that some OBPs expressed as nonolfactory appendages (i.e., 12 HeleOBPs) of *H. elegans* white grubs might transport or solubilise other relevant chemicals. Thus, it could be that the remaining HeleORs appear towards the adult stage, as reported [75].

Considering the phylogenetic analysis of chemoreceptors, the clades in which they are grouped suggest probable functional roles for some of these proteins. As expected, HeleOrco clustered in the conserved clade with another Coleopteran Orco [79,80,81]. These receptors have received particular attention due to their high conservation among insect species and their ancestral presence before the emergence of specific OR subunits [82,83]. The significant difference in expression between HeleOrco and HeleOR1 may indicate an important difference in expression during the larval stages of *H. elegans.* In other insect species such as *D. armandi*, *Bactrocera dorsalis*, and *S. frugiperda*, in addition to demonstrating expression at the larval stage, expression levels were also shown throughout the developmental stages, which may suggest that Orco is involved throughout the life cycle [84,85,86].

In terms of GRs, HeleGR1 appeared in a large clade with an expansion of GRs related to the bitter taste of *D. ponderosae*, which has been reported by other authors [87,88]. With this in mind, the development of GRs related to other functions, such as the reception of sugars (i.e., sucrose, glucose, fructose, and maltose) as well as CO_2_, which have been extensively studied [14,16,89,90], may be associated with the adult stage of *H. elegans*. Although our results do not reveal a conserved CO_2_ receptor, as described by other authors [31,91], it is likely that these GRs might be present in lower abundances. Thus, the present RNA-seq approach and depth criterion could not be used to identify these GRs. Noteworthy, it has been reported that CO_2_ could mediate long-distance plant-herbivore interactions [31]. In that sense, CO_2_-related GRs are crucial. However, field observations indicate that white grubs of *H. elegans* can move in the range of centimeters to reach roots or avoid insecticides [43], supporting less dependence on GRs to detect CO_2_ at long distances. Despite the above, it is necessary to consider that further analysis of the genome of *H. elegans* could provide clues to the identification of such key GRs and other genes. Likewise, further studies integrating functional assays and gene expression data for these chemoreceptors could shed light on their putative functions.

Few existing studies have focused on identifying chemosensory genes in the larval stage of insect species, and even fewer have focused on identifying such genes in Coleoptera. An example of this phenomenon is in *B. mori*, where BmorGR1 was shown to be differentially expressed in larval tissues, such as the larval maxilla. In contrast, other BmorGRs, such as BmorGR18, 19, and 63, were widely expressed in adult moth tissues and larval legs, antennae, and maxillae [92]. Another example is in the codling moth *Cydia pomonella*, where different abundance levels of GRs and ORs were detected; some of these receptors, such as CpomGR3 and 68, as well as CpomOR11, 18, 64, and 71, exhibited a greater abundance in larval heads than in adult antennae [93].

Phylogenetic relationships for HeleIRs suggest the possible functions of these chemoreceptors. Considering this, HeleIR25a appears to be a coreceptor as it is clustered with DmelIR25a and HoblIR25, which are highly conserved ionotropic coreceptors [8,94,95]. The presence of HeleIR68a is likely related to hygrosensitivity because it is clustered together with DmelIR68a, a reported humidity receptor [13]. Moreover, the thermosensing function of *H. elegans* could be related to HeleIR21a due to its phylogenetic relationship with DmelIR21a, the role of which has been described [96]. According to the expression of these receptors, HeleIRs appear to have a key function in the legs of these white grubs. It is known that in other insect species, the chemosensory system plays an important role in legs through the detection of chemical signals [97], the detection of sugars, amino acids, and salts [98], or mating behaviour [99]. Although IRs are expressed mainly in the antennae and/or head in most publications [100,101], some studies have reported that these receptors are overexpressed in the legs. For example, in the scarab beetle *A. corpulenta*, IRs exhibited a markedly greater expression in the legs than in other tissues, such as the female antennae, male antennae, thorax, and abdomen [102]. Similarly, the beetle *P. versicolora* IR9 was more highly expressed in legs, specifically in male forelegs, than in other tissues such as male antennae, female antennae, female forelegs, male middle and hind legs, and female middle and hind legs [103].

The function of CSPs appears to be well supported in Lepidoptera and Diptera [104,105,106]; however, in Coleopterans (e.g., *H. elegans*), the importance of these CSPs is still unclear. For instance, CSPs are found in both the antennae and nonchemosensory tissues of *A. corpulenta*, suggesting functions in female survival and reproduction [102]. These findings have also been verified in moth *S. exigua* [107]. In addition, it has been proposed that *H. oblita* CSP1 and CSP2 are responsible for binding general odorants with slightly different specificities [108]. In that sense, HeleCSP2 and HeleCSP9 might be related to general odorant binding due to their close phylogenetic relationship with HoblCSP1 and HoblCSP2.

In terms of soluble proteins, identifying a previously studied OBP from adult *H. elegans* larvae was of special interest; this OBP was identified under the name HeleOBP24 in this study. Thus, HeleOBP24 was reported to have a high affinity for β-ionone among 29 other host plant volatiles and putative sex pheromone components [40]. Similarly, HeleOBP1, 3, 4, and 6 have also been identified in *H. elegans* larvae, suggesting a host-seeking function in the larval stage since these OBPs have been reported to strongly interact with host plant-emitted sesquiterpenes [39]. Among these HeleOBPs, HeleOBP6 clusters with HeleOBP6(a) and OBP6 of the scarab beetle *A. corpulenta* (AcorOBP6), which are significantly expressed in the antennae, suggesting an olfactory role [102]. Similarly, Yin et al. determined that HoblOBP13 and HoblOBP9 are essential for *H. oblita* to receive plant-derived kairomones, (*E*)-2-hexanol, and phenethyl alcohol [109]. In this light, the close phylogenetic relationship of HeleOBP11 with HoblOBP13 could suggest a role in transporting host volatiles.

This study identified 47 chemosensory-related transcripts, 28 of which were reported for the first time, suggesting the presence of a well-developed chemosensory system for white grubs with subterranean habits. Notably, the methodological aspects of RNA-seq (sampling, biological conditions, extraction, and sequencing) and the lack of a reference genome could imply that additional chemosensory genes have yet to be identified. However, this study identified interesting candidate proteins, such as HeleOBP9, HeleCSP2, and HeleGR1, for further functional characterization. These chemosensory genes might represent suitable targets for both a deeper understanding of *H. elegans* white grub behaviour in soil and for controlling white grubs in integrated pest management. Future efforts will also focus on genome sequencing, combining short and long reads, as an efficient and cost-effective alternative approach to generate de novo chromosome-level genome assemblies [110], enabling more complete mapping of chemosensory genes for *H. elegans*.

## 5. Conclusions

The analysis of *H. elegans* larvae revealed a repertoire of chemosensory genes with some differences in the number of proteins according to the protein family, where a greater number of transcripts identified in chemosensory genes than in chemoreceptors was evident. In this context, the presence of more OBPs than CSPs may imply that OBPs play a prominent role in the chemosensory system of white grubs. In terms of abundance, HeleOBP9, HeleCSP2, HeleGR1, and HeleIR3 had higher abundance levels than did the other proteins, where they were also significantly expressed in a particular tissue. Finally, HeleOBP9, HeleCSP2, and HeleGR1 have attracted our attention due to their potential role in larval chemosensation and, moreover, in the phytophagous behaviour of *H. elegans* white grubs.

## Figures and Tables

**Table 1:** Summary of white grubs in L2 and L3 of the *H. elegans* transcriptome assembly.

Metric	Data
Trinity transcripts	48,112
Trinity “genes”	28,215
GC (%)	37.09
Contig N50 (bp)	2305
Median contig length (bp)	1061
Average contig. (bp)	1540
Total assembled bases	74,092,714

## Data Availability

The data presented in this study are available in the Supplementary Materials. The sequencing data generated from this project were submitted to the NCBI SRA database linked to BioProject PRJNA1070317.

## Supplementary Materials

The following supporting information can be downloaded at: https://www.mdpi.com/article/10.3390/insects15090660/s1. Figure S1: Ranking of endogenous genes; Figure S2: Multiple sequence alignment for HeleOBPs; Figure S3: Heatmap for DEGs; Table S1: Annotation of identified transcripts for ORs; Table S2: Annotation of identified transcripts for GRs; Table S3: Annotation of identified transcripts for IRs; Table S4: Annotation of identified transcripts for OBPs; Table S5: Annotation of identified transcripts for CSPs; Table S6: Primers used for qRT-PCR; Table S7: Read quality and mapping rate per sample.
