# Supplementary material for: Transcriptomic and Gene Expression Analysis of Chemosensory Genes from White Grubs of Hylamorpha elegans (Coleoptera: Scarabaeidae), a Subterranean Pest in South America
Source: Insects. 2024 Aug 30;15(9):660. doi: 10.3390/insects15090660 (PMC11432230; doi:10.3390/insects15090660)
Supplement: Supplementary file 1 [file insects-15-00660-s001.zip › Supplementary Information.pdf]

Supplementary Information For:

Transcriptomic and gene expression analysis of chemosensory genes from white grubs of *Hylamorpha elegans* (Coleoptera: Scarabaeidae), a subterranean pest in South America

Paula Lizana[1,2], Ana Mutis[2], Rubén Palma-Millanao[3], Giovanni Larama[4], Binu Antony[5], Andrés Quiroz[2], Herbert Venthur[2,*]

Ranking Order (Better--Good--Average)

Method	1	2	3	4	5
Delta CT	RPL18	ACT	GAPDH	TUB	EF1a
BestKeeper	TUB	RPL18	ACT	EF1a	GAPDH
Normfinder	RPL18	TUB	ACT	GAPDH	EF1a
Genorm	ACT | GAPDH		RPL18	TUB	EF1a
**Recommended comprehensive ranking**	**RPL18**	**ACT**	**TUB**	**GAPDH**	**EF1a**

Comprehensive Ranking | Delta CT | BestKeeper | Normfinder | Genorm

Genes Geomean of ranking values
RPL18 1.57
ACT 2.06
TUB 2.38
GAPDH 2.78
EF1a 4.73

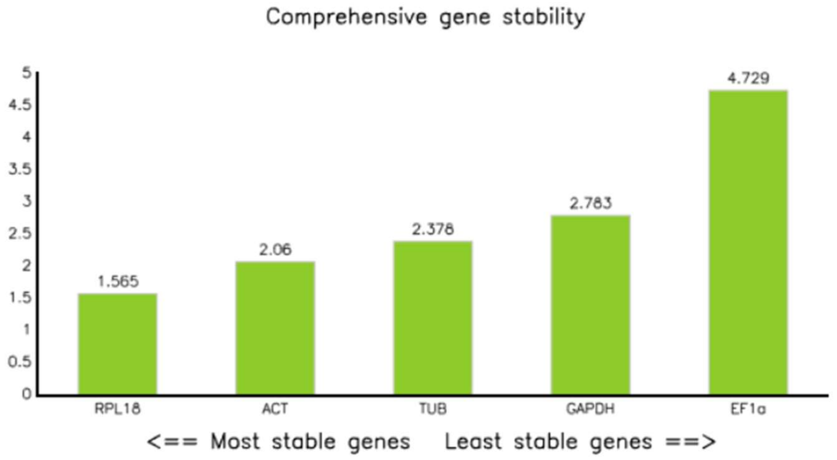

Supplementary Figure S1. Ranking of endogenous genes ribosomal protein **RPL18**; β-actin **ACT**, α-tubulin **TUB**, glyceraldehyde-3-phosphate dehydrogenase **GAPDH** and elongation factor 1-alpha **EF1α** using RefFinder (https://blooge.cn/RefFinder/).

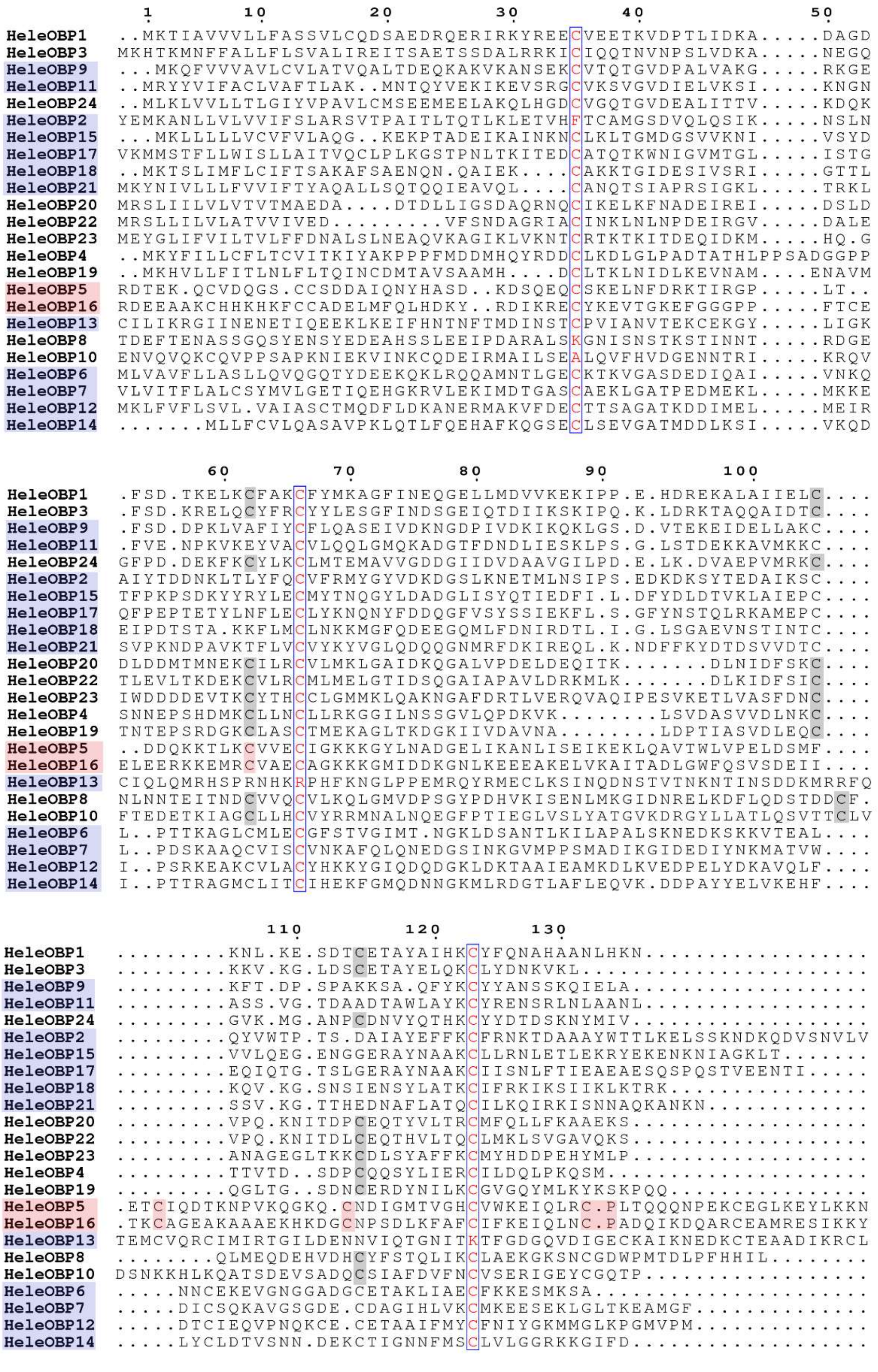

Supplementary Figure S2. Multiple sequence alignment for HeleOBPs showing the Cys pattern of classical OBPs (C1-X25-30-C2-X3-C3-X36-42-C4-X8-14-C5-X8-C6). Pink-labeled HeleOBPs were considered Plus-C, purple-labeled HeleOBPs were considered Classical, and the rest of the HeleOBPs were considered Minus-C.

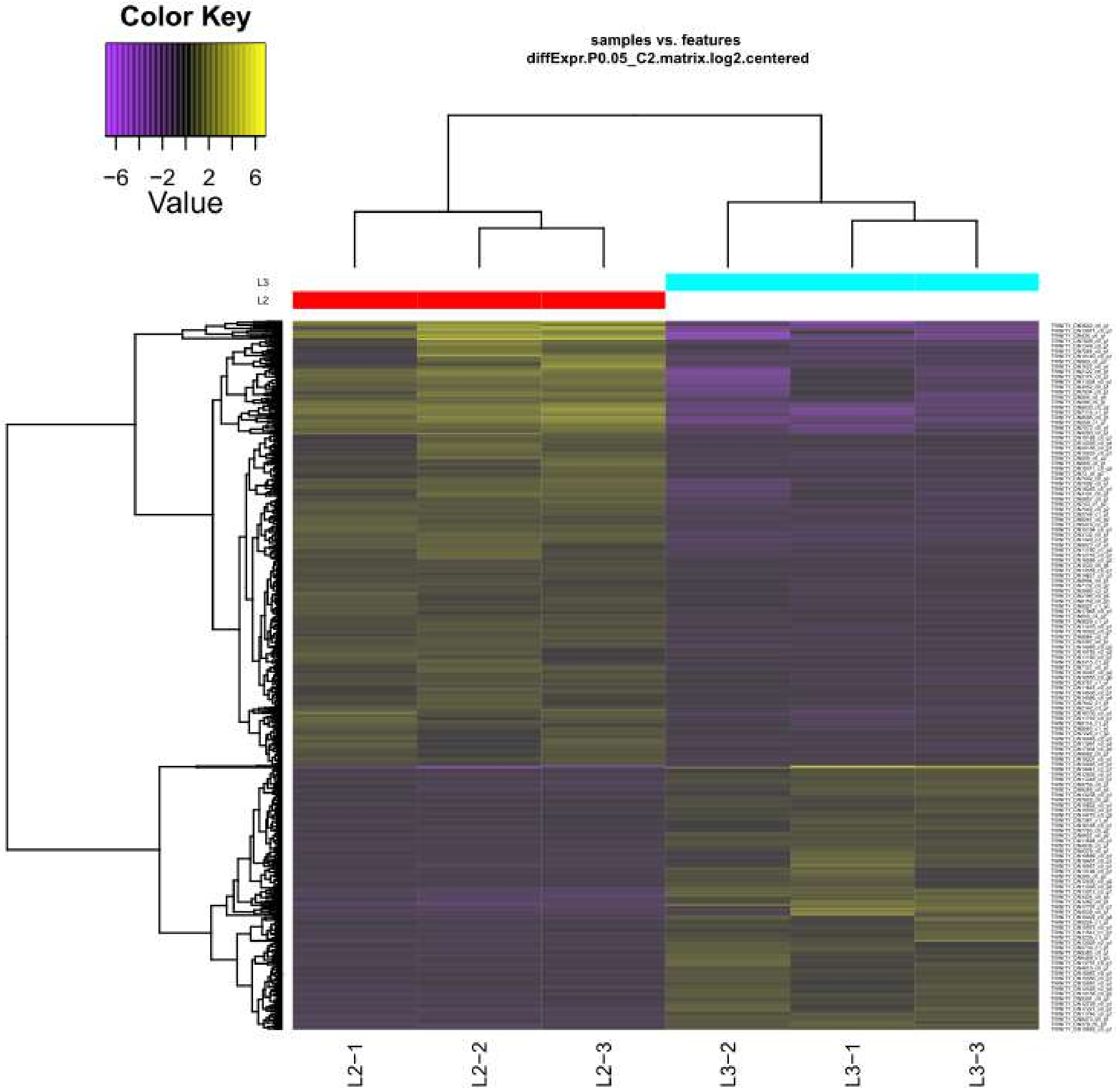

Supplementary Figure S3. Heatmap for DEGs.

OBPs sequences

>HoblOBP2
LTLHSITGFKMKYFVVFAALCAYVLGDTLQEHSQKVMEEIITVGAECAEKLGATPEDIDKL
MKEQIPDSR
AGLCVVSCANKKFGLQNADGTVNRGSTLVNIEKVKDIDNDMYKKMSSVWSTCSENTPND
SDECNTGVHLV
KCMKEEGAKLGLNKETMGF
>HoblOBP1
FPAMRSNIKMFKNSALFILGIILPSVLCMSEEMEALAKQLHDDCVAQTGVDEAHISTVKDQK
GFPDDEKF
KCYLKCLMTEMAIVGDDGVVDVEAAVGVLPDEYKDKAEPIMRKCGVIPGANPCDNVYQT
HKCYYDTDAKS
YMIV
>AglaOBP3
MKFAVAIVCLVVVAVARASLTEEQIQKLKGYHKDCAAETGVDTELVTKARKGEFSEDPKFK
DHLFCVAKK
IGFMTADGEIHRDVLKEKLGSAINDDAAAQKLIDECAVKKDTPQATAFDTIQCYYVKTPTHI
SIV
>AglaOBP16
MFPSVVCAVGSYLIFLTIIQAKEISSGRCDIPPSAPKKVEEIINQCQDEIKLAILSEALQAFNVN
EHSRS
RAKRATFSEDEKRIAGCLLQCVYRKMDAVNEKGFPTVQGLVSLYTEGISQKDYILATVQAV
NTCLIKSQK
KYLITPQSIDENGKTCDIAYDVFDCVSDEIGKYCGQRP
>AglaOBP15
MNNLVAFCALLVAAVSAYNFEDPDFNILLSDDLEELSSGVASFSHPRSRRDDEAVNDKDKCH
HRKRWGEL
CCAEDVMAKMRDVEKDLKRECFKEVVGKDKHEKFDPFNCETMDQRKKQIVCVIQCVGQ
KKDLLDTEGNPK
EEEFRSFLKESFSSESWLAALQDKVISTCLDEGKNATANRDASDSTSCNPAGIKIAHCLHREI
QLNCPAD
QIKDEKSCARLQERLKRRDFFHPPPPPGAFDEPDN
>AglaOBP14
MKFCFFLLCSCLAAPYVYSAMTEKQLNAAKKLMRNTCQNKAKPTSEQIDAMQKGDFNGD
RNAQCYLLCIL
STYKLLTKENTFDWENGIKALAANAPASVAGPGSATLKNCKDAVKTPSDRCVASTEIAKCIY
DDNPSNYF
LP
>AglaOBP13
MIVPTIVILVAVILVMDVDADLTDKLRQKLDLPEVQKCITSTGYIPKGPPEGPSQEFTPEQLCF
FKCIME
EKGLLDSTGNIIQDELNNVPLPIPDDKKNEIKKCAAGAGKIESCEDIQKLLSCLPM
>AglaOBP12
MSSRSIIFFFCLITLAYSKLQLPPDLQEYADELHDLCIKRTGITEDDHIAYDIANNPHDEKLQC
YIKCLL

MEANWMDKDGVIQYDWIEENIHEGVKDIVLAALRKCKNINEGANLCEKSSHFNACMYDA
DKENWFLV
>AglaOBP11
MKTVFVISMVVIMAISHAEMDKEHQECLDETELTEAEVSEFLLGDDAENDAKATKFLMCIF
KKKEAVNDE
GHFDVTKAQEVVKHYMTEVVGSEDQQALDCVQEKDTTEQTVLALGKCVEKRKVELSSSK
>AglaOBP10
MRTFVVIISISALVLGRPDENLATINMAHNECQSNPRTYVDEDILDRISGGEKIDNPSVRAHIL
CVTTKL
GVLNEYGEVNRTNLRTVLSRVILNEEKLEENLEKCAVEETDAEEVALVLDKCFWNNLDHDH
NSHIHYHHQ
KT
>AglaOBP9
MVSLNFFVTVSSLFAASVVQAALPQSEYGPQLEALQKNVRAACISTSGVDETAISNVGNGV
FTDEPKIKH
YLTCVLKEGKLVNEKGVFSEKNIAQLFPDKYKEESLTNIKSCIVKVNDITNLEDKIFALFKCY
YHQNPDL
FVFF
>AglaOBP7
MRTCAIVVCIATLVVSIHCASEEQHERVKKIHSECQADPKTHADDELLKKYHKGEEVDKSIV
GAHMLCMS
TKFGVIQEDGKINKSALKTSLSRLISDETKLNEAIEKCAVEKDDPKDTALALGKCFREQGGL
RGHEHIHN
RL
>AglaOBP6
MKVLALIVCVSVFCGLVIGTAIDRDLHKECQADKATHLDENIMKALDEGEITDRAKVGAHL
LCISTKAGV
INQDGSINKNMVKEKFSRYYSDAAELERITNKCSQQGTTPVETALKLAECINEYSLEQ
>AglaOBP5
MKSFLLVAFVVGVVTAASLPASEKKFINQVHANCQSNPKTFVDESLLKNLPANKDNAQVG
VHMLCMSKGA
GFQGPNGDINKETIKSKIALVIRDGSKVNELTNKCAVKTGSLEKTSINIFLCLNSNQVPYTPD
LN
>AglaOBP4
MKIFASFLCVFITCALVYGAPSIRDIHGECQSDPATRLDHDEFKAVRTGESFDRTKVGAHMLC
MNKKFGT
QNADGTVNRNAVKEVLAQDITDETKLEEITNKCVEEGSTPSETALKLSKCVSENTKGGRHG
HGHEHHHGH
HHEHHHDH
>RferOBP10
MSSLTELVVLLAVLSISIAKFDDSIISEDIRKLLKGLHDVCVSKTAVDEVLIEKLKDAEFTEDQ
KLKCYV
QCLLVQTGSMDLAGHIDIEAAVELIPEQIRNAVIKDVNKCAKDSEQVAEHCDRAFATLKCLY
SVNPDIYY
VF
>RferOBP9

MKKCGVIVVLCVLILINDVVARMTEKQLQAAVKLVRNMCTGKTKATADDIDKMHKGDWE
VDHNAMCYMWC
SLNMYKLMDKNNRFDRKSADAQLAQLPESMQKYVNKCIGQCENAATHFDDKCYAAWEY
SKCMYFCDPEKY
FLP
>RferOBP8
MGLLFFFLTLLILSINNGDCAMSEAQVKAAKKLVRNACIPKSKVTEEQVDGMHDGMWDLD
KRGKCYLQCV
MNFYKLQKPDNTLDWEAGIKMMETQAPPSLAPHGIKCMKECKDAAKTLNEKCTAAFEIA
KCIYDIDPAQY
FWP
>RferOBP7
MNGLLKVSVLVIVVSAISCQEFTEEQKKKILENRKQCIEETKVNPELIEKADQGNFVDDNSL
KCFTKCFY
QKAGFVNDEGEVQLDVVKAKLPPQADKEQALAIVEKCKIKGKDACDTVYLIHKCYFEHTH
PELFKKDEPK
KEEKKA
>RferOBP6
MFYSSLILLNILASVLAYGTMTPEQRQRFLTFQGECMMESGTTEEMLLKAFMGEFTESSVFK
DHLVCLGM
KTGVIDDEGNYHKDILKEEILSFIGDETKVDDILDTCYIHYDTPQESAFNMMKCMFKEHFG
V
>RferOBP5
MFYFSLILLNILASVLAYGTMTPEQRQRFLTFQGECMMESGTTEEMLLKAFMGEFTESSVFK
DHLVCLGM
KTGVIDDEGNYHKDILKEEILSFIGDETKVDDILDTCYIHYDTPQESAFNMMKCMFKEHFG
V
>RferOBP4
MCRFTAILLISLCGLIYGGMTPEQRTRFFNFQNECMQETGATDEMVLKAFAGELTDSPVFKD
HLVCIGMK
GGVIDEQGNFHKDVMKKGIMLFVDDEGKVDAMLDKCYTHYDTQQDTAFNMMKCMFKE
HFGA
>RferOBP3
MEKLFILLLQLVVLFVVVAGELTADQRKQFIVFQNECMQETGATDDMIMKALAGVFSDSPV
FKNHLVCMG
LKTGIIDSDGNFHKDILKKGIMLIVNNEAKVDAMLDKCHVYFETQQESAFHLMKCLYEEHF
GA
>RferOBP2
MNNLVVLLLYIGYFLVQADLTPQQREELFKVQIECMEETGATDDMIMSAFAGNFSDDPIFKE
HLVCIGRK
SGVIDDEGKYHKDLMKQGLMTFINDENTIDKMLEKCYIEQDSVQELAYRMTKCLYNEHFG
L
>TcasOBP16
MQLLVVVLAVCVLGANAGLDPKFLEKLTQEVQAVGTSCGEKEHATADDMIEIMEEKFPPTS
HEAKCVVAC
FYKHYKMMKEDGTFDKDAAVKAFDEIKAQDAEIHAKILKVIDACDAKKQMSDDHCVSAA
SMAGCVKTEAI

ANGLTKEAFMAS
>TcasOBP26
MMHLKNFVVLVVCPLFVFAKVEIPPDLEAEIDEYFEQCFEPNGVTMDDIKAYKMGDKDPKI
MCFMRCLFV
SGKWMDENENMQYDYIKETIHHAIRHITIPELENCGKEAQTGDKCEKSFNFFMCMNRAEPE
DWILDYKS
>TcasOBP25
MPLKNLIILIVCPLFVFAKVDIPPDLQAEIDGYYDICYKQIGLTKDDLKAYKIGDRDPKIMCF
MKCVFVE
AKWMDENENLQYDYIKNTIHHSIRHITLPELENCGKKAEGDKCEKSFSFFNCMNKAEPED
WVLIQ
>TcasOBP8
MIRYYIVLLLYFFAPPVLGISEEMQELVNQLHSTCVAETGVSEDLINKVNSDKVMIDDEKLK
CYIKCLLT
ETGCISDDGVVDVEATIALLPEDMKAKTTPVIRSCGAKMGANPCESAWLTHKCYLETSPAD
YVLI
>TcasOBP20
MATRFCFGLLILFVGTVLVFAENEHEILEVRALCMNETGVSEETARNYKPAEDPASEEILCM
VKCIFEKI
GCLKDDGSFCVDTMKKKNYIMDVINEENEEKIYECLRGVGKITNCRDMAAVEECFVKNDS
K
>TcasOBP22
MKPIFAIITLTLCTTVHALDCGIHINKNDALKATINKCLISNKTLEDLWDMAPMSSESDSSSEE
VPPVDG
KMLQNFRIKRASVRLTNTETNETTPEPKAVSSEAQATENCIIQCIFDNLQMTDSTGYPVHTKI
LDGLLKN
TTNRELRDFLQDTTDECFQVMDKEDTMDPCSYSNKLVTCLAEKGRSNCADWPVGELPFKP
>TcasOBP15
MNCFVIFALSLSATVFGQSLSEDEMRENARKLMTSCKDKVGASDADVEALKMHQMPESRE
GFCMLECVFD
SAKIMQDGKFSKSGMIEGFKPLIGDDKAKLESLEKLSATCESELGDGEDKCETAKRLVECVI
KNGKTHGF
EVPPPRE
>TcasOBP23
MKYFPHLCLCLIFFELSEAAMSEAQLKAAVKLVRNMCQPKSKATNEDIEKMHHGDWNIDR
TAMCYMHCAL
NSNKLITKENVFNRDYAITLAEKNLPTALKTASIEAANLCKDSAKTLDDKCVAAYEISKCLY
ESNPEKYF
LP
>TcasOBP9
MKAILLLLLVATLSFYHVYCAMSEAQMKAALKLVRNVCQPKTKATNEQIEAMHTGNWDLD
KNGKCYMWCIL
NMYKLIGKDNSFDWEAGIATLKAQAPESVRDPAIASVNNCKDAVKTTSDKCEAAYEIAHC
MYLDNPEKYF
LP
>TcasOBP2

MNFVCVIFILVAIIGAHGLSEQQTEKLNQLSKECRALTGVSQETITNARNGNFEEDPKLKLQV
LCIGKKV
GIMNESSQIDENVLKAKLRKVSDNDEEVNKIYNKCAVKKPAPEETAFETIKCVMKNKPKFS
PVE
>TcasOBP10
MKTVAVLLFLALAACTKQEDDDRQETIRQYRDDCIAETKVDPALIDRADNGDFTDDAKLQ
CFSKCFYQKA
GFVSETGDLLFDVIKDKIPKEANREKALAIIDKCKELKGADSCETVYLVHKCYFLHSYGTD
KKTE
>TcasOBP5
MKFLVVISTVLMANIVQGLTDEQKSKLEEYSKECLKESKVDESVLKEAEKGVYLDDPKLM
NHVYCLVKKI
NSQKDKGELEVTQIKEKLMMQINDEKEVDKLIQLCLVQEKSARYSLGKCEVSS
>TcasOBP21
MKQIYCLITVVVLIPTLTALENEGQNPDTANCVALGGQRIKDSEIAKMAHCILTKTNLMTDK
GTFNSNLL
KERLRQSVHSDELVDKVVMMCTVEKETPLKSAFSGYKCLRYLVPWFPLD
>TcasOBP4
MCRLFVVLSLFVASQALDVEKIRNELMADKNFVELRNKCLDKLGLKEEDLRDLKFDGDVS
EDLMCFGKCI
QEEDGLLDSEGNLNEEKLEKKIETMPFLSRVSDDTKNNIMECLKEIGKIETCQDFGKQRDCI
HKYV
>TcasOBP1
MILKASIFLILAVATFGAILEDSELMKVVENCVKKTNANESEFSSPNFLETTPSQPALCTAKCL
LESLEI
VNSEGNINMETLKEYAQPFESPAREAVATCGEEIKSVTTCDDMEKYRKCVEPLIKNS
>TcasOBP3
MWSFVTLLFSFLVLASAQKKGKYWTTISECLTEHSMGVEDMKKFDLPAEKMSEEMLCFNK
CFYDKLLITD
ENGEINTDNLMSIPLVNAIDASKHDDLVTCLKKVGKIEECDGVKKIEQCFVEFI
>TcasOBP6
MSPLLLIFISCLFPRVFGISEEMQELANTLHATCVDETGVSEDAIESARKGNFAPDDKLKCYM
KCIMEQM
ACIDDEGIIDVEATIAVLPEEYQAKAEPIVRKCGTKIGANACDNAFLTNKCWYEEDPEDYFL
V
>TcasOBP7
MYKTRVIYVLFALCLVEIFAIEMDDDMKELINNLHNTCTGETGATDDQIENARKGNFAEDDS
FKCYFKCV
FDQMGCMTDDGKVDSEAVIAVMPPELADKIASTVRGCTEVGANPCETAWLANKCYQKSNP
DMYFVP
>TcasOBP19
MKYFVVFASLFLATNALSQDFIDKFVAKVKSIGETCVPETNASKDDISSLLAHKMPDSHEGK
CLIFCFHK
QFQIQNDDGSINREGAIKALEPLKADDAELYEKVISIFKKCESTPVDGDSCLYAASLAECAVK
EGRAMGL
DNLIVLEIE
>TcasOBP14

MNSVLFLLVCALVACSGELDKEFLMQFLQKIKKVSEDCIAETQATKNDIKTLLEHKIPDSHE
GKCMIFCF
HKHFQIQNEDGSLNKVAAISLLEPIKDHSQDIYDKVVKIFNTCFDSAERDDDSCIYASNLAEC
AIRESKS
LGLDDLLVIE
>TcasOBP17
MKSTWFFLLLACSLTCALDQEFVDEFLEKMQEFGAQCAEETDATSDDIAELIARKLPPSTHE
GKCMIFCM
QKKFNMMKENGGIDRAGAIAALKPLQKADPELHQKVLKIFVTCGMRVKPSPDPCDTATEL
ALCGKKEAEA
IGLEDALLT
>TcasOBP18
MKLFILAGILFTGVCAVDQEFVEKFLQKMEKIGEECAEETHATSDDIADLIEQRDPKTHEGK
CLIFCYHK
KFNTMKEDGSLDKVGSVLALEEVRDADFELYKNILTIFVTCGDKAKIYDDPCETATALTMC
GRDEAKALG
LQDAIFG
>TcasOBP13
MKFLLVFLSVAILCTFAMDESFLQQTRDRVKAIVKECVTEEKATDSDFDDIMALKIPTSHEG
KCVFFCSH
KKFNMQHPDGSINKEGALDTFEVVKDVDAEFHDKVITVYNHCLSTPVDPDPCVYSVNLFQ
CFMKEAKAAG
IHELIIK
>TcasOBP12
MKLLITLATLVVATYAIDKEFVQELRQKLRSHVEACAKEVNAGPDDVSAIFAHKLPATHEGK
CIFFCMHK
LYNAQNEDGSLNMAGALANLELIKDMDPDVYTKVSTSFKNCESAPFDSDPCLYAANLVTCI
VKEGRAVGL
DEVLVE
>TcasOBP11
MSFLILLICVIPAIFCRSFSHDELDTDLSFIKTCNRTSPISMRTMNEVLINKKLGHGESSAFKCF
LHCLF
MKYGWMDSDGGFLLHDIKQTLEESDVEIASLEFILYKCTATESNNRCERAFVFTQCFWDKM
AEQQPSEDQ
FFYNIEDKK
>TcasOBP24
MSRMLPAALFVVLATLTFATAEIVVPDDLKDYINELHDHCLKEMGLTEGDHKNYNIHVKDP
KMMCYMKCL
MTTSKWMNMDESIQYDFILSSVHPAVKNILLPALDKCRDIPKGTMECEKAYNFNMCLFNAD
PENWFFI
>HoblOBP8
MMKLQLVLVLLLGLCLQVSSAINEAQMKAARKLIRNTCRTKTKISDEQIEDMHKGIWNDD
DDVTKCYTHC
CMGIMKMQNKHGGFDRELAAKQYPQIPESMRESLVSSLEGCADSGEGLTKKCDISYAFFKC
VYFSNPEEY
ILP
>HoblOBP5

MKFFVLLSFIAFANCMDQDFLTKATARMSQYVEECAKDAGATKDDLAELMEIRIPSRKEGK
CLLACYNKK
YGIQDKDGKLDKEASIEAMKDLKLADTELYDKAVKLFDTCIDQVPNQDCECNTAAIFMYCF
NINGGLMGL
QPGMVPM
>HoblOBP14
MKFVIVLLLSALAFINAYDFEDHAYNELLVEQYSLAHPRLRRAAENNCIDSRSCCYNDAVPQ
YHANETEE
CSKELGFDRSQIRGPLTDAQINQIKCLAECISKKKGHFDADGNLLKDELLKDIRKPLEQVAW
LKPKVEDI
FNKCLDGLPTKVPENQCNDIGMTIGHCIWKEIQLECPKDRQANLEKCEALQVYLKENKAFP
PPPPIRC
>HoblOBP24
MEQIIDYATSCADTLGVSPEDIKLLMEKKLPASKEGQCIPSCVNKKFGLQKADGTIDKEYKN
SDMEKVKA
IDEEAYNKMNSVWEKCVLNGAEGSDECDTGIKVVTCMKEESEKVGLNKEAMGF
>HoblOBP28
MLVDGIEKLELDAWDNENNAKYDNGEANQESRYNKELFKTCCEEPSFLFLQYHFEGIGKEC
YKKLERHSH
VHNSTKRPGNDTRTNTCIAECIAKKLDLINDGGFASKIKIHKLLTKAFSNVDWFLNEFNIHG
WGCIDQKQ
GNQTDCNTVPIKAAYCLWRQIQLRCPMDRIKDQIKCRKIKRKISKIDALTEKEEKETEPRD
>HoblOBP27
MKFIVLVLSALTFINAYDYEDYYFDDIIMEQFELPHGRLQRNMENKSTNGDSCCNKFFKPRS
PSTKKEEC
LKELGITKLPKLSKITEEQQQQVKCYYECRFRNHIDANGELFRAKQPQLRKIYKERMEWLA
PKIDDIFDK
CLTNLKPAEKVENECNDIGLRFTYCFWREAQLECPLDEQVNPEECQVLQEYLKKHNHILPT
P
>HoblOBP26
MKPDTSLVIVLMFAFVIIECESIPVLAKNVSQINEECSKQTGISNDVAKNLTSTQQMPKPSETY
YKFLEC
LYKRQNYFDDDGNVSYRNIENFLSNYYEKSILRKAMAPCEDLQVGTSLGERAYNAGSCIIK
NLVTIEAQE
DGESINSSEESANDI
>HoblOBP25
MEKIILLMCILSLEDVQAFSSDNQNQAITKCIQKTGVDESIVTRMGVTLDIPDNSVVKKFLA
CLNKKLGF
QNEEGDMLFDNLRGNLVGMSSEEVNSTVSTCKQVKGTNSIENSYLVTKCIFRKIKSIIKLKK
RK
>HoblOBP23
MTKVVLLFIVLIVAIQAKKKLSADEIKQINQDCLKSSGMDSSVIKNIVSYDTFPKASDKYTK
YLECMYVN
QGYLDKDGLISYETIEDFILDFYDLDTVKLAIEPCVVHQDGKSGGERAYNTAKCLIKNLEKL
EKQHEKEH
KDITGKLA
>HoblOBP22

MKSFIVLFALIVISTALTDEQKEKIKTIGKQCKASSEVEREKLEKLRNGEIENDPKLKAFLRCT
FEKLEF
QKPDGTIDKEKVKSRLPSKLSETEKEEIVNECTNVTETDVTEIAYSVYKCYRSKTTAHNADL
FL
>HoblOBP21
MKQFVIVVALCVIVSVQAITDEQKAKVKEYQTACVASTGVDPKLVEEGEKGNFITDAKLRE
FISCFLKKT
GIQDEAGEIQASVIKQKLANDYTEAEIDAVLAKCKSSGGTPAEVAAAFYKCYWANASKHVP
LV
>HoblOBP18
MKFLILLSALVTFITCTDQNFLDKANEKMTKYLQECAETTGATKDDMMELMEIKIPTRKEA
KCLLACYHK
KYGIQDKDGKLDKVASIEAMQDLKVADPELYDKAVKLFDTCIEQVPNQDCECETAAIFMYC
FNIYGQMMG
LKPGMVPM
>HoblOBP17
MKRMILLISLATFVISEKQDLIQYGEKCAEELGLGPSDLSEFKEKILSRAEMNHNSKCMARC
TAEKAGLI
VDGVLNEAAVSKCLPDGVDLEKCKALTGDDDCDKFYKIFRCLDKEKLEKSTM
>HoblOBP16
MKTIALVVLFAVLVSCQDSEEKRAERVRKYREECVQETKVDPALIDKADAGDFADTRELKC
FAKCFYTKA
GFFSEKGELLLDVVKTKIPPEHEREKALTIIELCKDLKGSDACETAYAIHKCYFQNAHAAQL
HKN
>HoblOBP13
MKSIIVLFALIVISAGLTAKEEEKIKEFAKECRTSSGIEREKLEKLRNGDIEDDPKVKDFISCLF
KKLSL
QKSDGNIDQDIFKSKLSSKLTEAEKETIINDCINLKGSNDSETAFLLYKCYRSKTPSHNADLL
V
>HoblOBP11
MPLLCIGADDPDKEKLREAAKTIMYDCKDKVSASDADVQSLLDKTLPTTKEGACLLECIFT
TSKVMKDGT
LDKDATLKTLESVLKKDKDKEAKMTQVLDACQKEIGKGTDDKCQTAKMIAECLQKQGKL
AGLGPAS
>HoblOBP10
MKEIVLYFFILILMKANCLNQPNYAYECMAELGVSWSQMEEFEKSNPPHNIKCLSLCMGEK
SGYITNKQI
NETAVLEELPSGVSIDFEQCKDITGIDDCDEFFKILECIREQFAKSMDDLKKT
>HoblOBP9
MKISLFCFALCVAIVNASIEKYIDQLTTEIATAKTECAKQVGASVDDVVEVSQGKTPTSKEG
KCIISCVL
KLFGGQDSNGKINKHAVIGKIEELKPIDTDVYEKFSSVWKSCSEKTSDDNDGCDSAAKLMI
CLAQEVDKH
GISKKLIGL
>HoblOBP7
MYYIVFLVFLTVDIALTQLIMPEYDKEQSQCVKELNLDADKIQKLSQAEGEITKDEKCVLRC
VLQKIGVI

DAQGVPIQDEIDKQFSKFKNIDMSKCIPPSSITDPCEQTYKLTTCFLSLLEAAA
>HoblOBP6
MLPVVILYFCLIGADSATQLHELFNTNAMKMGFQCLEEVGATEDTLQKIANREIPTSREGMC
LITCIHQK
FGMQNSDGTTNRAGTLLFLEALKDDPFYSQTKDHFMECLDTVSNDDEKCVIGAKFMECLS
IGGMKKGIL
>HoblOBP4
MTMFLYFLIVNIFLVETTQGAMTNAQIEATQRMIRRTCKSKMKITSDDELDGMLNGKWDNL
SPATLCYLH
CCVKMIKMVTGDGHVDYDSNVKQINNLPEPKRHPLTDSLNNCKDAGKSLTDKCEIAHEIC
KCFYFSNPGA
YIIP
>HoblOBP3
MMKVPLVLVLLAGLCLRISCTITDAQMKAARKLIGNTCRTKTKISDEQIESMHKGIWPDDD
EVMKCYTHC
CLATMKLQSKDGGFDKELAEKQYPKFPESIKESLIESLEKCADSGEGLTKKCDLSYAFFKCV
YFYNPEGY
IFP
>DponOBP3
MHCSRACLIVFFSICGLSSSLKITLPPELQEYVDDLHKLCLEKGGLTENDHQTYDINHKNEK
MMCYMKCL
MLESKWMKSGGEIDYDFIETQAYPEVKDLLLNALNKCRTIEEGADLCEKSYNFNKCLYDA
DPVNWFFV
>DponOBP6
MKSIVALFVCALTATALADAEINETAFKAGRDRIMAMSRTCDENPATAVDQKALKKYLQSN
GPAPANGAA
HALCITKNLGWQNEDGSVNKPVITEKVKAIFGSVDAKVQQYIEECTEAKATPEDTAEQLLK
CYRKHSPKI
E
>DponOBP2
MKQLVMVVLTALCVVHCKGLECGLSKISSEHFRKIASECVKDNETLNRIWELTSETSMEED
SVSSDEEVP
VTKGREAPNFHDLGSSAHRNMKMSGASRTKRSRKGFNNESPMSNVQKKSSPASTTTEHTT
TMQSEENEEN
AAANNVEESGEVCILQCIFEKLEMTDTNGLPDHKKVASALVKSASGRETQDFLQDSVDECF
QETEEGDFE
NSCEYSTKLVTCLAGKGKSNCADWPVGDLPF
>DponOBP21
MALTTWVLSIMLILPAIRALSDEMKELAQMLHNTCVAETGVNEDFIQKVNAEKIFADDENL
KCYIKCLMA
QMACIDDDGIIDEEATIAVLPEEYQALAAPVIRACGTKHGANPCENAWLSHRCYAEMEPSA
YMLI
>DponOBP20
MKVFVVLCIVLFAFTLIVSAKKNKSNDEEKAKSYKKVFKECQKKDETRVDASIIRKLKKHK
QVDLPANFG
EHKLCVFTGIGLLKADNTVDEDKLKKKIASAKPQKDIVDNIVMDCTSSKSTLQETALNLDK
CLTTYSIEF

>DponOBP19
MKAMFVTLTVATVVVFASADLTEEQKQKIVANGKACVAETGADPELIKAARQGKFADDAK
LKAFALCMSK
KSGFQNEAGEIQSDVVKQKLGLAIGDEAAAKKLVEKCLVSKGSGEETAIETFKCYYENTPT
HIAVF
>DponOBP18
MNGFSVFFLLLLAAVVKSDFDFSNYKEFENLAGDQREKAIKLFKECMAETGATHEMMEKS
VEGDIPDDIV
FKNHLVCIGKKSGFIDENGMHIKEKLKEKLTLLLGNEELVDKILDKCFMEKGSPQDTAFELA
KCCHREYH
N
>DponOBP17
MQVTMNQGWFLLLVSVVSVFAELDQTSLPPEAKELMAALHKNCIEQVGVSEADVDKLRA
ANFEEDANLKC
YTRCLMAESGVMDENGAIDIEAFGEILPEAIRGNIQAIFRSCSLTKNDIVDQCVKAYEMVKC
WHKENPES
YFMI
>DponOBP16
MKLMWILVLGAALKSTEGAMTEAQMKAALKLIRNVCQPKNKATDAQIAAMHNGDWNQD
KNGMCYMNCVLN
YYKLQLPDNSFDWETGLKVVESQAPPSMAGFIMETIKSCKDAVKTGDDKCKAALEITKCLY
DQNPEKYFL
P
>DponOBP15
MGTTIFLLVGLFMMTNAYVPNVNDKIRDFCIDDSGVSIEMVENLLANPEKELIDVESCYVH
CIFTEMGLL
SENGNVEIENFKSLKASEAPYIDLNCLEEIKSIDHCNEMMILRACHV
>DponOBP13
MSNLLKLSIAFAVVSVISCQDFTEEQRKKIIENRQQCIEETKVNPDLIEKADLGDFAEDQALK
CFTKCFY
QKAGFVNDKGEVQKDVVEAKLPPQADKKRALEIVDKCALKGKDACETVYLIHKCYFEHT
HPEADEKTAKD
GKSEEKKA
>DponOBP12
MHFQWLTNVSVFLCILGVAQLVAAGKPNDLFTRITPGDVEVCGKDTGVDRKDFEEAREKG
ALNHSMLCFL
KCAMEKAGFLKDGHLEIDQAKEASPDKMTEPVVECFKAVGPISTCDDIQKVENCLPGS
>DponOBP10
MQLLFVAVLVIALVQVNSLTDKQKELLTQHYNQCVAISKVDQAVLQKARAGDFANDPNLKT
HIKCISEKI
GFQGTDGKFRRDVIEKKLKETLPGDNAKNAKLIETCVVANKDPQLQAFNAFKCLYTNAKIN
LL
>DponOBP1
MLTKTILIWAAILLTVFISTGNCRLTEKQVAAAVKLVRNMCMGKSKVNPEDIDKMHQGNWD
VDYEAQCYM
WCGFNMYKMLDKENHFDKKSALQQMEQLPTDLQDYVIKCMGQCENAVTNFDDKCVVAF
EYSKCLYFCDPE

KYFLP
>DponOBP4
MKLMWILVLGAALKKADGAMTEAQMKAALKLIRNVCQPKNKATDAQIAAMHNGDWNQ
DKNGMCYMNCVLN
YYKLQLPDNSFDWETGLKVVESQAPPSMAGFIMETITGCKDAVKTRDDKCKAAVEITKCLY
DQNPEKYFL
P
>DponOBP5
MPVTMNQGCFLLVVSAVLVFAELDQTSLPPETKELMAALHKNCIEQIGVSEADVDQLRAAN
FEEDAKLKC
YTRCLMAESGVMDENGAIDVEAFAEILPEAVRGNIQTIFRRCSLTNKDIEDQCVKAYEMVK
CWHKEDPES
YFMI
>AcorOBP2
MKSLVVVFVLLGLYSPVILGETLKEHGQKVMEKIIDYATSCADSLGVSPEDIKLLMEKKLPE
SKEGQCIP
SCVNKKFGLQKADGTIDKEYRNSDMEEVKAIDEEVYNKMNSIWDKCVLNGAEGSDECDT
GIKLVSCMKEE
SEKVGLNKEAMGF
>AcorOBP3
MKYQIVLLFLASVCFENAISLNEAQMKAGIKLIKNTCRTKTKITDEQIAKMHEGVWDDADD
VTKCYCHCA
LGMMKMQAKNGAFEYELFEKQKPMIPETIRETLIASVDNCINAGEGLTKKCDLSYAFFKCV
YLYDPEHYM
FP
>AcorOBP4
MACISWRYFILLLFSVELTYCYLTEAQIKATQKLIRRTCKTKAKITNEEELDRLPKGNWDDV
SHTSRCYL
HCCLSMLKVINSDGFIDLEAGMRQSAILPPERRASSEIAIDTCKDKGEGLTDKCDIAYEIAKC
LYDFEPK
FYLIP
>AcorOBP1
MLKLVVLLTLGIYVPTVMCMSEEMEELAKQLHNDCVAQTGVDEAHITTVKDQKGFPDDEK
FKCYLKCLMT
EMAIVGDDGVVDVEAAVGVLPDEYKAKAEPVIRKCGVKPGANPCDNVYQTHKCYYDTDP
QSYMIV
>AcorOBP14
MQKLLVFVVQMCLILSIQALTIEEKATVTKIGKKCIEETKVDVKLVEKGERGEFADDPKLKE
FVFCFLKA
SDIINADGYPKPDEIKVRLANDAPVSEIDDVLSQCESKAATPVDRAADLWKCYWKKSPVHI
PLQ
>AcorOBP13
MKNIVLVSSLLLGLSSAYDFSDEFFNQLLSQEYDDFASGESVFLHPRVRRDDEASKCHHRHK
FCCGDELM
SKLHDKYRDTKRECFKQVTGKEFGGGPPFTCEELEERKKEMTCVAECAGKKKGAVDDKG
NIKEDEVKKLV

AECTAELEWFKPMLDEVTTKCIAEAKAAAEKYDKKGCNPSDIKFSFCIFKEIQLNCPADQIK
DQERCDAM
RASLKKHDHPPVH
>AcorOBP12
MCSSVAKFYILGIWIYSTLILTKALECGLSSSQNQDELRRYTDICMKKNLPINGDQIIENTSM
GQQGYDS
SYEDDSKPSSSEDSMSSKEHAMNSLREDSINDSMNRNGDNNLRNNTEITDDCVIRCVLKQL
GMVDPSGYP
DHSKISQNLIKGAENRELKDFLQDSTDDCFQMMEQDEHMDSCSFSTQLIKCLAEKGKSNCA
DWPMSDVPF
SHLF
>AcorOBP11
MKTSITLFLCALAAVNAYEFEDQAYNQLIAEELHPLHYRGARDTTQKCIDQNSCCSGPPISN
FHASDKEA
SQQCSKEVNFNRGSIRGPLTAEQKDQIKCIAECIGKKKGYLTADGELIKDKLLSSMKERLQS
VAWLAPKL
DSMFEDCLPQNENTAKQPKKCNDVGLTVGHCIWKQIQLQCPLNEQQNPQNCKNLQEYLTT
HNQFPPAPPV
KC
>AcorOBP10
MKTIAVVVLLFASSVLCQDSAEDRQQRIRRYREECVKEAKVDPALIDKADAGEFADTKELK
CFAKCFYVK
AGFITEQGELLMDVVKAKLPPEHEREKALAIIELCKDLKGADACETAYAIHKCYFQNAHAA
NLHKN
>AcorOBP9
MKQIIVLAVLCVIVSVQALTDEQKAKLKVVSDKCIASSGADPSSVEKGRKGEFGDDPKLKE
FIFCLLKAT
EMLDDNADVRLDKIKAKISKDLTEAEIDTLLGKCKPTATVPVEKAAEFWKCYWANTPKRIE
LV
>AcorOBP8
MFCKLFILSLCLLNVVCVTKLQRMIKDAIAHLGTECLSETSDATMSDVQDLVDHVRPTTRK
ALCLITCIH
TKAGMQDEHGKLKEEGGLNFVEPLKQEDMDYYEISKEHFINCINTVPDDAEACIVGGRFN
DCIIIGGKTK
GILD
>AcorOBP6
MRSIFVVTLLFGSFLSIHADAEYEAEKQKLRQKAVGLMTECKDKVGASAEDVQALTNKQL
PTTDKGFCLI
ECIFTNGNVMKNGKLDVQGTLQVLDTALSKNPDAKKKTTAVLQTCEKEVGAGGANGCET
AKLIAECFKKE
AKK
>AcorOBP5
MMKLLLLLVFVSLSMQFQAKRKLTQDEIRAANKKCLKNSGMDSGVVKNIISLDTFPKPSDK
YFKYLECMY
FDQGYLDSDGLISYETIEDFILDFYDVDTVKQALEPCVVLQEGQNGGERAYNAAKCLIQNL
EALEKRYEK
QNKNADNTT

>ItypOBP10
MNAFICMFLVFGVVKAYDFSDSIFNDHLNQIYYTLDNWQHERIRRNAEDVELKCRKPPPPM
PKPCCAQDS
FRDLMDKEREVLRDCFKEVVGEEHHPGRSNHPNKFDMFSCEAVEKRKNDIICIKQCLGSKL
GLVNKDGKL
DQAQIGNYVKSTFKNEAWLSPLADQIIGKCLVEAESVAPPKFHIEKLKPCKPSVITFKHCLDR
EIQLNCP
ADQIHNQESCERFRNHLNHKNDFDEDQPMMGPPDDD
>ItypOBP12
MLTVGKLVLVLVLVLIETSALQKTNNKCEIPTAAPKKIEDVINTCQDEIKIAILSEALEALNIN
EHKVSR
KRRSTFNDDEKKIAGCLLQCVYRKMNAVNQYGFPTVDGLVSLYTEGITQKEYVLATLQSVT
KCLGKAQKT
YDIPAQNGTASTACDVAYGVFDCVSEEVAKYCGQTP
>ItypOBP14
MYGSVLKVSLVFAVISVISCQDFTEEQRKKIIQNRQDCIQETKVNPELIEKADQGEFIDDQAL
KCFTKCF
YLKAGFVNDEGEVQKDVVEAKLPPQADKKKALEIVDKCAVKGKDACETVYLIHKCYFEH
THPDLPAKAEE
EKKA
>ItypOBP15
MIQHHRTNSDLTAVKMKILAVLFVICVLFQFTIARNGGNLHYSKISMKKVQKRCQKNEESRI
DPDVLKKL
RKGEEVVQLPDNFPDHVTCLMKGMEYLNDDNTVNEEKVRNMVQRRVTDDQDVDAIVGE
CKAVKTALKETA
LNLINCLRKHELLWNHNFHD
>ItypOBP2
MNSVAVFAVLALGAVCVIDAYNFQDEDFXSAVVVRDGRIVDSIDSGPVHPRVRRDQEAATV
AEEKCPKRH
RRPKLCCAEETLDALHAKKKEITKACFKEVTGLEKQDRHDHGPHFKRFDLFNCKEVEKRK
SDMICIDQCV
GQKKGLLDDSGAPIRDQLIQHLKQHFSNESWFDQTVVEKITSNCLAAAKNATETPIKFSTEG
LKACNPSG
ITLKHCLFREIQLSCPADQIKDKTACDRFQDRIQKEIEIDDLRLAPDDQQ
>ItypOBP3
MATGKVFVYFFVVLFLSEQSVSRMTEKQLAAAVKLVRNMCLSKEKAKLEEVDKMHEGNW
DIDHKTQCYMW
CVLSQYKLIGKPNHFDRESANIQVDTLLPESMHDYVVGCLDKCENAATNFDDKCVAAYEY
AKCLYFCNPK
EYFLP
>ItypOBP4
MISAVIFFALVGTIFCADADLTQQQKDKLLADGKACVAETGVSTDLIQAARQGKFTEDDKL
KAFSFCMSK
RLGFQNDAGDIQTEVVKQKLGGALGDLGVAAQLVTKCLVPKATPQETAFESFRCYYQNTPT
HLTVF
>ItypOBP5

MRQPGGNNKNTQQDYEMWTPSTGYQPSGSNNDFNVRTRYDGNTRFNRPSSEECRDQGNG
NIPRSPFGSSN
LPRRQRSSYFNREDDDNDNDCISQCVLGYMQLLDTDRSPSETLIIKWLQEHVTRNEMDRIK
ALRDTRKCF
GKLVTTDIEDGCEYAKELSKCLELDLE
>ItypOBP6
MVLYLCDLVQLAPTNQTEEVTTSTKRQLTREKKKKIGKTCMLETGVRIETILRAIKEDIPKN
DEKYKSYL
VCSYKKQGYLSEDGGTMLYDNLYSFLQESAGYAKEDLHYIDDCKTITAETPGDLCLKKLVG
ILDGLHKVE
KNREIDTNTIES
>ItypOBP7
MKVLFAFVCLVVLIQVNSQTDKQKELLAQHYKECLAKSKVNEATLQKARIGQFADDDKLK
EHILCVAQKI
GFQNSAGQFQNQVIETKLREALKGDAAKTKKLISDCAITNPDPKLQAFNAFKCVYQKASIN
LL
>ItypOBP8
MFCKLFILSLCLLNVVCVTKLQRMIKDAIAHLGTECLSETSDATMSDVQDLVDHVRPTTRK
ALCLITCIH
TKAGMQDEHGKLKEEGGLNFVEPLKQEDMDYYEISKEHFINCINTVPDDAEACIVGGRFN
DCIIIGGKTK
GILD
>ItypOBP13
MKNIVLVSSLLLGLSSAYDFSDEFFNQLLSQEYDDFASGESVFLHPRVRRDDEASKCHHRHK
FCCGDELM
SKLHDKYRDTKRECFKQVTGKEFGGGPPFTCEELEERKKEMTCVAECAGKKKGAVDDKG
NIKEDEVKKLV
AECTAELEWFKPMLDEVTTKCIAEAKAAAEKYDKKGCNPSDIKFSFCIFKEIQLNCPADQIK
DQERCDAM
RASLKKHDHPPVH
>ItypOBP11
MKTSITLFLCALAAVNAYEFEDQAYNQLIAEELHPLHYRGARDTTQKCIDQNSCCSGPPISN
FHASDKEA
SQQCSKEVNFNRGSIRGPLTAEQKDQIKCIAECIGKKKGYLTADGELIKDKLLSSMKERLQS
VAWLAPKL
DSMFEDCLPQNENTAKQPKKCNDVGLTVGHCIWKQIQLQCPLNEQQNPQNCKNLQEYLTT
HNQFPPAPPV
KC
>ItypOBP9
MTKLQIVLLLTTLMGSFNIITGEKKCNSSNCMYDRMLETVGKEFIEQCFKETGVTPEDIRSV
MEQNGYGE
KQIVFPKMLDKENWYFGKRWSNQYRLY
>HeleOBP1(a)
MKTIAVVVLLFASSVLCQDSAEDRQERIRKYREECVEETKVDPTLIDKAD
AGDFSDTKELKCFAKCFYMKAGFINEQGELLMDVVKEKIPPEHDREKALA
IIELCKNLKESDTCETAYAIHKCYFQNAHAANLHKN
>HeleOBP3(a)

MCLSLTIFVRPAPYKQLPISNKPHYNADSRTTLKVFISFLQENPTRRRMK
HTKMNFFALLFLSVALIREITSAETSSDALRRKICIQQTNVNPSLVDKAN
EGQFSDKRELQCYFRCYYLESGFINDSGEIQTDIIKSKIPQKLDRKTAQQ
AIDTCKKVKGLDSCETAYELQKCLYDNKVKL
>HeleOBP4(a)
MKYFILLCFLTCVITKIYAKPPPFMDDMHQYRDDCLKDLGLPADTATHPP
PSADGGPPSNNEPSHDMKCLLNCLLRKGGILNSSGVLQPDKVKLSVDASV
VDLNKCTTVTDSDPCQQTYLIERCIMDQLPKQSM
>HeleOBP6(a)
MKCMLVAVFLLASLLQVQGQTYDEEKQKLRQQAMNTLGECKTKVGASDED
IQAIVNKQLPTTKAGLCMLECGFSTVGIMTNGKLDSANTLKILAPALSKN
EDKSKKVTEALNNCEKEVGNGGADGCETAKLIAECFKKESMKS
>HelePBP(a)
MLKLVVLLTLGIYVPAVLCMSEEMEELAKQLHGDCVGQTGVDEALITTVK
DQKGFPDDEKFKCYLKCLMTEMAVVGDDGIIDVDAAVGILPDELKDVAEP
VMRKCGVKMGANPCDNVYQTHKCYYDTDSKNYMI
>HeleOBP12(a)
MKLFVFLSVLVAIASCTMQDFLDKANERMAKVFDECTTSAGATKDDIMEL
MEIRIPSRKEAKCVLACYHKKYGIQDQDGKLDKTAAIEAMKDLKVEDPEL
YDKAVQLFDTCIEQVPNQKCECETAAIFMYCFNIYGKMMGLKPGMVPM
>HeleOBP14(a)
MFRLTMLLFCVLQASAVPKLQTLFQEHAFKQGSECLSEVGATMDELKSIV
KQDIPTTRAGMCLITCIHEKFGMQDNNGKMLRDGTLAFLEQVKDDPAYYE
LVKEHFLYCLDTVSNNDEKCTIGNNFMSCLVLGGRKKGIFD
>HeleOBP17(a)
MYFTIPLSLILVVLVNYGETCSRPDDLVNNEKTCLESSTTFFEKIQHQAT
GLFTSAKRKFGIDPNAQDEPLNCDYYLCILRSIGMVNDYGILELEGTKLW
LNRNVPQEYNNEMVEHATLCFQNIGKNNVNETNCDRSPKYIKCLHNYEKC
QVFKFP
>HeleOBP18(a)
MLYKLVVLLFYFLEVSSVTRIQEGIKGGIATSGAICLTEIEATMDDLRNL
VEHVKPTTRAEMCLITCVHKKVGMQNEQGKLVEAGISNFFAPLMEADSNY
FAVSKQHFLDCAKSVPDDDDECVIGARFNDCVIIGGKEKGLLD
>HeleOBP7(a)
MKSVLVITFLALCSYMVLGETIQEHGKRVLEKIMDTGASCAEKLGATPED
MEKLMKKELPDSKAAQCVISCVNKAFQLQNEDGSINKGVMPPSMADIKGI
DEDIYNKMATVWDICSQKAVGSGDECDAGIHLVKCMKEESEKLGLTKEAM
GF
>HeleOBP5(a)
MYAMHKHALVAFALLLVWLTPTNCYLKQAMAAADGCLGDLGLDASELKDL
VQKMDNLLEPTHNGKCIVACSVQAVGFMKDYKYNENAIMKSLPGTASVKF
DNCQDITGTDDCDRYFKMSVCVTKQIPIND
>HeleOBP16(a)
MKTILLTVTLVLGFASAYDFGDEFFNQLLTREYDDFSSVDSAFLHPRARR
DEEAAKCHHKHKFCCADELMFQLHDKYRDIKRECYKEVTGKEFGGGPPFT
CEELEERKKEMRCVAECAGKKKGMIDDKGNLKEEEAKELVKAITADLGWF
QSVSDEIITKCAGEAKAAAEKHKDGCNPSDLKFAFCIFKEIQLNCPADQI

KDQARCEAMRESIKKYDHPPVH
>HeleOBP8(a)
MIFFTIVYISCIISAAQSLECELSSNQNQEDIKRFTNMCMTKTVTKTDEF
TENASAEQSYENSYEDEAHSSLEEIPDARALSKGNISNSTKSTINNTRDG
ENLNNTEITNDCVVQCVLKQLGMVDPSGYPDHVKISENLMKGIDNRELKD
FLQDSTDDCFQLMEQDEHVDHCYFSTQLIKCLAEKGKSNCGDWPMTDLPF
HHIL
>HeleOBP11(a)
MRSLIILVLVTVTMAEDADTDLLIGSDAQRNQCIKELKFNADEIREIDSL
DDLDDMTMNEKCILRCVLMKLGAIDKQGALVPDELDEQITKDLNIDFSKC
VPQKNITDPCEQTYVLTRCMFQLLFKAAEKS
>HeleOBP10(a)
MKYFILLCFLTCVITKIYAKPPPFMDDMHQYRDDCLKDLGLPADTATHLP
PSADGGPPSNNEPSHDMKCLLNCLLRKGGILNSSGVLQPDKVKLSVDASV
VDLNKCTTVTDSDPCQQSYLIERCILDQLPKQSM
>HeleOBP19(a)
MKHVLLFITLNLFLTQINCDMTAVSAAMHDCLTKLNIDLKEVNAMENAVM
TNTEPSRDGKCLASCTMEKAGLTKDGKIIVDAVNALDPTIASVDLQQCQG
LTGSDNCERDYNILKCGVGQYMLKYKSKPQQ
>HeleOBP13(a)
MRYSILLCFLTCVITKVSTFAGIDQYKDLCSRELNVSVSNWQREPTSPEG
KPTYLAEPDHGGKCLLNCMMLKAGVIDETGAIQSLFIYLPDSHKCANITD
TDVCQRGYLIESCIRKELRLAVRL
>HeleOBP2(a)
MCIYEMKANLLVLVVIFSLARSVTSELTPTANLTLETAHYTCLVGSGIEL
NQINDSLNGNYSENKQVAQYFQCMFRMYGLLDVHGNLKNETIFDAIHSLE
KEHSYTEDGVLNCQNDTFNRNITRNEIAYEFFKCFKNYTETAYYWSILKY
LLNKQGAYPAITLTQSLKLETVHFTCAMGSDVQLQSIKNSLNAIYTDDNK
LTLYFQCVFRMYGYVDKDGSLKNETMLNSIPSEDKDKSYTEDAIKSCQYV
WTPTSDAIAYEFFKCFRNKTDAAAYWTTLKELSSKNDKQDVSNVLVDFLL
>HeleOBP9(a)
MSAMPKHALLAFVFLLVGFTLTNCYPNELIAVENSCLEQLGLDESELNDL
KLKWENQLEPTHNGKCIAACSVQAAGFMKDYKYNEDAIMK
>HeleOBP15(a)
MRSLLILVLATVVIVEDVFSNDAGRIACINKLNLNPDEIRGVDALETLEV
LTKDEKCVLRCMLMELGTIDSQGAIAPAVLDRKMLKDLKIDFSICVPQKN
ITDLCEQTHVLTQCLMKLSVGAVQKS

CSPs sequences

>HoblCSP1
MRLIFYSFLIYLAIADERYTTKYDNIDIDAVISTERLLQNYIKCLLDLVVCTEEGSELKKNMP
DAIQNDC
SKCSDKQKEGSDKLILYLINNKPEYWQLLEEKYDPTGENTKKFIEFKRMMTERTAMYATVS
K
>HoblCSP2

MYKVFIFVIIALVVAVISEKKNDNEEYTSQYDGIDIPKLLANRRLVLGYCKCLLSKGACSPDG
AELKRVL
PEALEADCRKCSKKHKHGARLVLHHLIDHEPKCWKELEGKFDPEGTYAKKYKHNFTLED
>HoblCSP3
MRLLTVLPLIVSSSVVLLVMGADPPGGYYTTRYDHLDIENILNQKRLVHYYAACLLEKGPCT
PQGTEFKN
ILPEAIKTNCLRCTEKQRIVTTRTIKRLTKEYPDIWGQLEQKWDPTGANVKRLLASVNRPRPI
SGIPSLA
DRFGNEDGEITTRSPTSSSTIDGGISSTTSSSVSVPSSTGSSSSSSATTTTTTTTTTTTTTRPPT
TTFR
TIYKPVTARPFNSIGPNLMILNPKVIIDKVLYTADAVLNTVSGVLKG
>HoblCSP4
MIMLLYLLLSVILIVPISGGDEKYTTKYDNMDIEHILSNDRLLSKYVQCLLDLAPCTVDGLE
LKKNMPDA
LETNCSKCSDTQKVSSEKIISYLIDNRPDYWTPLQNKYDPTEEYTKKFIEAKKVKAKVST
>TcasCSP14
MKTFVILFFGVFFIIFSDFVNGKTLHRSTRDDKYTTRYDNVDVDRILHSKRLLLNYINCLLEK
GPCSPEG
RELKKILPDALVTNCSKCSEVQKKQAGKILTFVLLNYRNEWNQLVAKYDPDGIYRKQYEID
DDYDYSELD
SAKK
>TcasCSP13
MIPLIAIAGILAVSAAPAEFYESRYDHLDVESILNNRRMVNYYAACLLSKGPCPPQGVDLKR
VLPEALQT
NCAKCTEKQRTAAYRSIKRLKKEYPKIWEQLRAVWDPDDVFIRKFETSFESGKPSGVISTNT
SPPSPILS
NRFGENEEADAASNVISSTPLPPTTSTTTRTTLTTKFTTKPSTKPTNKPVVVTKPPQAPPFATV
GANLQA
TVSFGTNLVGGIVRSLGTLGSRVVESGTKLANMVISAAIRP
>TcasCSP19
MKFFIAFLMLLGAVWCEQYTTKYDNINVDEILASERLLKNYFNCIMDRGACTPDADELKRV
LPDALKSDC
AKCSEKQKEMTKKVIHFLSHNKQQMWKELTAKYDPDGIYFEKYKDKFDS
>TcasCSP12
MKLISAVILCAFLVAVSAAENKYTNKYDNVDVDKILNNDRVLTNYIKCLMDEGPCTSEGRE
LKKTLPDAL
SSGCTKCNQKQKETAEKVIRHLTQKRARDWERLSKKYDPQGQYKKRYEEHVATSRAA
>TcasCSP11
MYSYLIPLYLFLFVHYGWSEDTTHKYTTKYDNIDLENVVKNERLLKSYVDCLLEKGRCSPD
GLELKKNMP
DAIETDCSKCSEKQKEGSDFIMRYLIDNKPDYWKALEAKYDPDGTYKKRYFESQKDEVSK
VEA
>TcasCSP10
MKLFVINFILMSLVYMSFGASVPYETVDIDKLLADDKMVTEYMACLRGEGPCNPAEKDLE
EHIPLVLGNY
CADCNDKQKNFVIKLATFVIKNRFDEWRQVQKRFDPDLSHADDFNKFILGS
>TcasCSP7

MKTFVLVAFAAVLGLALARPQEKYTTKYDNIDLEEILKSDRLLKNYFNCLMERGTCSPDGE
ELKKALPDA
LHSGCSKCTEKQKEGSRKIIHYLIDNKRDWWNELEAKYDKDGVYRQKYKDVIEKEGIKL
>TcasCSP1
MKTLVPLLFFVIAIASSLAENSKYTTKYDNVDLDEIIKSDRLLKNYVNCLLEKGKCTPDGAE
LKRHLPDA
LHTECSKCSETQKNGSKKIMRHLIDHKRDWWNELEEKYDKEGEYRKKYEAEIKGKKD
>TcasCSP9
MKTLVLVLFVAVLSVVFAADKYTTKYDNIDLNQILKSDRLLKNYVNCLLDRGKCSPDGQEL
KNNLADALQ
TSCSKCSQRQKDGSRTIIRYLIKNKRDWWNELEAKYDPTGIYKNKYADELKAEGIVL
>TcasCSP6
MFLAIVLVVCACTNVLSEEYTNQYNDELDAALKSERLMKSYFECLLGTGKCTPSGEELKK
DIPDALKNEC
AKCNDKHKEGIRKVIHYLVKQKPEWWEQLQKKFDPQGIYKKRYQNYLDKEGLKA
>TcasCSP2
MFATSALFAFICIQGLVSAEEYLVPQNIDLDEILKNDRLTRNYIDCILGKGKCTPEGEELKRDI
PEALQN
ECAKCNEKHKEGVRKVLHHLIKNKPNWWQELEAKFDPKGEYKQKYNKLLEKEGLQA
>TcasCSP4
MFKVLFVVFACVQAYVYAEEYTVPQNIDIDEILKNDRLTKNYLDCILEKGKCTPEGEELKK
DIPDALQNE
CAKCNEKHKEGVRKVIRHLIKNKPSWWQELQEKYDPKGEYKSRYNHFLEEEGLN
>TcasCSP3
MLFTVFLVLTCAHVVFLEEYVIPDNIDIDDILSNERLLKNYVNCLLDKGRCTPEGKKLKSTIP
EALSTDC
AKCNEKVKANVRKVLHHLIDNKPDMWKQLEAKYDPSGEYRSKYKDELEKNGIHV
>TcasCSP17
MHCLLQFCLLAAIFTCVKPQLTRISDEAIESTLNDRRYLLRQLKCATGEAPCDPVGRRLKSL
APLVLRGS
CPQCTPQEMKQIQKVLAFVQKNYPKEWNKILHQYAG
>TcasCSP20
MRFFVIFFVACVSVALARPEDQYTIKYDNVNLKEILQSDRLTENYVNCLLEKKPCTPDGEEL
KRVLPDAL
KTSCAKCTDKQKQGAKTVIQHLYKNKQDWWKQLEAKYDPEHTYVKAHEDELKAL
>TcasCSP16
MTAIVFLLALACLKTYVSSQEYLVPQNIDVDEILKNDRLTRNYLDCVLGKGKCTPEGEELK
KDIPEALQN
GCAKCNEKHKEGVRKVIHHLIENKPNWWQELESKFDPQGEYKKKYDELLKKEGLAN
>TcasCSP15
MIFKIHFLVFGALLTYVSSVEYLILREIDTILKNDQMTRNYLDCVLDKGKCTKEAEKLKKGI
TETMKNGC
VKCEQKQKEDVHKVFQHLMIHRPNWWHELETKFNPHHEIKLQHLHQSKFNPHEEVKLQH
LHQFPHHDFLE
REGFIR
>DponCSP3

MWKLVLLGSLLICIGQTLAEVTEKSQYTTKYDNVDINEVVHNERLLKNYVNCLLDRGPCSP
DGLELKKNM
PDAIETDCSKCSDKQREGLEAMMRFLIDNKPEYWNPLQEKYDPTGSYKKRYLDAKRAEVA
IQPAEKTP
>DponCSP1
MWKLVLLGSLLICIGQTLAEVTEKSQYTTKYDNVDINEVVHNERLLKNYVNCLLDRGPCSP
DGLELKKNM
PDAIETDCSKCSDKQREGSEAMMRFLIDNKPEYWNPLQEKYDPTGSYKKRYLDAKKAEVA
IQPAEKTP
>DponCSP2
MKFCVVLVLVLQIAICLGQTYTSRFDNINIDEILSNKRVLNNYVRCVLDEGPCTAEGRELRT
HIPEALRT
SCAKCTPSQQKFVRKGANFLIKNDPDQWKRIAKKFDPEGKFAPQFRQFLNA
>DponCSP8
MKIFIVVCCAFIGLVLADTPKYTTKYDNVDLEEIIKSDRLMKNYVNCLLEKGKCTPDGAEL
KRVLPDALH
TECSKCSDSQKKGSRKIMRHLIDNKPEWWTELENKYDKEGAYKKQYREELKKDGIKL
>DponCSP11
MAPFPQSWLQFGALLLLLALVQGQILNGNVYVEKQLLCALDRAPCDNLGRQIKDALPEIIG
KNCKACDNK
QLSNAKRIARFVQNKYPNVWNDLVRKYGNPTN
>DponCSP6
MKTIIFLVVVASFYGLSSCKPQEKYTTKYDNIDLDAIIRNDRLLRNYIDCVLGKKKCTKDGE
ELKVHLPD
ALQSDCSKCSEAQRNGSRKIITHLLKNKRGWFNELQAKYDPAGNYLSKYSEELRKEGIVI
>DponCSP4
MLLIISVLIGMALDLTDAKPAAKNYASKYDHIDVGAILNNRRMVNYYSACLLSQGACPPEG
VELKRILPE
ALQTNCARCSEKQATIALMAIKRLKKEYPKIWSELSAKWDPSDSFVKKFETTFESLHGPGR
RVESTTSAG
NKLDPSEADGNTIDANITQTSPEGSDRVNQPDTTTTPQIITTNPSFSTSTKPAFSSTKRPSPIPG
LVPFN
TFFTNPPIPIRPIVNLNLGGNIGATVKAIKQVEKMVADIALEKIGIIRSILRPWRKAKKTRYA
>ItypCSP1
MKLIISFLLIAVAALSYADKYTSKYDDVDIDQILQSERLLRNYLNCLLDKGRCTPDGAELKK
NLPDALEN
ECSKCNESQXKGASKVIRYLIDNKRQYWDELAAKYDPEGVFFKKYEAEAKKDLLDQIGRA
>ItypCSP4
MALLIFFVVILTVGLASAKPAVKHYASKYDHIDVETILNNPRMVKYYSACLLSQGPCPPEGV
EFKRILPE
ALHTNCHRCTEKQATVTLRAIKRLKKEYPKIWSQLSQMWDPDDVYVRKFESTFGNRNKIPS
VVVNNGWDL
GSSTTSNADEPRPDTTTHQIITSPNIMSFTTSKTSSTPITTSSTANPSTKTSTTTVGTTTKPPSRP
APIP
GLLP
>ItypCSP5

MQCLGLFVVLVLGCSLVAAQSPYTSKYDNVDVDKILKNERVLTNYIKCLMEEGPCTPEGRE
LRKTLPDAL
ASGCSKCNEKQKDTTEKVIRHLMDKRTKDWDRLSKKYDPQGVYKQRFEKELSARKLA
>AchiCSP1
MNQYLLVLFVALIGIVATQKYTTKYDNIDLDQIIKSDRLMKNYIDCVLERGNCTPDGLELKK
NIPDALLT
DCSKCSDTQKNGSRRILKHLVQNKRAWFDELAAKYDPDNAYRKRNEQEFAKEGIVF
>AchiCSP2
MVCLLPVLVAAGTLLGLIDGANAQYYASKYDHIDVDAILNNRRIVNYYAACLLSEGPCPPE
GIEFKRILP
EALRTNCHRCTEKQKTVTLRAIRRLKKEYPKVWVLLEKQWDPDSSYISKFESTFGGKPAES
SPTPSVQIV
NRFASTENDNKTVENTISQLFTTPLPTSTSTRTSTSSTTTATTRTTSTATSTTTRTTTRTTTRTTT
PTTK
TTTTKPTTVSTKATPVTKATIDDFGTISTIKLNINTISASAKPKKRPRPNIGQSIQATVSLGTNI
VGDLV
RGLGAIGNRVVETGAEIAGVVLKNIARPL
>AchiCSP3
MITLNELVCLIIFFSGFAVPEEKYSSKFDNIDYEEVLRSDRLLKHYANCLLDKGSCPPEGAEL
KRILPDA
LETDCAKCSEGQKRGAKRVIQFLVINKPDTWEQLMAKYDPNGEFKEKYEREWLNED
>AchiCSP4
MKVVLLLAALCAFAYARPEYTTKYDNIDLDEILRNERLLKSYFQCLEGTKSCTKDGQELKN
ILPDALKTK
CSQCNENQRRGAEKVIRHLIDNKADWWKKLEVIYDPEGSYRKAYQEEAEKRGIQLPK
>AchiCSP5
MLKMIIYFLVAVGLTAYTTSTVTERTKYTTKYDNIDLDEIIHNERLLKNYVDCLLEKGRCTPD
GLELKKN
MPDAIETDCSKCSEKQKEGSEIIIRYLIDNKPEYWSPLQEKYDPTGSYKKRYLDAKKTEVNV
EPIVKS
>AchiCSP6
MKLVVLLLFVALCGFAYARPDDKYTTKYDNIDLDEILKNDRLLRAYVDCLLGTKKCTNDGE
ELKKVLPDA
IDNECAKCSEKQKDGARKVIHYVIKNKRDWWTELEAIYDPEGKYRKKYEEQAKKEGIDL
>AchiCSP7
MRAYVLAVFVVLVGAVASQKYTTKYDNVDLDQIIKSDRLLKNYLDCILEKGNCTPDGQELK
KNIPDALLT
DCSKCSDKQKDGTKKILKHLVKNKREWFDEVAAKYDPEGVFREKNREEYAKEGITF
>AchiCSP8
MYFFSGLVCLICISSVIGQKYSNKYDHIDIDRILSSKRVLNNYIKCILDQGPCTPDGREFRDHV
PEAITT
NCAKCTEAQINIIRKTSVFIMKNRPEDWEKIKNKFDPQEKYKDSFMKFINGHN
>AchiCSP9
MKVSLLFVVVVALVVCARADDKYTTKYDNVDLDEIIKSDRLLKNYVNCLLDKGNCTPDG
AELKKVLPDAL
LTDCSKCSETQKKGSKKIIRHLIDNKPDWYKELEAKYDKEGVYKKKYEEELELKKETKEEE
KEKKEEEKE

KKVEKEVKIEKKE
>AchiCSP10
MKVICQTVLSAMALLVLVSNEERSVSAAAVSRAKREEKYTTRYDNFDVAGVLASKRLVRV
YLNCLLETGP
CTPEGRELKKYIPDAIATECIKCSPLQRKQAGIVLSHILLNYRDDFNKLSQKYDPEGKARKM
YNIDQDGD
DDYQDLEEA
>AchiCSP11
MKVVLFLTVIFVIGCLCQKYTTKFDNVDLDNILKNERLLRNYMNCLLDKGKCSSDAVELKK
VIPEALENE
CEKCSEKHRDGVKKVIKYLVENKRDYWNELLAKYDPEGNYRKKYEELSKKEEVQI
>AchiCSP12
MHFKCQVALLLCIALTAIVTEAFPQADTDRPAVSDEALESTLKDKRYLMRQLKCALGEAPC
DPVGRRLKS
LAPLVLQGSCAQCSPKELNQIRKVLSYMQINFPKEWNKVLKQYSR
>AchiCSP13
MKILCLSVVIVLLSGYANTQKFTDKYDNVNLDQILRNDRLLINYVNCLLDKGRCTADGLEL
KKAVPNALQ
NGCNMCSEKQRNGATKVIRYLIDNKRAWWNELEQKYDPQGNYRRQYEEEGKRYGVHL
>AchiCSP14
MQPFSAVIFCALVGLVLADNKYTSKYDNVDVEKILTNDRVLTNYIKCLMDEGPCTPEGREL
KKTLPDALS
SNCSKCNAKQKDTAEKVMKHLMSKRAKDWERLTKKYDPQGLYKQRYEEHLSKT
>AchiCSP15
MTVRYTVVSLVLSFIIAVEVQGQRGLTGNNYVEKQLLCALDKAPCDNLGRQIKDALPEIIGN
NCKSCDQK
QVANAKRIARFVQTKYPDVWNALITKYGAN
>AchiCSP16
MHLTLIFVFTLVAGALSEEYTSKYDNIDLDAIMKNERLLKNYIDCLLDRGKCSNDANELKK
HIPEALETE
CAKCSERHKGGVRRVIKFLAENRKEWWNELVQKYDPDGTYRKKYQDLSKKEHVSM
>AcorCSP5
MEKCFSFIFVLCLVVVAIQAQNRYTTRYDSIDVDSILSNRRILTNYLKCLMDEGPCTNEGREL
KKTLPDA
LANGCSKCNEKQKSSAEKVIRHLIKNRSNDWKRLTAKYDPSGQYRKKYEAQYNIKA
>AcorCSP4
MRLLTVLPLIVSSSVVLLVMGADPPGGYYTTRYDHLDIENILNQKRLVHYYAACLLEKGPCT
PQGTEFKN
ILPEAIKTNCLRCTEKQRIVTTRTIKRLTKEYPDIWGQLEQKWDPTGANVKRLLASVNRPRPI
SGIPSLA
DRFGNEDQNNLGEITTRSTTSSSTIGGGISSTTSSSVSVPSSTGSSSSSSATTTTTTTTTTTTTT
RPPT
TTFRTIYKPVTARPFNSIGPNLMILNPKVIIDKVLYTADAVLNTVSGVLKG
>AcorCSP2
MQLVQIFFVTCIAATTLAMPQNRPQVSEEAIDRALKDTRYLMRQLKCAVGEAPCDQVGRRL
KSLAPLVLR
GACPQCSPGEVKQIQKVLGYVQKNYPREWNKILQQYAG

>AcorCSP3
MIMLLYLLLSVILIVPISGGDEKYTTKYDNMDIEHILSNDRLLSKYVQCLLDLAPCTVDGLE
LKKNMPDA
LETNCSKCSDTQKVSSEKIISYLIDNRPDYWTPLQKKYDPTEEYTKKFIEARKVKAKVST
>AcorCSP1
MEVTFLFSTLIVGLFYVSEAQDNSQGLFLWKYKVDVNTVISSKRLLINYINCLLDKGPCTTE
ANELKKIL
PNAISTQCKDCSITEKQAVGKIFAHLLQYHRDLWNELLDKYDPDGTFRKQYELDEDEDYDD
EKESN
>TmolCSP12
MKLFAVLLVTCLGLVFARPEEKYTIKYDNIDLQEILQSDRLTENYVNCLLDKKPCTPDGTEL
KRVLPDAL
KTNCAKCSEKQKVGARTVIHHLYKNKSNWWKELEAKYDPENTYYKEHEAELKAL
>TmolCSP11
MKILLLLSLLISLTSCEKYTTKFDNINVDEILSSDRLLKNYFNCVMDKGPCTPDAEELKRVM
PEALQTEC
EKCSEKQKQLSRQVIHYLIDHKSQMWKELSAKYDPDGIYFAKYKDDLGS
>TmolCSP10
MHCLLQICLVGCVVATAACATLEAPRTPIADEAIDATLNDKRYLLRQLKCALGEAPCDPVGR
RLKSLAPL
VLRGSCPQCTPQEKRQIQKVLAYVQKNYPKEWNKILQQYAG
>TmolCSP9
MKTFVLVAFAVVVGLACARPDEKYTTKYDNVDLDEILKSDRLLKNYFNCLMERGTCTPDG
EELRKLLPDA
LHTQCQKCSEKQKEGSKKIIRYLIDNKRDWWNELEAKYDKDGEYRKKYKAEIEKEGLKL
>TmolCSP8
MKLISAVIFCAFAVAALAAENKYTNKYDNVDVDKILNNDRVLTNYIKCLMDEGPCTSEGRE
LKKTLPDAL
NSGCTKCNQKQKETAEKVIRHLSQKRTRDWERLTKKYDPDGQFKKRYEEHVAKAA
>TmolCSP7
MKFQIFIFVIFCVTIDNWVQGKTLHRPIRAVQKYTTKYDNIDVERILHSKRLLMNYINCLLEK
GSCSPEG
RELKTLLPDALVSDCAKCSDLQKKQAGKVLTFVLLNYRKEWDQLVAKYDPDGHYRAKYEI
DEDYDYSQLD
DAKK
>TmolCSP6
MKLLLFVVLTTFWAVSSSAEKYTTKYDNVDLEEILKSDRLLKNYVNCLLDKGKCSPDGQEL
RTHISDALL
TECEKCSEKQRNGSRTILRYMIKNKREWWNELEAKYDPDGTYKKKYEEELKKEGIVL
>TmolCSP5
MILVALLVLASVQVALFEEYIIPDNIDIDDILKNDRLLKNYVNCVLDKGNCTPEGERLKKAIP
ESLQNGC
SKCNDKVKEGTKKVIHHLIEKKPDMWKELEARYDPNGDYKKKYKDLIEKEGLAI
>TmolCSP4
MKTIVVLCWSLVVIATTEAHPESEKYTTKYDNVDLEEIIKSDRLLKNYVNCLLEKGKCTPD
GSELKRVLP
DALHTECSKCSETQKNGSKKIIRHLIDHKREWWNELEAKYDKDGTYRKQYEAELKLKAK

>TmolCSP3
MRWLVAVVALVGLCHQVQGQLGLPGNNYIEKQLLCALDKGPCDALGRQIKGALPEIIGKNC
QTCDTRQVA
NARRIARYVQTKYPDVWNALVQKYSGKN
>TmolCSP2
MIATVFLVVICLQVITCEEYVVPQNIDIDEIIKSDRLMRNYLDCVLGKGRCTPEGEELKKDIP
EALQNEC
AKCNQKHKDGIKKVLRHLINNKPTWWEEVQVKFDPTGEYKKKYTDFLEKEGLSR
>TmolCSP1
MILTSVFIFLVLQIFVSCDEYLVPQNINVDDIIKSDRLMRNYLDCVLGKGKCTPEGEELKKDI
PEALQNE
CAKCNQKHKEGIKKVLHHIIENKPEWWEEVQAKFDPKGEYKQKYSNFLEEEGLIR
>HparCSP16
MRSVQIFLVSVIIAATFASPQNRQQVSEEAIDRALKDNRYLMRQLKCAVGEAPCDPVGKRLK
SLAPLVLR
GACPQCSPAEVKQIQKVLGYVQKNYPREWNKILQQYAG
>HparCSP15
MYKFLITICTLLITAKALAGETYSTEFDHINYKEVLANKRLVASYCNCLLEKGTCTAPAEYL
KKILPEAL
ETDCEKCTDKQRDLIPALVKVILTTEDNDCFRQLEQKFDPTGEYRQKYKALLRK
>HparCSP14
MKIFALLFIFVSVSSVLAEERYTTKYDNIKLNEILKNKRLVRGYGNCLLNKGVCSTEGAELK
EHMHDIIE
TGCSKCNVKQRNGFAIILRHLIDKEPKMWMELEEIYDPDGTYRLKYKEEANKEGIKL
>HparCSP13
MFKVLAVLCIFVVVTLAKPADEYTDKYDGVNIDEILNNKRLLQGYCNCLLDKGPCSPDGAE
LKRVLPEAI
ENNCEKCSEKHKNAARKVLKHVYEKEKDCWDPLEKKYDPKGDYKKRYKDSEFAEGINL
>HparCSP12
MYKVLVVLVVFATINLGQGEEYFKGKYDNLDVHGILANRRLIKPFYDCLMEKGTCTPEGSD
LKSVVKDAL
QTNCAKCSDKQRAYAKEVLIHLYKHEKEWYDDLVAKYDPDNKYRQNYKEEAEKEGIKL
>HparCSP11
MKAVILLGVLALVVIVSARPDDKYTTKYDNVDLDDILKNKRLLKGYVFCLLEKGPCSPDGA
ELKKVLPDA
IETECSKCSEKQNNGSRKVLRHLIDHEPEYWTELEKKYDPKGEYKNKYREEAKKEGINL
>HparCSP10
MNKFILLGFLALVVIVAGRPDEKYSTKYDNVDLDEILKNKRLLKGYVNCLLEKGACSPDGS
ELKKVLPDA
IDTDCIKCSSKQRDGSRTVLRHLVDHEPEYWNDLEKKYDPKGVYKIRYKEQAKKEGINI
>HparCSP9
MFKYIIIVLVLVGLVTSKLDEEKYTTKYDNIDLETILHNDRLLDSYVKCLKDEGPCTVDGKE
LKDTLPDA
LQNGCSKCSEKQKEGSRTVINFLVENKKETWDELKQIYDPEGKYEKEYQDIIKKENIQV
>HparCSP8
MKCCSLIFVLCVGVLVVDAQNKYTTKYDNIDVDRILSNNRILTNYLKCLMEEGPCTSDGKE
LKRTLPDAL

ANGCNKCNDKQRTTAEKVIRHLIKNRNNDWKRLTAKYDPSGDYRKKYEAQYNIKA
>HparCSP7
MEKCLSFIFVLCIVVVAIQAQNRYTTRYDSIDVDRILSNQRILTNYLKCLMDEGPCTNEGREL
KKTLPDA
LANGCGKCNEKQKSSAEKVIRHLIKNRSNDWKRLTAKYDPTGQYRKKYEAQYNVKA
>HparCSP6
MFKSLLIISVLAVVVLSKPADEKYTTKYDTLNLDEILNNDRLRNAYFKCLTNKGACTEDAKE
LKDVLPDA
LQTDCTKCSDTQRKNAEVVIRYYMDKRKEEWEELKKLYDPDGIYEEKYKDRLKAK
>HparCSP5
MNAISLLSIILICQIFYVGVSPKQTQGLFFAKYQVDVDRVLSSKRLLINYANCLLDKGPCTSE
SNELKKI
IPNALATQCAECSLEEKQAVGKIFAHLLQYHRELWEQLLKKFDPEDT
>HparCSP4
MNSFLLVLVLSVAAIALAQDSYTTRFDNVNIDEIINNRRLLKGYTNCLLDKGPCSPDGAELK
LRLPDGIK
TNCEKCSEKQRDGAKKIFKHLINKEPEIWKELEAKFDPDHTYRDRYKDNAAKEGILLPN
>HparCSP2
MNKLLATFVIFAIIVVVAGEEKYTTKYDNINIDEILNNKRLLRGYSNCLLEKAACSPDGAEL
KKVLPDAI
ETNCEKCSERQRDASRTILRHLINKEPAIWNELEEKYDPQATYRRKYKEEAAKQGIHFDV
>HparCSP3
MFKSFLIISVLAVVVLSKAEDGQYTTKYDRLDLDAILNNDRLLQTYIKCLLDLGPCTPDGKV
LRDLLADA
LESGCSKCNETQRDGATKVIRFLTKNKKEDWEALKKKYDPNGKYVDQYRKMSENENLKI
>HparCSP1
MRLFFYILLAHLAIAEDKYTTKYDNVDIDAVISNERLLQNYIKCLLDLVVCSEDGSELKGNM
PDAIQNNC
AKCSDKQKEGSDKLILYLIENKPEYWQLLEEKYDPTGENTKKFIESKTMVTEKTDTYTTES
GAKW
>HeleCSP1(a)
MYKVFVVLSVVVAVALAKPDDHYTTKYDNLDIDTILNNDRLLEGYTKCLL
DEGPCAADAAELKKVLREGFETECLKCNDRQKESAKKVLMFFFEKKQPTF
KKLEAKYDPKGVYRNKYRKEAEKEGVKI
>HeleCSP2(a)
MYKVLVVLSVVVTVALAKPDDHYTTKYDNLDIDAILNNDRLLEGYNKCLL
DEGPCAADAAELKKVLREGFETDCAKCNERQKASAKKVLLFLFEKKQPEF
KKLEAKYDPKGVYRNKYRKEAEKEGVKI
>HeleCSP3(a)
MYKVLVVLSVVVALASAKPDDHYTTKYDNIDIDEILSNDRLLEGYNKCLL
DEGPCAPDALELKKVVSDALETECIKCNERQKEAAKKVLRFLFEKKQPQF
KKLEAKYDPKGVYRNKYREAAAKEGVKI
>HeleCSP4(a)
MYKGLVVLSIVVVVALAKPADHYTTKYDNVNIDRILANDRLLAGHIKCLL
DEGPCAPDAAELKKVLREGLETECLKCNERQKVLAK

GRs sequences

>DvirGR3
MFGPNINIIEDTPTTERKRSVYLEGTLPYYQNPEQNPVTKVAPFHPQGPGPGHQLPIKAFQSK
SGSSSVVFDSLRPMLLFMKISGISPIAQLDMVFQVTLQWMIYSVVVFLIILGYIGYIKWDKLE
MVRSAEGRFEEAVIDYLFSVYLVPIFLTPICWSEAKKLARVLTNFVVFENMYHKITKKKFQH
FLGNKPVVVTIGLPILACSTMVVTHVTMVHFKLLQVIPYCYINIATYMIGGLWYMLCDLVG
SMALTVADDFQEVLKNIGPANKVAEFRSLWMLLSRTVRDIGNAFSFTLTFLCLYLFLIITLTIY
GLMSQIQEGLGVKDIGLAITAFFAAMMLLLISDEAHYASNCVKVQFQKKLLLVELNWMND
DAQQEINMFLRATEMNPTDMSLGGFFDVNRNLFKSLIATMVTYLVVLLQFQISIPEDGGAVN
ATMNRAPPQQPH
>DvirGR2
MEIKDLADLYGDELKLTKLIRKSSARAQEIAKRQELDSSDGQIIDDHDQFYKDHKLLLLLFRI
LGVMPIERGKIGRITFSWKSIPMIYAYVFYAVMTVIVVFVGIERVDILLNKSKKFDEYIYSIIFII
FLVPHFWIPFVGWGVAREVCDYKNAWGTFQLHYYKVTGQNLQFPHLSNLIVIISLGCLVLA
VAFLLTLSALLDGYTLYHTSAYYHIIIMLNMNSALWYINLRGIGNASQGLADNFQKDIDNFC
TGYIIAHYRRLWLELSELLQSIGNAYARTYSTYSLFMITNITVATYGFISEIMEHGITFSFKEMG
LIVASAYCMVLLYIYCDCSHKASDNIALRIQRSLIEIDLTTINLDTGKEIDMFLTAIRLNPPTVS
LQGYSDVDRKLITSSVSTIAIYLIVLLQFKVDLIKLST
>DvirGR1
MEYNDQQPNVYRRNPHQQNQQYLNAGFNIRDNDSIEPSETPLPTNKNKPDPELLHAYDNF
YHTTKSLLVLFQIMGIMPIEREMGKTTYRWTSATNVWAYFVFGVETIFVTRVFQERLNLVLL
PGKRFDEYIYAIIFLSILIPHFLLPIGAWTNGLEVAKFKNMWTKFQYKYYCVTGTPIIFKDLTV
ITWSLCIVSWVIGILMMLAQFYLQPDMLLWHSIAYYHILAMLNCLCSLWYINCRAKGRVAR
WLSENLHNALNSSDPATRLAEYRDLWVDLSHMMQQLGRAYSGMYAMYCILVLLTTVVAA
YGCLTEILDHGLSLKEAGLFLISFYCMSLLFIICNAAHQTSHKMGPEFRERLLNVNLAAVDR
RTRQEVNMFLTAIDKNPPIMNLNGYANINRRLISSCTTSMATYLVMLMQFRLSLMRNAAIAA
RKAAILNATLATENASH
>HparGR1
MVAVFKLMHDKLVLTACDLFSMDFSFLFSMFASVTTYLLILLQFDIDAAKSRMAALKANLT
STQYEEVE
>HparGR2
MTQTDIAALLNCTVKLKFHFYVVALRTRVEVINAYLNEKIADLTGPAKSTAFYKMEKDMEI
MLKIHKKVTDASRLVNGIYGFQELFSFVLYFVLLLSDGYIVLYSLTIGGDIDFVSVMAISLKS
VVFHLIELLIDLRACMLLCAKVNHTKNVLFKIKIEPENEEARNIVMVAVFKLMHDKLVLTAC
DLFSMDFSFLFSMFASVTTYLLILLQFDIDAAKSRMAALKANLTSTQYEEVE
>HparGR3
MALKFAMQKLTGEVIGPTLNRNEINGFATQFLHEKIEISAAGFFNIDVTVLVTVLSAVVTYTS
VLVQTNLNLSIFDRFFSNK
>HparGR4
EINGFATQFLHEKIEISAAGFFNIDVTVLVTVLSAVVTYTSVLVQTNLNLS
>HparGR5
MDEEISYYTDELILKGFINEFKHSARAREISKRSQLSSADGNILDQHDQFYRDHKLLLKLFQ
VLGVMPIQRGQIGRITFGWLSIPSIYAYCFYAITTVLVLWVGYERLIILTQRSKKFDEYIYSVIF
VVFLIPHFWIPYVGWGVAKEVCKYKNSWAHFQLDYYKITGKSLRFPHLSTLIVIISSGCLIVA
VAFLFILSALLEGFTLYHTTAYVHIITMINMNCALWYINCRAMGNASESVADSFEEDVRKFC
GSYIVKHYRILWLNLSEMLQKLGNAYARTYSTYSLFMITNITIAIYGFTSEIVDHGFRFTFKE

MGLLVDAGYCMVLLYVFCNCSHNASRNIADRVQTTLLNIELTTVDSHAAKEIELFLIAIEMN
PPKVSLQGYTVVSRELVTSSISTMAIYLIVLLQFKISLVKESKMA
>HparGR6
MVYEAIQGKIFSSYIAFAYIMKTLITLGFTRFQIGTWIHVVKEAQKTVTYIHDIWNNLASKDQ
IDCHAKHLQLIALQLYNSKLSFTAYGFFALDWTLLQRIIS
>HparGR7
MKMMSSRILLMTGTIFTLITLYVFSIISALTEFELTPVGKLLIFFLSWLVMASATYLGFFILTTE
MFYKELATMKTNLFRVFTEITDEDLRKEINAFSLQMLHEDYKITAAGFFIIDSKLFVTISAAIC
MYTTVLI
>HparGR8
MVLQLLTMYRPPYLVVLLVLLVHNFQTESAPTVTQIVQQNISSKPGYIPVYIRNGDTPLEEIN
PDLAEAFDSYAYKHYKIDYTKLKKRRLNFEEKKKEEKNVILNT
>HparGR9
LIDARRLHAQLCEVGTLFNKSYSLQLLLNIGNIFIGFTTLAYYCFDGCMKLQLNEESSNLYN
TVTTGIWTAVKLSQLLILSLTCSVVKKEAKLAGEIIYKIENRYDPELSTEVYSFGQQIIHWNFK
FTAFSCFDIDMTLFYSAVSSATTYLMILLQLDIANKQIEKSMENV
>HparGR10
ESKQTSTICYKIIQEMSPKSETITTNGESQIRENLSFLAQEATLRPSKISAAGFFEVDYTMMFL
LFTSVTTYIVVLIQFNMS
>HparGR11
MNDLNAIADMDTYRIELPNLKYLGQSNNGIYVKSGDDIKLITGNSLNPDASLVLSSTDTDQ
NDCESDRFSFTSSIVQSLDGNLSDSEPVLPDDGHKKVKFVESDL
>HparGR12
MIKKMNNSEDIYNTILPIYVISKILGLAPFKMTKKVFVIQCGPIVPLRIFIVTGIMCYIAVELEK
KEYATGIAALALKCELYLGIFMTFAVLILAVINQKDLMGVITKLVDIDENMRRIHIKINHKNS
RRFVLIQMIFISCVFLIKLMLQYFSHTSTMIVIYSAFNFVDYINTIMLFQYVNLLLLIRQRFIW
VNRKLKSISNFSYPICVGENKVTLTPIQIVKHKSFLFDARLQLEVLAKIYSKLCDISRLTNWT
YNIQILITVTSRFVMITTQLINTYNAIRDPRRGSYIEYSVMSTYLALHLSKIFMVASVSENTAY
TARHTAIYLHNAWEIVPDLRNEIQYFSLQILHQNLSFTGCGFFALDYTLIYNIVGAIATYFIIVI
QLEKAF
>HparGR13
MAVDSRTRQEVHMFLTAIDKNPPIMNLNQYADINRRLISSTLTSMATYLVMLMQFRSTLMR
NAALAAKRSAMNLNRTATDATA
>HparGR14
LSILVPHFLLPIAAWTNGHEVAKFKNMWTRFQLKYYQVTGTAIVFHNLSLITYSLCIFSWVL
GIAIMLAQYYLQPDMLLWHTFGYYHILAMLNCLCSLWFINCTAKGRVAKDLAQNLHNALE
STDPASRLAEYRDLWVDLSHMMQQLGKAYSGMYGMYCILILMTTIVASYGCLTEIMDHGL
SFKEAGLFLISFYC
>HparGR15
MQIMSLLYMGIHLHIIRLIFANIHHQMLSINCELQRVFILAKFKLNKFGMRISKCDILVENDLY
LEIYANTMNFNRFFIIPLGLTLSTYCTMNVTCSYLFVSTIRRALLTSKCNDIAMIVWLGILILG
CYGNICNIIYIFSNVKNEAQKTVTYIHDIWNTLASRNEIDKETRHLQLISLQLYNSKHSFTAY
GFFALDWTLLQRIIAAVTTYVVILIQFEMSSNTKNSFTNATLYQ
>AplaGR3
MDLIVNPIIKKISCLTLFAEAQKNKKHSFVFIYPKNKATRIFPSSYFSKFKNGIIYNDISVVVRI
LQIGAIFPVKATKNGEFRFSKFIFAYSVLIFAMLSTITAYLVFSNVKIKKTEGRFEDVVIEYLFQ
FYLTPIITFPLHWVESAKMAEIFSKWYLFEDLYRKIIGNKILIRKKRKYTIVTIGLWLMSSGM

MFALYITLAEFEALKVIPYCFFNVTIYIFGGFWCIYMDAIRCVATQLEKDFRRSLRNIGPSYIP
AELKILYMSLNELLQDFSSALSYSLIFLFLFLFVTVILSIYGLVSQLSQGITWKDVPHAVTAIM
ALAVLYISCEAAEKTTTSLKRNFQKPLLLLDTTWMRHETKKEITSFLRATLMNNSDVNISGF
FTVNRGMFGALMSTMATYLIVLLQFQIILPEEMEEENIVNNSTSYNDYLPTEVTDYGYTFNI
>AplaGR4
MIQNTKVKATNLNQGDNFVKSIKFLILIGNFFCAIPLKGLYGGNEHNQIKFEWIHWRTLLNIF
ITVAQIFVVICYICIMVYNGFRITYLATAVFFANCFLSFIFFLLLSKDWILILKEWDKLEKRVNR
YTCKYKTNMQRKILLLTITFLILALIDNLLNVISQVIRTLMHKQDSEFFEYFFVVVGYPGIFKI
TPYALWKGIAIEILNFIGVFSWTYVDVFIMGVSIALSSKFKEMTFRIEQYVRKDVYDIYLWKI
VREDYCQMCLLTKLMDSKLSYLILLSSANNLYFILSQCFQSLRNANDSGLKLYFIYSFSFVIIR
TVCVYLIAASVNIESLKPLKALYTVQSHMYNLEVKRLIRFIALDSVCITGKNFFNIKRHILLD
LASALVTYELVIVQFTKEEYF
>AplaGR5
MRECIIGDTFFDSMKLLVLVGYYICVIPLKEIFQDNTSLIKFRWLYWRTFVNVFITIMDFVLAI
VFIYAMIKWGFNISSFASAGFFLNCFASYLLFLILSKDWFVVLEEWKRLEQRINKVTCRYEV
KLKRKILIISAVFLILTVVDSASNTAVQFSIAFTNKGDVEVFEYFFTTMRLNLFFQMTSYSLW
KAAVVETFNFIGSFCWAYVDVFIMVNGIALSSKFKQISFKIHQYVQMEVSDLYLWKILREDY
FDLCILTKTVDKSLSNLVLLTSINNFYYILSQRFYNVEDMQHYGLKVCFIFSFTLSVTRTICVY
LFSASVNLESTKPLKALCRIEPDIYNIEVERLIRLIGSDTVYITGKNFFNIRRGILLDIANALVT
CEIVLVQYFGKSS
>AplaGR6
MVAKNQENFYQSLKGLLLLGGFFGVVPLEGLLYDKVFDLRFRWIYWRTFLNLFFMAGGLFI
TAAFLVQMVKVEFNIYELGGFVFYLNAFLAYFIFLSLSRQIPALFKEWYLMEKRVFFSKKYQ
SNLKLKVRTVTATFMTLALIDNMASNVKDIYITIKYKDEDVEFAEYFFATVSYKSVFSLISYT
LWKAVIIEICSFACTFIWNYTDVFIMASSIAIASKFRQMNKRIEEYLKMEVKDLYLWRILRED
YADVSNLTKLLNDKMSYLILLSFSCNVFVILKQCFNSLRDFNQQVEKIYFVYSFAFLIIRTSSV
CIYAAAINQEFRRPLKALFSVNSDTYNTEIKRLIRQIGSDDLCLTGRNFFKIKRSLILSIAGAVV
TYELVIIQFAGEYLKLNR
>AplaGR7
MRVPHIKSKFRKAQNAHQSLRSCIILAQVFGLFPIQGALEDDPRKLKFRWKSFIVAYSILMLC
GLFCLLLINFIIVVTTTVCLIKIINILFYAFAFLEAITFFHLAMTWPRLMKHWIRVDEKMKTYD
YPKKLDLKLKLTMIILLLIASVEYSLALIHNAIDYNGITITFQNWEFFIKKQFPLVFSVTNYSL
WKSIILLIVSTTATFLWSYADLFVMLCSFVLAARFKQITERLDKIDFQVVKNELFWRSIREDY
VRLAQLCRHLDDTISNVLLLSYSSNLSFIIYQLFNSIKKLLPLEKTYFYFSFSFLIARTIALSLY
GASINDESKEPIRILNSVPSSAYNGEIKRFLLHLKMDSVALTGHKLFKLTRGFILSIAATIVTYE
LVLIQFNQATLEEYRNNETKDICYS
>AplaGR10
MNLFRFLSVKMSRDVNVPITDEIYTTCEPMVMVSRILGLFPYAVYRDGLNIMFKKSLVLYV
YSYAVALTLVSMIIKGLISDYEAGDKSVRMKKPLSVYVTFADVTILAFTVFLGMVGGPTNGK
IFKIFVRNLVEVDKIIFLNDRQRMRKVSMVVLGAVLFCTSTLLALDFSTWIRFAMDSIDAEY
RVTNYIPFYLAYVIVVVLQCQFWFVIKALRRRLHVLTKLLSRDRIQKGLIFNVTNNKITNVSL
GINPGECTGPNCLGEIIDNFGKKTTSKNVIPNYNRHLFDLRQSDNIETLMKVYFKLSTAVDC
VNKFCGVFLFVCLLSCLIHLVVTPYFLVIEIYGRHDPMYISLLCFWIFGHCCRIIGIVEPCYRF
KEEEKRMLLAISKLLYFESGGGKFKEQVKNFFYLVKTTDNQFTALGLAKIDRSLITTIVGAVT
TYLVIVIQFQKSAVSTNN
>AplaGR11

MQRGDTLAPLANLIRIGRYAGLTFFDIKLNRVILSSANFVVFLPVFIITYIIYAILTVAHVKVVM
NRADEQGLAYTTTLLQIFWHIFRFVSIIQALINRKNMKNILEEIVNIDKILTTRTKSQTKITYLI
FGVYIFSIILWCYFKFTMTHTLSDIYWFPVAQKVTEISTGVFLAINFEINRQLKQQLETINSKL
EKDFEYCSFINDELRREHLNEEEIEDIVEMVMKRAKRNLHLRWSVIHNSCKVMKAFEAGIV
GNIVSSTCFAIIAFDYLAYMTIIKGFPISQLFECFLCSFEAAKDVMVVVLTTYSWIIIDKEAAK
TGLHIHKLWNQTMDLRKFTHTANFLKLASVNILFTKPNFNIRGVLYMEWGIISTVFGTIFTY
GSVLLQLQLDLDARKRWRTS
>AplaGR12
MQEDIPKRIEKLIPTCRYSGFVFFNIVQNKIVSSRSDYVVFLPVFIIIYLLYLTLTASNIIHQLSE
NVQRSFILSDIILHGVSPLSRLLLIIRAVVHREEVIKILKKIINTDRVLLTKKEVTNYATKVILHV
FTLNALIWFIHCYTLDSVAHLKYWGPFVGRIMEISNGVFLAFSFEINKQLKLQLETINSKLEE
CVTHCSIINKQYSRGSLSEEDAIEIIKMTATKIKQYLHFRWSVIENSCRLWQAFEIGISVYIFYT
TCSLTIVIDYMPYFLSIKMPSSVTASNFAVLFSETTKNLTVIIVIVYSWMMIHKEARKTGSLIH
NIWNDVPENILSAKCVQVLKLASINIWHTSPTYTIGGVFPLNWDMFSKVIGTAVTYAAMLF
QLQMNELKQYSLLQSQDIITTLTN
>AplaGR13
MQRDIYKRIESLSAFGRYMGLVSFQIKNSRVFLSKRDWLMPLLIFVIHLVIFVPSAILHLKFQL
KSETRFIFSDFLYVGAYELSWLLLTFQGITNRRRMKDILQKIVDTDQAMEIRIGVRSKTSFIVL
SIFFVNLLIWTIMDISKTSDKYWLPFLLKISEIANGISVILNFEINAHVKIQLETLNLKLENCTH
QCLKKHRNLQLNERNRESLYKIEEIYVNYVKKKLHLHWLIIQNSRGLVALLQIGIIGNLINVT
CSATIMFDYLVQMHTSGETIFSIACFIRNIAAIVEIIKDIIITITIIYSWVMVSSEASKMGPLIHDI
WNKITEIGPSNKSMEFFKLASIHIIHTKPNFKLFGFSPITWNIFPSFVNLVITYGCILLQLQLDV
A
>AplaGR14
MQEYFFKRIGKLLTIGRLLGLTFFHVVQNKVVSSKSDYVLFLPGFVIIYSVYLTLTVPYMKY
NFQVNDQQDFMLSETILHVFCQISRLLLMIQSFIHRKEIIEILEKIVKTDKVLITKKNNSKYTTI
MVLIVFVLNASVWLVHCYTIDFLLPVIYWCTFVGKIMEMSNGIFMVLNFEINKQLKLQLETI
NYELEGCIKNSFIITENRDIEDSNEENLNELVQMNLTRIKQQIHLRWTVIQNSCRLWQAFEIGI
SGNIFFTTCSLTIVTDYFLYLCIFEMSLLILVSHFLVAISEALKDVVVIIVTVYTWKMVEQEAN
KTGSLIHRFSTEAADNVSCKDSVEFLKLVSINNIHTKPTFKIRNILLLDWGILSTIIGAVITYATF
LLQFQITELERYYLLHGTEYTVGY
>AplaGR17
MSNNIFDSISNLVKFSRYMGVVFFTVKNNEVVFNWSDYITFVPSFIIILTLYYFAESPYFSNIW
KFNTQGFFLCESIYYLSCFISRHTLMLEGMMNRKIMKRILEKIYETDIKLNAQLSTNWVKKL
VILFVIVEPIVWTALKVCIMYLENYFYGSIVLNKVPPLSLWLIAIMNFEVNRHFHMELITVNL
LIKKEIGLLKDSIEDFTEGNITEEELDQMVDVKVNKIKKQLHSRWSILENCARAMQAFEMG
TLANLMFHTSVVSINIYYDIFYIIFQPFSKSVAVKLTVSAFVTLRSLCGIFMTIHTWNMIEKEA
SKMGPFVHELYNEVIEAGSSTKSLEYLKLASIHLIHTRPKFSIKQVLRLNWNVIYGSIDLSITY
AATLLQVHLNSLQK
>AplaGR19
MRESIFKSIKNLVNVGRYAGVTFFKINKNKVILSWQDYVIYLPLFVVFKYLSFLSAKQLFLYL
LNNNRRHFIFSEIVFHILCQLARYLFIYQGIVQRKAMKKIIENTNVTDVRLNNNDLRNTTFTK
YILLVPFLINSIYWVIVTPFLNYQHTGRTYISVINKTEEMINCLLMELNLVINHRLKYQLRTIN
YRLKKDIEYVTFIKKQFKEGILQIEDLNDKIDIKINDIKQQLHLRWCVNKTSMDTKKAFEIGII
ATLAVATCSLTVVIDYGLYLSLFNTFSWRIFFKLIGLLTELTKDMAVLMVIISSWTMIEKEATE
TGPLIHEFWNSVIDLESSTKSAEFLKLASLHIIYTTPSFSILNILQLNWDAMSAIVNIAVTYSTS
FLQLQLNAMRNRKNLL

>AplaGR21
MYKNIFDSVQEIVVFGSYFGVSIFRVEDKKIVLSRRDLVRFLVPFIIVNCLIFILTFRNFQSTWI
YSLKTTEIIRNVILYLVSQSPKYLIILQGLFNRHNFKTIFNTIEKTDEQLKHKPAISTHRCIFTYL
GISIFVTNLVIFLCFYSSTDFAHCKREYLFLEIINKLHVAINWLVTELNYKINWHIKTQLKTINF
LCERSISYIKNANQAFRNDNLLDEEHEFIINIELNKIKKLLHLHSSTIKNSLFVLDTFKMSIIAN
VIFASSVGSINGSLLLLHFSDNAFRKPYLVIFLYITEIIMVFSLIVVNILNWTMIKNEVAVTGLL
VHHMWHEVVNIKSDIKSIHFLKLASVQLIHTTPEFNILTLPLNWNGLGKIYTMLVTYAFTLF
QFQYNQN
>AplaGR29
MFNYLQKKLQLWEDEIWIVPQPFKFICQAFGLVSVHPDNRFFKYYKLIQGIYLAFIVSITIGLS
PHLCIYGGYSGIIFPMIYSSVAVTNVFSVVHAVFVRKNFYRELASKVQYIDGILKINRNIFSRM
RLRLVIFTLVGFSIHIYNMSFVELLSKCGLPVYVIIWHKFAVLLDVLLFYNVLSIIKQRFVSFN
QKLVRNRQGNLFYTTKHNDEKVTITNLIVCHQKMYDTVEFVNSYFTFVLLGTLICSTLPLIS
ATSIIVSPLQSSHFISIPFTVVLYSGYICFLCDIVTNFMKEADKTATLCYDIILSQDISYQSTEWS
KTRNSLLLTLAKQVEVCNIRITAGGLFVIHRNLLVQIFAALYSYVLVFVQIKWSINKSTRSFNS
TSLYNNNSRFG
>AplaGR30
MNFRPFKYWDKLFELVLPGLTKIDKNQPLFYKVVVIIMPVCLLCGCLHAVISNRSGYSVME
WLLITRFGIVVSMTFSIFVYETFFGKNAASKLISTEEKITEILKIDTKKLENEIERKLLIIRIICFT
GTLITSAVALFFRYHFGTIIRTILCFFAFQRTFLILSCFKNVELEAKLLKEWLMTETSRPEHNIF
EVMDSCSQNYCLLIKKINYLSDYFGLYFFGMFTIVVQSILILCLHVSKIFFTNNIVYRNIATEL
PSFFEFLGVFLICDMVETAKKELVTIERCCYEILSNQSQLSIYKYPEKRKCIRVFISQVRNHSP
TITAMGFFEIELYTLIGCFKLIFFFLTTFIQTSEYVEL
>AplaGR31
MTVPVIRDIRYSHFLGRIILINSFIVKPNGKIEKPKCIFYVFSSIFPLITITAVVNYVMRSGGVSV
ISEDFTRSFITILNFIVQVAVCVHAYAVNMFGNGFKDASSFLKIIIFIDKQLNNNYSDQKNNQV
QRKIMTRVIVMVVIMTLLAVSDFVMYSTLFFMENGIVFFVASIISILIGIQAIYSHIEGNLLIICD
RLLRLKKMVEIACHNDCRNYETFEDFLKVPASKRQRKINKIYFALFRATEHINRAYGKQILM
LYIHLTMRAVMFLILLYLDLRNCIRTAEMTSLAVLYQLSIRGGWSMGILLSSCAAKIMATSDSI
THLVYETMYKEYGNVDVERVAFTEELHLYLQMSSSRKLEITADDFFTIHRYMPLSLINIVFTY
CIVFLQLNG
>DponGR1
MDGQFLSPYPPEGPDVSRRKSDNIRIVTPETVRPEHVPDNELLEKLHTYDNFYQTTKSLLILF
QIMGVMPIERLRGKTIFRWFSANTCWAYFVYTVETIFVSIVFKERLVLILKPGKRFDEYIYGV
IFLSILIPHFLLPLGAWRNGQEVARFKNMWTKFQLKYFKMTGTVIKFHRLTLTTYSLCVLSW
LVGILIMLAQYYLQPDMLLWHTFGYYHILAMLNCLCTLWYINCTAKGRVAGWIAEKLQEA
LQTKGSAKKLADYRELWVDLSHMMQQLGTAYSGMYALYCLLVLLTTIVATYGCLTEILDHG
LSFKEAGLFLIAFYCVTLLCIICNEAHAASRKMGPEFRERLLNVNLSAVDQRTVEEVHMFLT
AIDKNPPIMNLNGYANINRKLISSTATSAATYLVMLMQFRLSLMRNAAVAARKTAGQNSTM
HG
>DponGR2
MNGKHSFRQLDIKDLEDLYGEQLDIKSMANMRGSPRARDIAKRFKLDENDGRNIDDHDQF
YRDHKLLLILFKWMGVMPVERGEIGKITFSWTSKPMLYAYGFYVITTIVVLLVGYERVDILL
NKSRKFDEYIYAIIFIVFLVPHFVVPFVGWSVAHQVCDYKNSWGRFQLNYYKITGRDLEFPY
LSTLIGTISLGCLFLAVSFLLSLSTLLDGFALYHTIGYLHIITMINMNCALWYINCRAIGNAST
GVAESFKKDIHSYCVAYIIKHYRVLWLELSEILQKLGNAYTRTYSSYCLLMMTNIIISVYGFTS

EVVDHGVKFTFKEMGLLVDAIYCFTLLYIFCDCSHQASANIAERVQWALMEINLNQVDHAT
IKEIQMFLKAIHLNPPKVSLRGYTVVSRELVTAMISTIAIYLIVLLQFKISLVNMRG
>DponGR3
MYNNPGTPLPHGMMDNSYQIPNQNIYFDDGMQFYRTKNQNTITKVAPVPPPKYSNAENME
MMESIDDKSVKKSVIFDNVKPIFVTMRLLGILPVVRRGSIFIISTKWIIYSFLLLLAVAGFIGYL
KYYNINVTRTAEGRFEEAVIDYLFTVYLVPIFVNILALYEAKKQANVLTQMVSFERIYTRMF
KKRIGFDLGSKPLVMTVVLLILGCGVMVITHFSMANFIIYQVVPYCYINVITFIIGGAWYIYC
DIIGNIATCLAEEFQFALRNIEYSNRVCDYRSLWMILSKIIRNVGNSFGYTLTFLCLYLFFVITL
TVYGLLSQIQEGMGVKDIGLTITGVSATLMLYFICDEAHYASTCVRTYFQKKILLVELSFMNE
DAQQEISMFLRATEMNPTDMCLCGFFDVNRNLFKSLLATMVTYLVVLLQFQISIPSSGDDVT
NNTTNTTPSAPK
>DponGR4
MRVLRKINSDTTLTIEENNVNNIQMYNRPHIFYENIRFALILSQCFGIMPLHNISKNAQEVKFS
FMSFRFLYALAHFGCVFATGIASIVKLALNGFMIDETSIVSFYIFNSFASIHFMYLATRWQNIL
KEYSYVERAMKNYASNRNVKRINICTATFFMFFGLTEHILFILTNLNHSFHCEHYNKYPVEIY
FGCAFPQWFTLITYSHWAGALVELTNFISTFTWNFTDLFIIMISISLREKFNQISNRIKQSKNPP
HKFWKEIREDYYRVSNLTKVVDIQIAGLVLISFLNNIFFLCIQLYNSIKEREAVIDSIYFFYSFG
YIVFRVVAVSLYSATLNEAARKPLKYLYSLPTENYTIDVSRLITQINYLPNGITGHGFFLITKN
FLLQAAATVVTFELMIFQFSPVKTTSNQTSLSSGIICIK
>DponGR6
MHININDEMVSTNPEMTISKCLKKLLLLGQIFGFFPVQDINKPTQKFACKSFRMLYSVFSMC
GSLFLCIMQLNKAVKQSIGIDQIDYIAKYVSSAYVAIVFIGLARQWPHLMAEWTRVEQKMH
KYGFPKKLNKQVTIIISFFMTAFLVEYVLHQASRIDRVMECQSDKDRAIQFFFGNLTLSHVFQ
YIPYNIPTSLIFQYWVYQMEFGFTYTDIFVTVISMCLAERMRQVTKRVKLACAKEIADEQIW
KEIRKDYTRLQSLIELVNDKISNIILISFLPNIFTILTQVYSTLKPKHNNTEAVYFYFSLIFLVSRT
VTVCICGAKVNEESRRPLNILNSVPHTIYNDEIEIFISQISNFEIAMNGKHFFNIKKNVILELAG
AIVTYELVLIQFNQESLNSWSRSTRCE
>DponGR7
MYRFNKPKKHFRYKCHSNSFQKCMAWPLIILEVFWIFPLSGIMEKSHQFLKVETVSWKMV
YAILCAILQCIYLPIFSSIIIQDISDRSMQIRSFYVEIVAILQTFNFMHLTHSWKQIVYEWEVLEK
SMMHYKGPYVNLEKTMKIIMGVLLLFLIVLNVFTFLGECVVLTKLYELYTPYGQQSFPATIT
STVYYIGLAQREFIWVFKDILIIIVSICFTFRLRQLKERVFFIIQMRINNVEVWRATREDYAKLL
SLCETINNKFCWLIVISYFQNIQYLLLGIFSAVVHHDNCPTSKQFTKEIQLYGISSMIIRLILVSF
IGGSINSENENLIDLLTSVPSNMYNKEVERFLNHVASREMILTGGNYFKITRSLILQIANALVT
YELVSIQFVQNFKHIE
>DponGR8
MGALKNALRPSIFNQSSDRTSFQEVIKLPLILLQFFGYFPVTGITARSYKGLNFNWRSIRTFLS
VFLLIGYLFQLGCFCAHMRINKLLTLNPLMFHLESVLLSALFIYLATQWRTFSKQWFFVELS
MKRYGLESNMKRKISIVMLLILGLTFVEHALVTSQSILLSMQKHPNSTYEALQEYFVHVEFV
EIFYYLPYSAGVGVFFKILIVQKTFMWSFVDIFIIVLSIYLNFRLQQISHKLMHMSRSKVINEH
SWSAIREDYVKLSRCCELMNSQLCWLIILTFFFNLYHILAQLFSSLRPLDNSFHKTYVWLSFL
LLIIRVTSVCFFGGSIYDEHEHIMFILSTVPTASYNIEVERFLIHLNSAEMALTGKNFFKITRGLI
LKVTSAIVTYELVLIQFNQQEFKTVK
>DponGR9
MFLLKHKRIKPAKVFLPPALKPSSKPPKSILCSFRLFMRISHIVGMLPQENFSKSPEDLKFKW
LSWRAIYSLLIISALLLAVTCCFMHWVLHGSSISGVATAVFYGGSLITVLLFFYLALSWSKLL
KSWHAIDRTMAATYGYPEKLDKQIRVLTAVFLTLAIVDYLLSVANKYEHLNYASGYQLTTFA

YFNDTFPQLFAYIPFSIGTGIFCTAITVHSNLTWALNDLFIIIASSALAMRFRQISQKLIKERDDF
KHAQLSFWRQIREDYDRLTRFCKELDCYISVIVLLSYFMNIFSLLIQLYRSLEFISLPIRKVYLI
FSFGYLIFKTTMVSLYAAWINDESKAFTHILNSVDSTLYNTEIRRLLTQISFDKTALTGCKMFA
VKRGTILSVASAIVTYELVLIQFSQANLYDTY
>DponGR11
MEDKQPSGKSFRFVAPLMKFCTIFGMCPPVFPRNSDQPLSFLWYKMYSCFIGISSVAVTIFVM
YKKENTSGPIASVHAANTFAHCALTLTCLVMLVSLVYGKSKGSALKKMFEAMDDIDEKLKF
DSFSIRKQMYIGVTIYIVLIVVYVVYDAVTWTLLLGISWYILYMPKTIQICQFSLGMLLPMAL
LSLIYLRIKGFNKALRHYSKVNFSQISCYSLNEQRQFFFKEMCHMHTDLFQMVKSFNRIFGI
VLLFGTLMAIGLILHYIVILLVYFEFNTELKGQSNHYVLAQCITGILIPALQVLALANVGDRV
SFEAGKTSNITMVLLNKFPSHGTTEVEKAIYVELKTLVMQCSFRNIQITAKRFFSVNHTLLG
VLLSSMVSYIIIIVQFLSMNNEQ
>DponGR12
MAHYSAVVLTLNNVFILFPPVKGSSSRCFALFRCFSWLFLAFAAITGGPMSIIIKDQVFSNVAT
IHKVIYDVSAMLLFITNIYLSGVSILRHRKLSQLLEDVLVDISNGSTFLINFAAFILIHLVNTLFI
LVTQLERTDNIWYQMTILFVDGYMTFRVITGLFIIFLIINDAKNKLAELNEKLLRSTTATSIRH
WRYRHNNLCDHLHNINSLFGVVLLLIIFYFITTFLRHAVFISVHSEANLVVAVRCLWIISYGTF
QFQAIYLGYMGEQIVKESQKTVAIGYKQICQWSRLKIPDQSSEIFLLTQQAFNRITRISAAGFF
NVDNSMILFILTTFFTYFIVVTQLMAGGRKPT
>DponGR13
MQLNCQNSLLYIIRVMLTIEGFVYMLWHPMMQKRKMNRCIWLFCILIASLYYIIQKIIGFISM
ETSSIFNPDALTVVTLITSSSIILINACVTIKLFDNSLSSKLLITHFIGVLWNNKCLPISQALILIC
FLCATIVIGVIDIFIEGTIYYCWIRSLAMLFWLFMLIWETRLCLRQLNKDLQRASELSSLKQIR
SDHSYVCSVVGALNKNYGIVIMISVFSTISGGCQTILMIPMDVPFAEKILLYVHYFSFTLYIVL
ITIAGESLKKQGEHTLTICYDRLQEFGLSKDNSFRKQLLNMTQQAFEQKLQISASGFFFVDH
SLIMFICINTCTYMIFVFQFMTETSSAKSMVRPTNSEILINSTVH
>DponGR14
MKPIFPTMLIYISTIMGLCPPIYFKQRFAAKVLDIIWILFWTGAVIATCVSFQYTRIEQPFLAM
KILEICSHFFQCALAIVVSVAVLKNGWRIKRLLNVLRKIDYHGGMPKKCDYVSRKIVVVWF
FLLFVKMFFTRDMFVHMWIFACAYICFLRIDVLLAAAVLVLYQIIMKYHCLNQQLNSTDNN
EELIFLASTQRKFFYIIDEINRVFGPALFCSILVAISLILIYLSLFILAFTDRMVLVWSSNVSLLIDI
TYLEIHVCGVILLADKLSSEIKKTVAVCHQKSQRCALSRADEILSDRFLEMAQQTHLRQVEL
NAAGFFVLDNSAYVFIISTICTYLTVLCQTTFFA
>DponGR16
MTRNYMKYVLLLFGLYPPTEICNKKTQIFYSGYVIASTCGMVARKLFVLGSFGKEKEPLPVL
VIVFTRQILISLTNIIISTQIISQGKKIGHLLNILQYMNNKYMKFGKSRRWTKLFILGYFLLSGT
RFLYNYIMLTQTQGFLYLLNYHVTISRIELMLTWISLLIFKTQIAFAFTNANLHLSANSLAPILT
CIDSINGAFGIIIAMAVFLYTATLLFGLVSLVIAISDATTSFILEVLLYFVLSGVYIFILAALGEQL
INEKRICITICYQMCQRFNHLSAFKEGKLMQSRFLAISQQLHQRSTTLDAAGFFTVAHTMIIFI
LTNVCTFLIVATQML
>DponGR17
MRNNIVSLPLYLLLSLYCLQVIQNPRFRPYYRLYEWLWILLFPCAQSGYIVWRISLQKPSVM
QIPGTLYDVLLLLCNLSISTVLLKNRNKLDGLLHAMKCLARNTQRKGSRVFAVFLIAAAFVL
IFFRVQVVLSNFGTSNILEIFMAFISIWLAQRLEIMLVGICWIAWELQLYHGDFNKKLQQIQG
FDSLRPHLQVHNRLCRAVLDFNDLFGYPIMFSVFTILCLALGILSKMISSNADVCERLNTSDV
VRRLVYLAPYLSYIIALSVLGEKLRREVRKTIEICFSYSEKSDISYVIGPVSETKEKLQVLANQ
VFQRSTALDAAGFFSLDSSTILFVVNTFCTYLVAIWQFM

>DponGR18
MANKFVAFSVSVLLTFYSLPGGQKYSRRLSYRVYEVLWLVAFSFSQFGYIVWRASLGNPLVI
QIPGSLYDVFLFLCNISISGIALRNRSKLDNLLHANTFLAKVAQWRRNSVLAACILIVAYVLII
LRNVAVEYVFRNSSKLAILILISVWMYQRLELLLVGICWIAWELLQFHRRFNKKLQKIQHFD
SLRPHIQIYIQLIQAVADFNDLFGYPIMFSVFTIICFVLGILSQIIFGRMYYFETMDLVDVVGRL
IYIFPHLSYLVTLSIIGQKLRREGKRTIAICYNHYEKSEEDCMIGRASNIGKDFLALANQAFQC
KITLSAAGFFSLDSAMILFMVNSFCTYLVALCQFMSGNTKYRRRV
>DponGR20
MLTEMLFLKPPALCQKGKLKVAYLIVLASVQMLFFCISAMFCMGFGTNISGIKRANLIIVKTL
ISLVFNSLTNLVITVQLPSRTGTVRSIPIAAEVWKCRQPSFNRASLTVTAAWTILHMCKSSTLL
LAIGHIPLLNLFGLFAYRLLFVRSQGTILYICLESWKLRVQIACLNAQLKSWHTLKGLLQLSQ
QHHLLLSALQELDSVAGPIIACYSFCCIVNLISTCVDLVLPQELNVNQVIVIWHVLSAITFSIDI
ICMANAGQKLTATANETIPICHKMCENLDKALILSERQQLLIQYQLIAQESFQRDTKVHADG
FFSVDHSMIIFIVFNVCNNLVAICQYIAKQ
>DponGR21
MENRAYGRKYPVVVFVAKLFNVFPIQYGNIYSKYLIWAAAVCQIILLTTTCLTNLAWKRQVL
KHQNIERATDDLAQLLILICNVLNNITITFCKHDEILAFLQSISSQDRCIQFSSQNKWFPWISN
GMLIFCCSIFLFEISYGMQMITVHKLFITGMLERFIQLCQLNAVLLIISMVINEITHRLENLNQ
MLLGMNDYFTRTSQNQVFSINDDGRPSEIFAEGTDNPSFCACEKLVTLRKCHIFLCNKVDEC
NQIFGVIMFFLIWVITITWLDASVMLISIQVIGNFQIDGMLDIKSNMGFLIFMGLFGIAGSMV
QAVILACTAEKLSVETKKTSIICCTLQADFSSGRKFNNQTCYIRELGVLNRQSFQRQPCINAA
GFFSVNHKILSRIMANVCSYLLIVSQFLIQKYRDNKTRTKHE
>DponGR22
MVKNKVQPGEKAPIFKERHRHNMEYFYIKQTLKLLEKKHVVYPMTVVLLVILGHIYSLIGK
PTPFPNQHVAVKTAEHVADFCLTASIIIIILYLPKNAENFYQLFSSLYPEGNKLFGPLLAMKKH
HFWRNFLICNVCIGSLITFDASFFLVNFGWSTYKYLVFGDVQFYLYNLVLLLLLTLARKLEL
RFAELNELLEYKTDNFLKTTSNNKTSENEIETHLSYILSSEHFYSVRTFKMMHYELCKLVKR
FNEMFGRIILFTILFSIANILAKVTFIIDFYVTPRNDSDKNKWALFMGIICFWIFANMAQSFLFA
FYGEQITKEGEKTTRTCFYLLNKVPFQPKNNDEAFLQEELKCFAFQSYSHIPCLSAGGFFDV
NFRVLGLMISSVTSYLMVIIQFLLRQQTS
>DponGR23
MANNHIELLRYAIVLVLITGLGLSLYGRHGTIYRTEVPTIILIDYAVLFLLAASNLASALTLCR
KSSVSKFQLFHQPTKFVTRSAYMFFAFGLMIASGACYLSVAGWAFYKYHIYRDVNFLFYCA
FIYNLHRLAGKLSKEFLDLNQTLETVCNFRRFDVFANALEVSRLMAKRRALKTVREVSRR
MNKLLSFIETLNRTFAPMLILAVMHLIVGMLRAVVIMVQYGIKQNTLNGNKVTYWVAVIYP
GWFLAYTFTVWLLASAGQKIENQISSTNKVCFNIINASLDWNGEEDQQIHMELLELHDLIKS
RHISVNAAGCFEVNFNLIGLTYSALITLSIYAVEFLLYY
>DponGR25
MQKGMKSVHSLAPLSRKEITINPIPSLGLKFLLLCVNQRYGHALLMVAGALYIFMFGYTTY
GKTLRGEKTDGIIILTDHIHNFVLTLSNISTVLVVRNRQTRENIYKILSFILSQKYRKNLFEKLG
IALAITCLAACVASVLMFGLYLGWSSCKFYSCRDIQFCILNLTLYTISQITYYFKEMFSAINEE
FLSMYAEHLKSCQQLTNMNPWCELTEPKEVITYIHKTKKCISQVTRISKRHNELCNVLDDFN
SIFGTLIFMASLNITTNTVWSITLIAHFNVPALLKSLKGFDVPVMLVISSLLMNVIWIFLMVAQ
SVLIGSAGRALTIEGLNTSKCCLRMVSHQRYEIKCKEELCLKQKLISLAKQADTRCPKLTAN
GLFLVDLSILSKIASNVFAYTILIIQFIFNNAKSGIEF
>DponGR26

MDYSYINSYPYTQYDPIPVRCLVFFFKHSKIQWIVQICAVSLHLTILIWGQVEKSQWFFARM
HIVEESLKTVDFITFALLVTTNIMCLAYVHKNIDIFNAIFSYQKKLSNKRPGRNNIIANRLIPPL
LMVLIPTDIILLLLIVDISEFKYLFIFSIEYCSFAMNISMMICVFGLLKYILQTLNNFLAEQDSK
VLVEKHSTNERFFSEYDILENMKLIVTIHNDFCDTLDQFNNQFKPMMVLLVLCINATILWNI
VMVIKFDMEFTTSEINLPSSFVYSGLILGAVLALGQGALAAWVGEQLAEEGYRTTKICLSLL
HSLPINLSSANGKLIEKELYLLLDQSRSRKIGLYAGGSFQLDFGILGSITSTVATYSIVIIQFLLK
>DponGR27
MAHLHTFKYKHSKSDPLPIKGLAFVINHQKFRWVVHICTAILHLDIFLYGFFGKTVIIYPQW
HITEASLQVVDIMTFCILLVTNLMSFAWIHRNVELFNLMFYLQENSRNAKPSTSSSSGTFTIW
FLIFLGIVDAIFSFLQFEFTSAIRYPVAHNIEFWCFAMNICTIMYCFHRIKQILGNMNNYLLEQ
EYRNIAVKTKNSISLPTTQLNYQHRAMENLKFILRYHNDFCNMVIKFNMKFKALLTVSFLCI
NVTILWSVVLIIKLSMLVSAGMTEQCITAYLSLSFRAAISLSEGALIAWVGEQLADEGHRTTK
ICFQLLNALPCDLDALDGNIFEQQLNLLLDQSRAHNIRLHAGGYFQLDYRIFGSIATSVATYT
IIILQFLPN
>DponGR28
MDINFEYSSNKSDPTPLKFVVVWIVKLLRHKKCKWLVQLCNLMFHLIALIYPSIDKNKTYYS
TVKITEPPMKFLDYFGFILIAMTNFICIATVQRNVDMFDLTFILRNKLRNAKSSTKFSRWLSIL
YVLVIIFIPFNIAAIVLQYEAGKVKYPIALSTEYWCFNLTIGLLSCIIHYIKEIFENANSFLTDRIL
ANFYFTGKIINSVIINDMRFITKHHNNICNLLDDFNNAFTPLMAFVLLALNVLILWDFGMIIL
LRTKLSTEVIHTHVYIFGVAGLIVFGQGAIAAWAGEQLAEEGTRTTKICYQLLNSVPSNMSSE
TKIHVEKQLRLLLEQSKSHKVSVHAGGFFQVNFGILGSIASTVTTYSIVIVQFLLKTNNNTCK
QD
>DponGR29
MRTVKKMFGKLFRAKEIELLIKALSYKRSLLIVQVVTTTLQVAAYSYSTIGKCRIYSQLQKF
NSDVVKLVNSLSDFILLTNNVVAIFSISNYNIIVDLTKYAWAKNMLQSKTTRLSVQLLIWGLR
LSVFIPIAINIYFLWFCIGWNMYKYYLNRDISYWFHNLITYMFLTTAMMVKHIFSCINEQLRK
CRENYISSISLTQISTVHDGKYSVNPPSYFTSLKMITEVHNTVCDFLECMNQNSKPLLTMLM
FCIIGNIFYDCTILIEFGIKPKIIGNIDMKSYIYTMHFFFAAISVAQVAAAAFVGESLEEEGQATT
KLCFSYLNKLPKEFKTQTDLLIEQELYLLLDQSKTKRVCLRAGGFFCINWGILGSIASTVATY
SVVIIQFLTK
>DponGR30
MVAFVKNYPFEIRLLLLSLHYKTFFKIFQIILIILNVSGFIVCIIGRLALYHKSEYFKNEMVIFIS
CFSDFLLVFVSIMGVVGFIRSAFIVKLFTFLSKRKFTPKESNSSHSRIICAMRLAPLAPILVEGF
FLQDRVGWHLHQYFVPRSIWLYCLNLMVCTCAQLVDKINIHFIGMNDYLEELGRDCPFAHQ
RVFYIPKFYTRKNRISPELSCSIRDLLTLSRHYTVLCNLIDRFNCFIKAFLAIMLITAIVNILYNC
TVLISFGTKPKSGNTLLYSNIFVFAIQSMSALSTVGQIAVSSLIGEKLQGQGERTASLCYTFLN
KINNSKQYPCDQLVVQEITFLLDQSKTRKLCLHAGGFFQVNWGLLGAVTSTVSTYCIVIMQ
FVSK
>DponGR31
MWERVQLVCPAFPELQLFLQARHHHRLFRCAQVALIVAHVALFGYGQLGKPLVYGRFLPFG
NHVVAFAALMNDLMVLAHLVFTVLATTSTRALADLIAAVLQERATKSHCAFHLCVALLRLL
PIAPIGFGVWLYWDLYPYLLHRDVCYYSLNLTIYSCAGLLHKINGSFGAVNDQLEQLGAQL
GRQHWRLILNRLARLRASFNVLYQLLDTFNSYVKALLVVVVTCISINILFNLTVITQFAVKPG
DAYSTTLIYVIHALFILMSVGQAAVVAFVGEGLEEEGERTTRICFTLLNQLDHAQGKSEPLVA
RELRFILEHSRARKICLHAGGFFRLNWGILGSITSTVATYSIVIIQFLLK
>DponGR33

MLKTSTVFCDDSIKEPLLELCKVGAIIGVCPPIGLEKGTVRYGTLFRLKIFSTTLFVTILGLGD
LWGCELWGGLHLVNVIRLSILISIIITLEPLLGTIVRRTIWIKFYEKYVMLLQNPSWTNLTKNY
HIYRNIKFYLAVYMIITVTVPTQGYILWIREQDMDFEQKLNIIVCLLGDEATLFYYNFVKFIM
CAILNDLRIRYNYLSAKINVLGATQSRRRILADIYDVEVTALKINDVACLFEKLYGAHLFLM
MAYAFSIGLEFFIFTYNLFSADIVYLYLIILIPILSCIYSCDITYKAAQNLSANLLAASKSFEDDI
EIIEKLKDVSYKIQVLQNGFSAAGFLKIDRKIAVALLTAVGSYFLVLQQTYVPENKRT
>DponGR34
MESWQASSIDGLEKQTMLKLLKFGRFLGIAWAPLPKGKKRIILTIFCSLKLGLMTVLSLFFLR
ARFHFMVAEGVSKTLILITALTAAASWVVCMKCFAEVGYGNKGSFYLQKHFKEVTEVARN
SGFEAKKASTILKYYSLMVKVILLIYVLAKVCVVWQHNQWFKAFPQLATFYFPEFLATFILI
YKMSVLIALLRSLKNKYSFVLDTITELRNCEKHLSDFYKMMKIKKLEKIIRFLYYLMRSINE
FFGFYFFICVMLTLFGCLYSVAFVLSYRKLGKIRYEQLYDLFVDSVILLVFIVVIILQADTLQAI
GSKLPIVINLLRSQTNTKPIKEELGFFGKYSKELAPMFMACYFKVDKHLLISCVSAFSTYLVV
IIQFSIDAPDLKAGTFTVGINTTHD
>DponGR35
MTSSGSQLLHLSRFFTFLGILPPYSASTRKVLTTQYHLVKVATLIAVYTALNFISLVFRQMYH
QRPIVYNIVECLTNVSLWLLVVATIGYSYSRTNLFQELYDELRVISVKLRENESGAEIGNRKLL
HCYIKVMVLLVITWISYYAPPGFNTDVQYLFISLMYIQQEIVFYFYCISTCFVLETLQSTCKTL
YQDLNRKIWKIRSSRIFDDTELLKEIKTCADIFMRLSRVVKILSKLFAPIFSFLPMVCSSWIVH
FSLYMKHWGHTYFTSLGDKIGAFIFISILLAATSTIMIRCDQIVTESKKIMTTCYDLQDCLSIY
SKPYAELQEFTKKIPSIKVEFTASGLFKVDRSIIFSVMGSVATYFIVVEQFLDKNGK
>DponGR36
METLNYSRRSIAFFKPILLVSSLFGLVPPYDFSEKKLVLTKCYIGYTLVLKGVLLYSMTVGFG
NKYQNLPSGQSQLFFAFSTLVMVLGGATILQVMTCSYQSRLKWQDLLQRLSFLESATFLNT
ENEKEPNIFKNALLHFLFGNIFLAITIMAYYFFFDELDASDGFRIAMFFYNFTISMIINNLCVAI
KLKFAGAHEKLKSCKHAQFHNAIEITRKCRIIYHEMYLIVDIMNGIFGPTIFLMHFLYGIQVL
EVSTTLFVHRKSELDLQSQPNFAIVLVLSVLFLCIPVITTMCCDFAKREAQTLLKTCRDLQKP
LDIFTKRHQELEDFMDQMNNTRIEFLAFRCFEVNRKAIILLLTVTSNHFIVIVQFLNTS
>DponGR37
MLFNRRKTSILICKSKFAYYSVIGIVPFYEFHENEIKFSKLRYFIVISFLFFYSLTIYFGIKHRVST
FSNKIEDRFAQFFAEIFICLSSYFIITNSFISTILNKSSWQKLLSSIVSLEFRIMPFAPTMSKNKSL
KSFVVLVLLHAAVIGFVVLWSDYKFEMWFLVYRYFNMLYSGHITLLFTDCVRSQELLFSFLT
KELLKGNDVHCLHTTKKLRKFRKIIFDSQEMSVIIEDLFRNVLYFIYAYSWMCGVHLTSNIY
REVISQQQFAPAPHHIAAILIQFLFLSIPWIITYQCGNASSEARKLLHTCQNLIRNMNILSEERE
ELQDLVEQLRNKPVKFTLSRVLEINNTIIMRYVSTICSYFIINVQFIQLEIQK
>DponGR38
MLKSSRNDIVSIKPLYIYLNILGITPFFDFQTVQVKHKVAQVYLVIFLTALAIYLAVSGINDMH
ILRWNTMPASEFLFFIICFGIATYTRLISLLKCQLWEELFKSVYFLESPMNLNTRGNLEKVCY
KRPFCQFILLLSVVFSACTINFSFSLRSSCNFFFYRTTNFIGITSVTIISCELFKIILQKYSDINSLF
RSTQQIPAKTGQHLIRKCRIMYQEIQKLTKIINQLYGGYFVMIFFYSGYKALYLAIAMYRWY
SLGLIVHFPNNFLRVSLVTNFLDFLIPLFVSLFGHQANSQAKKLQKTCRFLQDSLDPQSERFV
NMAFCFLLNIEHQQVKFTAERFFDLERSTIIRYLHVTSTYFIAFIQFVILTK
>DponGR39
MFNLILETFLNIFIKLLQCWGILPYKDYHKNGTRELLLLTKNLTLMVAIVLYGLSGTYFGLVY
IYTDFGNMYLLVDVLKNIAAIFSSISFIPAVFFRQSKWTLLLDAAIRMKSNLRKYKKLISVLM
LYIVIVHIFIIATFTSARSHVRLQKFLKYSELFLACTNFMIKFPLVAYYFENLSIVIMIVLLLKIN
DKLAAMQTNLKKLEKFQFHSLTTIKHSKRLYLSAIKLKVLINDYFGLKLLTIYANSTVRLLW

FFTLSIQMWNPDKFANSLIVDWKHLATSVGFSILGMVPACVITMTCYAISKKADYLLEDCYR
LRYKFSYESYEHRELLALTTYMSENRLVFTAVDYFIIRPSVLLGIIGTSTTYFIAIIQFS
>DponGR40
MSSQWLFETLFKILLKVVYVLNIVPMPDQQVNITYCLKTFIYFASYTFLLVTSVQYEYNFNY
GLSKTEIFNICIAITSVILSYLILMLILYSYLRNSLWQCFYQKVGKFEYGQVNEILSFRNITLLL
FFIFEVVGVLVMAHYIFIIYEQTHQSAYRTILAVSCPCISFCYQICQWTVMLLFIKNIRDQVEK
SEKHLDLKQLNVKMAKHLFLEARLLSVLFNRIFGYQLLATFGQWILFILEVGIQSDIILNPEK
FNAHFESPISVFFVSLSGRIAATAFGPCSIAFACDLVTLETEKLLNSCYQLQEEFDCKSHEYQQ
LQSFGSYISNNGIRFTAAEFFEIKRSTILSVIATSTTYFVALVQFF
>DponGR41
MIIQAGKFQAPLFGPNLQILKYFGILPNMNFQTNRLQGRKFSLFYVIFLLTVIVLMEIYIWVH
KFYMDYSTESYLPVIVRVLCELTGFSMFVCVVTWSYTKQKLWEKLFQDITSIYIQQIMRETQ
ERKKSVLRKANLYFVLISFLILLLYCLDIIFFRIEYQSINFILSYVYFYYNFLLSNVIGHIASEISS
QYKYMTNAVAASEYTNEQDCKKMLSETRKLYTEMHKVIETFNTLFGYVLLLMAVQCSLQI
LDFGVFLIEMVLPNFKFEYDAFFIYMGFILLDLIWLSVAQSRCEMAKDESMKLRSACYNLL
DNSSGSGDLHQELKALIKQIKNRPVRFTAADFFEIKRSTTFTIVGATATYFIVYVELRSNENTT
WNNGTL
>DponGR42
MVLSYSRKSLMSVQFMCNVCAIFVVVPLFSFQEKRFLRRKTFLVYLSFVAVIMIASELETIYS
IYLIQDFTLVHLVAVMGEICGVLIWSTALYQTCFKWHVWESLFTDILSLEDVAQLNTIRTKER
NVLKNPNFQLLLSLMIIIALLVYERITFRGEEKKVFLFNTSYIYFFLHFILSTIIGNIALVLKNKF
HDINGLLSSYEADQSTTLIKGTQKVKDLYSSMTDVVGRFNELFGPQLFLMGLHCSSQILQW
SVSLVYSMFTTKDDNVNDYARLAVGIIYIFLMLIWLGITMFCCDMAVLESEEIITICYRIQLKY
SVFSEAYQSIKNLLYLVTNTKIRFTAMDYYEINRSTMFDLIGTTATYFVVLIQFYDGNKKTS
>DponGR43
MVFDWLMLQHRKMSSLALTKPRADFVIVQRWYRLAKLLGFTPISTENSERKLWNYWAIVY
PVLLLIIYLGGTIFSFYERFRIYESFNLSQTILDILQGIVESVFIEHAVIWSLLNKKHWNKMMR
SIEQLEQKLHWTCTTEVTDYSTAGLRLRIFFYHAVYAAVHIYDSIVNWMFLSYSLAFIIFRFT
TYYIMFTTLFIDFVCTWARNRYLYMHNYLRVYGTARNIKFSIVQKEHVLSKLAEFSNCYKH
LFLIVEEINTIFGTYFFFVAICTVLEVLNAVNYGMPSKNQDIDQAVTGVNVLFLALYIICNVQI
VMACNSVLKCAKSIQKTCLILHEQMENRELREEFLRLSYYVQGLYPEFSAAGFWSVNQSIL
STLFSSVMTYLIIIIQFNMTLK
>DponGR44
MQADLKVIIFIHRIAQYMYLSPCVSKNKFVFRLVGAFLAIFSIPLGCVLLRPRVTFFKRLPTSC
FIMDLANFFMGYTCLAIFCGMQNIPRRNWRAKVSSRIVLIERMLTNMKFSFKKNKQIVLKY
WILHGVYILIHSLQLYDWSSRYDFDIISSYLEFRIVNYWAGFSTLMIFEWLNFIRRRYEHLDQ
YVKTVVSQKVLLLPVVVKELRLIGEVYKNLTELTVELNALFSFHLFFVISVSMVLTLHGLSF
SLVLLYTLVSSIAALIFVVVFFGNVLVLVLAYEKLRNKRQNIMKTCFIIAESLAPGEIKNELCD
LTNFMRNLPAEISACGLFEMDFRLISSYISVVFTYLIIILQFNMTFV
>DponGR45
MRTLSSTTKLLKCFKIFCVFPIQFNSKYKKYEKQPLKWFYTICLIFLTAIHVGTIVVLGRFYEG
NASEKVRLSLKIFQQLMSLTVIITCYFIRELLTNTMNLYMEIDHILKKYKATLNYRAVITFLTL
KHFISITCIICTNILCCIWNGSTGDCMIYTLEHFFNYEILTQVSTSLCSLLILSHQRFDILNASA
MNIVMGGKIDEKTGKTMNKITFNEKAPENTLKALTTLHFKTVQVTNMINKTYSFQLLIILEF
TLVRVIENAHYLLNHNRSNTGAMTVDIFWLIMIIIESFITFYLSTKLEEKASKTARIVNMICCN
DNHLLQSEKDMFLLQCLHQRLYLVAGGLYTINNKTYLKIWGTALTYIILMQGLNKY
>DponGR46

MRLSWKYPFIIFPKVSDCLVCSFKIILNLGVLFTSECPHRDKNVLKPRMWSPTWRLYYYALL
CSPVFFSWINNFYIWNYAFYESDGFIQEMFGFLTQSWTLILTLSMLTNRHLCVKYFGGIVELL
SKRKRYGIPTLLTARDVERHQYSLLFILILSNVIGIVTVASRLADDGFGYVNLTQSMSYYLNII
NGAAASMHTVSALGVYEQIFHKLLRLMEKTLETRKNCTNPAFADKLRSYQNYYSSCVINY
QNHVLIYTTKLFLAWHFTYVIICQVFLFLYVSILVIHWDLELKRGATTGVIQSFYVVCASFYV
VKRANNIYNINDDFLTYLHKYPISRLTKKESYLVEALINNLTNYKPNLVCGDILQMHRRSVP
SIISHAITYSLVALQFNLLKYFY
>DponGR47
MLLYRGIKLIQKCSQCFLMLPYSFSRNRFRPAVVNLRFKARLIMLLAFGTIFPIIDYGYYRDQ
YYSLGLTMDSLHTVYYFMKLPVCIMLILLMHIKRREIIAFLNTSLELTADFEKLVGRKIDYKV
LNRWSYILIVFFLFGGAIYLFSMLNDYGHRTWDAIKLGNIFLHIFTQQVKAVVNMHCFIYQR
ILRKHIQLLSQFLNTMQRSITIISREDRFKILHTTYGLYNKIIRQAGNLNAAFGWQILLIVPLTV
LMLLYNIYYLSKLNAENLIGAFHILLEVLLGVVSLVLFVVPSTQTIDEVKAFSDIFQNINRSIE
EDNLLEEMMALFSMQAFSYKIRFTAMEVFSLDVTLLTSVWSMLATYLLILVQNY
>DponGR48
MMRQSSDNKVHPYSKLSIMETPLKVSRHSLCVCQTLKLCIFPMRLIGRFPYSWRHLNGKCA
YELSSFWMLYSCVLMTLLAVASVHVMPSLRFQITNMAVFLDSLANVFSVVYWSVAILYLWN
VRLWLRMTEGLIKASRDHLFCPRAKKLTVKFQKSLLILIASVFTFENGAFMFFAFFTSYKLPL
HVLFFRNMNITPSVNYVFWFSYIQYCTGLFICFENVVCNYLGYRHIHPKKSIDLSRNEYQFS
GLCSPKKMCDEAHFLPEKMEKLTRVEIIEYLRISHEEIYEIVLLFQQFTSPQLLACIVVEVSILII
NIYSAMVYLMYQSDKVITTSTFALNCIYVVAHFIGFFWFFKNINNLMRIVQGLKPILIACSLS
AQTDEEHQQFRIFVNKVTNQRPFSMNDFFNADLGIMGPVIGSILTYLLVALQFDIPAQEHNN
>DponGR49
MKITVAKKHWKIAPLLSGNYKTQSDDCFFQLLKPYWILGCLHGICQHLLVIYTRGIKGKLA
AFFYVVFYYGVFVFGLYREFKPTQLVSYTLMEISVLLSAISVAVAALAALSSCVRHQKEFSEL
RDQLIKVEDGAKSLGIWKSYRRIRAVSLAVLVLTLSEWAVLLGLSLQTGGFTGAIVLMHCMI
QLFPLKVTQIYLLTCCMCILFISHILAVVNANIQPIYQSKLPPMYQMKHERTLEKTVSLLSAL
LRLSKLIERIYGFPLVLLMADLQIILFTHMYIFLETGFGIKALIKSAQVTLAIIFLATVSQMAVS
EFKRTQLELLTLPVNSNVLFRRKISALIRGVIEEQYELSAYGIFTLNATLIQTMASSVGFYLIM
LLQFQLMFNQDQSAKH
>TcasGR1
MNSNTVGAIFSVGKCFLLSPKSPSEKKPSLLKHIFAFILIILYTIAALAHLLYNLPDYRSLSLM
QMTFLV
MTDVALYCSAIYFPLMTNKITLGWYHLIRNLSQVCGTGANRSYSFAGFFTVSYFVTVSLYVI
LGRKEGVS
FFYYFPYRILAANSMNFGMVTGVTVLQMLHQCYHVQKNIVQRRILTKVKSEEIYSLLEPFE
RGLFTLSDA
VSHFNDIFGWNILFSHIIGVCRTLIYIDELVRTQTNFQFSLETGAILGSLLIFWNLDDILDVLLK
IKFNL
MKMGKLQEAFLDEIEQIRPTFSAARFYSISRPTIFSLSNPATTFLIVLLQFHIN
>TcasGR2
MCYFNYSKKDIRSLSFFYKICNIFGIVPPYSFEKPDSQKSLWKKIQGVVLVSIIAAGTAYSIYV
RHTYYR
RLYTITHLVLDYLDEFLIVALAFQVILNSCFCDPAKWIRLNNNLQYIDEVLKNRDSHETNLLR
NVSVQFF
VYVILYILATVFLAYVWICEMGVVTLQAYTLHLFCVLYVVLLHFLTYNLSLGIKLRYDDVNN
LFRIEESE

EKNIIVSLRQIGSLSQLLSETVALFNDVFGTCLILITGKSIVQLLTCLNFITNNLKSENEEFKEK
LLAAN
LCLVIYTLVSVSLVMVTCDACTIASKQTISWCYKLQEQFVTNSEVRTELFKLAQHVSANIVH
ITAGNYFE
INKATLCGIFGTTTTYFIVILQFNQSSAK
>TcasGR3
MPLKLLKLVFKIGGLFAMSPARIEKNGLVFPSKAYSLLWIILLSAGISVTAVYRTTSYKTLSTI
GLMLQA
STDVILFILNISTILVTVTKKYEWNKFIDVLKILRNGSEDMNVFWFTPFLATNVVFVIIMIYET
YLVIQI
IGPDFFNLYAIEYFQLYAQFIVYSMIYSCLNMILDSIQNISKAINSLKPNRLNSFLLKQIKSDFR
ALSQC
VEIFNNIFGWLILLLIGFTFFELLRSVQVMIMGTRNTTVATVIIRSMFIIWLMVSFQIASNKILL
RSLRS
GRLTVFSSVIQLSKKL
>TcasGR4
MTIQLLSLICKFGGWMGLTPVSVEPTGFSPKGYSLLWILLFTLGVTISGVYRTDFYKKLSPM
RLIVQTCL
DFLLLALNISTILTTVRKQQQWAQLIQNLKVVATTKTKAWFLPFVVTNIIFVLFHTYEAFVW
TRIMGVAY
YEQYAVEFFQFYAQFIVYFLIYAVLEMLLQKYKTVTYVMTGQLKAQNSNLLKSVCADLCLL
SECVDLFNS
LFGFLVLLLVALTTLQLLIYIQVIVIGTKNTIETVAYSVIFILWHIVGTFSGIFLCDLIRREIANVQ
VLA
YKIEAKRRDNEIKMFVKIVDASCRNFTAARFFELNRRTILGVSNAVITFLIVMVQFENITI
>TcasGR5
MQHEVVKKFLHSVRIVFVQSEIFCLVNFNHRESYFRLSKAKSFCTFVAALVYCSVTFFALSE
LLTDLATS
ILLKVSSLLIGFCASIYVGTVWINTSINGTKFIEFINKLIEFDVKLQNVSLIINYENQRTRSRIHL
FVRY
VFVTSYLLFDYFVQRNQEYKYYQILSHLSGIFFTVFNVAQCYLTTELVLMLQTRFVILNKQL
TKITVKNF
ATKTQSAVLGKICTLHHHLSKLVTRFNEIFGLGLLLMFAVSFLIITQVIFVICVLVQSEKIVWL
HLVYIS
FLGIICAADVFYICHVCYATIQEVRAKIYFFYLVKFYKVSKSGELIHKIETNEHEIIDKIEMFSL
QILNE
RAEFNAAGFFPIDYTLVFSVSFG
>TcasGR6
MPTRGTISPFHRFVIHLCNVFSEILSCTQILFFMVLTSFMIHHMLRVNIAIAIVEMVVKPNTTT
PTEENF
GPKYAWNEREKNDLFAWFFWGFLITQIPGGRFSEIVGSRIVLGLGILVASVATLLLPLCCNVH
YYLVVAS
RFCVGLGLGVHWPAIPPIAIRWSSSATARTMFMTHLFAGSLGAAIVLPVSGHLIAYVGWPSV
FYVTGGMG
VLWSVMWFYLIYDSPGQHPRISAKEKEILEQKIRNEITPQVRHIPWIKIFTSLPVWAIVVANA
SICFGFY

IIFNHLPTYMSSVHNVEIEKNGWISSLPHLGEFFREKFVISAYFISGRYITTVAVSYLGARMLH
KNKFST
LTIRKFLSVVCSWSAVLLFGMEALFGYHYYVTNFVSITFFLFLSLSIPGMIVNILDISPAFSGTII
GFNQ
VIVCLSGITSAKVVAFFTATKQSFEQWRYVFIIVAIVNFVGGLFFLIFASADVQSWNPKENVS
QKKNTLL
NDTTSKDHEEF
>TcasGR7
MTSKARTFTIKCPVKKRKCTVSNVSLHSLKNKRSGASLQVGILDSLVFKCYNLRRIYYFYC
VSITFNVHL
LFLLCSGYFTVHLLFYCPFIIFTVHFLLCTYYFYYAFIILLCVYYFYYAFIIFTVHLLFLLCIYYF
VVPL
FFLLCIYYFYCAFIIFTVHLLFLLCIYHFFCAFIIFTMHLLFCCAFIFFNMHLLFLLCLDYFTLH
LLFLP
CIYYFYSAFIIFTIHLLFYCVLIILLCIYYFYYAFILFTVHLLLLVCIYYFYYMHLLFCCAFIIFT
MHLL
FLLCIYYFYCALIILLCIYYFCRAFIIFPTHFYCAFSLYPLKSTFYLNVVRPFPERSMGITPMIW
NSTRR
LFVVW
>TcasGR9
MDHNLLRLIVTVGEFFAITPSKNKIVTKIYATCFIPVLMTASTVSVIYYRQPLYVNFSPIKLVV
TIAMVL
IVNLFNCYTVLAPVFCKRQQYGQLMAKLVENHHKPDFRTCGKFLAPNLIYWVVTIYVAYV
WTDILGFNYY
KEYFVEVVQLYFQFYYNYFLCVVVAIFWGKYRNVNFLLAEQLVHMQSRPFVDHDRFHAFI
KRVEGLICSI
KELNMIFNDVFGWPIIMIVLYSSLLLLNYLDDIFKNSFGYDNQQYLGVIISNISVVFLTTVGTT
ILILLC
DSVLEEVKTTILLAYKIRQYAVSKEKKEIYEFINVVLNNSPQFTAAGFFSINKTTIFHMMGTV
TTFFIII
IQFNTNMRV
>TcasGR10
MSLYSSINSLVLISKIFALLPVKKHHRLEKLVPCVYLTSFSVVLSLGSFIVSVYLTTKIDNDGES
ISLAS
GWLDLYLGITIQTLGIISNCINAKQITIFFDKIQRIDKQFGLLGYSINYRRISIFVNLAILGVFGE
FVLV
FVPDYVFFVENEPILYLIFSYCPIVTTGIIKIQFATCAHLILQRVNHVKKILEKETQTVAFPKDN
HFLAL
LDIVHGVHNELCNLCQVANSIYGFQNFLFVLSTFSICITQFYYCYDSGFNSDNLTDVYFTLQ
WAFLQILE
ILALAYLCDKVQLKIGEIKYSVNKLIFRSNDPKVTLELCGMISTYLIIMIQLDLARTVPKFENK
NKETEQ
>TcasGR11
MCQIKNKLLLLDSSTQPNYLILSNKNNTYLVLNKYLLVKHSKPKMPLLPKTPLPLTLLYKLL
GIIQFPIS
AHFSFMSRFLCLPFYSYFFYLSYIYTTYSRKLSGIFKYIDQMAGYTGFLAMLTSMVMFYKRS
NDLKTLLS

NLESIQIYSIKPKERNSNHWVRSGLFALITGNVLFYPFLPSDVSYNLFSFVPLVVNALDHLFL
NDILSDI
CDKFEQINQHFRRQIKSVDLFVIFPLTKAEKVRNLKEDEVTFSVQKIQELSHLHYKLANFTV
KISGLFEI
TTITAMVMWFGYVIDTMYLFIHIRSRQEDTDTLVVIYTFNLFYLCFCFYWLLVMVAMFSRT
QQSANKTAT
FVHEIWNKYALKNEVDKRVRHLQLVAIRLLNTKLQFTAKDFFNLDWTFCHMVSHKWLNYI
NQFEANFR
>TcasGR12
MSSQLLKLTHDALKILGIGPKSNKIYSFLLLTTLTLLVILSSIDRPYLKSYTYIKLVVSVLMDFI
AYFFN
FWTILFSRSDFFQQFECDNEPKLRHYLIFIFVNVFFWVIILMSNYAFTRIILLKSVLEIVIIDIEIY
SQF
LYGFMIYLILDTIKSKYRQMHRLLANYKVITSDEFFYLVSKIESLACELKNTVDDFNDIFGISF
LLIISY
STLHFVNYIDDLFFFRFEKSKFEPFLISNISLVSLIFLFNCTLIIMCDCVRSEASKVVKNAQKLR
HKFDL
KTLVLNFPKFTAGGFFHVKKSTIFSILNTVSTLLIVMVQFDKER
>TcasGR13
MTVRSVYNVIDLLYYILKMYGLAPYTRRKNQGGSYVYVQCKPSRKYLFSILGFVVSLLGLG
MVIFQVNQE
QEMDTLEKTLYVGIIVQLVFNSAIIRVTFARKIGQFFETLEILNLIDKTLSQIGVQISYKFIQKFS
SVFV
AIYILHPIFTCVHIYDQRDTLLTFGEYFGFGVPLLTFQISLIIFLIYSVTLWNRFALINQQISQLN
SHNF
TKIFVKRRIVLFSDLHQRLCKATLFLTECLSPGLLTMYLQFLQMFTILMLIVLNVGLPSYKAD
AMTWTLL
ITGENLAMAIIIELIIYESEKTFRVLIDLENYHRDRNTELKVNTFLLQMLHQKISFTLGGLFEF
NLNKLL
QSVGTVTNFVMFVLQIKHN
>TcasGR14
MCLAIILMNNKLNMVLVKSMLQRIKMEEKLVVEKFLNSLQIYLQHNQIFGFVTFTCTRSNF
RSSKLLILY
NIILQVLFVSFVSYWLYLVLEADDMLPIYKNTYLIILFADFAYLETTWICTLLKKDKLLELFK
RLIHFDT
KCQENSTVIDYKRHKKRLLCYLLARYVALALVILFSEILVIVSEQEWSFSTGLLVMIFNSALS
YKASEIV
VMLRSRFAILNKQIRFLNQYLRLKPEGRISNRRVFISFSKICYLHQHLSKSVKLFNEVFGVSL
LVLFGNS
FLSIVLALFRTAAELQASQIKWTRIAYMALASVPFIFDSIHLCDVCYSTIGTVSWCELNFDWS
SQVSKAG
ELIHQIQTEDHDIIDEIEMFSLQIANEQVEFNAAGFFPINYTLVFSVRSVQVERI
>TcasGR15
MSLKLLKLVFKIGGLFAMSPARIEKNGLVFPSKAYSLLWIILLSAGISVTAVYRTTSYKTLSTI
GLTLQA
STDVILFILNISTILVTMTKKNKWNKFIDILNFFKNGSEDQNVFWFTPFLATNVVFVIVMIFET
YVWTQI

MTELDFFKLYAIEYFQLYAQFIVYSMICSCLNLILESSQNIYKTINFLKPKRLNNFPLKKIKGD
FRALAQ
CVEIFNNIFGWLILLSIGFTFFELLTCIQYMIVGKGNTVPVIIYRVMFLTWLMVGTFNSVFICD
SVEEKV
MNIRMVAYKTAAKCAEETEDMKKLLSAINNFPHFTAAGFFDLNRKTILGFFNAFLTFLIVAIQ
FENFEM
>TcasGR16
MYLFETLPNYMIFVYRSLGIIQFCPKNSHKIVKISPTVLYVCLQLYFLYTSVKFSSDYMTLGF
TGLFHYL
DLLSTVGTVLSMLTSLVMFRKRSKRLKTLLEQTDKIEIFQTNEKSRLSCIQILLFAGIVGLILL
VPFSNS
MIDLSFFYFIPLFINIFDHMFLCDILNFISSKFGSINHHLRHQIKTEDEDIKTNIRRVQELSFSHY
NLVD
LTKKITRLFEITTLAAMGMWFGYMVDGSYYTLHVLVHGSPSFWTDVGFNCMFGVLHFFW
LCFVIDGFART
QNTANLAAIYLHDIWNKYLERGKLDRNVRYLQLISIRFYNTKVRFTAMDFFSLDWTFGHLM
IAAVATYVV
ILIEIEI
>TcasGR19
MHTLQHFTIDTRSTHSMKNFKYLKVLVTFAHFICLFPITINIRKNGLNYNFTKKCYFLRMVLI
DLLIVGC
ILKHIFNILMKNVTLNDVVFIFCSAPIIITILDIEILGYVNKAKFGKLLINLYTINYNFKESERND
YVSL
LQLVFVFCYFIYLFTVYYIYYSVHEASIINFGYTLAKFMIFTSTCIYTNLLRIIEADFSKLNHLL
SGTEN
LDLVLPIYSQLVFMCKKINKLYGHQLLLTILTYLIWTIYEMYHLAILWSCTSTNCPRFLIMLA
LSYTVIQ
EVMLFTILWNCQNTRVASEDFKAIWYSILIKKADSFCKKKLENYSLQLINHRVVFTAMGLYV
LNMEHFFS
VKCSIQSESLLNDILFPVAGFISDFDCDINSIQYFCLMARCLNTLIFYYHISTYEYSI
>TcasGR20
MNQSKISKIAKHLCKLWSLHGLPIFTLKNGKFVNKPTKSYIFSLLFVAVVVAIYTRIKKFDNR
VYSYITT
IQFFSIFLLNTGFIICSHRKRKKIEKIFANLLHAETMVHRFSGDKFPSSKIGRNLLIYIIFQFMLT
LVQF
VSVTSTLSRDNLSLYFEILCHQCLLTYSFNFNIMIFFFLFSLREIGKHFVGAIQAKMSHKVLDR
EFRQVM
EISTLLHNVANDVHQTFQLVLLLKIFKDFILAVSSAYHSAFTMANLTTVLSYLRNITEVGVFL
FATLMSN
FGMVYYFDEISKQDVNLMEIMDEILISFKDDLIVSYKSVELFDLRLKNRNSTFYVCGLFPLN
CTFIYTMI
AGMASYMIYLVQFTQVQQKDSTVSNN
>TcasGR21
MFWEPQDLCELALPLLKIWKYTGLLHYELHGKINSPDRRFVFKSSYFTLLIACLILGNLVVY
MYYFDVTK
DLKSCIVFIQMISNTVQNLGMIYTSHQKKSEFGVLFSRLLKMEKLISRKFGNKFLYNDILKYF
LRVVLPK

YSVVILVFVLNALYLFGANCEDLIYGACFYVGWIFDLNSELLLTFFLITVHQLYKKFNTGVK
NNTNLGLR
DINRIHSTLHDVTIILKDVAQGFIFYKLIPDFTCSVVGLYYLFTNGVDNFLNTFEKFLDLVLSL
LGIPSI
IVSNLYLMYLFEKISQEANETLEIVHKNCYNQKAPKKIFYKDKEIFYLKTIIRPAEFSIFGLFDF
NFPFF
YSMIVAVTTYLVYLVQFRQMEHV
>TcasGR22
MTRAMVRPELDLNSLKCITKVLTILGLLSCSFPKKKLVYCIIFGAIATLTCIEGLRDCPRKYST
PLAKIT
TILQRYCSVLLVFLTYFFNILFRKKLLNAVKILSKIDETLNSKPLKPVKIRTKVYLVYCSSALV
LAAMTT
FHLIYEAHGFSYKCGIQYHICNTVISATVCFMMLLMLEIWRRFTILNNYLTIVLGETWTSLSV
YHLVEIS
EIHFDLCQAADDLNRYFEVQVLTIFGVSFYFFISTFFYFFTTGNIIATYDKQFYIHNALIVRALF
LFFQL
WATVYTSSQVTREVQSRSNRMTPLKLQPQNFSI
>TcasGR23
MKRSHFNKPLEFVILVAKILGIVPLSSGPQRKRVRVLLILKQVLGFCGIVFICWLHFFHYSCTK
IKFSYL
SSTISSILNVGYNVLMCIGTYLISILYEKKYRSVSVVISSFDQSMESRVKSRKKISTVETIVFGL
SVTFV
LVILCVQFGHYFRNYRRNNFCILFTVLSLVITTVVFLLNFVLLIGVSKRFKLLNKYFKSIIFLA
VDYENS
NVSLVRETEECSKLCHMLMVTAELVNQIFELFIAIDFMIFFCLLIFLIFYLSADNDTFRDRFPT
KIEMYT
MFFWTLVAFLKTVLTTYAFKSVATEVRPILVKITV
>TcasGR24
MLWQPKNVIEFATPIIKLWKYLGLPIYEVTGLRVTRQKSYFFPFAFLFLGVNISVYAVYYGVE
NSLRSWL
SVCEFVAIQTQNVSFVLLAYWKRNQIPKFLAKCLATERRVSALSCTNFDFVGTGRQFLLLLI
VKLLLVIF
IFVIDCIFMARDFKDYIFRYCFYTGFVFYGASDFTILVLITPVEKLYGHFLNNLENHNLEESVR
VFFMLQ
DLAQSTFNIIEEFMLAKILIDFFYASTDVFYGTASALEVQTSLLATVLSSFIVVLWLLFVVHSI
VTISFV
FGKVKEQHQVMNEIICERFRSKAAKNYRCEAKLLLRGKVNPLKFTICGFLPLDYCLAYTMT
AGIVTYVIY
LVQFSQVSHCVTCK
>TcasGR25
MDLNIIRRFFQVGKFLALTPTTLRDYSATPWQKNYTVLVFFFFTVGATAALQFCWSDYMSE
NPIALVLWI
VTDITRYVHNLYIFISVMIFKRKLWFVLIANLKKTKFRIDYSEYKFLYCAFITSHVSFSIIMSCII
FICF
QLDDWYTCLRYYFVEFVQIFTQFFYLSFAPIVLKMIFLRYQHFNKTLQTQKTWHQIKTGQL
RLKQTVDTF

CEIFGGTILLNIIYNSSKSLIYLNKLIKQTKSWSSGSPIVILMKLTQIGIIGLFWVGLIFWVIFLC
DAIV
SQNEEIRKNLYQMLQADPIFYENKQFEKFLKTVLSNGPEFSAVRFFSIDRSTIFQIMNSIVTFLI
VVIQI
KTD
>DmelGR64d
MERSVQENTLHYTIGHVLIIARIFGVLPLAGINPNGKPENVRFRWFSPYILFFVVAFTFVIADF
MLSTKI
VLNDGLQLYTMGSLSFSVICIFCFGSFIKLSRRWPHIIRETALCERIFLKPCYANQEGLNFTRFL
RRWAL
ILLVAALCEHLTYVGSAAWSNYVQIRDCNLKVGFVENYFLRERQELFSVFEYRAWMVFFIE
WNTMAMTFV
WNFGDIFLFLMCRGLKIRFQQLHWRIRQNLGKPMAKEFWQEIRSDFLDLDSLLKLYDKELS
GLILVCCAH
NMYFICVQVYHSFQVKGAFMDELYFWFCLLYVISRLMNMMLAASSIPQEIKDISNTLYEVR
SSPWCDELG
RLSEMLRNETFALSGMGYFYVTRRLIFAMAGALMGYELVLFRQMQGAVVQKSICSRGPGS
SMSIFFS
>DmelGR64c
MQQSGQKGTRNTLQHAIGPVLVIAQFFGVLPVAGVWPSCRPERVRFRWISLSLLAALILFVF
SIVDCALS
SKVVFDHGLKIYTIGSLSFSVICIFCFGVFLLLSRRWPYIIRRTAECEQIFLEPEYDCSYGRGYS
SRLRL
WGVCMLVAALCEHSTYVGSALYNNHLAIVECKLDANFWQNYFQRERQQLFLIMHFTAWW
IPFIEWTTLSM
TFVWNFVDIFLILICRGMQMRFQQMHWRIRQHVRQQMPNEFWQRIRCDLLDLSDLLGIYD
KELSGLIVLS
CAHNMYFVCVQIYHSFQSKGNYADELYFWFCLSYVIIRVLNMMFAASSIPQEAKEISYTLYE
IPTEFWCV
ELRRLNEIFLSDHFALSGKGYFLLTRRLIFAMAATLMVYELVLINQMAGSEVQKSFCEGGVG
SSKSIFS
>DmelGR64e
MARTTGDPAKRRRCMSRIKFWRRSRVGSEVVEKDTKRFKLSLIKAWLLRIRQEDYKYSGSF
QEAIKPVLI
IAQIFALMPVRKVSSKFAEDLTFTWFSVRSYYALVTILFFGVSSGYMVAFVTSVSFNFDSVET
LVFYLSI
FLISLSFFQLARKWPEIAQSWQLVEAKLPPLKLPKERRSLAQHINMITIVATTCSLVEHIMSML
SMGYYV
NSCPRWPDRPIDSFLYLSFSSVFYFVDYTRFLGIVGKVVNVLSTFAWNFNDIFVMAVSVALA
ARFRQLND
YMMREARLPTTVDYWMQCRINFRNLCKLCEEVDDAISTITLLCFSNNLYFICGKILKSMQA
KPSIWHALY
FWFSLVYLLGRTLILSLYSSSINDESKRPLVIFRLVPREYWCDELKRFSEEVQMDNVALTGMK
FFRLTRG
VVISVAGTIVTYELILLQFNGEEKVPGCFEN
>DmelGR64a

MKGPNLNFRKTPSKDNGVKQVESLARPETPPPKFVEDSNLEFNVLASEKLPNYTNLDLFHR
AVFPFMFLA
QCVAIMPLVGIRESNPRRVRFAYKSIPMFVTLIFMIATSILFLSMFTHLLKIGITAKNFVGLVFF
GCVLS
AYVVFIRLAKKWPAVVRIWTRTEIPFTKPPYEIPKRNLSRRVQLAALAIIGLSLGEHALYQVS
AILSYTR
RIQMCANITTVPSFNNYMQTNYDYVFQLLPYSPIIAVLILLINGACTFVWNYMDLFIMMISK
GLSYRFEQ
ITTRIRKLEHEEVCESVFIQIREHYVKMCELLEFVDSAMSSLILLSCVNNLYFVCYQLLNVFN
KLRWPIN
YIYFWYSLLYLIGRTAFVFLTAADINEESKRGLGVLRRVSSRSWCVEVERLIFQMTTQTVALS
GKKFYFL
TRRLLFGMAGTIVTYELVLLQFDEPNRRKGLQPLCA
>DmelGR64b
MPQGETFHRAVSNVLFISQIYGLLPVSNVRALDVADIRFRWCSPRILYSLLIGILNLSEFGAVI
NYVIKV
TINFHTSSTLSLYIVCLLEHLFFWRLAIQWPRIMRTWHGVEQLFLRVPYRFYGEYRIKRRIYI
VFTIVMS
SALVEHCLLLGNSFHLSNMERTQCKINVTYFESIYKWERPHLYMILPYHFWMLPILEWVNQ
TIAYPRSFT
DCFIMCIGIGLAARFHQLYRRIAAVHRKVMPAVFWTEVREHYLALKRLVHLLDAAIAPLVLL
AFGNNMSF
ICFQLFNSFKNIGVDFLVMLAFWYSLGFAVVRTLLTIFVASSINDYERKIVTALRDVPSRAWSI
EVQRFS
EQLGNDTTALSGSGFFYLTRSLVLAMGTTIITYELMISDVINQGSIRQKTQYCREY
>DmelGR5a
MRQLKGRNRCNRAVRHLKVQGKMWLKNLKSGLEQIRESQVRGTRKNFLHDGSFHEAVAP
VLAVAQCFCLM
PVCGISAPTYRGLSFNRRSWRFWYSSLYLCSTSVDLAFSIRRVAHSVLDVRSVEPIVFHVSILI
ASWQFL
NLAQLWPGLMRHWAAVERRLPGYTCCLQRARPARRLKLVAFVLLVVSLMEHLLSIISVVYY
DFCPRRSDP
VESYLLGASAQLFEVFPYSNWLAWLGKIQNVLLTFGWSYMDIFLMMLGMGLSEMLARLN
RSLEQQVRQPM
PEAYWTWSRTLYRSIVELIREVDDAVSGIMLISFGSNLYFICLQLLKSINTMPSSAHAVYFYFS
LLFLLS
RSTAVLLFVSAINDQAREPLRLLRLVPLKGYHPEVFRFAAELASDQVALTGLKFFNVTRKLF
LAMAGTVA
TYELVLIQFHEDKKTWDCSPFNLD
>DmelGR61a
MSRTSDDIRKHLKVRRQKQRAILAMRWRCAQGGLEFEQLDTFYGAIRPYLCVAQFFGIMPL
SNIRSRDPQ
DVKFKVRSIGLAVTGLFLLLGGMKTLVGANILFTEGLNAKNIVGLVFLIVGMVNWLNFVGF
ARSWSHIML
PWSSVDILMLFPPYKRGKRSLRSKVNVLALSVVVLAVGDHMLYYASGYCSYSMHILQCHT
NHSRITFGLY

LEKEFSDIMFIMPFNIFSMCYGFWLNGAFTFLWNFMDIFIVMTSIGLAQRFQQFAARVGALE
GRHVPEAL
WYDIRRDHIRLCELASLVEASMSNIVFVSCANNVYVICNQALAIFTKLRHPINYVYFWYSLI
FLLARTSL
VFMTASKIHDASLLPLRSLYLVPSDGWTQEVQRFADQLTSEFVGLSGYRLFCLTRKSLFGML
ATLVTYEL
MLLQIDAKSHKGLRCA
>DmelGR66a
MAQAEDAVQPLLQQFQQLFFISKIAGILPQDLEKFRSRNLLEKSRNGMIYMLSTLILYVVLY
NILIYSFG
EEDRSLKASQSTLTFVIGLFLTYIGLIMMVSDQLTALRNQGRIGELYERIRLVDERLYKEGCV
MDNSTIG
RRIRIMLIMTVIFELSILVSTYVKLVDYSQWMSLLWIVSAIPTFINTLDKIWFAVSLYALKERFE
AINAT
LEELVDTHEKHKLWLRGNQEVPPPLDSSQPPQYDSNLEYLYKELGGMDIGSIGKSSVSGSG
KNKVAPVAH
SMNSFGEAIDAASRKPPPPPLATNMVHESELGNAAKVEEKLNNLCQVHDEICEIGKALNEL
WSYPILSLM
AYGFLIFTAQLYFLYCATQYQSIPSLFRSAKNPFITVIVLSYTSGKCVYLIYLSWKTSQASKRT
GISLHK
CGVVADDNLLYEIVNHLSLKLLNHSVDFSACGFFTLDMETLYGVSGGITSYLIILIQFNLAAQ
QAKEAIQ
TFNSLNDTAGLVGAATDMDNISSTLRDFVTTTMTPAV
>DmelGR8a
MSGHLGRVLQFHLRLYQVLGFHGLPLPGDGNPARTRRRLMAWSLFLLISLSALVLACLFSGE
EFLYRGDM
FGCANDALKYVFAELGVLAIYLETLSSQRHLANFWWLHFKLGGQKTGLVSLRSEFQQFCR
YLIFLYAMMA
AEVAIHLGLWQFQALTQHMLLFWSTYEPLVWLTYLRNLQFVLHLELLREQLTGLEREMGLL
AEYSRFASE
TGRSFPGFESFLRRRLVQKQRIYSHVYDMLKCFQGAFNFSILAVLLTINIRIAVDCYFMYYSI
YNNVINN
DYYLIVPALLEIPAFIYASQSCMVVVPRIAHQLHNIVTDSGCCSCPDLSLQIQNFSLQLLHQPI
RIDCLG
LTILDCSLLTRMACSVGTYMIYSIQFIPKFSNTYM
>DmelGR98a
MEQMSGELHAASLLYMRRLMKCLGMLPFGQNLFSKGFCYVLLFVSLGFSSYWRFSFDYEF
DYDFLNDRFS
STIDLSNFVALVLGHAIIVLELLWGNCSKDVDRQLQAIHSQIKLQLGTSNSTDRVRRYCNWI
YGSLIIRW
LIFIVVTIYSNRALTINATYSELVFLARFSEFTLYCAVILFIYQELIVGGSNVLDELYRTRYEMW
SIRRL
SLQKLAKLQAIHNSLWQAIRCLECYFQLSLITLLMKFFIDTSALPYWLYLSRVEHTRVAVQH
YVATVECI
KLLEIVVPCYLCTRCDAMQRKFLSMFYTVTTDRRSSQLNAALRSLNLQLSQEKYKFSAGG
MVDINTEMLG
KFFFGMISYIVICIQFSINFRAKKMSNEQMSQNITSTSAPI

>DmelGR98b
MVAQKSRLLARAFPYLDIFSVFALTPPPQSFGHTPHRRLRWYLMTGYVFYATAILATVFIVSY
FNIIAID
EEVLEYNVSDFTRVMGNIQKSLYSIMAIANHLNMLINYRRLGGIYKDIADLEMDMDEASQC
FGGQRQRFS
FRFRMALCVGVWMILMVGSMPRLTMTAMGPFVSTLLKILTEFVMIMQQLKSLEYCVFVLII
YELVLRLRR
TLSQLQEEFQDCEQQDMLQALCVALKRNQLLLGRIWRLEGDVGSYFTPTMLLLFLYNGLTI
LHMVNWAYI
NKFLYDSCCQYERFLVCSTLLVNLLLPCLLSQRCINAYNCFPRILHKIRCTSADPNFAMLTRG
LREYSLQ
MEHLKLRFTCGGLFDINLKYFGGLLVTIFGYIIILIQFKVQAIAANRYKKVVN
>DmelGR59a
MKRIGQAYNVYAVFIGMTSYETMGGKFRQSRITRIYCLLINAIFLTLLPSAFWKSAKLLSTAD
WMPSYMR
VTPYIMCTINYAAIAYTLISRCYRDAMLMDLQRIVLEVNREMLRTGKKMNSLLRRMFFLKT
FTLTYSCLS
YILAVFIYQWKAQNWSNLCNGLLVNISLTILFVNTFFYFTSLWHIARGYDFVNQQLNEIVAC
QSMDLERK
SKELRGLWALHRNLSYTARRINKHYGPQMLAMRFDYFIFSIINACIGTIYSTTDQEPSLEKIF
GSLIYWV
RSFDFFLNDYICDLVSEYQMQPKFFAPESSMSNELSSYLIYESSTRLDLLVCGLYRVNKRKW
LQMVGSIV
VHSSMLFQFHLVMRGGL
>DmelGR59b
MVYWMIKLYFRYSLAIGITSQQFSNRKFFSTLFSRTYALIANIVTLIMLPIVMWQVQLVFQQK
KTFPKLI
LITNNVREAVSFLVILYTVLSRGFRDTAFKEMQPLLLTLFREEKRCGFKGIGGVRRSLRILLFV
KFFTLS
WLCVTDVLFLLYSTDALIWVNVLRFFFKCNTNNILEMVPMGYFLALWHIARGFDCVNRRL
DQIVKSKSTR
KHRELQHLWLLHACLTKTALNINKIYAPQMLASRFDNFVNGVIQAYWGAVFTFDLSTPFFW
VVYGSVQYH
VRCLDYYLIDNMCDVAVEYHDSAKHSWSEVRWTKEISSYVIYANSTKLQLWSCGLFQANR
SMWFAMISSV
LYYILVLLQFHLVMRK
>DmelGR59c
MVDLVKTILLIAYWYGLAVGVSNFEVDWLTGEAIATRRTTIYAAVHNASLITLLILFNLGNNS
LKSEFIS
ARYLHEYFFMLMTAVRISAVLLSLITRWYQRSRFIRIWNQILALVRDRPQVVRGRWYRRSIIL
KFVFCVL
SDSLHTISDVSAQRKRITADLIVKLSLLATLTTIFNMIVCQYYLAMVQVIGLYKILLQDLRCL
VRQAECI
CSIRNRRGGVYSIQCCSLADQLDLIAERHYFLKDRLDEMSDLFQIQSLSMSLVYFFSTMGSIY
FSVCSIL
YSSTGFGSTYWGLLLIVLSTASFYMDNWLSVNIGFHIRDQQDELFRVLADRTLFYRELDNRL
EAAFENFQ

LQLASNRHEFYVMGLFKMERGRLIAMLSSVITHTMVLVQWEIQNDES
>DmelGR10a
MTSPDERKSFWERHEFKFYRYGHVYALIYGQVVIDYVPQRALKRGVKVLLIAYGHLFSML
LIVVLPGYFC
YHFRTLTDTLDRRLQLLFYVSFTNTAIKYATVIVTYVANTVHFEAINQRCTMQRTHLEFEFK
NAPQEPKR
PFEFFMYFKFCLINLMMMIQVCGIFAQYGEVGKGSVSQVRVHFAIYAFVLWNYTENMADY
CYFINGSVLK
YYRQFNLQLGSLRDEMDGLRPGGMLLHHCCELSDRLEELRRRCREIHDLQRESFRMHQFQ
LIGLMLSTLI
NNLTNFYTLFHMLAKQSLEEVSYPVVVGSVYATGFYIDTYIVALINEHIKLELEAVALTMRRF
AEPREMD
ERLTREIEHLSLELLNYQPPMLCGLLHLDRRLVYLIAVTAFSYFITLVQFDLYLRKKS
>DmelGR43a
MEISQPSIGIFYISKVLALAPYATVRNSKGRVEIGRSWLFTVYSATLTVVMVFLTYRGLLFDA
NSEIPVR
ASFRMKSATSKVVTALDVSVVVMAIVSGVYCGLFSLNDTLELNDRLNKIDNTLNAYNNFR
RDRWRALGMA
AVSLLAISILVGLDVGTWMRIAQDMNIAQSDTELNVHWYIPFYSLYFILTGLQVNIANTAYG
LGRRFGRL
NRMLSSSFLAENNATSAIKPQKVSTVKNVSVNRPAMPSALHASLTKLNGETLPSEAAGDKA
AARSLILNV
ELLKLGYFPAKNKGLLLKSLADSHESLGKCVHLLSNSFGIAVLFILVSCLLHLVATAYFLFLEL
LSKRDN
GYLWVQMLWICFHFLRLLMVVEPCHLAARESRKTIQIVCEIERKVHEPILAEAVKKFWQQL
LVVDADFSA
CGLCRVNRTILTSFASAIATYLVILIQFQRTNG
>BmorGR53
MAHIKDENQSKQQQKEHETLNKNKLKKVVYTLKPALMLENWFGLSDFLLVNEDELVLLM
QTEKFGVILSI
FFIVMFAVFVDFPDTETESIMELMDEVPSMVVLSQYFIASITTSSCLSAIAIRIFETFADLDSML
LITTT
QDFYNKSRYQTNKYLIILGVSHIISSTLDLLTDDEIVWCKFFVLPIYFLQKLEVLTFCKLIVMI
QCRLQI
INKYLTNFIEEQEKNKALVFTLAESNPKKTDKFNWIGCPSPNNMKIRDLATMYDVIGTICSLI
NDLFNIQ
IFMTLVSTFTYIVIAIWSTLYFYRAPNFTFGTLTTIIIWCITIILSVVVMSFVCERLVSVRNNTKI
LVNK
VIMNYDLPKTMRVQAKAFMELIESWPLKIMVYDMFSVDISLMLKFISVATTYLIVIIQLSHFV
>BmorGR8
MAPRSVRSMVGTSKKDMLKGGFYETVRIPLYIYRLIGILPISGLWHRSSKYNRFSLKSFYTII
YAPTIVM
QTFLLLVHIYDLFAFFFGHQRLGRLIYHMNFYTITILIFMGSRKWKNVIKEIETIELTLPRLRN
SKKALA
LTKSFVFAFFVFSLAEVVLILQFTLRLTKQRHVLPGDSGLYLRSYFVYIFPYLYDHFPFSYVM
GFIVQII

KVQGIITLNMVNCSVVILSIYLTNRLKHYNRIVFAKGSKTNNTRLKWVELNLLYTRISNLVKI
IDKNLNP
FVFISFTANLSYICAQLFYILNKLTSSRTVKITSFLEDKRSDWETVLYISISFALVVLKVLLVSIT
AAEV
HTTSREPLRLLYTLPTAEYTIETQRLMTQVYYSNLSLSGLNFFHITRGMLLGMVATLLTYEIV
LLQI
>ItypGR2
LMMTNIIISVYGFTSEVVDHGVKFTFKEMGLLVDAVYCLVLLYIFCDRSHKASENXAEXVQ
WTLMEINLRQVDEATVREVQFF
>ItypGR3
MVETVHPITKVAPAPPPRYSNGDLMEAFDDSGELNSRIFSCLKPAYATLRLFGLMPVTQSGP
VFHVTAKWIIYSFMLLCSLAGFLGYLKYFNIAITRNAEGRFEEAVIDYLFTVYLLPVALNVIA
MYEASKQAGVLTQIVAFERIYTRTFRKRLTLDMGSKQLILISVLLILGCVVMVVTHFTMANF
IVYQVVPYCYVNIVTYIIGGSWYIYCDVIGKVATSIAEEFQFALKNAEHSSRVADYRSLWMM
LSKIIRNVGNSFGYQLTFLCLYLFFVITLTVYGLLSQIQEGMGIKDIGLTITGVSAVTMLYLICD
EAHYASSCVRTYFQKKILLMELSLLNEEAQQEINMFLRATEMNPTDMCLCGFFDVNRNLFK
SLLATMVTYLVVLLQFQISIPAGNDNLTNSTLNS
>ItypGR4
MQFVNIVELNALEFHLEGVLLNLIFLYMAKIWAAFVREWSLIEKSMKYYEGPKNAKIKIVV
AMVSIMGIALFEHALVTSQIAWTSIRNNPLSFFNASREYFVHHEFVEVFHFVPYSIWTGLFFK
LLIIQKTFIWSFIDVFISTVSICFHCKMVQISRKVARLSAYEEKNCMIWRSTREDYTKLSKLCQ
IVNSKLRWLIILSFFNNVYHILSQLFNSLKPNDDAMHKIYFCISFTLLILRMTTVCVYAGSICD
EQDRLITVLTTAPSGVYNIEVERFIMHLDTFEMALSGSNFFKITKGLLLKISSAIVTLRISVNS
V
>ItypGR5
MSVLFWKGIREDYDRLASFXXELDSHISVLVLLSYFLNIFFLLIQLYHGLE
>ItypGR6
MSYNGDVIKYPREITVFAAVCAVVFCVLGIVGNSITILALFRCPKLKSHATTAFVISLCVSDLL
FCGFNLPLTAARYIAEEWIFGDTLCQLFPVFFYGNVALSLLNMVAITLNRYVLITLYDYYTKF
YSKLSICLQLFCTWLIAFLIMMPPLTGIWGQLGLDPSTFSCTILEKDGRSPKKTLFLFGIGFPC
LVIILAYSCIYWTVRNSKLRLRSHEPLPNQRTSKRDRDDRRLTRLMALIFICFVLCFLPLMLV
NVFDDKVSYPTLHVLASIMAWASSVINPFIYAATNKQYRSAYKRLLALVRSSIGPESMHSNS
LRSKQEKFQGVSYKAK
>DmelGR21aNEW
MSFWAVSRGLTPPSKVVPMLNPNQRQFLEDEVRYREKLKLMARGDAMEEVYVRKQETV
DDPLELDKHDSFYQTTKSLLVLFQIMGVMPIHRNPPEKNLPRTGYSWGSKQVMWAIFIYS
CQTTIVVLVLRERVKKFVTSPDKRFDEAIYNVIFISLLFTNFLLPVASWRHGPQVAIFKNMW
TNYQYKFFKTTGSPIVFPNLYPLTWSLCVFSWLLSIAINLSQYFLQPDFRLWYTFAYYPIIA
MLNCFCSLWYINCNAFGTASRALSDALQTTIRGEKPAQKLTEYRHLWVDLSHMMQQLGR
AYSNMYGMYCLVIFFTTIIATYGSISEIIDHGATYKEVGLFVIVFYCMGLLYIICNEAHYASR
KVGLDFQTKLLNINLTAVDAATQKEVEMLLVAINKNPPIMNLDGYANINRELITTNISFMAT
YLVVLLQFKITEQRRIGQQQA
>DmelGR22a
MSQPKRIHRICKGLARFTIRATLYGSWVLGLFPFTFDSRKRRLNRSKWLLAYGLVLNLTLL
VLSMLPSTDDHNSVKVEVFQRNPLVKQVEELVEVISLITTLVTHLRTFSRSSELVEILNELLV
LDKNHFSKLMLSECHTFNRYVIEKGLVIILEIGSSLVLYFGIPNSKIVVYEAVCIYIVQLEVL
MVVMHFHLAVIYIYRYLWIINGQLLDMASRLRRGDSVDPDRIQLLLWLYSRLLDLNHRLT

AIYDIQVTLFMATLFSVNIIVGHVLVICWINITRFSLLVIFLLFPQALIINFWDLWQGIAFCDL
AESTGKKTSMILKLFNDMENMDQETERRVAEFTLFCSHRRLKVCHLGLLDINYEMGFRMI
ITNILYVVFLVQFDYMNLKFKTD
>DmelGR22b
MFGSSREIRPYLARQMLKTTLYGSWLLGIFPFTLDSGKRIRQLRRSRCLTLYGLVLNYFLIF
TLIRLAFEYRKHKLEAFKRNPVLEMINVVIGIINVLSALIVHFMNFWGSRKVGEICNELLIL
EYQDFEGLNGRNCPNFNCFVIQKCLTILGQLLSFFTLNFALPGLEFHICLVLLSCLMEFSLNL
NIMHYHVGVLLIYRYVWLINEQLKDLVSQLKLNPETDFSRIHQFLSLYKRLLELNRKLVIA
YEYQMTLFIIAQLSGNIVVIYFLIVYGLSMRTYSIFLVAFPNSLLINIWDFWLCIAACDLTEK
AGDETAIILKIFSDLEHRDDKLEMSVNEFAWLCSHRKFRFQLCGLFSMNCRMGFKMIITTF
LYLVYLVQFDYMNL
>DmelGR28bA
MIRCGLDIFRGCRGRFRYWLSARDCYDSISLMVAIAFALGITPFLVRRNALGENSLEQSWY
GFLNAIFRWLLLAYCYSYINLRNESLIGYFMRNHVSQISTRVHDVGGIIAAVFTFILPLLLRK
YFLKSVKNMVQVDTQLERLRSPVNFNTVVGQVVLVILAVVLLDTVLLTTGLVCLAKMEV
YASWQLTFIFVYELLAISITICMFCLMTRTVQRRITCLHKVLKNLAHQWDTRSLKAVNQKQ
RSLQCLDSFSMYTIVTKDPAEIIQESMEIHHLICEAAATANKYFTYQLLTIISIAFLIIVFDAYY
VLETLLGKSKRESKFKTVEFVTFFSCQMILYLIAIISIVEGSNRAIKKSEKTGGIVHSLLNKT
KSAEVKEKLQQFSMQLMHLKINFTAAGLFNIDRTLYFTISGALTTYLIILLQFTSNSPNNGY
GNGSSCCETFNNMTNHTL
>DmelGR28bB
MSALRRVRKYFISSQVYEALRPLFFLTFLYGLTPFHVVRRKMGESYLKMSCFGVFNIFIYIC
LCGFCYISSLRQGESIVGYFFRTEISTIGDRLQIFNGLIAGAVIYTSAILKRCKLLGTLTILHSL
DTNFSNIGVRVKYSRIFRYSLLVLIFKLLILGVYFVGVFRLLVSLDVTPSFCVCMTFFLQHSV
VSIAICLFCVIAFSFERRLSIINQVLKNLAHQWDTRSLKAVNQKQRSLQCLDSFSMYTIVTK
DPAEIIQESMEIHHLICEAAATANKYFTYQLLTIISIAFLIIVFDAYYVLETLLGKSKRESKFKT
VEFVTFFSCQMILYLIAIISIVEGSNRAIKKSEKTGGIVHSLLNKTKSAEVKEKLQQFSMQL
MHLKINFTAAGLFNIDRTLYFTISGALTTYLIILLQFTSNSPNNGYGNGSSCCETFNNMTNH
TL
>DmelGR28bC
MDIEMAKEPVNPTDTPDIEVTPGLCQPLRRRFRRFVTAKQLYECLRPVFHVTYIHGLTSFYI
SCDTKTGKKAIKKTIFGYINGIMHIAMFVFAYSLTIYNNCESVASYFFRSRITYFGDLMQIVS
GFIGVTVIYLTAFVPNHRLERCLQKFHTMDVQLQTVGVKIMYSKVLRFSYMVLISMFLVN
VLFTGGTFSVLYSSEVAPTMALHFTFLIQHTVIAIAIALFSCFTYLVEMRLVMVNKVLKNLA
HQWDTRSLKAVNQKQRSLQCLDSFSMYTIVTKDPAEIIQESMEIHHLICEAAATANKYFTY
QLLTIISIAFLIIVFDAYYVLETLLGKSKRESKFKTVEFVTFFSCQMILYLIAIISIVEGSNRAIK
KSEKTGGIVHSLLNKTKSAEVKEKLQQFSMQLMHLKINFTAAGLFNIDRTLYFTISGALTT
YLIILLQFTSNSPNNGYGNGSSCCETFNNMTNHTL
>DmelGR28bD
MSFYFCEIFKPRDAFGAEQTLLLYTYLLGLTPFRLRGQAGERQFHLSKIGYLNAFLQLSFFS
YCFLAALIEQQSIVGYFFKSEISQMGDSLQKFIGMTGMSILFLCSSIRVRLLIHIWDRISYIDD
RFLNLGVCFNYPAIMRLRLLQIFLINGVQLGYLISSNWMLLGNDVRPIYTAIVAFYVPQIFL
LSIVMLFNATLHRLWQHFTVLNQVLKNLAHQWDTRSLKAVNQKQRSLQCLDSFSMYTIV
TKDPAEIIQESMEIHHLICEAAATANKYFTYQLLTIISIAFLIIVFDAYYVLETLLGKSKRESKF
KTVEFVTFFSCQMILYLIAIISIVEGSNRAIKKSEKTGGIVHSLLNKTKSAEVKEKLQQFSMQ
LMHLKINFTAAGLFNIDRTLYFTISGALTTYLIILLQFTSNSPNNGYGNGSSCCETFNNMTN
HTL

>DmelGR28bE
MWLLRRSVGKSGNRPHDVYTCYRLTIFMALCLGIVPYYVSISSEGRGKLTSSYIGYINIIIR
MAIYMVNSFYGAVNRDTLMSNFFLTDISNVIDALQKINGMLGIFAILLISLLNRKELLKLLA
TFDRLETEAFPRVGVAMHQVAANKKMNRLVIILVGSMVAYITCSFLMISLRDTTTFSISAVIS
FFSPHFIVCAVSFLAGNVMIKLRIYLSALNEVLKNLAHQWDTRSLKAVNQKQRSLQCLDSF
SMYTIVTKDPAEIIQESMEIHHLICEAAATANKYFTYQLLTIISIAFLIIVFDAYYVLETLLGKS
KRESKFKTVEFVTFFSCQMILYLIAIISIVEGSNRAIKKSEKTGGIVHSLLNKTKSAEVKEKL
QQFSMQLMHLKINFTAAGLFNIDRTLYFTISGALTTYLIILLQFTSNSPNNGYGNGSSCCETF
NNMTNHTL
>DmelGR32aNEW
MSPNTWVIEMPTQKTRSHPYPRRISPYRPPVLNRDAFSRDAPPMPARNHDHPVFEDIRTILS
VLKASGLMPIYEQVSDYEVGPPTKTNEFYSFFVRGVVHALTIFNVYSLFTPISAQLFFSYRE
TDNVNQWIELLLCILTYTLTVFVCAHNTTSMLRIMNEILQLDEEVRRQFGANLSQNFGFLV
KFLVGITACQAYIIVLKIYAVQGEITPTSYILLAFYGIQNGLTATYIVFASALLRIVYIRFHFIN
QLLNGYTYGQQHRRKEGGARARRQRGDVNPNVNPALMEHFPEDSLFIYRMHNKLLRIYK
GINDCCNLILVSFLGYSFYTVTTNCYNLFVQITGKGMVSPNILQWCFAWLCLHVSLLALLS
RSCGLTTTEANATSQILARVYAKSKEYQNIIDKFLTKSIKQEVQFTAYGFFAIDNSTLFKIFSA
VTTYLVILIQFKQLEDSKVEDPVPEQT
>DmelGR47a
MAFTSSQLCSLLTKFTALNGLNTYYFDTKTNAFRVSSKLKIYCAIHHALCVLALAHMSYST
ASNLRVSVTVLTIGGTMACCVKSCWEKAQGIRNLARGLVTMEQKYFAGRPSGLLLKCRY
YIKITFGSITLLRIHLIQPIYMRRLLPSQFYLNVGAYWLLYNMLLAAVLGFYFLLWEMCRIQ
KLINDQMTLILARSGQRNRLKKMQHCLRLYSKLLLLCDQFNSQLGHVAIWVLACKSWCQ
ITFGYEIFQMVAAPKSIDLTMSMRVFVIFTYIFDAMNLFLGTDISELFSTFRADSQRILRETSR
LDRLLSMFALKLALHPKRVVLLNVFTFDRKLTLTLLAKSTLYTICCLQNDYNKLKA
>DmelGR47b
MQRDDGFVYCYGNLYSLLLYWGLVTIRVRSPDRGGAFSNRWTVCYALFTRSFMVICFMA
TVMTKLRDPEMSAAMFGHLSPLVKAIFTWECLSCSVTYIEYCLSLDLQKDRHLKLVARMQ
EFDRSVLMVFPHVQWNYRRARLKYWYGTVIVGFCFFSFSISLIFDTTRCTCGIPSTLLMAF
TYTLLTSSVGLLGFVHIGIMDFIRVRLRLVQQLLHQLYQADDSSEVHERIAYLFEMSKRCSF
LLAELNGVFGFAAAAGIFYDFTIMTCFVYVICQKLLEREPWDPEYVYMLLHVAIHTYKVV
ITSTYGYLLLREKRNCMHLLSQYSRYFSGQDVARRKTEDFQHWRMHNRQAAMVGSTTL
LSVSTIYLVYNGMANYVIILVQLLFQQQQIKDHQLTSGKDVDIVGPMGPITHMD
>DmelGR63a
MRPSGEKVVKGHGQGNSGHSLSGMANYYRRKKGDAVFLNAKPLNSANAQAYLYGVRK
YSIGLAERLDADYEAPPLDRKKSSDSTASNNPEFKPSVFYRNIDPINWFLRIIGVLPIVRHGP
ARAKFEMNSASFIYSVVFFVLLACYVGYVANNRIHIVRSLSGPFEEAVIAYLFLVNILPIMIIP
ILWYEARKIAKLFNDWDDFEVLYYQISGHSLPLKLRQKAVYIAIVLPILSVLSVVITHVTMS
DLNINQVVPYCILDNLTAMLGAWWFLICEAMSITAHLLAERFQKALKHIGPAAMVADYRV
LWLRLSKLTRDTGNALCYTFVFMSLYLFFIITLSIYGLMSQLSEGFGIKDIGLTITALWNIGL
LFYICDEAHYASVNVRTNFQKKLLMVELNWMNSDAQTEINMFLRATEMNPSTINCGGFF
DVNRTLFKGLLTTMVTYLVVLLQFQISIPTDKGDSEGANNITVVDFVMDSLDNDMSLMGA
STLSTTTVGTTLPPPIMKLKGRKG
>DmelGR68a
MKIYQDIYPISKPSQIFAILPFYSGDVDDGFRFGGLGRWYGRLVALIILIGSLTLGEDVLFASK
EYRLVASAQGDTEEINRTIETLLCIISYTMVVLSSVQNASRHFRTLHDIAKIDEYLLANGFRE
TYSCRNLTILVTSAAGGVLAVAFYYIHYRSGIGAKRQIILLLIYFLQLLYSTLLALYLRTLMM

NLAQRIGFLNQKLDTFNLQDCGHMENWRELSNLIEVLCKFRYITENINCVAGVSLLFYFGF
SFYTVTNQSYLAFATLTAGSLSSKTEVADTIGLSCIWVLAETITMIVICSACDGLASEVNGTA
QILARIYGKSKQFQNLIDKFLTKSIKQDLQFTAYGFFSIDNSTLFKIFSAVTTYLVILIQFKQL
EDSKVEDISQA
>DmelGR93a
MFSSSSAMTGKRAESWSRLLLLWLYRCARGLLVLSSSLDRDKLQLKATKQGSRNRFLHIL
WRCIVVMIYAGLWPMLTSAVIGKRLESYADVLALAQSMSVSILAVISFVIQARGENQFREV
LNRYLALYQRICLTTRLRHLFPTKFVVFFLLKLFFTLCGCFHEIIPLFENSHFDDISQMVGTG
FGIYMWLGTLCVLDACFLGFLVSGILYEHMANNIIAMLKRMEPIESQDERYRMTKYRRMQ
LLCDFADELDECAAIYSELYHVTNSFRRILQWQILFYIYLNFINICLMLYQYILHFLNDDEV
VFVSIVMAFVKLANLVLLMMCADYTVRQSEVPKKLPLDIVCSDMDERWDKSVETFLGQL
QTQRLEIKVLGFFHLNNEFILLILSAIISYLFILIQFGITGGFEASEDIKNRFD
>DmelGR93b
MVYGFTMSGLLVMPRILRCLNVSRISAILLRSCFLYGTFFGVITFRIERKDSQLVAINRRGYL
WICLVIRLLASCFYGYSYDAWSGQYEDMYLRAFFGFRLIGCLICSVIILVMQFWFGEELINL
VNRFLQLFRRMQSLTNSPKNRFGDRAEFLLMFSKVFSLLFVFMAFRLMLSPWFLLTLVCD
LYTSVGTGMITHLCFVGYLSIGVLYRDLNNYVDCQLRAQLRSLNGENNSFRNNPQPTRQA
ISNLDKCLYLYDEIHQVSRSFQQLFDLPLFLSLAQSLLAMSMVSYHAILRRQYSFNLWGLVI
KLLIDVVLLTMSVHSAVNGSRLIRRLSFENFYVTDSQSYHQKLELFLGRLQHQELRVFPLG
LFEVSNELTLFFLSAMVTYLVFLVQYGMQSQQI
>BmorGR9
MPPSPDLRADEPKTPCLVGGAHAFILKISSFCGLAPLRFEPRSQEYAVTISKGKCFYSYILVTF
LVICTI
YGLVAEIGVGVEKSVRMSSRMSQVVSACDILVVAVTAGVGVYGAPARMRTMLSYMENIVA
VDRELGRHHS
AATERKLCALLLLILLSFTILLVDDFCFYAMQAGKTGRQWEIVTNYAGFYFLWYIVMVLELQ
FAFTALSL
RARLKLFNEALNVTASQVCKPVKKPKNSQLSVYATSVRPVSCKRENVIVETIRVRDKDDAF
VMMKTADGV
PCLQVPPCEAVGRLSRMRCTLCEVTRHIADGYGLPLVIILMSTLLHLIVTPYFLIMEIIVSTHR
LHFLVL
QFLWCTTHLIRMLVVVEPCHYTIREGKRTEDILCRLMTLAPHGGVLSSRLEVLSRLLMLQNI
SYSPLGMC
TLDRPLMVTVLGAVTTYLVILIQFQRYDS
>BmorGR1N
DIYGPEITDKDDGALLDKHDSFYLNTKSLLVLFQIMGVMPIMRVPKSAQTTRRTTYNWISK
ATLWAYLVWGLECIIVVKVGQERLANFQIGSNKRFDEVIYNIIFLSILIPHFLLPIASWRHGPQ
VAIFKNMWTHYQLKYLKITGKPIVFPNLYILTWGLCIFSWVLSFAVVLSQHYLQDDFELWHS
FAYYHIIAMLDGFCSLWYINCNAFGTASRGLAINLHKALEAEHPALKLAQYRHLWVDLSHM
MQQLGRAYSNMYGIYCMVIFFTTTISLYGALSEILEHGLSYKEMGLFVIVAYCMTLLFIICNE
AYHASRKVGHEFQDRLLNVNLGAIDRSTQREVEMFLVAIAKNPPIMNLDGFTNINRELFTAN
ISFMSTYLIVLMQFKLTLLRQGARKTVTAIVRAIFNTTITDNGAGGSDEDQE
>BmorGR2NJ
KEQEQRDLLSSQDGDTCEIHDQFYRDHKLLLVLFRALAVMPITRSRPGTITFSWKSTATIYAV
CFYIAATAVVLIVGYERIQILQSIKRFDDYIYAILFIVFLVPHFWIPFVGWGVAHQVAIYKTNW
GKFQVRYYRVTGENLKFPNLKTLIVIISVGCLLLAVCFLLSLCALLDGFLLKHTSAYYHIITMI
NMNCALWYINCKAIKIASQSLSECFQRDVDIECSAQLIARYRYLWLNLSELLQSLGNAYART

YSTYCLFMFANITIAVYGALSEIVDHGFGFTFKEVGLFVDAAYCSTLLFVFADCSHKSTLKV
AAGVQDTLLSIDVLAVDRPTQKEIDHFIQAIEMNPAFVSLKGYAHVNRELLTSAISMITIYLIV
LLQFKISLPKEPHGTGQ
>BmorGR3F
MSFEIKNNFFRTSVPIPNGFPVQTEAKSKNKPIFLDVSPAPTPKVNSPNAIIPMKNNLIDPFINK
DIIYENIKPVFMVLRIMGVLPLTRTTSGVNEFHFISPAMVYSLTVFIILVSYISYLSLHKVQIVR
NSEGKFEEAVIEYLFTVYLFPLTVVPILWYETRKIANVLNGWVQFEVTYKQLSNRILPVKLY
KKSLLIAIIIPILSTTSVIVTHVTMVHFKTSQIIPYVFLEILTYMLGGYWYLLCEILSLCANVLA
DDFQQALRHVGPAGKVAKYRALWLRLSKLARNTGVANCYTFTFVNLYLFLIITLSIYGLLSK
ISEGFGTKDIGLALTALCSVFLLFFICDEAHYASHNVRTNFQKKLLMVELSWMNTDAQTEV
NMFLRATEMNPSQISLGGFFDVNRTLFKSLLATMVTYLVVLLQFQISIPDATQPEIPTNIDDH
VQNITDTTTEASSPISTLMSAFAKRKND
>HeleGR1(a)
MVDNVNNVGKTFSLEDFYKIDNSNLYQYLPYSLVLNVTLQFFSVIILLQY
SLINAFLILLATNFDSNLILFKSVIDERISKFRGTYMECNQFIKSDAEKY
WKSIRKEYNKLCGLFDVVNGFSSSTIFFYILPSIVNIFILISLIISNSEG
PIQFGRNNIFTLICEILRLRAVLIAASDIYESHKNITYTMYKISASEYNV
QSSHFLKQVTLEKMCIKVFGMFELTRESILTAFGTIVSYSVILLQLIPYL
IYFVRFTDGDGIEMLRRINLTVQNI
>HeleGR2(a)
MCSESYPIRKIYMNAEGDQSAVDVKDILRNVRILYILMTDVVKNLNGSYG
VFLAIDQFYVIVTFVVNLYVFFFTGKQDLNLLYFVLVNGSVILVMAFVSH
DVNYEANKIAELMQKLPIASLDMDSKLEIQMLLSQIFATPIHISAAGYFK
MDREQIPSIMSSIATYLIVLIQFSPQIWPETSGGKNGTDPKTSI
>HeleGR3(a)
MEIEYGENNLLHRVKNPHQLITENNVRAQRLSNAGLENLQKGAEEEPDPE
LLERFDSFYQTTKSLLVLFQIMGVMPIERSAQGKTIFRWYSGMTIYAYCL
FLAETVFVSIVFKERLLLILQKGKRFDEYIYSIIFLSILIPHFLLPIASW
TNGHEVAKFKNMWSTFQLKYFQVTGTAIVFHNLTAISYGLCIFSWVLGIA
IMLAQYYLQPDMQLWHTFGYYHILAMLNCLCSLWYINCTAKGRVAEDLAQ
NLHNALESSNPASKLAEYRDLWVDLSHMMQQLGKAYSGMYGMYCVLILMT
TIVASYGCLTEILDHGLSFKEAGLFLIAFYCMSLLYIICNEAHHTSSKMG
PEFRERLLSVNLMAVDSRTRQEVHMFLTAIDKNPPTMNLNQYADINRKLI
SSTVTSMATYLVMLMQFRSTLMRNAALAARRSAMNFNHTATNGSA
>HeleGR4(a)
MGGLQRQLKYTLMLSQIFGILPVSGVSHDDPLKLRFSWRSWRICYTSSLI
IVTFFTMFMAFYNLCSNDFDLSNLTTFVFFSNGFIVILLFLKLAKEWPEF
MYYWHCTEIFIRSKLLLPNFFVMLPFTILTAAAVEHGLFVTYCITYDDFN
SNITINGFENFMSNDMFVYNYFSYTWYLGLLLKFTSFV
>HeleGR5a(a)
MRCWETTEMFIRSKIVVPNIFSYISFAILGAAAVEHGLLIVYSMTSERNK
NNTSGFEHFMTRDLYFFTYINYTWYWGAYIKFISLANTFYWNFMDVFIIA
MSIALATMIKLINDRIRMTEPKYMLWVDLRKQYNKLYELSFRLEEKISHI
ILISFASNLYFILVQLHGSVKKRRSTMESVYFFSSFGLLLLRTFAVCLFV
ASIDDESKTTLKLLSCRNTHNAESERLLQQVASVKLALTGMNFFTVTRGL
ILNIAAAIVTYEIFFIQYINIIGE
>HeleGR6(a)

MDVFAIIMSIALSRMLAVVNDRIRYSNEVKIMEWTEIRRQYNKISELWMQ
LENKISPIILLSFTSNLYFVLVQLHGSVERRNTVMESIYFFYSFALLLLR
TLAVCLFVARVDDENKTSLKLLSCLRTFNSESKRLLQQISSTNMALSGMN
FFSVTRGLILNIAAAIVTYEIFFIQYVNTPT
>HeleGR7(a)
MFTSKLDRGYLNKPKLLFYCSNILGVTPITQYENVIAKIFKKVYPILLIF
LVISTYFYQLVGGIENLKTNNAERLPEACAAFVLYYMNIYITYKFHLGNG
MEKYQELMEKVLRVDEIVSIYNIPIPLRATNFTPNIVLMFALLITGTIMD
TIYILRIDTELLKYVCVDNFYRIRLSCATLLICYMIKRIYAALRNVHDIL
IVLTSRVSGIRLKKTNHMEFLPHADMCLTLNHLSQVHMILTESVAVFNSV
LGFSIFLIDMYAITCFLVVFDETRKHFGNGTFEIGIIVWTLLFVIITLVG
PIWIASICSKTSKESEKTAKLAYNLYYTLNLHGNSASDKMIAENLLMLIK
QTSSCSFKFSAAGFFPVDYAGLYVIINLITSYTIVALQF
>HeleGR8(a)
MYYIHSLVEDLLEKVIAINTLLERTFKRTHLEDLQTLNHLENVLRDMSKT
YTKCVQIICYFNQIFGWQMLFILLNYMFLFMVTYEAALRMASNDSAHLNY
QYYMWMACCMIYAMVGSVLLTLICHKTSKEAGRTADLSYAYFYDLNISGL
NPREELLGRSLLELFRQSRASPTAFSAAGFFSIDY
>HeleGR9(a)
MYRPKLQKLKFLRWFMNVLVVSPIEENNTLTRMYGVFVLILISWSFYFEM
SGRMCVKFVNDMQVSQTGIVIGTLNTFVEMSYNILIALTAILVSNRSKKE
FFEEIEELEAVILKHDANILSNNKFVYRDMFVLHAFDMLALILDVILFFP
TSSKHYRSYYYVDQFYRYRISLQVLYIYCFIKELLQKLTVVNQTLENAFE
LTTANDMESPEVLNKLEGVVRDVSVCCAKFSNIITHFNKIFGWLLLALFI
NCLCLFLMAFDSGLRIKISSQPIELEFYTWVVSVMVYALVGAVLITSICD
RTSSEGQRTADLSFKYFYNLNIGKYNAGQATLARDMMMLIRQSRTSGTIF
SAAGFFQINYTTLFLLLNLLTSYVFVIIQFE
>HeleGR10(a)
MNYNARKVRYYALLHRKTTKMVRHFNLLISPGILGSIAIDVSITLMLSHY
STVLLLALSNNRPANMNYMIFNSVTIIWISTRLFTMLSIWWDVKREADKT
ATYIHDIWNYLASKDDINERTKHLQLIALQLYNNKLSFTAYGFFALDWTL
MQRVIAAVTTYVVILIQFELSSGTKNVYTNTTMMAQ
>HeleGR11(a)
MKQYQNIFESTRFLCTLFKYLGFLSFRYQRNNLKIVQLDAYRLHLILTAI
FDTIAVIFVLLGFYVILVSFNMESLMETIFYVLSTIMEGTMVITNILRSK
IYNNRLLGNLNELSTIEEEFANQFDIRFKHVNVKIISTIWIIMYTFLSVV
APVICIHFSTSYNILELVCGSVIIILLYSSETCVPAIFSSVLAVLIEFIM
ELNDHIEKKILYELPREARANRGDTIQCLKEISTLYRRIFQTMREFRTIY
GFNVLLQFAVTLLVIVLHVFGYVSIYYSRVKYNKSILAIQSVLLFLYVTI
IGVYIVLGELYSLQVEKLKKILFKALNRAKDEQLRKQINALSLQIFHEDY
EISAAGFFVLDLRMVTSIVSATTIYIVILIQYNPDARVFDEYVKESSL
>HeleGR12(a)
MSKITFYRSLRPLLVFGSCLGVISFNVAEGKYSRSGLRIFANIWLILFYL
VFTAHSLYERCDSNTSLLSTTTDIMQVTSATLQAVVSWFVSAIAQEKSIS
FLKRISDLDKTFRKLGVYIYYDAVYKSVIRRLTVRLTITFASIISQLFLY
DYVLLARLIGFNVTYYFPILINVVIVEEFYIYANLLRSRYEILNQHLLEV
QKCADISKEKVSYIKLTGVIGTKLSTLRIVCPIHHELTKISKLLDESFGV

MLLMSFQASFVTIIVSLYDCSVYLQYFNMDHVKELCAAVIMCCTYVLDCL
FICYSCHSTVEAANKSGRLLHHIDTEDAEVKDQIEMFSLQIVNEKLEFTA
AGFFTVNYGLLFSIIGGITTYLIILIQFSADPNQSKG
>HeleGR13(a)
MLYLDQGYSALFETMSVKNLASKFRPRIEDVFDSVYPLYTLSRVFGLINF
KLTTFLDKRQYKVSVPDLFRQVLQLIVVTVVIYSTITSDVVGSFFKQPFS
NMVLIIEEIIGVALSITGTIIGIFYARKIISILNSIYSIDTDFKQLEMSI
SDWNVWYFSIVSSAICCIYIGSMMTLNVLNRHIDVWPTCYEIFSYIVDVI
PEMINMLVQWQYSGYALIIRNRYNALNQYLESLLDGCHHGFIRINVWVSE
NAQLDVKKQRKFLKSTSDSLFIVDTVASYHIKLADIAAILNQAFCVQLLL
LITTSFLGIVTALFLIAINFKQPMGDDKNSYNFVFTAWAFSNTIALLVVV
HATCALCDEANKCPRILHKIRNNTNDAKLHTTIEMYSLQMYHNRLQFHVC
GLFPLDYTLLYTIVAGVTTHLVILIQFNSFDYGKKPLNDTVFFNYTSIE
>HeleGR14(a)
MLFIRIILVIGGMCYVAFLVEKLEYKAGIAAVALKCELYLGTLMTFTVLA
LAISNQKLLIQCIEKIANIDVSMRNIQIHINYSNAAKFVLMQVIFITCVF
TSKVVLQFFIHSAVVLSMYTAFNMFDYINTIMLFQYVDLLLLIRQRFIWI
NRKLRQLAKFNRSMNKITPIACGIYKDRQCYTQIDSALVLINLAKIHGKL
CEVSRLVNRTYRIQILVTVGSRFVMITTQLINTYNIIRDPTRGTSVQYAL
LTIYLALHSSKIFMIASLSENTAAKAKHTGIYLHEIWEVMPSLINEIEYF
SLQVLHENLKFTACGFFVLDYTLIYAIVGAITTYLIIVIQLEKVF
>HeleGR15(a)
MPFKLAKKRISLQYGPIVSLRIFIIVGVMCYTAIRLDDVKFNRRIAALAL
KCELYLGTFMTFSVLILAALKQQSLINIIKQLVNIDEHMKSIKIKLSYDN
RKKFVLAQTIFVTFLFVLKIVLQQYCYTTAILVMYTAFNFVDYINTIMIL
QYVNLLLLIGERFFLLNGKLRQAYRHPVSFDKPRIFLIPKLNFKSADISA
ITKIEIEMVLIAMAKIYAKLCTVSRLVNRTYNIQILVTVGSRFVMITTQL
INTYNAIRDPSSEKAIHYLILAIYLSLHFLKIFMIASVSENTASKAKTTG
IYLHNLWELLPAFKNEVKYFSLQILHQNLTFTGCGFFVLDNTLIYSIVQG
KLS
>HeleGR16(a)
MIIPTLIKNLHVCQFTTYTFLLYLQFKNVNDSLVKLQNVGEMYKNRFCTK
IELIEIRKRHELLCRSGRLLNRGFSVQLLVLIAFDFVIFVMNVYFCYSKI
VDERFNRDADLIDATMLGLISGHVFMRIVALTTVCFITQRQAKLSVALCF
KIDHLYEKISNEVDLFVLQTEHWNNKFTAMNFFNIDMTLIYSIIRSATMF
LLIVIQLDGTN
>HeleGR17(a)
MEKYKHGNNSIDNGPTNIQYLAKNRRIVMIFGAKPKPPRSRTFSYWKAIV
DGLNEPKNLYDTLIPLYLFTKCVGLSPFTFEITDGKSSFRPSLLGSSYSI
TITLMFVGYYIYAVGQREDGNDSNKVARSIDMYHLYCTITVMSWCIVLNN
YHQKTLIRAVKRLDEADANMNCYGSKIDWKMTRNYTGAYFLIITTVLATC
EYMNCTMFLRQVATLSTYCLLMCYIPMVVNGFTEAQFVSYILLLKQRFAI
LNDELRSLALRRKYIPSIKITKIGPVKNENRSEPIAKSLANARLMHSHLC
EIGATFNRSYSLQILLNIGDVFIGFTTLAYYCFDGCMKLYLNEEGSNLYN
TITTGVWTTIKLSRLLVLTLSCSAVKNEARLAGQIIYQIDNRYEEEVSSE
IYAFGKQIIHWDFKFTASNFFDVDMSLFCAAVSSATTYLMILLQLDIANK
QIEKTIANNRIE

>HeleGR18(a)
MTEIDSTAAKVKCCLINDRKTICRNTCLLLVFMFLSIVLRWSLILLTIKT
DIIGQLLIFLAIFMMKFPKYQFAIVLLEIRIRFAAINDKMKNSIAELAPR
DFIEKLNALCRLHCKLCHASGLVNAVFGVQLLFSIAVSSATILLQAYYFY
VCLSRAYVSPNALVLTRSTVCMLQEFTEVYLVIYISNKICGVANKMSVIL
HGLRSNIATNAEIERQIEMHSIELLHQTLTFSAMGFFDIDHSLTFSMVSN
IITYLVIAIQFDQNRVFGNARNATLTL
>HeleGR19(a)
MFDISRRTDMKYIRPLFALASFWAIIPPFDFNSYKIKYGFNYKIFRTVII
IYLIAGFLYAFVEKALYIQPLLHNTVVVVDYLAFISYFLINILTLLSTNF
YNFDIFEKMLSILVGIDRTLMKYHRSNATNTKTYIKTELLVCFTTLFCAI
VFDSILWNRATITPVIQFSTLYENAQKIQTHVMMHVVCNFISCIRYRYRD
ANNLLVNIVMKKTTKKISGKLAIQKFKEEYTVKNVINIYIGLNKMIDCIN
RLYGWVLLCLNINLVSTLLLSFDFIIEYTSDANILKENNGNWFIFLMLIW
SAFSLILGVLLANLTHSTSLEIQNTSHLSYKLLQHVPYDTTANPFLQEMR
EDLMLLSEQMSSRTPAFSAAGFFNIDYTMLFTLLSSITSYLVVLIQFN
>HeleGR20(a)
MQHIKLSIKTQTENLNGMLEMCAEDVVNDETFLSVNDVHKLKTMYLILLR
QIQDFNDVFGRLIFLYFLYTVTGYLVCTCTITVSKYSMKQLGVGFFATSS
VNLALMLWMISIIAITCGGTTKAARETGPLCYVLCSKLRRSRNVALYDEL
LTFARVASMSNPVISAAGFFTVDYAMLSNVFTILISYIIVILQLYR
>HeleGR21(a)
MNTEVLYYSDEISAKGTVSDFKHSARLKDINKRSMLSSANGNILDQHDQF
YRDHKLLLKLFQVLGVMPIQRGKIGRITFSWLSAPTMYAYCFYVVTTILV
VLVGYERLIILTKKSKKFDEYIYSVIFVVFLVPHFWIPYVGWGVAKEVCK
YKNSWTHFQLDYYKITGKSLVFPHLNTLIVIISSGCLILAVAFLLTLSVL
MEGFTLYHTTAYLHIVTMINMNCALWYINCRAIGNASKSVADSFEEDIQE
CCRSYIIKHYRILWLSLSEILQKLGNAYARTYSTYSLFMITNITIAIYGF
TSEIVDHGFRFTFKEMGLLVDAGYCMALLYVFCDCSHNSSINIASRVQTA
LLSIELSSIDQHAAREVELFLIAIQMNPPKVQLQGYTIVNRELVTSSIST
MAIYLIVLLQFKISLVKESKLS
>HeleGR22(a)
MSSGFVVKPSRKTNIRYITPLFRLCTILCIIPPYDFRNYKFSISFAYKVY
RLLVMTFLLVGFVMCFMSKVYNTFHLIQKTVVVVNVLAYVSFFLINIIAL
SSTNFNNFKSLEKFLNELVSIDRKLVAYHVANKTKTRTFLWVELFLNHCV
LIFVMLFDAVLWYLATDNQFHFYMLYENMQKYQTHVLVHLMVNLIMCLKQ
RYKIANDLLAEAVRQRDVVDVSSSKIFFKPYKDIYTVRNVIKIFIGLNRM
VEYFNKIYGWTLLCIHINIIASLLVSLDFIIEFSSETNTLRDQYGLEFIL
LMSMWSLMALCLGILLASIANSTINEAQNTSNVSYKLIQYIPPSSTNVLD
RELREDLLLLSEQSSLRTPCFTAAGFFNVDYTMLFTLLSSITSYLVVLIQ
FSS

ORs sequences

>HparOrco
MMQFKPQGLVADLMPNIKLMKFAGHFMLNYYAENSGAVHTLRLGFCFGHLFLMLLQFGFT
FGNLVQESDD

VNDLAANTITILFFTHCIVKFIYFGVRQKLFYRTLGIWNQSNSHPLFLESNNRYHQLALTKM
RRLLIIVM
VGTIGSWIAWTTITFFGDSVHTRKDPSNENETITEEIPRLLVRSWYPWDAMSGIPYYITLVYQ
VYYVGFS
MLHSNLLDSLFCSWLIFACEQLQHLKEIMKPLMELSATLDTYVPKSADLFRAPSASSQDRL
MDSDYNARN
EDVHMKTMYSTHHEMGVTYRSGQLQDFSGGIGPNGLTKKQELMVRSAIKYWVERHKHV
VRLVTAIGDAYG
IALLLHMLTSTITLTLLAYQATKIDGVNKYALTVLGYLFYALAQVFHFCIFGNRLIEESSSVME
AAYSCH
WYDGSEEAKTFVQIVCQQCQKAMSISGAKFFTISLDLFASVLGATVTYFMVLVQLK
>McarOrco
MLKFKVVGLVADLMPNIRLIQASGHFMFNYHADNSGALHTLRLGYSCMNLVFVLLQYGAI
FGNLVAEKDDVNDLAANTITVLFFTHCVTKFVYFAVRSKLFYRTLGIWNQANSHPLFVESN
NRYHALALKKMRILLICVGMTTILSAAAWTGITFVGESVHTIKDPNNENETITEEIPRLLIKS
WYPWDAMSGMAYYASLVFQVYYVFFSLSQSNLLDSLFCSWLIFACEQLQHLKEIMKPLME
LSASLDTYVPKSADLFRAPSATSQDNLIENEYNAKNEELNLKGIYNTRQELGGHFRSGTLQT
FGQGGGGVGPNGLTKKQELMVRSAIKYWVERHKHVVRLVTAIGDAYGVALLLHMLTATV
MLTLLAYQATKINGVNTYAASVIGYLVYSLAQVFHFCIFGNRLIEESSSVMEAAYSCHWYD
GSEEAKTFVQIVCQQCQKAMSISGAKFFTISLDLFASVLGAVVTYFMVLVQLK
>DponOrco
MINKFKVVGLVADLMPNIRLIQASGHFMFNYYADNSGSLHILRLGYCCMHLFFVLVQYGCI
FGNLVKEKDNVSHLAANTITILFFTHCLSKFIYFAARSKLFYRTLGIWNQANSHPIFLESSNR
YHALALKKMRSLLYIILFGTIFSASAWTAITFVGESVHFIKDPDNDNETITEEIPRLLIKSWYPF
DAMSGMTYYVALVFQIYYVFFSLFQANLLDNLFCSWLIFACEQLQHLKEIMKPLMELSATL
DTFVPKSADLFKSPGSATSQDHLIENDFNAKNDDLKGVYSTRQELGNLNFRSGALQTFGQG
GGGVGPNGLTKKQELMVRSAIKYWVERHKHVVRLVTAIGDAYGVALLLHMLTATVMLTLL
AYEATKIDGLNTYAATTLGYLLYSLAQVFHFCIFGNRLIEESSSVMEAAYSCHWYDGSEEAK
TFVQIVCQQCQKSLFISGAKFFTISLDLFASVLGATVTYFMVLVQLK
>TcasOrco
MMKFKVTGLVADLMPNIRLIQASGHFMLNYHADNSGALHTLRLGYCCMHLVFVLVQYGC
NFVNLVLERGDVNDLAANTITVLFFTHCVTKFVYFAVRSKLFYRTLGIWNQPNSHPLFVESN
NRYHGIALKKMRRLLYIIIIWTSFSAIAWTGITFVGDSVHNIKDPENENLTITEPIPRLLVKAW
YPWDAMSGMPYYITLVFQIYYVFFSLAHANLLDSLFCSWLIFACEQLQHLKEIMKPLMELS
ATLDTYVPKSADLFRAPSATSQDQLIENDYNEKNEDLKGVYSTRQELGGHFRGGALQNFGS
GGVGPNGLTKKQELMVRSAIKYWVERHKHVVRLVTAIGDAYGVALLLHMLTSTIMLTLLA
YQATKITGVDKYAATVLGYLLFALAQVFHFCIFGNRLIEESSSVMEAAYSCHWYDGSEEAK
TFVQIVCQQCQKAMSISGAKFFTISLDLFASVLGAVVTYFMVLVQLK
>ItypOrco
MMNKFKVAGLVADLMPNIRLIQASGHFMFNYYADDSGSLHILRLAYSCMHLFLVLLQYGCI
FGNLVREKD
NVNYLAANTITILFFTHCLTKFIYFAVQSKLFYRTLGIWNQSNSHPIFLESNNRYHALALKKM
RNLLYII
IVGTIISACAWTAITFVEDSVHEIPDPDNENSTIVEAIPRLLIKSWYPFNAMSGMTYYIALVYQ
IYYVFF
SMFHSNLLDSLFCSWLIFACEQLQHLKEILKPLMELSASLDTYVPKSADLFKSPGSATSQDN
LVDNDFNA

KSDDLKGVYSTRQELGNLHFRSGALQTFGQGGGGVGPNGLTKKQELLVRSAIKYWVERHK
HVVRLVTAIG
DAYGVALLLHMLTATIMLTLLAYQATKINGINPYAATTLGYLIYSLAQVFHFCIFGNRLIEESS
SVMEAA
YSCHWYDGSEEAKTFVQIVCQQCQKALSISGAKFFTISLDLFASVLGATVTYFMVLVQLK
>RferOrco
MNTFKVAGLVADLMPNIRLIQASGHFMLNYHADNSGALHGLRLGYCCMHLLFVLLQFGCI
FGNLVKEKDNVNDLAANTITILFFTHCLTKFVYFAVRSKLFYRTLGIWNQANSHPIFIES
NNRYHALALKKMRNLLYIIMIGTIFSASAWTGITFMGDSVHYIKDPNNENETISEEIPRL
LIKSWYPFDAMSGMPYYIALVFQVYYVLFSLLHANLLDSLFCSWLIFACEQLQHLKEIMK
PLMELSASLDTYVPKSADLFKAPNSASSQDNLIENEYNSKNDELNLKGVYSTRQELGNLT
FRSGALQTFGQGGGGVGPNGLTKKQELMVRSAIKYWVERHKHVVRLVTAIGDAYGVALLL
HMLTATIMLTLLAYEATKIDGVNVYAATTIGYLLYSLAQVFHFCIFGNRLIEESSSVMEA
AYSCHWYDGSEEAKTFVQIVCQQCQKALSISGAKFFTISLDLFASVLGAVVTYFMVLVQL
K
>ItypOR46
MNAFPDSDSLTALKFTSVLGLFPWKLAFQQNKFYQTLYYWYSLFVLLWDIGFVFTSYVELV
ILLRGEVLH
IDEICTNIRITTIYTCIIIRLVMIRTSSGLLKLIQEIIDSDKQVTIVDDEETTKLVKTETSINNLKFI
WY
VGICCSIGLQFFIRPLVAEPEIIQIGNITEVGPRDLIILTWFPFDEQKYYWVAYFLQVIDGIIGTLF
VAL
SDVFIVNLILYPTTQLKKLQHIFRNFEHYQQSYKRLNSCETEESAGIKVITHLVQRHQRIIKFV
DTFNGW
MGPLMVFDFLQSSIQIASILISDLRRDITFAMVCFIVTFFVGMVLRLYLHYYSANELILESEKL
ADAIWY
SNWYEQSPKIKYLMLIVIIRAQKSLKYNIGAFGIMSLESGIAILKATYTYITVFTSNN
>ItypOR49
MTMTIYPKTENMRLTAIYSSTLGIFPWKFLFQDRKFYQKLYRYYSIFILTWYIGFVVTAYIELF
VLLSGE
TIKMEEVCTNMCLTLVFTCAGLRACVMRYGNRLDDTIQSIIDTERNTNLLDDENVREYENK
YISVMRTFT
HCYAASVIIPDAQRSVFVAITSPQITEVGNVTVSYKPHIMSSWFPFDKQEHYWPAYWFQVFD
GSMGASFV
AFVDIFMFNLISYPVGQLTKLQHLIRNLKTYQDKAWQAGVQQADLSVLNDLVRRHQKIISY
VDVYNNYMG
TFAIFEFIQSSVQIASVLAQTSPDNLTLEEASFIVCFFISMTIRMFLYYFSANQVIVESRKVAISV
WESN
WYEQRPEIQKSLLLMMIRAQKPLCYRIGGFGIMSVESIIAIMKGTYTYITIIYRGY
>ItypOR29
MAAYPQCKNLRVAIIYSSIIGVFPWQFMFQHNHLRQTLYRWYSVFLHFWFSGFIITEYIELYL
QCTADEL
KLDEICANICVVMVFTSTAVRQLVMRFNKMVNDLIQSIIDKEKHNDFLEDDKTREIEDKFIK
SSDSISNW
YAAPVYITLFQYVLFPMMSKPDIIQIGNTTQALRPLIVDSWFPFDKMEYYWIVYVLQLFDLL
IGALYVTY

LHILMFNMYRYPVAQLKKLQHVLRNFGRYKVEYMRQSNCNEYISALVVFRECIKKHKKIIQ
YVDGINECM
STYTVFDFLQSSFQIAALLVQTSPNDMTFISFLTVFTFITTVMIRLFVYYHSGNELIFESVNISM
AIWES
NWHEQSPQIKSMMLLVMRRAQKPLCYTIGGFGVMSLQSVIAILKATYSYVSIIFRQ
>ItypOR28
MGLYPASRYFKNPIMWSSILGAFPWQMIFQENAKLQQVYRWYSNFMLTWYFGMVTTEYIQ
LYHILNANVI
QMDEVCENVCMSLVFTCTGLRVWVMRRTNGLSEIIQTVVDAEREADGLDDEKTRQYEDIH
VKHMEKVSFI
YAAFVFMSVTNGCLATLYADTKSVIIGNSTIVEKPLIISTWFPFDKNEHYWVAYGLQVFDGY
MAALTVAC
TDILMFNMISYPIGQLTKLQHLVRNMAVYKTHFEAFPTFTKIVQRHKHVIKYVELFNQSMGT
FAIFEFVQ
SSVQIASVLVQTSPDDLTLMSFCFIVLFFTSMLTRLFMYYYSANEVIIQSINLGDSVWESSWY
HQPHQLK
QAMLMVLVRAQKPVSYKIGGFGIMSMQSIVAILKATYTYISVILRN
>ItypOR27
MRVYPDIENFKITAIYSSTIGLFPWKFMFQDNQVLQQTYRYYSYFIYGSFVIFIITAYVELIIML
NGDVL
KMDAICSNICLTLAFTCSALRATVMRVGPNLLKIIEQVMHAEKNPASIEDQTSFNLERKSIKT
MRKLSHL
YAVAITMIASSKCALAPFEKGEIVHIGNTTIIDRPLIMSAWVPFNKNTHYWAAYIIQIYFAALG
AWHVAY
VDMFMFNMLGYPIGQLKKLHYYIKNITTLTRNDDSLEEFKNVIRQHQQIISYVKFYNDSMG
TFAIFEFLQ
SSVQIASIFIQTSPSDMNLGQFGFIGGFFIGMLFRLFLYYYTANEVMTESEKVGVSVWESDW
YEQPTNLK
MALLTVMMRGQRPLYYKIGGFGLMSVQSIVAILKATYTYLTVVVRNN
>ItypOR25
MKIYPDTKFFDVTAKFGAIVGLYPWQFMFPDNNTCRQIYRWYSYIVLLSFIVLLLPMYVELII
LLRNEET
SKDELGSNLSITIVFSSAGLRALFLRRGSNLINLIQNVMDEEKKQLFVDCKKVQLLEDKCLK
VVRKLSYI
YAVIVVVAASQKSVTALLQTPTSSTGTPSRDLIISAWFPFDKQEYYWQAYCIQIYHTIIGASYL
SYMDIF
MFNLLSYPIGQFKKLQFIIKNMEVQHYSYNDSENKSIDDGVRSIIERHQYIIQYVDFYNKSM
GTFALFDF
LQSSLQIATVLLQFSPTVGTIIFMLIFFALMLLRLFLYYYTANEVSVQSEKVKMAVWESKWY
EQPPKIKY
ALLRIMTRAAKPSKYIIGAFGGMSTYSIIQILKATYTYITIMFR
>ItypOR23
MAVYPKSEHLKVPAIYCSTIGIFPWKFMFQDNKNLQTIYRCYSIVMLAWCIGFVVTDYIQLVI
LLTSKTL
DMQEISFNTCITLLFTCIGLRAVIVYFSPNSANLIQSIIDSEKVTYLDDAECMKLEKEHLRSVR
LISHCY

FIFIIFSTTSRCVYFFSKEPDFIQNGNETEIVKEHMLSIWFPFNQEKYYLTVYNIELLDSFLGTF
FVAYV
DIYTFNMISYPKGQLKKLQHIMKHFHNYKAKYSSETNEENDFIVFKDLVQRHKQIIQHINAF
NELMEFVA
IFEFVQSSAQIACGLTQTSLENLTIGSFLFVMSFLISMLVRLFLYYYAANDVTVESTKLAQCIW
ESNWYE
ESQKIKLSMLMVIIRAQKPLIFKIGGFGTMSVQSIVTILKATYSYITLAYKRT
>ItypOR6
MLRKNPRYATDFFSVNQWMLRRAGLWRPSNKNKTVQFCYTLYAIFVFIFVNLWFTSTEFISL
FYTYKDKY
ALIKNVNFFLTHFMGAIKVVFWYFCGQYLMEIMQDLENPNNHYEGYKDYQPGIISQKYKK
QGSKYSLMFL
MLAHATLTSSYVFSTITTIQHMKGNSTVALPDRLPYYSWMPFSYDTGPKYLLAMAYQAGP
MFSYAYSIVG
MDSLFMNIMNCIAANVTIIQGAFKTIRERALPGQPNNVLHESKADMDVMRVELRKIVNHLQ
TIFKACDKL
ENVHRMVTLCQVTATLFILCTCLYLVSIAPPLSKQFLVEFVYMLAMSFQLYLYCWFGNEVTI
KFQELPRY
IWASSWLATDTQFKKALLFTIMRTKRPVFLTAGKFSRLILPTFMSILKTSYSIFALIRNTSK
>ItypOR5
MLNHDYPKNVLSSVDTILLICGLGKISKKIPWVIRLAYSIYNYLIMSIALIFLMFELIAFQMAL
DDLPTF
LSQIAMVLTHSAGFVKLWMLLYMMNPMEKIRNKLQDGRFKYVPVGNFQPGLKMRRAKVL
MSRVTVLIFTM
YSFVGVSAHISAAVDVIKNTRNGKFVKGITCHDILPFNFYIPFDISTPTMCHYALMYMNISLD
AQAFYIA
TFDLIFVCFLLFLSAQLDILSDAFTTIRKRCLKKLEMDSEKQCFYDDQCRELEEEMYREVTH
CNQHLNLL
IEVRNDIEHVFSLITLIQTSASLLICASCLFVAAQVQGNSPTFFSQLEYTAAILSQISLYCWFGD
KITIS
SSEIPMALYKSDWLSCSQRFKKSMLMAMTRMRKPLYVSIGKFTPLALNTLLAVLKGSFSYF
TLFQRAG
>ItypOR31
MHIFPKSDHLDFSFALCSMLGILPWKLVFQDNSFLQTLYYLYSKTLLIITVIFITTEWMEVCRI
LNXDPV
NLTDLNNAIAPVLLFTVTAIRMIIFNRNPDFMKLLNYIINRQEFMAQRDDEIRKPSQKFIKINK
WTGVGY
LIMYLAVIYQLLALPLVLGPIEQQTANQTTTIRILPLLSWIPFDTQQHYWGCYLWQALNLQL
ASCNICHI
DVLMFALILYPIEELGYIKHVLRNFDSFKSRTGIENSNFASITVFKDVIKIHNNVINYVGTVND
TLSFVM
LLDFLQSSLHIAVILGAVVVGGPTDLASLSFVGTHFFSMVLRPFLYYYYANQVMVLGGDLTK
EVWNVDWF
DESKDVKYMVRFFNMRAQKPLQYFVGSFEVMNLQSFISILRVAYSYVMLLHTLQ
>ItypOR16
MYNIPENERKNYFLKFSRVTMLMLGIWPVRRGGDLLEKLYESYFLTTFLYYIAFNLSGLALA
IRTWSNNY

LTTASSMGIVIEYMSNAYKVWLFKTSVFKSLIKEIQDREREIFEGPDEAFKEIYIRNAESNKK
VVLFYTI
MGTSGISLYFITPLVSNVLMPLGYNNVTGVYEHYFIVFNWFPFDPNRYYWAAYLIQFTGCLI
GYSYIVHC
GAFYISILNFIRTQLKILRHVIVNMSEYSLLYKNTYKLTEEQSQFVLLRAVVLEHQRIISFVTK
TNHTIQ
LFTLINFVISSFQLALLVYQIFQVAILQQVTVLSYFITLSTQLFLTYYAAHMILFESSNIASSIFE
GNWD
TYPPQTLKLLQMICMRAQKPLAMTIGPMAAVKVTALFQIFKALYSYICLIIKF
>ItypOR34
MKFRELIHNDFLGICIQLGYYFCIIPEKAVTTDKIESRNYFFYTCIVRTLILYCHICQWVKMYQ
IITADI
FIFDELVRNCAITSIHFQSFVKTSIFRQNYQLFENVIDFENVLYKNNDQKVLLIYRDTLQAIKN
SRLVYV
FGILIVIVFYIAAPLFRGPYYVEMGNETVTIIQLPLSAWSPTNNYFSNFAVTGAMGAYLAMVF
VQTDLLY
YCFLYFSICQLNILEHYIVHFHHYSNELVNDHKCSHVMALSLTQKIYIKYHQNIIKNVKQLN
DALKNSLL
IDLVPSSIQFANQFYIIATNLNIMQCVCIGFFTIMLMSRVMAYCYLANQISVQSQKIGSAWFQ
MDWSDFP
NEMKKMISFCIMRAQKPLVITLGNFGNITLMTFVGILQASYSYVMLFITL
>ItypOR2
MKVLQRETEITFFKFNIWVLKTCLLWPEDLNYKYDKKRFIKDLTMVASLMPCFLPILADFLQ
QLYEEVPD
LTEAVENMIALNCLIGMFYMVICFVRNRRMIIQLMIDIRTFNKYGNDSITQEVDNKANLFSK
MFMFYGIL
GNFVYMAMPQIRVSKCHLNRTEDMIEKGVPCGLVVRSYFPFKFDYSPVFEIVFVHQIYTCT
MVSVVVLVL
TMLFCGFLMHIVNQLKHLRVLIARLKNVPPEKFERKLIFVVRYHVAIIQYSQNTAGAFSTML
LFYITLTS
VVLSVLCFEILMVDAFADSVRFTLHLLGWLIILLSICYNAQLVLDQSQEVANDVYSLDWVSI
LSVDVQKK
SKXVIMRSQKALVMEAGGMGVVSLSAFLKVLSSAYSFFTLLLKFK
>ItypOR8
MSELFLLHFPRILMVICGVWRLPYFKSKKVQTVYDIFSIFLQFTFSLMCLSMFFELVNLINTW
NVLNLIE
FSRVALSSYLCIIKALVLRNSSIQRIMVYMIKEERNVLRSKHQQPKALYMDTVKLINRVSFLL
LLVVLPT
LLAFSADCLRKGIIDFDDAVKYVYLPLIDQKKYKTVQLTVQTIFINLIGFYYCMTQAFMVTA
MKFAQGQL
ELLQLYFREFDYYAARQSTTEIAYLKTLLEYHQKIIHFVETLNKEMRLVIIIEFFFSAVNIACSL
FTLLT
MATNLIDILFSVNCLVFLLAQLAILSYLGNEIYQAGLNIASASYELKWYEKNREFQKNLLLVI
KRSQKPL
VLSVGPLGPLTNETFVSVLKASYSYFNLMTRYN
>ItypOR17

MGLMTTPRFFLQLWGIWPVNTLLPPKYVMYRFCIIGWYSFFNIFQFIASIRLILNNESFERISR
CISVM
VTLVLMLVNSLIYQKNCIPQLCSTVMEIEQXLARSNDAKITQTYHTTVAKNKYLNLYIVGSS
LFTLVAFI
GLSLLDVIKAGPAFWDFDNVTFMHELYVPFNRGNHQALIITTNIFTACESVVVNGVIQTTFY
ALVMYGAL
RFKILQLNLKKIEQTEGDRKWRMRELIQDHQYCIRFVGELNQATKNVLLMSFVLNSLKVAS
VLFPLMAIR
EFTDLAFPLIYSSMLVSEVVFQGWMCNEITEQSLQVAQTIYDTFWYKESKQYNVLLQLMLM
RAQRPVTMR
IGPFGAMTTSTILTTMRAAYSYATLMMNSS
>ItypOR18
MYTLSKNKPFYYALVLLRAFFWYPSSPKCSVTFILCSLVLRLLSTLAALGTLAHLVLNLTGE
TKAEISED
IGDLTGFVGCMSACLNFLWHRSRWSSFINRLLTFKQFGTPPGYVKVVRRGNLITLACILYTIP
GMLWYSH
LTHLDIPRCEALNREFGMKEACGMVNPTWIPPGYDRRNGWRFWVLYVLQCTGIFVYLPSTF
VISNIPLEA
VGVIVTRIHLKYHLKRCGSDLGRLYHCVKYHQDIIEVSKELSDLVQATLGTLLLTGAVVIGS
LGSQVIK
ASTPKAVTFILGYVTTIFMVCHAGQKLINQSLTLADQVYWMEWYEQQPKIRKDLRFVLARC
QKPLGLVGP
PSMGXAGYSLFLIMLKTSYSYLTLLNEVIS
>ItypOR9
MFSSKNLYKYYSHFASTSIIMYTIMLTIRLVQLVIEGQTPSAKLYRCFTINIVIYMMTANLIIFR
RYGLP
DLISQVMKDEEAALNSLDKDIRKTYLAQTKIYEFTSVAQVVSTFASGLMFIALNVYMKVKG
LLKHEAFMY
ELWFPFNRENHDGFVIFFNLYIVVLIMFCNVASRIIPQTMIIYANAQLRVLQILLEKAFDAPCS
DPLVKI
QELVKKHQDLINFITFLNSALRNVIFMEYIINAINVAAGLLQFITVRAAMDLVYAFVHFSLLVI
QIFVLA
LNANNVSTQSEAIANAAYNSQWMDQSNNIKKIIYIMIMRAQKPLVLNIGAFGVMNAESALT
TMKAAYTYV
SIGLQR
>ItypOR13
MSNYQRYNIQTFLKEERLFLGITGFVSGNLKLHLPTGVTYVLTTMQVLASIYYGLSTTDLAE
ITAAFMIT
LSHINTLNKLFGLHLKSLTQLDRILVKQIFALADDRELTILKRTLTACHNMLTMYLVTVLGSIL
LYGATP
LVANYTTMERNYPTLAKFPFNPDDYYWAVFAGEFFIVALSALSNGCMDRLFAKHVAIATGLL
KILRHKIK
QIMDVDDQKVIEAKMKHCVLYYNEVMGYANQIENQFSFGIFIQFLCSCIVICLIEFQVLLATS
SETIGLL
LTYLTCMITQVTIYCWYGHQLMEESNSISMEFYDLNWIEMSVKNRKTMLTSKERAKYPIVL
KASGVFPLN
LATLMKILRTSYSYFAVLHQVYTK

>ItypOR4
MDKQSQILKFHVKVLKFLMIWPFDGLDPNQNYYLMRGCFAYACFCSIPVFSGAAFQFCVGI
DNVKVLLEV
LVGVGNITGYNIAYVCFLKNQEKIQFLIRDFQEFVQFSGPEIIQNTEEKTTRYTKYLLGYASIG
LVITFS
WQMLSTESCVAQRGGDYYVRHDPCWLPVRNWYPFDASQPKLFWIVFPIEAIYSIHICLFFSL
ATSTIIGF
LMQITSQLQYCSNRFEHVFDEVDLKPFQQIKPDFLFLIKYHKKILDYSKKLFNVFDALIVVYI
SLTSFIM
AIICYQIVDPKISAQDRIKYAILLIAWCLLVYLICYYGQKVQDEALKIGQSIFKSHWYGGTTA
VELKPYI
LFTLARTQIPLEFKAQLFGTISLLQFMKVMKWSYSGLTLLLAVTDED
>ItypOR19
MLIFGNIISANVIVLFGSSLINGDYFLVTSSFPFALAIIVVNGSTLSFAVNHKQWSNLFKSLTDC
QKFGK
PPNYDLLKKNGDRKGMICMTCYISTCTLFAIIEAVEEQRCLKNVSKHEICGFIIPVWWPTNY
HPSTFLKT
MVQVYEVGTIIILSNFTIIATLQYQVCEYIAAKAAHLGLNFNAIDPNSDAKTQFEQFKLFVDY
HQHIISL
CAEFDSSCKRTVGHVTFTTAAISALFSYHGMQGNYKLLAFLALYILNLGFMCHTGQNLEDA
MLGISNSIY
SSKWYELNIRVRQRIPFLLARTQKRIGLDAVPIGYLNYALFMTVLKTTCTYLNLLNHTI
>ItypOR43
MDAALALMKNITFVAVVVFKTVVVQSDAIVKLVKAASVEEEKIRNLTDQAIRKIYKSNVDY
CNRVTKVII
TYLYGSGTIYVLDGLYKSYTYYENHPNVKPEDPKPHTVLFWFPFDHNRYYKIAIAYESFHIF
QTLNYNGV
AQSVVSSVMVFLKIELKVLQHHIRAIQGGSRDYQKVLIKCAIKHQQIIQWVNDFNNNFRFIIL
FEYSMIS
LTLATILIDILQGTKICFNATFFALNFTQLFVLAWNANQISDESSISISDALYACSWYEFDKTTQ
DFVLF
MTLRCKKPLNISNGPFGYINMDAALSRVKLAYTVVSVLSTSTK
>ItypOR36
MKIPHMFEHAMKHERNIFKSRDLELIICYQEQVKYGRRVNLSQFLVTTLSTSTFAVTALLDV
YWAADMSK
YEKEPFMHDLWFPFRRETHMNWVIFFNLFMIVQGTCFNTATQATLINLMIYSSSRLKLLGLK
LRKFDAIA
SQNGRDILETVHDLIFEHQDLLSFVESLNVRIKYVLLMEFILNELGLASGIIQLIVIDTTSYMV
SVVTII
ILQLFQIFVIAWTANEITIQGAKIADSVMASNWVEQPTNIKKLFLIMVMRAQRPLGLTAGPFF
NMNANTA
VSTVKAAYTYLTFMMNNYN
>ItypOR1
MWITMFCTKNRHVLISLVQGLSDFTGFPPNFDKFMQQLNFYSKIHLCYLTGGSLMYFVLFA
PLHKRNCDE
LKREKNLTETCSLLLPLNAPFVEYQSFGKFPTLQLLNIIIFLSLMYMYMCAGTIVWLNVELV
EHIRIRIR

HLKHMILRALKSNDKQFRREKFRKAVRYHEYICSMSRLADEFFGTELFLHVVLTGAILGISA
YLIGDGSL
ETVMIFVGWLNAIIMGSVAGQRLINESLGISDIIYEVDWYNFETALKKDILFFLVYAARNLCL
LGLGMW
>ItypOR3
MPTRNLYPFDASQGWSFWILFVIEAIFCYHTCCVFTLATVTLIGFLKHILAQLRYCGHEFETIF
DGVNEE
SGGKHLTLQHFIRVVKYHQEILRYTEKVFSTFNVMIVVYTGVTSFILAITGFQITSPETGGED
KIRYTML
IIGWALLFYWICYYGQQIQDEASQIADAIYNSKWYENTNTVVLVRRDIIIIYLRTKRVLDFKV
QFLGAVN
MEVFVAVMRRAYQIFTLLLSVT
>ItypOR39
MHDLWFPFRRETHMNWVIFSNLFMIVQGTCFNTATQATLINLMIYSSSRLKLLGLKLRKFD
AIASQNGRX
ILETVHDLIFEHQDLLRFVESLNVRIKYVLLMEFILNELGLASGIIQLIVIDTTSYMVSVVTIIIL
QLFQ
IFVIAWTANEITIQGSKIADSVMASNWVEQPTNIKKLFLIMVMRAQRPLGLTAGPFFNMNAN
TAVSTVKA
AYTYLTFMMNNYN
>DponOR1
MCLINFLSYPRKILLIGATWPKYHSSLGYKIRRTVTLANILSLLLCLSYNASFHMGDFVQFSE
SLYMLISVVNAFLKIVLLTLNGKVFLKLIGMLETPSFKRFETLYKKIVKDFLKTVKAVEFFY
WIEVSGTVLFLSLFPIFESEALPMDFPHFNNGTFHYPFYIFEAFSLFVSAYDNMAVDLLTVGII
SIAAVQLRILNQKLVDTDKNIKNMPNYSVGNVERLTIGYLTECCIHYSDIEKYVKSILEVFSVI
IFVQLGTSVVAICNAGMMILSVKLASIEALSLFFYIVTMFAQLGMYCWFGNFVYVESLEVTT
SCYLSHWDERRSAVRKTLFMLMERAKKPLAIRAVKFATLSFDTFIAIIKWSYSYFALLNNSM
KN
>DponOR2
MNLSKFIEFPRKTLVLTGSWPQQHSSWPYLSRRIIVMTSIALLLLALVYNASFHFDDPIKLSES
LFILISVVNVFLKLIIMVVNEKVFLNLIARLETSTFIKGGILYQPIYVKFMEIVRPVYVTYFILV
CGCVTFRSSFPAFDNKKFPLDFPHFHDGYPHYVFYFFQVFSLSSVAYDNMAIDLLVVGVVSI
AAVQLQVLNSKLRDTKQNVQFLPNYSISNHETLTVGYLKDCCIHYSDIEEYIKCLLDMFSIII
LVQLGSSIVVICSSGMVLLSLKPLSIEAISLYFYLITMFTELGMYCWFGNFVYVESLEVINSCY
LSHWEERGPAVRKTLFMLMERAKRPLEIKAVRFFTLSFDTFIVILKWSYSYFALLRNWMAD
>DponOR3
MSVLGETTQIKKKWRSSIAITEKVLVITEIWPNDDTSLYRTMKVVFITIVCIVFNLTVIDELKM
LAIRQDYKTLSMHLSTFGLYIGFSVKIILFQFTKHGPLKNMLDSMDSPIFHAYPPEMQKHQD
NCIRVSNLIGKFFVYLVGGTILFYLNKPFYSSYPLPITFSHPLTTTTFYLLLTLQCVCSFYLIMI
GICFDMLVMGLANVATAQLDMLIEEITTFTPTSIETLEKEEHRFIKRCAERHNAIISYVNSIED
VFTYIFLAQCVVSVTCICNGLFQLTHVAPVFSIHFYYNCIFTFNVLFEIGICCWFATLMTNKG
NDVADACYNYNWLHSSSATRKLLLIMLCRSQKPLFITVGKIIQLSIGSFLSVLKTAYSYYALM
QHLYDKTSQ
>DponOR4
MNFEELFKVPKLILNSFGLWPQTNRSLPAKIRRFLSIFLGVTMSILLLFECYILNNIDSTFELIT
VSIPPISTLLKQLAFCLNGDHFLAELIFLREPALTSVPRHLENQMKRHLNLAKYFGDRYLLM
CLASAVGYNLLPLITDKPLPIPFTLLHASLYQPGIYLLEICYLLNGTANNCCLDYLALNCMCV

IRGQICVLNGKLREARVICKEEKDLNVELVRYISACVEQHAKIIELVELTEKVFSEIVLIQYLTS
IAAICIIGFRMVTVDLVSFPFLLMAAFLLCMFCQLGIYCWFGNEIILQSLLTREACYEFDWIN
ADLQVKNMLLIIMERSKRPLHLTAGKFSILSLDSFTSVINSAYTFFAVIQTKYSQSSSHNL
>DponOR5
MDFIQLFAPFKLILNISGFWPQKNPKPIVKVRKVFTLLVNLFFCLSLSIQCLFLRSQIEEFLDVL
TVITPPVAYLFKQIVFFGHSSAFLTLMDFLKDDDLVSIPLHLQKQISDSLQVAKIIGVGYQACC
TMTILFIVIWPMFTEHQLPVQFTLFDLGDFYAFMYLLQIFALANAAANSSSLDLIALTLMCIV
KGQICVLNDKIRSLGEINASKGHGAQRVKYVSGCVLHHTKIIELVALIENVYSQIVLIEYLTS
MVVICNIGFQLVIVELASFAFLLMLTFLVTMLCQLGMYCWFGNEIMLHSAAIRDACYESDW
IHSCPQVRKMLLMIMERSKRPLYLTAGKFSILSLNSFTSVIHSAYSFFALMQRMYGKSTTF
>DponOR7
MANLKQALQIYDICSFLEGEHIRLGIGGFYPRRIKRTFIVNLVTVFAYIITIAQMAVVMNFVLS
ITDIVTITEVLLFSMTQVGFVNKLVNFHRNSRKVATLDELISQDIFTRVTVAEMDIMKTSFQR
CQKVLNIFLVSCFGVTLLYGVVPAVNGIMTGTKMYPFPGKFPFNPDDYFVLIYGGEVATVAV
SAWNNGAMDCLFTKHTVIATTLFRILRKKIKDLHYNTNEGERPLENRIKHCVRYYNEIIKYV
SAIENIFAYGILVQFMCSAIVICLTGFQLLVVASESGQSGLLVVVYLFCMMFQLVLYCWYGHM
LMEESNRITEACYAINWHEMKIGQQKMLITIMERAKKPIALKALGIFRLNLSTLMTILRSSYS
YFAVLQQIYRK
>DponOR8
MIKQPSYEVYATDFFSVNRWILKCAGLWPPSTPNRVVRRLYQLYTIGVFLFVNLWFTGTEFV
SLFYTYKSQYELIKNVNFFLTHFMGAVKVILWYFYGHLLRDIMNALESPQLHYEGYADFSP
HRISHLHRAIGRRYSLLFLCLAHATLISSYIPPLIAVAEYLNQPQGGLQKLPSRLPYFCWMPFS
YDTPGKYLLAVAYQAGPMFSYAYSVVGMDALFMNILNCIAENMVLIQGAFKTVRERSTLH
YCVGALAPPCHAIREHPLVLRQMDLETKKIIKHLQITLRACKHLEGIYHIITLSQVTATLFILC
TSLYLISTASPFSKQFFAELVYMMAMLFELFLYCWFGNEVTLKYEQLPMHIWESQWLATDD
CFKKQMIFTMLRTNRPVYFTAGKLARLTLPTFMSILKTSYSIFALIKNFSK
>DponOR9
MDTLGPALTKVRLRLPSLDIPDSKGTDYPKNLYSAIDRISFLCGQAKLSKNRSWILRFAYSLY
SYTLIIIAIMFIISEIITFRKSLTELTTVLSEIGMMFTHLVGMVKFWILIHKRDEIEQVKNKLRDV
QFEYVGIDDFQPGLKMRKEKLFIIIISTFIFALYNFVGISAHISAASMMYKYTANGNFLGNTTC
ETFVPYYFYYPFDVSSPSSCHYLLFYMDLSLDIYASYIATFDSVFVILLNLLATQLNILGDALR
TIRKRCVKRLQMKVDSSSLYDADNPLLENEMYNELTHCTKHLYLLLEVGNDIESIFTFLTLL
QTIASLLIFASCLFVAARVKPTTPIFYSQLEYFSAVLSQLTVYCWFGNEITLASSAIPYSIYSSD
WFSSSERFKKSMLLTMARLQRPLYVSIGKFTPLALTTLLSVIKGSFSYFTLFQSAGTD
>DponOR13
MYNRNQRFFVARICKLAGLYPVQLLPADENLRKLYTVYYHVLVVLFFICLMSFFAELFRLLH
AETVIVDDILKSFSLATPLVVTSFRQGIIRRSPDVRKMLKNAGIVEQRVYEENDPEVVKLFEK
AGQVSMLYYLYYVVATAFLSLISILEPMYDKHRVFSGNATVSPGKTPLPLWFPYDVQAHYW
ETFFLTILLVWAVVLFQVGMDVLFFYFIRSPVIQLEILHHYLKHFNYYTARISVEPGNVASNIM
MKKCIEMHQKVIKFVDIFNENFSNIMVLDFVQSSLRLASICVVITMTESVTVSSFGFTLIYLW
ISIIREYYIYHSGNEIIFLSSELVHSVYETDWHEENRQFKYMVAMFMVRAGKPLNIKIGPFGS
LGLPAFLSILRASFSYFSLVTGTQQL
>DponOR14
MYNRNQRFFVARICKLAGLYPVQLLPEDENLRKLYTIYYHVLVVLFFICLMSFFAAVFRLLH
AETVIVDEILKSFSLATPLVLTSFRQGIIRRSPDVRKMLKNAGIVEQRVYEENDPEVVRLFEK
AGQVSLLYYLYYAVSIVFLGLVCMLEPMLDNEQVFSGNATTSFRKMAIPLWFPFDMQAHYR
ETIFLTILVFCILAGFQVSMDVLFFYFMRSPAIQLEILHHYLKRFDYYTGRMSVEPGNVASNI

MMRKCIDMHRKVIKFVDIFNENFRNIMVLDFVQSSVRIASLSAVIIMNESDSVISFGFSLIHF
WMALVREYYIYYAGNEITFLSSELVFSVYETDWYKENRQFKFMVKMFMVRAGKPLNINIG
PFGNLGFPAFLSILQGSFSYFSLVTGIQKS
>DponOR15
MFIRNQRAFVARTCKLAGLYPVQLLPEDENLRKLYTIYYQALIMLYFICLISFCTELFHLLRA
EKATVDDILKSISMTTLFAMTALRQWVIRSSPDVQKILRKAGNVEQRVYEENDPEVVNIFER
AGHVALLYYIYYAVGTVFLCLGCILEPLYDNQKVFSGNATAFSRKLPLPLWFPYDIQAHYWE
TFCVTILLICLLVVFQVAVDVLFFYFIRSPVIQLEILHHFFKRFNDYTGRISVEPGNVASNVMM
RKCIDMHRKVIKFVDIFNENFSNIIVLDFVQSSFRLASISAAIIMTESFTVTSFVFTLIFLWITLV
REYYIYHAGNEIIFLSSGLVHSVYETDWYIENRQFKYMVKMFMVRAGKPLDIKIGRFGSLG
FPALLSILQASYSYVTLVRGIQKS
>DponOR16
MAIFPNCKFLNLVMKCATLLGLFPHQFLFKKHYYLQQGYSIFSKITFVVFTLILLSTYMQLLV
IVCAKEIDVTELSKNLIITPLFTITIIRQSIMYKSGFKRLLEHILEREQWMNRYANDNQLNFIEE
RSANELNRNIKIYLGMMIITELSYTIRPLLDPATETLIYNNTVLVKSLPLSIWLPFNKVDHFIA
AYVCHIIYTIFGSSYDTFGEFVLISVLVYPTTQLKGLKHVLKNMEFYQEKILQHYTHISKEDA
AIVVLRQSIQQHKIIVQYVSDFNELMGTCLFLDFIQSSLHMACVLAEVLTEDVTSMQLVSVI
AYLVILNFRLFLYYYYANELTVLNQELAVAIYQSKWYQYSKKVGFMTFMIIMRNQKGLKYK
LGLFGYMSLNKFISILNAAYSYVMLMYSVK
>DponOR17
MNSKVNQAEKLDPVDKRSKVFPSSDFLRLYAIFTGQMGIFPWQLMFERNKTYQNLYNLYSK
LILSYYMYVTVSMWLALVFLCLEDTLRIPEITKNITVSVICTVTIIRLFVMKLHPAFLRNITFII
DAEQYILSSNDAEVHRIYKNCKIISNRHTIFFIVLSYLMALFISLRPFFTDAYEINYKNESLQIT
SLPLSIWVPLNEQEHFLSVYFWNVLNNLVMTSIVLSIDIITFLLLIYPVGQLQILHHILSKFEN
YKNRMKLNYPGLDDDTIGAITLKACIDLHRNIIAYVDDLNACMNIFMVVDFAQSSLLLTSVF
AQLLWVEPSITFYGFVFMYVTYLNQRLFMNYYYSNEVWLLSENLNLSVWKSNWYEQSHY
VKFMIYFFIMRTRKSLKFKIGPFGFMNLSTYIAILKASYSYIALLHSTQK
>DponOR19
MAIYPKCRLIQISMISSSLVGTFPWQFMFQDNKILKNMYAMYSKLMLGYFTLFVFSQQLELL
ILITDEEVMRNAIFANISVTPIYTITLAKQLIMMLNSSFRATIKQIIDTEKCKSPIEDDEVFEIEL
RIVQRSNKLVKYYGLMMFVLGTLYCVKPILMTPNIVSSGNTTKAIGFFPLSSWFPFDEQKHY
PYAYMWQTLSLLQGTMYVTTTDILMFNLIVFTAVQLRKLKHLLKNFVHYKERFMTLYNIVD
DEQAAKITLIYFIRRHKEIIEYVRLFNESMEIVMVFDFLQSSLHIASVLPEVLMSEISLMVVLT
VASFLGSMLFRLSLYYYHANNVIILSAELSYSIYESNWFDQTPKVKQMILIFMLRTQEPLTLRI
GGFAVMSIESLIAILKATYSYVMLMI
>DponOR20
MAIYPKCRLIQISMICSSLVGTFPWQFMFQDNKTFKNMYALFSKLMLGYFTLVVFTEQLELL
ILFTDADVMTNAIFTNLSVTFLYTITLAKQLIMMLNSSFRVIIKQIIETENCNSPIEDDEVIKIEL
KIVRRSDKIVKYYGSLLVVLSMLYLVKPMLMTPIIVSIGNTTTVMRYLPISSWLPFDKQEHYP
YAYIWQVLNVLQGATYVTSTDILMFNLIVFPAVQLRKLQHLLKNFAHYKEKVKTLYNIADD
EQVAKITLVYFISRHKEIIRYVRLFNESMEVVIVFDFLQSSVQIASILTEVSMSEFSVMVVVTL
ASFFASMIFRLILYYYHANNIIILSAELSYSMYESNWLDQAPKVKQMILIFMLRTQESLTLRIG
GLGVMSIQSMIAILKATYSYMMLMI
>DponOR21
MAIYPKCRLIQISMISSSLLGTFPWQFLFQDNKTFKNMYAMYSKLMLGHFTLFLFTAQLQL
WILITDEELMRNAIFANLSVTFIYNITLAKQLIIMLNSNFRATIKQIIETENCKSPIEDDEVTNEI
EFKMVQRSDKIVKCYGFLLIVLTILFFVKPFLMTPTIVSIGNTTKVIRDLPISSWLPFDEQEHY

SYAYIWQVLNALQGSTYVASTDILMFNLIVFPAVQLRKLQHLLKNFAHYKEKVKTLYNIAD
DEQAAKITLVYFISRHMEIIQYVRRFNESMEIVMMFDFLQSSLHIASILPEVLMSEFSVMVVL
MVASFLVSMIFRLILYYYHANNVMILSAELSYSMYESNWFDQTPKVQQMILIFMLRAQEPLT
LRFGGFGVMSIESMIAILKATYSYVMLMI
>DponOR22
MAIYPQCEYLKIAMICCSLLGIFPWQFMFEGQKRYQSAYAMYSKVMLGYYIFFLISAYIKLFI
LLNTDDELQMDAISANICITFIYSITITRQLIMKNSTGFRSLIKHIIDTENSVSAIQDSEIDKIRE
NAVKESKRKSKWYLYILCVVTLQYILRPAMVDSKMIKVGNMTKIIKPLPLSSWFPFDEQEFY
KVAYVWQVLDSLVGASFVTYTDILMFNLIVFPIGQIKKLHHCLKNFEIYKENHKILYNITDDE
KAGEATFIEFVFRHRQIIDYVTYFNDAMGASMVFDFLQSSFQIAAILTQVLGNQITWVMFVF
VSTFFVSMVFRLILYYYHANDVMYLSQRLSYSIWEANWFEQPNRLKRMILMFILRTQKPLT
YRIGIFSVMTLQSCIYILRATYSYITLMVGYN
>DponOR23
MLLPDNQLFQIPMYLSVLVGIWPIILTESKWLKKAYRLFATMLYYYYLEYICRAYFQLVLLV
RAEHLNLEEILGNLCITLIYTVSMIRLRAFDTQQIKTLFSTIVDAEKLILQDPDPEVRKIYLAV
VRRNKVSHFVFFICGWMVSFLYFLHPFFMELPTMKVNNETITLKTLPLSTWWPIDVQKHY
WTAYCWNVFDGTLGSSFVIDSDMLAFSLVVFAVCRLDILSYRLQRLQLAGGQTSQQVNWEI
FRNLIVQHQEIIAYVDTFNDGMKYVMLFDFLQCSVQLATITLQLLVMKINFQNVLFVGVFLI
TMVMRLVIYYFNGNEIIYKSQQLAIDIWMTDWYKQSHNVKHMLLFFSIRAQRPLKFLIGPF
GVLSLETFITIIKATYSYMMLFVNTSK
>DponOR24
MKNDFFGFCIPLAKYIYILPDITPQDRTKPLLHKICAVIIYVLALFCYFSEVVKLYQIVTGEYF
VYDELIRNYTVSYFHFTSLIKAINIKGAISARAFKTIIGFEDNIYNGEDEDIRKVYKASVTPIQ
RVRKYYMAGMVMVVICYACAPAFRDPIEIQRENETIRIRQLPVSAWSPVEEYFWLDFVWKS
LVGAYLAYFFVTTDLILYSFIAFGACQVRILQHYIHNFNRYCEEIMHTEGVPKNESARLLQKQ
LIAMHQDVISYVNMINGSIKQLMMLEFIPGSVQLAGMLYQLMTNLNAIQCIFLGQFISCLIA
RIFIYTNSANDLSQLSQQLAADWFEIDWIELPKDIKMNLNICILRCQKNLCITVGDLNAIDMT
TFLTILKGSYSFLTLLTTI
>DponOR25
MGNDFFGVCIPLAKAFCIMPDLAPSTSRKSLLDKIYAFLIYFLVLFCHITELFKLYQIVTAEYFI
LDEFIRNYTVTSFHFTTIFKIVFIKGEISKRAFREIMEFEDAIYKSLNDDIHQLYKACIIPVQNS
RKYYLAGFVLVVAFYLAAPLFRDPIQIQKDNESIRIRQVPLSSWSPVEEHYWYAFVWTGLTG
SYLTIFFVTTDLICFSFIAFGTCRIEILRHYIRNFNQYCRNIMLTHGYSREDSARILQKKIIIYHQ
DIISYVKMINGSLKNLMLLDFLPGSLQLAGLIFQLMKNLNYIQCILLGEFICSLIARVFIYAHN
AHNLSQISQQLADDWFEIDWNDLPKGVKTNLHICIMRSQKSLCITVGDLDVITMKTFLVILK
GTYSYLTLLMTI
>DponOR26
MKNDFFGFCIPLARFIYIMPDKTSQNVYGWRNKLWAVFMYGLAVFCHLTEIIKLFQIVTAKY
FLLGEFIRNFVITSLHFTSLGKAMFIGGKTGKKAFEKILDFEKHVYKNLGDDIRLIYKNKVTS
IQKVKKYYLIGIILVVIFYVAAPIFREPIHIQDGNQTIRFRQVPLSSWSPFEQYYWLTFIWTGLT
GIYLSIFFVTTDLICYSFIDFGVCQLEILQYNIRNFNRDCEEITRVEKCCKIKSARVLQKKLIRM
HQDVISYVQMINEGMKNLMVLDFLPGSVQLAGMIYQMMTNLSVIQCILLGQFICSLIARVFI
YSNSANNLSQLSKQLAVDWFEIDWTELPKDVTNNLNFCIMRSQKNLQITVGDLSVITMESF
LTILKGTYSYLMLLMTI
>DponOR28
MFSASKWVIMSSGSWGLEVDSKYRILYKIYVLYIRFIYITSTVAVFAMFLVNLGSNNDKAIEA
LSLTLCSVSCIIRLAVCLKQKVVNLLKIVMEDQFNYAVNDPKIKVMLQEYKSYVTFLCVFVV

CYTYSLVILFNIFNGIIEFQSFRKLHPNATEYPQYLVSIWLPFNVQTHFTLALICQTVLFLQSC
VLNYSSTVLFNTLMIYVVIKLKILQHLFQNFNTYPKNLENFHMELRDVLAIDNLKHLIRQHQ
DIISFVKELDKNIKIGVLIEYTITSLMLATISIQVLTGNKVASFSFYGLILIYQLFLLSWNAAEIK
TQSEKIAGAIYATDWYVYGPGVKQIIHFIIMRCSRGLSLDIGPFGPNDLGAASARLKLAYSYV
SVMGNNK
>DponOR29
MAQLQILRFPRYMMLLGGVWQLPYFEDRTAQQVYKLYGLIIQSAFFLACFSMCEEFVRLVR
ADDIKQLTVNIKITFPASLLCLKLVVFQSGRFSHLLSRLVESEASFAASCEPGTLTLYLQCQNY
VEKCSKAILILAGGTGSFLIVSTIVAGLSGSSAPDLQKPMMFLAHFHFDQNLHYQRALVLQTI
TVLLAIIYFCLCPILYLGVLGFIRAQLQVLQYRFSNFNSCKGSAGDVEMMKQLIQAHQFIIWF
VKDFNENMRSLILIEFLISSVNIACVAFQLISANRITDDIFTPCFLFVLFGQLLVLAWPANEMS
MESLGVSIAIYDFPWYQKNIAIQNMARVVLLRAQTPLRLTIGLFNPLTTDTVIKVLNGAYSY
VTIMTNNKEEA
>DponOR31
MENRLKLMKFFKSTMIFTGLWRLKLTDNCFLRSLYLLYSVAIQVLFSLMFFGSLGNISEAISN
DDFPGIIDNIWFSLLTVLICIKLLMWQRKSFANLILNGIAEEQRLYMEESINIQDTFENNYKIV
KHFILLAFGCTSASGFLVWVCDTIKYFQYRSLNDTEVKPMMTLHVRYPLNRNKYFFETYIL
SLSSTLTGAIYCTISQVYFIVLTAFVISQLKVIKRLARDFHLSTESNFNEEEALTAIRNIYNKHL
YVIRFVHEVNLDIKYLILADCMVNSIIIAFLIVQVLFVTTSLSSLMFYVAVSCTVLSQIFIISWF
ANEIEVMSTSISDALFESHWWEQTEKVKRVISIIMMRSRKPLRIMIGPFYPLTIQTALNSLRAA
YSYVTLIFAISTRNTMHV
>DponOR32
MDSIFKISKFFMIINGLWVLPLHHNLIVQILYKAYSQILHFIYALFSISLIIGLIDLIVQKAPSDRV
FGSLTLTLILSMISCKIFFYLRNGVPHLFAEVIDEEAVCLNSQDPDIKKSYLNQVKYNKWAMT
VHTMCTFITVGLFIGMNVYKKINRLFKTDEVFMLELWIPIDKTENDMLIIVFNIFIIIVAFFNN
VAVSAVPQTLMVFINAQLRILQIEIRKVFDPNREELDVTHDVNRLIMKHQHIIEFASQLNNAIE
NLILLEYISDSLNVAAALLHTITIPFSGEMMYSIFHSLILLARIFIVAWTATEVNTQSMNVGIAI
YESNWTGQSNQVKMLLQIMMMRAQKPLAICIGPFRELNNEACLITMKGAYSYASLMMQK
YLKT
>DponOR33
MAIRTSLFQFAKYFMTFCGLWKVPFSPKVQRFYVVFSNVSHFVYCSFVLSLFVKALLIVVGF
ESSDNVFNAISVAVIMFDINFKAMIYVKFGLPRLFHQLMKEEQSIEENSHQEISDYYLRQCEL
YVNICTLQLVTTIFPTYFYILVNILQFPLDAENFMYEMWIPFPVQWKVLAIFKIVICQYGIFM
NTAVRSALQSLMMFITSQLWILQVNIRNVFSEFSEEAAREELGKLIRKHQFLIGFVEKVNDSV
KYILLLEYLLDSINMAAAMLQIATASSVTEMTFTLVYFILLLTQLVILAWSANEINTQSVEVS
NAIYQSNWMDQNKSIKTTLLIMLIRAQKPLGLTIGPFRFMNLEASLMTMKASYTYSRVMTE
TYA
>DponOR34
MAKSIVGITRVFMIIIGLWKLRLFQDPLKQQTYQVYSIGIKTVLFAIGASSTFRFFQLLNEDNP
NSTIVYSALTINILIYLMSASVIVFLKCGIPDVFAEILLEETRALNSEDEEIRQCYLGQAGYYQ
FTSICQIFGAVSSAVVFVSLNVYNKANGFFQEQEVFMYQLWLPFDPDKHFVAVLLINFLVLF
VAVSCNVASRVIIQTLIIFAISQLRILQILLRKVFSQDETQLKRLIRKHQKLIRFVEFLNDSLKNL
IWIEYMINTVSVAAGLLHIITASTLFERVYSSFHFVLLTVQVFVLAWSANEINIQSTKVAHAAF
YSNWIDQTEQTKKIIHIIIMRAQKPLVLKIGMFRPMNAESAVITMKGAYTYASVMLQKYS
>DponOR35
MAISLYMVAKWFMLPTGVWKLSISPNPSTRTIYSIYSVVIQSLYIMYNASMLIRLIQMWGKY
PMAELYVFLTLTILILEINYKIMIYLKNGIPQMFATVIEREESVLNSEDGEMKKIYLSRVKYYK

FATFCQCFCSGFGISWFVIINIYTKYYMQVKVDHFMYELWFPFDKEKHDTFVAGYNVVMAF
YGFLFNCASQTPLQTLMVFSAAQLEILQLKLSRSFEETVSSEEAKLEHVKKLIREHQFLIQFV
TNLNEAIKYTILLEFAVESVHGAGALLQLISTKKAIEIPYALMYLSLLVINMSALAWSANEVT
TQSENVTVGVYGSNWTAQSAPLKKLLLMVLMRAQKPLSIDVGPFRPMNNEAALMTAKGS
YTYAQFMIHSRSISK
>DponOR36
MRLMAIAEVFLKLWGLWPMTSINPFRKILYLHYKVVVIGWYTAFNCCYLVGSLRLIANNEP
SERISKCLSVLLSIGLLLVKGLLYLKNHISQLCIKVTESEVTYSDSLDSKIKEAYKRTELGNNH
LNYYILGSTFSTLIAYAGVSCIEVYNTGPDYWKMDNVSFMHEIYLPIDKLKWRWWIMVANI
ATACESVVVNIAVQSSYCALVMFGSLRLEVLRIRLKKFHKSSCNVADMKGFVIEHIDSIRYID
DLNEATKSILFITFLLNSIKVASVLFPLMAVKSLSALAFPAIFSSMLVAEVFYLGWICNEVKEQ
SLKISTDAYKILWYNEDKGVAVLLQMMIMRAQKPLTMQMGPFGPMITDTILSTMKAAYSYT
TLMLNARE
>DponOR37
MKPSKTKKAQLISWTKVFMIGGGFWNQPLTNSYIGEKVYFCYSIFMKCGCFMWWSMMVG
ELFRLVAYGYDVEIILAQFGLVVNASKIMFKLVVYIRENLLALFKDITEKDVEIWNLDNEEIH
TVYWKNIKLIKSYVLALSVSTSLCLGMLDVSGIHVILKTVEHNKAFNDTLEAHAMYQTILP
LNKLDNLPLLFTLQAYLAIIGFVYNCLTHLMFATLLVYAATQIQILQIRSKNFIGADQLSGSD
MRDKLLVLKEISQDHQYIIGFVENLNSRTRYIVLVEFILSSFDLASVSVNLITLDFSSSDIAGQL
IFNLSFFVLLSIQISILGWSCNEIKCESEELANALYASNWYLLNPKGQKMMQIMMARAQKPL
IMTIGPFGAMTTNSVLAILKGAYSYVSIMRK
>DponOR38
MSNPENQKLISWVRTLMILAGFWNQPISNNQIITKMFYVYSVIMRLSCILFWLSLVAELARLI
IYQYELSIIVTSLSVVVTDSKIMIKMILYLKNNTLNLLEDIIEKEGEVWASRSEEIKALYRRKIA
FLKVAVFIMGTSNFLTIIWLEISGVFTVIQHKEHNAIFNDSIESHVMYQTIFPLDRTNHVYWLL
TTQVLWAWVGLIFNLVTQLIFVTILLYAASQLEIMQVRFRNLIEQDFDIMAGEEKVAEKVKE
LKGLIHDLQYVVTFIKNFNKSTKYITMMEFIFSSLDMATVSVSLMKQQEYKSLWLLFFIVLL
FTQLFLIGWTSNEIKVQSEAIGDALYQSKWYVLNKDGKQIILISIARARIPLMMTIGPFGPMT
TNSILLVLKAAYSYINIMMRG
>DponOR39
MSDSKSKKVFAVMRIILMCAGFWNQSISKNRLINKIFHGYSVFVKLSCLVFWLLILAETSRLI
ICQYEITIITASVAILLTDTKIVVKIVIFLKHNILDIVADVIENKDEIRTEHYKEIKMLYDRKANF
LKFAISVLGGSTTGAVFLLQGSGGALVLQDRKHNKLFNDTVETHGMYQTIFPLHRNNHIYW
LFATEVFWSYLGITANIVTQLICVIILLHAASRLEVLQVRFKHLIMPDFQIKASDEDMKAKVT
ELKSIIRKYQLTIRFINEFNQSTKYITMIEFCLSTFDMASCCASLTKMKGYESVWLLFFMMVL
LTQLYLIGWTANEIRVQSEAIATALYESNWYELNKEGRQLILISMIRAQRPLNINIGPMGPMT
TRSILTVLKGAYSYINIMR
>DponOR40
MTIIRSADNEQFIKLAKVALISTGVWIMPITENRSVAFAFKIYSLFMKGSCVMYFSLLFAETIR
LIIFKYDMDVILASVGVLFNAAKIMLKVFIYLKYHILEHFEDVIEKERALWNSDNEELKALY
RTKVRHCNVFVITVFTSSLMAVTALQLSGAFTAFELAEYSKANNITIEPHVMYQSLFPFSKLD
NLYWWLASQALWWWVGLTYNTMTHAVFAILLIYAATQLEILQIRLRNCIEPEFSETPSQMLI
KEKVLLLRKLTQDHKYVIDYVKHFNECTKYAILLEFLLTSLDTASVSVNIIKMKGAELSWLL
SFLVLLVMQISLIAWTCNEIQVQSMAIADAIFASRWYCLLDKEAIAYVHFMIVRAQKPLLMTI
GPFGPMTTASALMVFKAAYSYVSIMKE
>DponOR47

MYSKHESLVGMLKPMMMFTGTWRLDETKSTVQWLYWLYSLIFQGFGLLFVVLVVTKFIEF
VQSGADSSDIISAQVYLLSTTSISGKVLIYQVYKVSDIFKAILDEEENLWRSEDHEFKSAYQR
EIKYIRKWNWGILLSSMFTALALMSAGVASLVPVDNSTIDSDTNHKEEFSMFPIWLPYRQSD
HRASVVALKCILTFVCASLYIVSCMIFVALMIYSVGLLKMQQEKIRKCEWNSYDMAANPSV
DMKALIINNRGVFRFIKHVDKCIRYVILVDVLLSSINIASLATNAWHFRSDDFLFSICFLLMQI
VQVLVLGWFANDIIVQSKATADVLCNMNWHYLDIKNKKMFLMMLMQYQHVSVISIGPFGP
MTIKSVISVIKAAYSYMMLMRTYK
>DponOR48
MCSENRSLLGMLKPLMIVTGTWKLDKNNSRTYRFYWFYACIYQGIGLTFAFLVTIKFIQFVA
TGADSEDIISSQVYLLTVPPILVKTFIYQTSNASDLFKSILEEEEKLWLTGDHHLISDHRNEVK
HVRKWNWGILLSSTFTAVALMSAGVASLIHGDNSLLISEGKEKEEWSMFPIWLPYNEREHK
TLAVVSKCIFTLVGGLLFAVTCMMFVALMLYSVRLLKIQHNKIRRCDWPAYNIEDSFNDIKNI
IVSHRRVIGFIRDVNKSIRYVVLVDVLLNSINIASLATYLTTARSGESLFSVSYLLVQIMQVVV
LGLLANEIKFQSQATANVLYNLNWHYLDTKNRKIFLMMLTQCQQIAVISIGPFGPMSINSVIS
VIKAAYSYMMVMQSYK
>DponOR50
MASREFIKIPRILLLISGFWPFPITKNVFCAKIYYVYSRIQLILYVSFIASITINMLILIIKHDQPT
RMFSSINVMIIVGEACLKLLVFQAKKIPSMFSHIMTYEQRIENLNDEEVNVFFNNQTIYCRWI
NCVQMLLTTVSCATYALVAVMQLIRIEDLAFYENEPFMHDLWYPFRKKEHMQWVVFINMV
IIVQGTVFNSTTQTTFICLMIYATTRFGVLQIRLKKFDRIARKEFQGNILSVIRDLIVEHQESISF
VKALNDNTKYVMLLEFLLNSVGLAAGIIQLVVKTTTGGFVAFLTIVILLLSQIFILSWSANEIS
LESSKISDAIFESNWEDYSQRIKAYFITMSMGARKPLGLTAGPFFQMSTNTAISTVKVAYTYL
SLMLQNFEV
>DponOR51
MAQASFSEVSKFFLKLSGLWPFKISDNVLVDKIYRFYTLCQICYYLCVILGLSINLVILIIRFDE
PQRIIRDINLFIIAFEICLKVVIFQFRNVPHMLYQITRYEDTIEGSSDAEVKAYYAKDAIYCRRI
NVFQFIATFLACASFAQDSVVIFLTSDDMSVFKDTPFMHDLWYPFNRADYIYLVICIAFICDT
QGLICNTASQTTLLCVMIYARTRLKILQIRLRKFDKIAVEEYEGDVVRAVKDLIAEHQYLINF
VKSLNDRTQHVLLLEFMLNSLCLASGTSQFIIIDTTSGWLATVFLNLYVIVQIFILSWHANEIS
VEGLAVSDAIAASQWQKQSKEVQKLLIIMMMRAQKPIGLTAGPFFRMTNSTAVQTMKVAYS
YASIMTQNMPE
>DponOR56
MKFFKKTEENVFFGFNIMVLKACGLWPDLIDYRYDKWRLMKDCLMISSLMPCAVPIMADF
IMQLYDGVPNLTAAVENMIALNCIIGMIYMVICFIANRRTIIKLMVNLKYFNKYGNSRKTKE
VDEKANLFSKIFMFYGILGNFVYMLMPQLSIDKCHNNRTAKMIDDGVPCGLVVRSVFPFKF
DYKPVFEIIFVHQIYTCTMVSIVVLALTMLLCGFLMHIVNQLKNLREFIAKLRHCPQDKPGE
RLFFIIQYHIDVIEYSQNTAKAFSTMLLFYITLTSLVLSVLCFEVIMVDAFEDSVRFALHLVGW
LAILLSVCYNGQLMIDESVEVANDIYSLNWFNFPVGIQKKIQMIIMRSQKPLILDAAGMGLV
SLPAFLKVLSSAYSFFTLLLKLK
>DponOR57
MEKDKFKILSLHINVLIFLQFWPNPLFNNVVNNHIMSIVCFITVTSCIPCIWTIYKVFEGMYDI
GILFESFICFVNIMAYLTAYWTIFRNKAVIENLINDICIFLPYCPTNLIRDTDASSIRYTKYLIVY
VTLGVFVNLAWPAISPEGCMRQRQSEYLLKHDPCGMPTHNYYPFDASKPIPFWIAFACEAL
LTCNICILFSMVTAIILGLLMQITEQIKHCCDKFEHINFKGDVETARKEFLECVRYHRAILEYA
ERVFTVFAPVMSAYLVVTSFATALIGYQIVETDNTQDRFRYAMLLLAWGCLFFMICLYAQILQ
DESVLIADALYNSDWTCNSIYFRHYIIRVIARAHKPLYFNISFLGKISLTRFVSVMKTAYTVFT
VLVTVVDRK

>DponOR59
MIQVNEHQILKLHIRILKCLLIWPLNSLSRKQNFYIAYGIFFTTQCCGIPVWSAAGYQFYVKI
DNVKILLEALVGIGDMVGYTLAYVCFLQNLDKINQLIVDFNQFLKYCDSKVIREAEAESNR
YTKYLLSYVTGSLVFNFFWQMMSTKTCMQQRGGDFYVKHDPCWMPVRNLYPFDANQSR
YFWIVFPIETVYSYHVSCFFSLATATIIGFLIHIGCQFKCCSAKFEHVFDTIQKDEESQKKARFE
FIELIKYYQGIQEYSQRVFQVFSSTIIVYIALTSLLVAVIGYQIVNPKISTQDRIKYAMLLVAWC
LLVYSICLYGQELQDEALKVGDSIFKSNWHKHGMALKVKPELIFTLARTQKPLEFKTQMIGS
ISLMQFMRVMKCAYSGLTLLLAVTDDE
>DponOR60
MYATSRAKPFYWALMLLNLLLWYPMRNGKSYMKIFISLSILLRTISLLSFSGTLAHLILVKKN
GTNIDISEDIGDIFGFVGCMSVSINFSIYHGSWSRFFNNLLNFGQFGEPPGYKKIVKRGNMISL
VCIIYTFPGMLWYCLLSYMQVPHCKEMNVKMGLNEPCGMINPTWLPVKEMDKSHFKLVYI
FQAVGIFVYLPSTLVISSIPLEAVEIIVARLNHLKGLFRDAFKSEDPKICSKQLSYCIRYHQDIIK
VSQELSGLVKPTMGSLFLTAAIVIGSLGSQILKASTPKAISFIMGYILTVFIGCHAGQRLIDESL
DLAKYVYDTRWYEMEPKVRKDIVFILKRCQKPITLGAPPSMGSAGYLLFFILIKTSYTYLTL
LNELE
>DponOR61
MYQPKKTDAFYYSLLFMRYLHYYPSRANESSWKIYRIISLFIRILNSFVWIECVLHFSMAVK
DNVPVDISEDIVGFTGLANSMFLCIMFELNVKEWSRVFFHITDTSKFGMPPKMSQVIDKCNR
FSWIYFLYCCTGIIIYGIINIVDPGPCERWNAEHNIHDVCRTLTPLWWPEDDIEPGLKTIIVICQ
LISCISYVPPSATLTYIIWEAGELIIAKIHHLKQLFESALDNDKLEIRRARLRFCIQYHQDIISTIE
ELNGAARKVCGQLSFVASIVFSCIGTQMLKEYSIHPMLHLIGYAMAVFLVCQNGQKIRDQTY
DIQDAVYKARWYDNITSTTKDSQLIMLRCQKPLCMDAIPFGIFNFSLLLVIIKTSYSYLTLINK
TS
>DponOR62
MYFPNQNEPFYYTLRFLQYSSYYPWPNQAEKWYNSTSILVRVFTSFVSIECALHCTMALKD
GIPVGISEDIVGLTGVANCMFVSAMFQLQAKRWSSCVLDIIDTKKFGLPPKMPQMVKKTERI
AIWYSIYCTVGILTYTFYSAIKATSCHEQNLKKGTHEICGMITPFWWPYKDINPIVKLLFNLY
QLVSVLLILPPSALVTFASWAASQIIKTKIHHLKGLFADVFSDKDSKRQKARLKCCIQYHQHI
FKISKELNGITRLLLGQSSFSSAIILSCIGSQILKEYSVGAFLHLMGYTAAVFLLCETGQTILDE
TFNVRHAVYASDWHLADSSLAKDIQFVLLRSQKPILMDSYLFGSFSYSLLIVIVKTAYSYLTL
MNKSSI
>DponOR63
MYPIRKDLPFYASLRMLESIGFYSENTNGFKKRSIVRTLIFCILCWSIILLSAVLLIYENLNDKN
YASVFFNIAVAVASTSTYCCTLLFVKYQEKWSDILTALVNYEKFGKPRRYNQLKERGDRVAM
ACWGGILTGVFLYMLFAILHENDCELEKTGGGVCGLIIPTWLPAPYDNSLLARRLVLLYDIPN
AAAVSSFVLVTHLNIQVNEFNIARIDHLSLLFNDIEFCKDPQAQLNKMKHCIEYHQDIIRVSL
QFKNLSKRTMGHMTLTFTIVTASMGCQLLQTSKNFYQENAFFFEIIYVINMFIMCYCGQRLE
YKMKTVGDFLYSTHWYNLNPKLQSLIPLVILNSQKTIRMDAVPIGYLNYELFVTLLKTTFSY
FSVLTQLT
>DponOR64
MLYPVRKGLPFYHNLLVLKLAGYYPKSSNYNKKLFFYCLVCWMSLWTGTLWNLIILLYLSI
QNKSSHGITEAMGYLIGNSSLALICLHFALKHQDWSHLMDALIDFQKYGKPPKFNTVQTNA
SKVGITFFKVLFCAAVMYCVLQVLLEEECEKKLTFGKTSCGMLLPTWFPASHAESKLAKRL
LLIYQLLACWAIAPFTVIVSLVLQANEFIAYRIDHLKSLLRKVGSNENPDLQLHHFLAYVQY
HHHIIRLCRKLNYVAKYTTGHVALTFVTVVACFGHHSIQEKSLQSLTYQATYVISMCILCYAG

QNMQDQMRSIGDALYSSTWYNCSLKVQKMIPLVLLRTQQPIGLDAVPLGVFNYMLMVMV
LKTTYSYMSFLSRTI
>DponOR65
MEGPKRVKCCKVSLNFLRIARILPAAGMENSRQYLAAIAGIFSLVTLLTLLVISYVFVGLNNH
FPMVFIQFPIVCLVSIWGVMWINGFCVKNRHLLLQLEEALADCSTFGKPPAFKIFQEKMNLL
AKLHLSYVTGGALLYFIFFAPMHKKNCEALNIQRNFSEICALLIPIHIPLVPYAALEKFPFLQLI
NAAEFLMLMYIYVVCGTVVWLNVEVIEYICLRIRHLKTVLLEALACPNRATRRGLFGRAVQ
YHEEILRMGRLADEFFGTELVLHVVLTGAILGVSAFVMLESATLETLMIFVGWLNAIIMGCA
AGQRLINESATISDVLHEVDWFEFDNGLKKDLLFFLVRSRKSMYIRAGNKLSELIEFFQILRT
SYSYVTFLNSIDGN
>DponOR66
MLRSKHLDALFQAGIERGDIFWMLDISVSAVSGNCVIFAYTLVILLQCVIEVTGQLYLLTTKP
SIGKILLLAPMFFTSFMVITTIIALLFQRSSHGRIYSQYEALSMEQLRRANKPILAEVRQLFDA
GIYQFAAFLAAVALGSIGYLPLNEFDYEHNVWPAIQALKLIEDGPKAVIYTIATLNYLVMPGK
GCILLLYGLHIMHLCSLYCVSSILLRGKLKGIAVNRPAAADLMLSDQEWVTQELKSCIKQDV
RLKTFCSAIIDHFKWILLFHVALAVIILSLLYYINLFLNGGYSPLGSRCFVAIANILSFTYSYNS
ENFREQVENIRQEICNLPWYSFNKCNQKLVHVFLSNMLQPRYLSVGGLFDANHEFLVLVMH
KVFNVLTILSNLDPDRAGN
>DponOR67
MESKSAKEQLKSILIGMKNTFKPENLFKSQMEDTHYELSPCVYFFTLSLPCHYPILRKLNSY
LICLNILTFFTEVIYQFTCHIDVLVLLQREVMRFYFLFGSLNAFYILNLQDGLKESEKEIKKAQ
WPLKLVSTDVKCAIGKRGLMQSLASEFCVLMGCTYICVTLRELKSTCVMAYVRNLRIKPLE
YLVYFGECINYVNCIYLISIIPFLCIYFATEAEVQIFLLSEYVRKITVQKQLIIKKNSPKLELQVR
CRLRMLMTRHLELRQLYVNNKGMIRHTAVQYMIPATLSFILNLYAHAEKIMNPVSTIMYFAI
FIIIAMYTMFSGQRFEDEWRRYYNELWNLPWYTWSVQNQKAYLLILTGASNITAVKIFNTTA
VNYALLLRIWKFVYSTANAVYNIYRK
>DponOR68
MSLQLIKDELKVFIKIVFRLFQLKYLFKFEMEDTNYVLSPMLSFYVVDYFCHFTFIRIWNLM
YVYINAVICCLEVALFFRTDMVFGKHGFILGFQLLGIIASIIVVLVKPHLLEANKRFKDFQWT
LKDSKPAVKVTIGRLALFQSLMFELIVIVSYIDYLFVIQQINTTSIMNFVQEIDQAILTNFVYLI
ELIDIWTICYFVLILPHIQIYFATAAQVQVFLLVQYVKDFNKALHQNPNERNGDRQTADCLK
RIVSRYCEIKQVFLDKKTIFRSAGINCIVPTALLSIYVIYATVEGRISFMTAIMHFLVCFCVLIFA
IIAGQGFEDEWKYFYESLCDLPWYRMNLANQKMYLMLLIGANHEVQLNILPSVAINFSLCI
KVWRILYSSSNMIYNVHLKST
>DponOR82
MSDSSDYFDLNYKISTWFLLIPPKKFTLKIIYWIIATPHFILACGSAFLEISKLFIGGSGEFSANI
LNLGVSALHFMAVNRVSRWLFKKGEFDIILERIRKLSMDYSLFEYQSNCLESNENQPNQPV
QPTIRRVFVAEAEINPIVEYKLEKMEKSKIHCLRITIVFLIYVVLNLSISYAFNYMGPTYEKWN
PARNKTFVYRDYPYPLWYPFDTSISDGYYLLGFFYQPYAFFFIMAAFLSIEDLSVAAIIHLTTH
IKILGYAFGYVDHNINPMLEHHEIIELKQQRIIKLINDLKEIYKCAEILNSYLSDQLLIQEFLMS
IVICCSVYRVTVVTSSTEMSYLSTMAVIIGADMFIVSWYNQGFTLEVFELQHRIYELEWIDY
QPKLRKLLLFLMCRVQKPFHFTMGFGFPLDARVFLSMIKMSYSFYTLITRSSQKLTANT
>DponOR83
MDYFDLNYRISAFFLLIPPKISILKIIYWIVAVPHFFLICGSAFLEIGKLFMGGSGEFSSNILNLG
ISALHLCATNRVSRWFLIKNEFEVILANLRKINTDFSLFDYQSHQSGFKMDANEPENAIIKYK
TTKLLETKRYCLGIFLTFLINVALNISISYAINYGNPLYEKWNPLLNKTSVYRDYPYPLLYPFD
TSVSDGHYLLGFFYQPYAFFCLMCAFFCIEYLCVGTIIHLTTHVNILGYAFSYVDENIDPMLD

YSKVIMLKEKRIIKLSGELKEIYNCAKELNAVFSGQLLMQEFLMSTVMCCCVYRVTTNISTA
EIGYLSTMVAVCVAEMFTVSWFNQCFTLELFKIQQRIYELEWIDYPPKLRRVLLFLMCRVQK
PFNFTMGFGFPLDVNVFLSMIKTSYSFYTLITRSGSKFSNEDV
>TcasOR26
MEGIVRNSFTLNLTIMKLMGIYPLENHSRLYKVFGYALYIFSIIPGSVLGFLQLFFKGDITGVG
YKDLSSVVVIFLSPKLCSMVFAADNVKKCIDYLDEGYFTIKNQNQEQIVTECVQICRRNSVI
FLGGCTVSFITWSGTLLYRDDIKQLPLIAWLPFNSQDYSLLHYVLYCSHGFGVAYVAFAAGT
VDPLIPGLICHASGQVQILKDNLQHLDDYIDRSDLVYEKIKECIDHYGAIINFVFKYERSFSV
VILCQLLESAIVIGICCLQISKLEAYDINLIIMGNYLVFLLIQVYFYCYYGTALVEENNSLINAI
YMNRWYEYKKESQKALIILMECSKKPLLITAGRVVDLSLETFTLILKRSYSLLAVLKNY
>TcasOR27
MLETKQIVFHSFKLNVTVLSLIGLYPPKNYSILYKIYAVILFLAVHTPQLVLGLLHYFLMGDFT
SIDYSDFVTVGMMFYAFKLLPFVTSVTKIQKCINYFDTLGYKILKSEEKIIEDCVGSCRRNTN
VFFVGCCLSWMGFVAQVFLRDEPQQLPLKVWFPYSRDESPVLFYCIYILLIFGPGYSVLACG
TIDPMIGGLAYHAAAQLQRLKRNLQYLDEYIKEKNVGKSKENKRGVIYEEIISCVQRYQEIA
TFVDLFKDSFSQVVFSQFMGSVFLIGLCCFQIITATEVDINFVITANYIWVILFQIFFYCYYGT
MLIEENYTLTNAIYLSNWYEYSIPEQKALFMLMERSKKPMIVTAGKILDLSLDTFTMILRRS
YSLLCCLK
>TcasOR28
MHSFFNIKVHNKMPVTVTDCFGVNLTVMGLFGMYHRSGNPTIFEKVIAYCMFFFFTIPIPILG
SLYFFFQENVDLEELHNNGFLIAEMVCNLAKYLSFIKNGHRIRKCIHYLELPIFATRRENQERI
INASGWICKRNSRVFFVSVLAANVFWAGRPFMENKQKFPIEIWLPFDAKANAMAFYLIYLF
LVIAVAYASIACAVIDPLIAGLASLASGQVEVLKDNLENLSKYTREQMLEKGIICNKFDEVFY
ANVRKCVDHHNAILHFIKEYEECFSSSVFSQFLGSILVICFCCLELTKIEPLSFNFYSMTFFATC
FSAQIYMYCYYGTVLYDESNSIASAIYMGTWYEYDVKSRKALINLMERSKKPIHLRAGKVL
EMSVETFTMILRRSYSLLAVLKNY
>TcasOR29
MFNHPNYYTMSNLIEDSFGISLRVLEICFLYTQRKPKFLHRVVSYLLCSNVTFTVPVFGLLYI
FLETNITMKRMADDAFVVAGMWCYGPKLWPLLTKRTQIEKCIRYFENPQFVTLRENQTEIIK
THSEICKRNSKAFAIFMTTAVLIWATKPFLFGENNFPVDVWLPFDPKTDLKKYYLLYVFVAS
GVVYGCVANGAMDPLIAGLVCLAAGHFKVLKDNLQYLYKYTREDLEKQRIKIPTNQIFKC
KLFHQKLKDCIRHHIAILQFVKMYETNFSFIVFNQFLFSILILGFLIFQLSILEPLSYEFLQAIIY
MVCVVVQIYFYCYYGTILYEESNSLNDAVYMGNWYKYDIKSRKTLLILMERSKKPCIVTAG
KVLDLSLVTFTTILRRSYSLLAVLKNQK
>TcasOR30
MSSLVQESLHINLRVLELLWLYTPGDQTNFQKVRSCILFFVIMFHVPVLGGLHFIFGKNNNPT
KLADNAFAVTAMWCYAVKLWPLIGNRSKINDCINYLDKPIVALRENQKVILQTCSKICRRNS
SIFLFYMTIAVIGFVTKPFLFEERGFPVDVWFPISLTDRLDVYWGFYIYVAIGVAYPVIASGVL
DPLIPSLLCLATGHLKVLNDNLKHLDEYSNEANGSKDNNLYKNIQKCIKHHTEILNFVEFHQ
NCFSYMVFSQFVSSSLVLCFTCWNVSILQPFSFEWFQSLGYFLILLVQLYFYCYYGTNLSEEC
DKITTSVYMGKWYKYDVKSRKALILLMERSKKPTIVSAGKILDLSVETFTTILRRSYSLLAL
LKNQN
>TcasOR32
MCLSTSEQSFSINLKIMKLCRLFPPTEGKKFYKIQAYLLQFLLLLPIPILGNLHLLLDENLDME
KVNYNAVFLAQVTCFVIKLMAIIANSEKIKKCITELDSPKFAAVRENHKIILQHCIKVCKRNT
LIFVVFVICGASSWATKPLFWSRRNLPLDVWFPLDTTSTPVYCSLYIYLLIGVYFTSFANMVI
DPLIAGLAYHATSQIKILKDNLQHLNNVYANEEITSSKNKIIYMKIKRCVQHYDDILSFVKEF

EECFSLAIFSQISASVFVICFSCLQLSKIKTFGYYFIQLVFYFGVILAQIYFYCFYGSTLFEESSSI
INAVYSSKWYDFDVPCRKALLILMERAQTPITVAAGKIMDLSLVTFATILRRSYSLVAVLNNY
Q
>TcasOR33
MSDIIGQSFGPNLLIMQILRLYPSQRYTRLHQLQACIMYVFFIILVPTLTIYNYIIQENFDILQFN
YTATFLAETVSWIAKLLPFITNANRMKKCITYFGTRYFEEMLQRYTSAKIMKECISVCRRNS
TVFLYGVICGMTSFITKPLFWKGYQLPLDMWLPFDATSGPGIYYTTYSFLAIAISYCAFAGTL
IDPLIGGLACHATGQLKVLKVNLQHLKEYTETEVKQSDETSDQIMYEKIRQCIDHHNAILIF
VKEFENCFSLVVLSQFTGSMVAICFCCLQLSMVDLVSMSALSSTIYIFIILGQFFFYCYYGSRL
FEENNSLTNGIYMGQWYEYNIKLKKALIILMERSKVPMLITAGKILPLNLETFTLLLKRTYSL
LAVLKNYQQ
>TcasOR34
MSSLIQESLHINLRVLELLWLYTPKEQSNFHKMRSCILFFLLMFHVPVLGGIHLIFGINDNPTK
LADNALTIAGVSCYALKLWPLIGNRSKIKDCINYLDKPIVELRENQKVIFQSCSKICRRNSKIF
QIYMITALIGFVIKPFFFEDKSFPVDIWLPKALRDRLDVYWSFYFYVSLGVAYPVIASGALDP
LIPSLLCLATGHLKVLNDNLEHLDEYSNEISESKNCVLYKNVQTCIKHHIEIMNFVENHQKC
FSSMVFCQFLGSPLILCFTCWNMSILQPFTFKWFESLAYFLILLVQLYFYCYYGTNLSDECAN
VTTSIYMGKWYKYDVKSRKALILLMERSKKPTIVTAAKILDLSLETFTIILKRSYSLLAVLKN
QN
>TcasOR35
MEGTHSIKPNSLSSFSLLMVMEGLVKKSFRVNLLVMQILGFYPPQKYKILYKTYAYVVYCAF
TILIPVLAALNLLLGEDVDLEQISDNAFLVCQTGCFIIKFLPFVNNVAKIRNSLFMIECPLFHIY
TKSQEYIIDECVATCRRNCRLFLTFCIITLINWSISPFFLPGNALPVEIWSPFEHKASRKFYFWS
FVYVVAGVGNGAISSGVIDPLLAGLISHATSQLKLLKNNLQFLDEHAEEKIASLNISFTERGT
VKADFIYQQIKLCVNHHIAITEFVNVYENTYSSVAFIQFAASVVVICISCLRLSMVEPFTFTFF
AMVLFLCTMLSEIFLYSYYGTILYEENQTLTNAVYMGKWYNYDTKSRKALVILMERSKRPM
LVTAGKILDLSLETFTTVLRRAYSLLAVLKNY
>TcasOR36
MKGLVEKSFRVNLLVMQVMGFYPPQKYKSLYKIYTYVVYCAFTTLIPVLATLELFLAENINL
EQISDNAFIVCEAGCFIIKYLPFVRNADKIKKSLFLIERPMFHIYTKRQEHIIEECVAICRRNCR
LFLTFCTITVINWSITPFFLPGNNLPVEIWSPFEHKASRKFYFLSFVYIVAGVGNAAVSSGVID
PLLAGLISHATSQLKVLKNNLQFLDEHAEERIASRNISFIERKRFKADFIYQQIKLCVNHHIAI
TEFIDVYEDTYSSSVFIQFAASVVVICISCLRLSMVEPFTFTFFVMALFLWTMLCEIFLYCYYG
TILYEENHSLTNAIYMGKWYNYDIKSMKALVILMERSKRPMIVTAGKILDLSLETFTTILRRA
YSLLLVLKNYESTPTE
>TcasOR40
MSSLIQESLHINLRVLEFFLLYTPGEPTNFQKLRSSILFFALMFHVPVLSGINLIVGKHDNPMK
LVDNSFGFVGLSCYIAKLWPLIGNRSKIKVCINYLDKPIVELRENQKGILQACSKICRRNSNIF
LYYMIISVTGFVTKPFLFEERGFPVDVWLPTSLKDRLDVYWGFYIYVSIGVAYPVIASGVLD
PLIPSLLCLATGHLKVLNDNLEHLDEYSSEENGSKDSNLYKNIQKCIKHHIEILNFVYNHQKC
FSLMVFSQFLGSPMILCFTCWNVSMREPFSLEWFQSLAYFLGLLLQLFFYCYYGTRLSEEFE
HVTTAVYMGKWYKYDVKSRKALIILMERSKKPTIVTAGKILDLSLETFTIILKRSYSLLAVLK
NQN
>TcasOR43
MDDTPVKKFFKVNLTVMRLFGFYPPKKCNIVYKIYALIVYVCCTVTIPTLAALHLLVSENVD
LAQVCENSFVIFEVGCFMFKLKPFIFNVEKIRQSIYMLNWPIFNNHSKQQEQIINECAWTCRR
NTWLFLVFCIITFITWAATPFFTSVYKFPIDVWLPFEATADPKTFFSVYLFIVLGIGNTAISNGV

IDPLISGMVYFAVNQLKVLKDNLEYLENNSETQLCFNLTVKHKTIYKKITMCIEHHNAILDFI
KYYENVYSPVVFTQFTAAVLVICISCLQLSMVEPLTFTFFAMITFIVTMFLEIFLYCYFGAALY
EESNTIIRAVYMGNWYTYDIKSRKALIVLMERAKRPIIITAGKILEVSLETFTMLIRRAYSLLA
VLRNYQS
>TcasOR44
MKQIVKPSFRIHLTLMQILGFYPTRSYKKLYKLYAYFVYCFLTIPVPTLATVYLLVEDNIDLLQ
LSNSGFLICQIGCFIAKFVPFWKKPEKIKRSIYMLENQIFTNHAPEQCKIIDDCIRICNRDCWL
NLALVLSALTSWAVTPIAKGISKLPVEMWLPYDVNKDDKTYYLTYLFVVTGTANGAIASGV
IDPLIAGLACQATGQLRILKNNLQYLWENVDKEISDSEKDNALREKIMYDKIRSCILHHNSIL
NYVELFEDTYSSVVFTQFTASVLVICNACLQISMVEPFTFAFFAMISFIVTMLAEIFLYSYYGT
LLYDESNSLTNAIYMGRWYEYDIKSKKALIVIMERSKKPMIVTAGKILDLSLVTFTTILRRAY
SLLAVLKNY
>TcasOR45
MNNHVQKSFGVHLRLLHILGMFPSRRISKIYNIYTYFMYCIMITPIPILVIINLFLEKHVDIVKI
SNNAFLTCQMACLSVKLIPFLGNIESVWTTLDMLEKPPFTTYTKRQEKLVDECITTCKRNC
WLFLVFCIFSLISWASKPMGLEGRNLPLEIWVPVDFTENTELYCFAYIYVVLGVAAGALSNG
VIDPLIAGLASHATTQLKILKNNLQFLSEDADQVIAQQLTKARIIQDKIRFCIIHHNAILDFVD
YYENVYSGIVFSQFIASVFVICLACFYLSIVEPFSVIFFAMVIFLVTVLSQIFLYSYYGTLLFEE
NNTLTNAVYMGQWYTYDVTSRKALIILMERSKRPMTVRAGKVLDLSLETFTTILRRAYSLL
AVLKNY
>TcasOR46
MSKSEKIHTLATYFDSNIAFLKLTAFWIYDDETTRRKKYLQHAYNIFWIFYLFVAYQPAELLY
VYYSFNDLSVFLRALRDIGNHVSLAYKAFNYFIMRRDILKLMETLQHGNYHYEDCGDFQP
KLIVDEEKKEALKWTKYFLNFCNAICLSMFANGVFTFIFLSDKQYVERNGQRVYHQEQPVN
TVSPFGSGTKLRFFVTFIYTMIALTFYAWTIVALDSLFITIMSCISSHLKILQGAFKTVRARFIK
LCASLSKLLISVSGKLESIYSTQTFVQTFISLGEMCFSLYLLSETADQNIGNEITYLIATGFELL
MYCWFGNRITEASLKISYALYESDWFPTSLSFKKQIIFTMTRMQKPINVTIGKITPLAFSTFLTI
ARGAYSFFTFLKQRHGINH
>TcasOR47
MYLDLNKGIADNTVLFRIMGHWPFGNPKLYRVYTHFVLINMYLYNLTSLINMLKNLDDTEE
VTATIYNLLSTVAVIIKANIFHYHFNHVKTIVTMFESEAFQPKNKQQEKILKNGIFWARFIFYY
FFLTADLTLVMWILFPIMDGERRFPSNAWFPYDYLSGRNYTLTYIWQSIFIIYHALSNVCMDT
FFAMLMVQTGAQCDVLNNQVSLLGKESVDSDTVRGELGKCIHHHKLILKLAETIGLVFRNI
VLVQFATSVSVLCETMFLLSLVKTLNATFVMLLFYQVAIFTQIFLYCWFGNEVVLKSAKLYY
SAYESRWYECPQSFKKDLLFFMQRTQKPIVLFVGKMFPITVITFTSILRSSWAYFMALRKVH
DKS
>TcasOR58
MPFTIKDYDLRNAFETERTLLTLSGFYPRRTKKYNFFYNTSALINLFIAYGQLFSMVVQMVI
DRNELSKLSETLLFFMTHFTFLCKLTNFVYYKKKMFEIEDNLSRKIFYGFELWQIKPKIDSCK
FIAKIFRILCILVVLFYTLVPYLDDKEDLSLPLPGWLPYNTKKYYYPTVIFQVMSVSVSAYNN
SSIDVLTCMLITVASAEFNLLKGALKTIDFHPKGHNTKQLIEAKFENCVNHHKEIVKFAYQIE
TIFSKGIFLQFFASIIVICFTGFQMIVVPIPSMQFIFLIIYFSCMMCQVAMYCWYGHDIITTSDSI
GQAFYMSNWYESDVKIRKNICIFLERTKKPVILTAGKFVTLSLTTFTTILRSSYSYFAVLQHLY
KEDS
>TcasOR59
MDEEFLIGTFETEKKFLRYGSFYPCGKRIKFIFLGLFMFVYSWTEFLSMITVLFVERDNLTKL
SETLLFCMTQAAFLFKLVNFLYHNKTMLRIESILKNPILNCLDQFEKNIIEKYMIRVKYLARL

FRILCILTVSFYGLFPFIDEDPDHMLPLPGWFPFDVKTHQIELVIAQTCGIAIGAFLNSTLDILP
TILITLGSAQFDILKIRLENITSVDTSKSWLVKKAIKKCVIYHTILLNYITQIEILFHKGIFVQFT
ASVVVICLTGFQMLVISVRSIQFILLMIYFSTMTCQIALYCWYGNELMYRSMGLSDACYMSE
WNKCDTSVCKSLAIIMERGKRPVVLKAGNIFSLKLTTLMTVLKSSYSYFAVLQRLYATSE
>TcasOR60
MSEDYTFRNVFAREKKILTISGFYPLREYEKNYFHFFSGTIQWIISLGMLFSMIIQSVIKRNDL
MVLSETLYFLTTHLTFVCKLANLEYHKKLLLDIEDMLKTTRFQKTLSLDLIEKTGMNEKIRK
FNLVAKTFRIVCVWCVVLYVLVPYFDPGKSKTLPTPGWFPFNWTDKYYYGTYFFEVAGISIT
AHMDSSIDILSWLLVTIASFQCDILKENLKNIYYNYDKEHDIRETFKDCIRHHEEIIKFTTKVE
QSFSQGILLQFLCSALVICFTGFLMLVVPVLTFQFANTIMYFCCMMIQLGMYCWYGHEIMTT
SDEIGQYFYLANWYDSSLTLRKDFAIFLERAKRPITLTAGGFVVLSLNTFTRILRSSYSYFAVL
KHLYNKS
>TcasOR61
MGDYDFRAAFAFEKAIFSLSGYYQRQAGFSSLIICAIASLITIAQFLSMVMQIIVAGNDLTVLS
ETLLFFMTHFTYMCKLVNLLFYKSKLLHIEDLLSRPRFYGFSQNELTIIKDGIEATNTVANLF
RIFCVLACIAYGLVPYLDHTKAMALPLPGWLPYDTTKYYYPTYFFQMVAVSITASVNSTIDIL
TWKLITIASVQFDILKRKLKDLDYKLETTSLQIQFKTCVKHHKEIVNYVKNVEKTFSKGIFIQ
FFASVIVICFAGFLIIITPVLSMQFLYLTLYFMCMISQVAIYCWYGHYVMTTSDEIGQDFYMSN
WYESDVAFRKDIIIFMERVKKPVTFTAGNFITLSLVTLTRILRSSYSYVAVLQHLYNEV
>TcasOR63
MGFMIQDYDLRNAFSLERKLMLVVGFYPKRDNKHEILYWLSAFFNLLISYGQLTTMIIQMV
FDRSDLSKLTESLLYFFTHFTFLCKLLNFQYYSKDLIEIENFLTDPIFYGYSFEQLDIIKAKIRSC
AFISNAFRICCTFTCSFYCLVPFIDESRKKILPLPGWFPYDTTNYYYSTFFVQSLSLFISAYCNT
AIDILTWKLITLASAQFEILKENLTKIDYEGGFNETKGALVRCITHHAKIVNYTERVEAIFSKG
IFLQLFGSVIVICTTGFQLIVVPIPSVQFAVLGTYLCGMTTQVATYCYYGHEVMTTSDAIGMS
LYLSNWYASHVKIRKIVMIFLEKTKKPTIVKAGNFITLSLATLTQILRSAYSYFAVLQRLYKDS
>TcasOR64
MMSDEYVKDVFIANRWMLRCAGLWTPSTRSKLVQIPYKIYAIVVFLFVNVYFTSTEFLSLFY
THKNLYNFIKNVNFFLTHFMGAVKVIFWFFKGHVLRDLMRTLESPEFHYEPCEGFQPGLIW
RKYRRIGFKYSLGFLALAHMTLSSSYIPPLLTKLPYFSWMPFSYSTPRSYLLALGYQAGPMF
SYAYSIVGMDTLFMNIMNFIAAHLVILQGAFASSKMRVLDPGQMNNEMKRNCRHLQTILRV
SEDLERVHRYLTLGQLTATLFILCTSLYLISTTPASSKQFYAELVYMVAMGFQLYLYCWFGNE
VTLMASEIPVNVWKADWYDCDQSFKKSMIFTMTRMQKPIYMTVGKFAPLTLQTFVYILRT
SYSIFAVIKNTSI
>TcasOR65
MTATKSLKEIPPIYLRVHLTVLQILGIDILPVESVPQNLFYTYTALIISTMCLFTIAEFLDMVLN
YEDIYRLTFGLCYCVTHVLGTVKMFLMLYLRKKLWGNLTTLEEGIFKPNPTRGGPEELQIV
NDAITMCNRQGYVFYTLVFLIIGARLLYASLANWPYDKHNYFDGNVTVIVNTKEMPYTTW
MPFDYNDSPLYETIFAFQIFSTTVYGFYIGAADAVICGFMMLIKAQFLIVKRELETLIERAQK
AAIAENPDNEDNFGREIERIELLDKRTQDYVAKYANECVYHHQELIALCDHAEEDFCYLML
LQFISSLLIVCFQLFQVSTLSPDSVEFFSMVCYLLLMLFQLLCYCWHGNEVQIVSGELSRYAF
GINWIIMRESPKKTLLLLMMRAQRPCYFTAGKFSLLSLQTFMTIVRGAGSYFMFLRQMNI
>TcasOR66
MSKNLKEIPPVYLKVHLTVLQILGIDILPNERIPQTLFYTYSVLLIATMVVFTTAECLDLVLNY
EDIYKLTFGLCCCVTHVLGAAKMFLMLYLRKKLWGYFTTLENGIFKPNPCRGGAEEFEIVT
SAINMCKRQGYVFYVLTVGVTGGQGLYAALANLPYDKHNYFDGNVTVVVNTKQMPYAT
WTPFDYNDSPLYEIMFAFQIFSTTLYGFYIGAADAVICGFLMLIKAQFLIVKRELETLVERAQ

RAGNPDRGDFGGGINRIEMLDDGTQVFVEKCANECVYHHQELIALCEHAEEDFCYLMLLQ
FISSLLIVCFQLFQLSTLSPGTFEFFSMACFLLFILFQLLCYCWHGNEVQFVSGELSRYAFSIN
WIIMRESPKKTLLLLMMRAQRPCYFTAGKFSLLSLQTFMTVVRGAGSYFMFLKQMNT
>TcasOR67
MDFTIRDFDLRNSFSLERKLLLVLGFYPIRDKEKHRILHQLSAFLNLLLYYGQLLTIIIQMVID
RNDLSKLTDSTLYFLTLFTFLCKLFNFQYYGKDLIEVEKSLTDPIFYGYSFHKLQIIKAKVRSC
TLVCLAFRISCTCSCFIYSVVPFIDRSGQKTLSIPGWFPYDTAKHFYITFFLQSLSLFISAHCNS
ATDTLPCKLISLATAQFELLKDNLRTIDYENSFEETKHALVKCITHHRKIVNYTKRVETIFSKG
IFLQLFASVLVICTTGFQLVIVPFGSLKFAIHGIYLCAMTAQIAIYCYYGHDVMITSDEIGTSLY
MSNWYASHIKIRKIMVIFLEKTKKPTIVLAGNFITLSLVTLTQILRSAYSYFAVLRRLYADD
>TcasOR68
MPSLIEQSFKINFRILKITAFYPPTKNKRLNQMLAFVLYMLFVVSVPILEVLNLVLQEKITFKQ
IIDNAFMIAELGCLIPKYWPFVRNNDRLVKCIHYFDSPAFQPTKKKHREILQNCVKVCRAITI
FFVAAVSSGYVSWSSRPISWKNHIFPTDLWLPYDPKVAPKLYNFLVYTYLIKILKDNLQHLG
EDTEAEFNQQSVINRLPKSEIMYRKIVKCVEHHNLILEFVKEFQYCFAQCAFSQIAGSVVVL
CVSCLQLTIVDLLSFDCLAMILFLVSMLSEVYFFCHFGTLLYEESSTISDAIYMGSWYDYDK
KSKQALTILMERTKRPVIVIAGKLVQLSLITFSMILRRSYSLLAVLENYNIEIN
>TcasOR69
MPSIIDISFKFNLKVLRISGLYLPEKFKSLYQIYSYLAYFLLVIPVPILECTNLLVQEKITFRQIA
DSAFLIAELGCFIPKNWPFVRHADRLKRCIHYFSAPIFKTKRKEHEEILQDCIKVCHRSTAFY
FASVTVGFFSWAIRPISWKNHIFPTDIWLPFDPHTASKVQVAGVYFYLVLGKGFSSKVLKFW
FNFSCGIKILKNNLQFLGEYVDEELATQMHSLSPSEKTQLTYQKIRQCVIHHKHILAFVKEYE
ECFSQVAFSQFIGSVVIFCVSCLQLTIVDVVSLDFLAMMMYLIATLSEVYLYCHFGTVLYHES
NTISEAIYLSKWYEFDIKSKKALSILMERLKRPMIIKCGKILDMSFITFTMILRRSYSLLAVME
NYNIELN
>TcasOR70
MPSIIDISFKININVLCLAGLYLPDKFKSLYRVYTYLVYVFIVIPVPTLGCVYLLAQEKITFRQI
ADNLFLIAELGCFIPKYWPLVRHAERIKRCIHYFSAPIFKTDRKEHQEILDDCIKVCHQWSAF
YFASVTAGFVSWSIRPISWENHILPTDIWLPFDPHTASSAKVASVYFYLVLGKGFGLGIKILK
NNLQHLGEYVDEELASLEPCRKAQLTYQKIRQCVIHHEHILAFVEEYEECFSQVALSQFVGA
VVIFCVSCLQLTIVEVVSLDFLAMMMYFIAMLCQVYLYCHFGTILYDESDTISDAIYLSKWY
EFDKRSKKALCILMERLKRPMTVTCGKIFTMSLVTFTMILRRAYSLLAVLENYNIELN
>TcasOR76
MMESTVTRLKRMYLWPTASVTSRKPAFFLITFSCFLLYGSVMHLIVNDISMEEVHVIETTAG
QFGVLYYLTLFTIYRKGILEIYADLSNFTKFGKPYNFDKRNKQLNQWSRWFSVVLYFFVISV
FAWPGIFTQSCEDLNVALNKTEVCGVVSPVWLPFRFDYKPMKQFVYFWQSFCCLYSNGGA
GTISFAMSETIEHLILRVEDLKILFPKIVAERSPEVRRKMLAKWVDYHLWLLSIGKLMNDTY
RYSFSVIVLCAGTLFGCIGYTVMKNASTNFNSSFIFFGWMESVFVICVCGQRLMDAFHSVG
TTVYNSEWCDTDVDFQKGVILITIRAQKPVRIYAGPFSYVSHLLILTVFQTSYSYINLLNASS
>TcasOR77
MKYILMKKTIAFLSVTGFWPKTKESTKTRAFCILFSSSFLLFGSLGYLIVYRKFGSDDIDSIET
ATSHFGVLYFMFFWILKRDGLVHIVNLLSDFSKFGEPRFFNDRNRQLDYLLQYCIFVLSVAT
GGVFLCPIIFVKNCEMVKQEKNLTKVCGLVSNVWAPFDYSEYPMKRVVSLWESYCCFINFG
CGGIMSFTMIKTMEHLHIRVEQLKDMFPDVVNEKNLAVRKQKLEKWVKYHLHLYDIGELM
NNTYRYCLSVIVLCVGILFGCIGISTMQPGSSHNSLFLFMGWFQSICILCMVGQRLLDVFLSV
GVMAYDSAWYEKDVDFQKAVLMIMIRARRPVLIYAGPFTNLSHLLILGVLQTSYSYINLLN
AK

>TcasOR78
MGHAIMTEILTYLTLMGFWPRSPKSSKASAFLIILSTSFLFFGILFYLIVNRQFGSSEIDSIETIT
SQFGVLYYLILFTWKRNDIVEIVELLSDFSKFGKPPFFDQRSTRLNYRLSCIVLILIVANIVVA
ALPVIYIDSCHKANEQLNLTKTCGLIAPVWLPFDYNEYPRKHLVFAWEVYCCVMNYVGSGI
GALTMVGTMEHVIIRIEQLKYIFPKILDQPNPRIREQMLKNWVRYHLALFEIGRLMNDAYK
WSLSVIVLCVGALFACIGISMLQSTASQINSICLFFGWFPSIAFLCMWGQRLLDSSLSVGTAV
YSSRWYDMDVAFQKSVLMILIRSQKPIRISVGPFTHLSMLLLLGVFQSAYSYINLLNATS
>TcasOR79
MGHVIMNEILTYVTLLGLWPRSRKSTKTISYLIILSSSFLFFGSLLYLVVHRKFGSNEIDSIETV
TSQFAVLYYMTFFTLKREGTVRIIDQMSDFSKFGKPPLFDQHNKRLNYLLSYFVICLFVAIVG
VVALPAIYTGSCHKANEQLNLTKTCGLVAPVWLPFDYNGYPLKFLVFAWEGYCCIITYACSG
ISSLVLVGTMEHLIIRIEQLKLMFPEILNEANRHIREQKLKNWVQYHLALFGIGKLMTATYTY
CLSVIVLCVGILFGCIGVSTMQSASSNNSVFLFLGWFQSLIVLSVCGQRLIDTCLSVGIAVYN
SRWYDMDVSFQKSVHMILIRSQKPILIYTGPFSYLSHLLILSVLQTAYSYINLLSARG
>TcasOR80
MGHVIMNEILTYLTFLGLWPRSRKSTKTVAYLIISSTSFLFFGSLFYLIAHRKFGSNEIDSIETV
TSQFGILYYWVLFTLKREGTVEIVERLSDFSKFGKPRFFDQRNRRLNYLLSYFVLVLMVAIG
GVVALPVVYIDSCHKANERLNLTKTCGLIAPVWLPFDYNEYPRKNFVFAWEVYCCIMTYAC
CGIAALVLVGTMEHLIIRFEQLKLMFPEILDEPDRHTRQQKLKNWIEYHLTLFDIGKLMTSN
YTYCLSVIVLCVGILFGCIGVSTMQSASSHNSVFLFFGWFQSIGVLCIWGQRLLDTCLSVGIA
VYSSRWYDMDVSFQKSVLMILIRSQKPILIYAGPFSYLSHLLILSVFQTAYSYINLLGAKG
>TcasOR86
MALNQEDAICSKSCFYLRYSFLWPEEAPTRSFYAKFILVLILSFLTAFLPLFIHFLILVERGLDP
SEDLFVIISYTGFALIMIIYVIHVKKTSYLIVQLSDFEKFGKPRGFDYWDKKFRLISSGVYYYV
LIASSGLNLGRWVGMAECRKERDFQVCGIVIPYWLPWKVDSWLFFILLDLYVLKMTLVVN
CALFLIIIQILEITTHLKLRIDHLKEMLVKCFDSDSQTNRKQLVNCIRYHTYIINCSKLFKKCFT
HAMFSLIVTMALSCGCLESQVVKFDLWALPPISAWIFILFIACMAGQILMNASLSIGDAGYH
SKWYQTDANFRKYLILVLMRSHKALVLSAGPFNILCFELFVAIMKFSYSVFMLLNQN
>TcasOR87
MKHVIMDELLIFLTFLGLWPRTPTSPKIISYLMIYSTSFLFFGSSIYLILHRKFGSDEIDTIEIITS
QFGVLYYLTLLVVKRDGITKIVNLLSDFSKFGKPPLFDQRSRRLNLLLRLFVTVLLAATVAIV
SVPVVFINSCNKQNLQLNATKICGLAAPVWLPFDYTQNPRKYFVSAMEIYCATMNYAGSGS
GAFLVIGTMEHLVIRIEHLKNMFPEILNEPDKQIREKRLKKWIEYHLSIFEIGELMNETYKWP
LSVIVLCVGILFGCIGVSTMQSVSFQNSSVFLFFGWFQSIFVLCFWGQRLLDSCLSIRKAVYN
SKWHEMDVSFQKSVLMILIRSERPVLIHAGPFSYLSNLLVLGVLQTAYSYINLLNARS
>TcasOR88
MTEEKQLRICLSSCFFLKWSFMWPTKSEEFRTSKGLYFRLLAFVIISGLTFTAMIVMHLLKSV
EAGDYDISEDIAILATNTGYILMMLLYIIRQKDLESLLVDLSSFKKYQKPPKFDEVNRKLEWC
TRMVFGYCVFGSVFYNLVKILAIPSCKKSRRINEVCGVAIPYWVWFDTENWSIKLPLILHTF
LVIIIVDKVTLLVSLQVLEIACNIKLRLDQLNCMLVSCFDGDVEASRRRLNECIKYHKEIISYS
EIFSKCFSIEMFTHLTTTGIICGCLENQVVQEHRPEAILHIGGWITAIFVSSFGGQILIDSSLSVA
EAAYSSAWYEADVSLRKDLILVILRAQKALFVSTGPFNVLSFALFVSIMKMSYSILTILQ
>TcasOR89
MKEAVLQQSKKEMHLLNLWPKGHVKHFRFRYVITLIIVSPFTLGTLTHFINVLKENLDVDLS
GDISVIAVVTGLHFMLITFVWGHKKIAYLWENLGPHEYFGKPDNFEKRCKQLNFYSRLYAY
YCYLGLTVYIIMKNRGGIECRRLNVERNLTEICGLVTTFWAPFDIDFFPFRQILFVDQVFATYF
IVKGGAAISFTTLEVGEYIILKIKHLKRLVKEVFDDPREEVQRKKLVFCIKYHQYIISIQELYD

GRYKHCNGCYILMVGIIIASLSNEIMKNHNIEALLHLVGWVFSFYICCFSGQSLLSESLTIPDA
AFESKWYEAPVYMQKDLLLMMLRSQKPLMLHATPIGVMSLSLFITLVKTSYSYFTLLNQST
>TcasOR90
MAKDTSPVLRESIEVMKYLQLWPQNERTNLRRRYFIVIFLCSPLHLGLATHLVVCLKDNLDV
DLSANIAVLSAVTGLTYMLIVFVWSQDKLVHLLAKLDTHEIFGTPDNLTKRSRRLNFYAKLY
SYYCYFGIVIYSLVQIIEMPQCRKMNEEKGLSEICGMIVPFWAPFDIDWFPLKQIFWLNQLLG
IYIIIKGGAAVSITTFEVAQYICLKIKHLNRLLREAFDDPCDVVVEQKLLHCIRYQQHIIRTNEL
FNVCFKHCNGCYVVMVGIIIASLLNQILKEKSVGALVHFAGWICSFFICCHAGQAVISESLTIP
EAALDSHWYEAPVKYKKVLLLLLVRSQKAFNLQATPIGIMSFDLFIALLKTSYSYFTLLHKS
T
>TcasOR100
MSPKDKIKICGITRKVLRYSLLWPVENDELSPGIRYKLTILAFFSITGILVFSISVYSVLEIKQG
YDIDVEDVAILIAVYGTYYMVSAYLNNQHQIALLERDLSQFYKFGKPPGFEQLNSQLNFAVK
VLIIYSFLGTFVYNGTKMLLREECKKNSQEKGLSDNHCGLIATFMFPFRVDYFPVFYIVLVIT
FLLAHTLIKLCMHISFNAYEIVNHIVLRIEHLKEMILSCFNERNQTIVQKKLRVCILYHIEILD
MAARLDKNFFNTMFGHFALTGAICACLEKQIVLGVNIVAGTLHFIGWIIALFVGCVAGQCLL
NASEIIPNALWAAKWYHADLRTQKTLLFMLARSQKELTIKAGPFGILCFPLFVSVLKTSYSIL
CMLTS
>TcasOR102
MQNQSKPCQLDMMDETYLQFFVKSFTYLNMLPEKTTFCTTIQQYYVSVIITITTFPILADLVS
QFYEESISFTSVNENFVALSALFAVIYVSVCFINRKHKIRALIADLALFETFSSKAVITETDKSV
KFYTKLFIVYGIVGNLCYGLLPILGYKKCHESKSVHMTRYGIPCGLVVRFLFPFKFDYSPLA
ELVALYEILVCILGTSVVIVVTTLICGVLIHITVQLQCLRKIILDLSQVNDLEILEHKMKFCVK
YHTAILDYGIRTDLAFNQMMLLHITWTGFIISVLGFEISTTDDYVEAFRFFMHLLGWLGMLF
VVCYYGQKILDESLAIADAVYTFLWYKKSVIVQRYVLLILLRSQKPLTLRACGVKVMSLATF
LGVLYSAYSYFTLLLKLKP
>TcasOR103
MKQALKLADVLGFNPLKNDNLTKLKKYSSLICMISVVVSAILEFVSNFSALETYESAPESLV
PQFQTLAKISSLLLSQKDITELIDEIKYFWKLDQFGDFHTRKLKKIYKYVTIFFYFYTLMLSG
ACVLFTITTVIFTPEKPLFLCYGGLHGLPSPQFEIYFVVDLAAIVIMSFGVAAYDGIFFYFAFH
VYAEFKLVKVAFKGKSTFIEAVKHHDFLLKYLRKLNEIYSPIFLCQFFSNLLGICFCLFMLSRS
GMPPELTSFSKYFISLVAFTVQTYIFCLIGDLVSELSLDISNVIFYVDWLDDEVYKSKTARLVI
MNKAQSPVKLTIGKFTGMDLRTFLLIVRNAYSFLAFVNNALD
>TcasOR105
MKPALKLANVLGLDPLRNDNYTQLKKMFCALCIVSLFVSAYLEFFSNFTTFETYETAPESLI
PHFQTMFKMYSLIFSRTEIVELIQMAEQFYKFSQCDERKKLTKLYKRVDLFFYVYASLVAAA
CVLFAIVTLIFKPGKPIFLCYGGLHGLESPEFEIYLVVDLIGIVIISVTVPAFDGLFFYFALYIYT
EFKLLKIAFKTMSGQELREAVKHHDFLLKYIKKLNSVYSPIFLYQFFCNLLAICFCLFMLSRS
GIPPEMVSFSKYFLCLLAFLVQSYTFCSIGDLITELSEDVSNAIFYTDWLDDEAYENKTARLII
MSRAQNPVMLTIGKFANMNLRTFILIVRNAYSFLAFVNHALN
>TcasOR106
MESALKLIDIIGLHPLKSDKYSTMRKTISFLSLVVILISAQLEFLSHLSVFEVYNSGPHSTIPPL
QSLLKMATLHFYKNELIDLMEKSKSFWKLDKFGDLYKQELSKLHRLVTIIVYIYIALLTATCV
QLAVLTLIFRRGKPIFLCYGGLYGLESPHYEIYSILDAIGIGVISIAVSGYDAMFFFFALDIYTEF
KMIKSAFKRHSDQTVSSYNKQFIEAVKHHDFLLQYINQVNDIFSPMFLFQFFSGLLGICFSLF
MISRSGLQDINTLSIYSAGLLGFTAQSYTFCLVGEVISELSEDISNEIFYTDWLDDEVYRNKTA
ILIVMNRAQESPKLTIGKFADMNLRTFIMIVRNAYSFLAFINNALD

>TcasOR107
MENPLKLLHIIGLDPRQSDKYSTIKKVISFLIVLAVLLSALIEFFLHHNESQVYDTAPQSTVPN
LQALLKMFALIIYKKELIDLFTKGNHFWKLDKFGDCHKQKLTKLHKYVDLFFYVYAVIITG
AFLQLALLILIFEPGKPIFLCYGGLYGLESPQFEFYAVLDFLAIGVIAISVTAYDSIFFYFALYIY
TEFKMIKIAFKRENCAQFIEAVKHHDFLLQYISKVNEVFSVIFLTQFFSGLLGICFNLFMISTQ
GTRDMKSFSTYFVGLVGYTAQSFTFCLIGELISELSEDISNEIFYTDWLDDEVYRNTTARLIV
MNRAQESPKLTIGKFADMNLRTFIIILRNAYSFLAFINEVLD
>TcasOR108
MGSILLLNSVLKKMEKALKLVNILGLDPRKNDTFSKFRSIFCFTILISASFSSHLEFFLNFKGL
ETCERAAESIIPQYQTMCKMATFLLYKTEMLDLIKKSERFWKLDRFGDLQAKNLHSTYPIFQ
IFFYVYVVILFLTCAMFALVNWIFDTGKPISLCYGESEGLETPWVEFYIVLQSVEVTIIFLGIT
GYDMVFLYYAGSVCIQFQMLKMAFAERKMNERQFLKAVKHHEFLLQYVEQLGDIYSMWF
LLQYFSSLFGICFGLFLISKEGLPTEPERLSKYFPYIFSFTMQSFTFCMTGTMLSDWSSEISDEI
FHSDWSDDQVYKNKTARLIVMNRAQRPAKISIGKFLDLNLRSFILLMRSVFSFLAFVNNILN
RIN
>TcasOR109
MGKVKFTEPLEFLNVVGLNPENCSNFSLFRRVISLGFFLVVITLGLLELLLHFEGLETCSRAS
EAMIVQYQLFIKIAVLLKHRKNLVVLMQKTRKFWPLDKFGQDAKIERPHKLLKAFFFAYKLI
MILMALQYILRKFVSKNGKPLAIAFGESKGLSPKVDHLYFVLHSTSTFVVLHAVTGFDRLFF
FLIGHVLTELKLVKKSYRLTQNRREKFLETVQHHAFALEFVRKLNRIYSQVLLNQHLSCLFG
ICFGLFLVSKDGIPPDLGHVTKYVPYVISFITQTFTFCFIGSLLITWSLQVPDAIFYNDWGKNQ
AYKYKTDKIIAMIRGQRAAKLTLGGFGDLDLESFNLVVKNAFSFFTFVNAMNQK
>TcasOR110
MDKVEFSDPLFFLNVIGMHPFKADKFSKFRLAFSIAVYFAVIFSGVLELIVNSQGLETYARAS
DTLIPQCQLVCKIFVLAKYKKQIARLLNGSQRFWDLGQFGARYGNSFGKTHKYLKSFFLLY
KVMLTFTCLQFLAVKIIFKIPKPIAISFGETKGLEPLYDHLYLVLHAMITLVTINLVNGFDGLFF
YFIGHVLTELKMVKVAFGDSPIETNWSEEKRFKFAVRHHRFVLDFIEQFNIVYCTMLLVQHL
TCLFGICFGVFLMTKDGVPPDLDRASKYLPYIVTFIFQTFTFCFAGNLLLSWSLEIPNEIFYHD
WAKKTTYENKLAKIISMKRGQRAARLTLGGFANLDLDSFRMVLKNALSFFTFVNAMMNK
KAVTSV
>TcasOR111
MEKVRLTEPLFLLHIVGMSPHDSGTFARIRKIFSILVYTSTVVLSMAELFFNYKDLETVIRATE
SFFTQYGLAWKIAVFVVYKTELAQIIRLCDNLWPLDEFGTGHNFQFLHKFLRRFFLLYTGNL
ALLCTQFAVTAFFDDQFKSVMVYYGEKESRSQIYDNFVFTLQVIYLYVGCFVVAGFDCFFFY
LLGHAVTELKMLTISFSCKEIGRNWGYEERFKCSVKHHIHVLELLDKINKVYSVMLLNQHL
CSLFGICFGIFLMTKDGIPPNVDHFSKWSTYIFTFILQVWTYCFAGDQIMHWSLKIPDEIFYD
NYWNKYSLKNGLNKIIAIQRGQKAAGVSLGGFAMLDIESFNVVIKNAVNFFMFMDKMYKR
E
>TcasOR112
MITRLMAQFAIKGRVGTGGYIMDKVKLAQPLAHLNIIGLDPLKNDRFSKIRTVITVAVFALC
NVFSFSELFLHYNNPHVIVRSSEVVFPFFQNDWKIAIMLVYKKNLAQLIQNTSRFWQIDAFG
KNYQYSMGIKHKYVRIFYLVYRLMLMFSCSQYILLTIGSDRPMILSFGETGGLGSGALLFYLI
FHIVYLLIIFNVINGFDGLFFFLVAHVLSELQMVKVAFSSSKVITFWNHKRRFKSAIQHHRFV
LDYINRLNSIYSILLLNQHISCLFGICFGLYLFISDGFPPDYEHISKYVPYVIYYITQVWVFCFA
GQLIIDWSVNISDEIFYHDWTLNRTYENKTDKLIIIQRAQHAARLSLAGYGNLDLQSFNLVL
KNGLSFFTFVNAVIHK
>TcasOR113

MLDAWERLTFYPFKWISLGGLHPQNDRLVAKFLFLYNFAGFGTILGLAITQIYLSYENIYYTI
DSILTIVLYLHIASKYVNLHLHKDTLAMLIQERSKFWPIDTFEMTVRQKCVRILTKSLTIIKSY
LGYSLLVVISFLIQPIITGQLPVFMYVPRGTYYIFFVIFMTITPGIMSSIWGVDTLFFSITTPVSI
QFKLLAHKFETIDLKMDSKRVRHEFRKLVDYHNFLINYCQNINRMSSGIFLTQYLVAIATSC
MQLFITSQPEFGLLNKIKCLTYFIMQIIETGIYCFTAQLISESSENVGNAVYKAPWYDFNCGTR
RDIALVIVRSQKKVVFNGLGLVWIKMETFTKIFKTALSFYTYLNTMVYQN
>TcasOR114
MNDPSYYSLKLLKVCGLHPNSNNKLLNVYFVVNYSCFLAILTLAIIGISKTYDSNVFEAIENL
QTVFLYIHLLGKYPTLFFKKKTLKELLERQKQFWPIDNVEPKLAKKFDQILTNTTKFIRYFIIV
TFLVIMNFFLQPILTGDLPVRVYVPSGWFHYINSVYWYLIPVIIGSIYGSDLIFCSLCVPVIIQF
QLLAHKFEKFKPQQLKKLVDYHNFLIKYCNDLNKYIEPIFLNQVIVATAIICMQLFIVSQKEF
LLPNKLKCLGYCFGEVIETAIYCFNAEMISDAAEKVGIAVYNSQWYKVPRKSVVLVIAKTQ
KKVVFNGLGLVAINLKTFTQIFKTALSFYSYLNTMVAFEKN
>TcasOR115
MSNKQLDPTYYILKIFKAAGVHPDVKVTPFLVFFFWMNCTIVSAVIVLATIGAIFGALDNDIN
TVVECLQSTFIYIHILGKHFVLFYSKPILSHLLAQRTHFLQLETFDLSTIEQFQQLLKKTSKFV
NTFMFCTSCVVFSFYMQPYLTHGDLPVSVYVPDGWYYYIHFGFWPLAPCIVASIYGSDALF
CAISVPVIVQFRLLARKIQNWKIENTKLNNQKSRKIFKKNLKELVDHQNFLFEYCNEMNKF
NNGIFLNQFLLSVGIICVQLFVVSQKGFKLPNKIKCVGYSFMEIIETAIFCFNAELISDASEDV
GNAAYDSLWYESDDPEVRHAITLIIARSQNRIVFSGFGFVWINLKTFTQIFKTALSFYSYLHN
VVLN
>TcasOR116
MPETLSFYPELMLKSAGLHPYTKLKIVKFFYHHYLNLFFFIFLLFLAILEVGVSVKCDIYRAI
EALSSVLFMTLTLFRYVVNYRNKPSLAWLLEKRSNFWLLEHFEGQIRTDCAKIMHTSSNFIR
NYKNYAIVLAAVFYVQPFIFHELQMKIYVPEGWFYYLYLVYWYMTPPLFVSVYGVTSMFC
AICIPVTIQFKLLAHRIQNLDFKSEKFQRDLKHLVDYHNFLIDYCTRINRYSNGVLLFEFFITIS
VCCILIFIAANDYPFVDKIKYAGFIVSQFLDTAIFCYNCELISDASENVGKAAYDSLWYESESK
IRRSLILIIVRAQKKVTFSGYGLVRINMMTFTQVFKATLSFTSYLNTVTVDEKMLNN
>TcasOR117
MDKNLDPDDLSAYPLKFLWYGRLHPGLNPWWTKILVPVNVSVAFLYLVLAIKGIFSSYNHD
TFFTAECVQTCILVVHAIGKFSNFLVHKNSLLRLVAKKSQFWKLESFDGDLYNECVWISTFV
KKITRFYYFLNLFVLISFDLQPFTTGYLPTGCYVPEGWFNFLTGLLWYLSCAVLFGLPGTDG
FFCSLATSLIIQFKLLGYKFKNTKLYKNEPDITLWNNLKQLVDYHNYLLSYSKELDATFKTIF
LLQFMISIGSASVSVFIFMQPGDWSNRIKFLLYFVATMVQTAFYCIPLEFVVSSAKQIGDFVY
ESNWYQVKDIKFEKCFTLILARTQKNVVFSAYGLIWINLGTFLVICKTVFSFYTYLNSVNKIT
S
>TcasOR119
MLMSLEPDDISADSLKILWYTGLHPALSSRLINIYIYANLFLCSVLTILAIIGIVLSYTNNIFFVA
ECLVTIILMVHAIGKFIALHWNKKSLINLLKKKSQFWKIGSFDGEIHNECLQISTFVKNIIRFY
YALSFCGGVFFDLQPFTSGLMPSGCYVPEGWSNILMGVMWYITFPVVFVVTGTDALFCSLS
TSLIIQFKLLNHKFKTLKLTNKSQTQLWNDLKELVDYHNFLLSYCEELDATFSGVFLLQFIISI
APASVSIFIFMQPGAWANRMKFITYFLAVITETTFYCLPLDIVVNTASQVSDAIYESKWYEVD
VLHFKKCLTLVIARAQKSVRFTGFGMVYINLRTFLIIWKTVFTFYTYLNSAKKITK
>TcasOR120
MAKKFLLDISADSLRLLWLGQMHPLSPFRRFVTFLILNLAACWLMIALAIKGITISYKSDIFF
VAECLQTCNLMFHGVGKFLNLYFQKNNLKSLLENRSKFWQLDDFRSEKLYSQMQGITFVIK
KVLRYYYLLVLCVVFLFDLQPFATGLLPTGCYLPEGWFKGLTLTLWFLSVSFFLNIQGTNGF

FYSQSVSLIVQFKLLSHRFKTTQFDKKELKELVDYHNFLTSYCKQLNQAFAAIFLLQFFTSIT
SASLSIFIFMQPGAWTNRIKFILYYSYTLVETSFYCIPAEILVNAASEIGNSVYDLDWHKIRINR
VKKCIVIILARTQKTMVFTGYGLVNMNLQTFVVYVKTVFSFYTYLNSVRKI
>TcasOR121
MPKKFRSDDISADSLRLLWLGHMHPFFPFRRSFAFLIINLTACFLMIALAIKGITISYNNDIFFV
AECLQTCNLMLHGVGKFLNLYFHRNGLQALLENRSKFWKIDDFKCENIYEDLSGITSTVKR
GLRYYYCGALVVIFLFDLQPFATGLLPTGCYVPEGWFKGLTLTLWLLSISFFLIIQSTDGFFCS
LSVAIVIQFKLLSHRFKNMHLLYAESERKMWKELKGLVDYHNFLTNYCKQLNAAFAPIFLL
QFLVSIVSASVSIFIFMQPGAWSNRIKFVLYYLAIMVETSFYCVPAEIIVNAASEIGNAVSDLD
WYKIKINKVKKCFIIILARTQKTMVFTGYGLVNMNLQTFVIYVMTVFSFYTYLNSVRKI
>TcasOR71PSE
MAELKYFKGIVLILKWSLLYPLVKSWPKKCLYYVWALLYVLSFILTFVQCSMYLYKTPFNLI
EEAMIIMNTADXIYFSLFINFTFLYTKRTKLSRFVKDFNQKCLDQTDNKIEKITMEKSSKLSD
KFVFYWTFNCIASSLMPMIASLLGGNKNLPMVVWYPYDPNKTPYFHLTYIWEIFCISNLGLI
YAVLDLVFPCIAIVLGQQFKILASNFKNNVYRALVDSEVSEKIVQTFSKNLHNDSFNEEIFEI
MNSAKFKKNNARYLRKNVKHHQQLLQYCADVSDILSIFLMGKVSAAIFNTLFMAFSLITNA
DIVGTLYESPWYMCDVHFQRTFHIVQMRASKIVNVKAGNYFTMSASSYITFMXNRSGSYIA
LLKELTDRGK
>TcasOR72
MAKLEYLTGATFTLKCAVLYPIDSNNPKIKKILYAVWAIFFILTFVTGFIQCFVFVCINPFDLVQ
EAMIIMSLVFYSTTFFYFIVFYKNWQNMVALVTNINKNFHRATDNVIEKISMDQASELSDKL
AYVWTSSLAVGSVVPVVLAIATGNLEMPMPAWFPYDYNKSPVFEITYLWQVFCLITLAIIYG
ASDMFFPCITIIIGQQFKILASNFKNNFYTSLIKLGAEESIVQNFSKDIKTHEFRSFYIKYGNIFK
ILNNAKFQTLNRAFLKRNIKHHKLLLRFCEDLNKILNTFLLIRVSAIVFNLIFIGFNIIINADFL
WTLYECPWYLCDVTYQKMLILVQMRVKRMVSTKAGNFFTMIAPSFIAFQRAVFSYITLLKE
VTDLGKD
>TcasOR167
MAKTGDIFPVRDPVKRCLFIPKLLLESTNFWPEKRNFLTKFANWVMLIICVLIESGQIAFVVV
NIKDITKIASAMSTVSTTFQAITKLTVLYIYNDKLRLILKSVWYEFWPSYTAGREINTKLETY
NKIVIVSFLTILISGICFAFGFLSSPLISGERILPFETVYPFDWTKSPYYEIIYVTEWMTNIAFILI
GICGHDFLFMGLCSNVVGQFTLLRELFGYLGTKNVAQIIKKLGHDTNIEPNRQLLRICIIHHV
RVTEICKEIAEIFSFSCFIQLLSSVTALCVGALIMTFADIDAALFTVSSAYIVGHLLQLFLYATL
GNEVIYYASRLPNAIFHSHWYNIDLEVKKDILFVLQRAQKEVKISAMGVSVLDYQTFIQVLR
LSFSFYTMLSKVTDH
>TcasOR168PSE
AATKLIVLYIYNDKLRLILKSVWYEFWPSYTAGREINTKLETYNKIVIVSFLTILISGICFAFGF
LSSPLISGERILPFETVYPFDWTKSPYYEIIYVTEWMTNIAFILIGICGHDFLFMGLCSNVVGQ
FTLLRELFGYLGTKNVAQIIKKLGHDTNIESNRQLLRICIIHHVRVTEICKKIAEIFSFSCFIQLL
SSVTALCVGALIMTFADIDAALFTVSSAYIIGHLLQLFLYATLGNEVIYYASRLPNAIFXSHWY
NIDLEVKKDILFVLQRAQKEVKISAMGVSVLDYQTFIVLRLSFSFYTMLSKVTDH
>TcasOR169PSE
AVTKLAVLYIYNDKLRLILKSVCYKFPSYTAGREINTKLETYNKIVIVSFLTILISGICFAFGFLS
SPLISGERILPFETVYPFDWTKSPYYEIIYVTEWMTNIAFILIGICGHDFLFMGLCSNVVGQFT
LLRELFGYLGTKNVAQIIKKLGHDTKIEPNRQLLRICIIHHVRVTEICKEIAEIFSFSCFIQLLSS
VTALCVGALIMTFADIDAALFTVSSAYIIGHLLQLFLYATLGNEVIYYASRLPNAIFHSHWYNI
DLEVKKDILFVLQRAQKDVKISAMGVSVLDYQTFIQVLRLSFSFYTMLSKVTDH
>TcasOR170PSE

TVTKLAVLYIYNDKLRLILKSVWYEFWPSYTAGREINTKLETYNKIVIVSFLTILISGICFAFGF
LSSPLISGERILPFETVYPFDWTKSPYYKIIYVTEITNIAFILIGICGHDFLFMGFCSNAVDQFTL
LREHFGYLGTKNVDQIIKKLGHDTNIEPNRQLLRIFIIHHVRVTEICKEIAEIFSFSCFVQLLSS
VTALSVDALIMTFXADIDAALFTVSSAYIFGHLLQLLLXHSHWYNIDLEVKKDIXFVLRAQK
DVEILAMGVSVLDYQTFIQVLRLSFSFYTMLSKVTDH
>TcasOR199
MSMTRSKYFQDSDDPFSFIRKIFIDYGYSKKINYYNRVTFTFNTCSILLESYYMITNFSLDLFV
RYGGALSLMLYHVVTQFLVIAKQKSLEQLLEESKSYFWKADIFNSSVKNQILKSCNHMQRK
FCLLWTPFVACGIVLLPVWGDFTESHIFPQVYKAYFGHWSPIFYYFCISSYPFAVYTSIRLPAI
ALYLFLQAHFQIVLLNQKILQISKNNDLDETTIFENMEYQKTIYRNLRSCISQHVALQKYITRI
LVSIQKAIPVYFCLAVLCLIAVIFFVLNNLNMSASNHFKARIFVSGVCGSLILYTFTEAGQLLA
DTTGDIFNTLMQCPWYYWNIKNRTVFMIFMLHSLNPLKIDWGGFTLGYSFGGAVIRTCCSY
AVGLYNLRESKY
>TcasOR200FIX
MSIQNTKKTLNGKEKHPDFIKFCFTDSNDSFLILKRVYIDFCYHKITKTCNFLLVISQLFFYLI
QIHFLLSRFSLELLARYSTIMMITTVALFGLILSFYLEEDIHELYKILTEIAWPLDKASKKDQQ
DLRQKSRRINSLNLYFLGFLAFMIVIFLPIFGDEENLFLCIQVFDEYFGDRAFIFYNLYFIGFPF
LIYFSVQLCFMFLYAILHLHVQINLINHHICEMGASFELLSDWKKLHSVVYQSAISQLLCQCI
RQDIALKRFIVKLNETVQLGLPFFLPVSGLCGISVIFFLLNYMCTMSLVLWLRVSAFFICLVFV
ALIFSVSGQLLIDETGKIFDTLVKCPWHIWNVRNRKIYLICLTHCVRPNCISYAGITLNRIFLIT
VFYKTVSNAFILYQVRNS
>TcasOR201PSE
MVEFKDPFIMLKTIFLLNVKEMTKFSQVFLPIFAFHSLVXYLYKNFDVNLLIRYAPMTTGTLF
VSNTKLLSSSLNIIPVFSVVSETKLLRIATFIDKTCWSLDTIRKEARIKLERKCRATNISISCILL
LLSVAVFSNFPCFGKQDDFFLCIKIFEEYFGQWSSIPNYIYFTLFPILCYSYFRIVFSFVYAILET
QLQFSLIEEYLFEVYQMDDLNWKYLQDPRYQQEIGKSLQLCIEHHTALKKLIHSIMNVTLTA
IPFFLPFVILLFVSCFLFFINFGDTMTIILKIRLLFYVGAILSMTLLMCWYGQQVINVTNSIFFT
LVGAPFYFWNLNNMKILLMFIMNCTKNESIVLTGIYVDYTLAVSVLRISVSYTLSLLGLRNR
SFD
>TcasOR202
MVEFKDPVIMLKTIFLVNVKEMTKFSQVFLAIFTFYSLVHCVQMYYLYKNFDVNLLIKYAP
ATTATLFVSNTKLLSSSLNIMPIFSVVSETKLLRITTFIDKTFWPLDSIRKEARIKLERKCRAINI
SIYCILLLLSVAVFSNFPCFGRQDDFFLCIKIFKEYFGQWSSIPNYIYFTLFPIFCYPYFRIAFSF
VYAILETQLQFSLIEEYLFEVYQMVDLNWKYLQDPRYQQEIGKSLQLCIEHHTALKKLIHSI
VNITLTGMPIFLLFGIGLFVSCFVFIINFGDTMTMILKLKTPLLLYVATMLSMTLLMCWNGQQ
VIDVTSRIFYTLVRAPFYFWNLYNMKVLLMFITNCTRNENIVLAGICLDYTLSVSILRISVFY
TLGLLELRNHSFD
>TcasOR203PSE
TSHIFDSLMLCSYINVKNRRVLLIFLANSLKSMTYSLARITINCRFALGFMRTCLSYAIIIYNFN
NEAQITRCSSELFLLKINVSSKMGNQNSHYLKKDHHSSSVPKLYV
>TcasOR204FIX
MTNFFSNFCSPLKNHWAKTKHLFSKFSLSSDQPFIMIKLVCVDIGYHPVAKTINYICLAIHISS
FLLEMNYLRLNFSTDLLIKYGCGISAVVYDISTLIVAPMIERPTIGLSEGITTSFWPIDFCGPKV
KQLILEDTKKTSKIYYRTLVTIFGFAAVIMLPIWGDQKEWFLCVQVYEHYFGKWAQIPYHIY
FLSFMWFAFTSVRLPLMMSYAIKNIRVQVFLVNQKIAKMSKEYEEAKIEDVNYQNRVYKNL
RLCISHHVLLKWWLRKLQKIVRFCLPVFVVIGILTESSVVFYLIYNFKKVNLLLKIRFLLLAC

TTGVIIYFFSEAGQSLYIETSQVFDSLISCPWYSWNVKNRKVLLIFLTNSLQPMFFSLVGFTID
YRFALTMIRTSFSYAIILYNLSSGSQIASI
>TcasOR205
MTNIFSNFSLYFKNTWTKTKQRFSKTLPSSNVPFMMIKLVFVDIGYHPVSKIINYICLAIYMS
SFLLEMNFLRLRFSTHLLIKYGCGSSLSVYFISSMTVAAMTELLAVDLSEGILSSFWPIDFCGP
QVKQLILKQSRADKRMHYVVLLVFSITGLAMLPIWGDQKEWFLCVQVYEYNFGEWSKIPY
YIYFFTFPWVAFSSLRLPFMMNYAILNLRMQVFLINQKIAKMSNAYDQTTIEDVNSQKRIFK
NLRLCISHHILIKWWLRKFVNHVKFCIPIFVIVGIATSISIVFYLIYSFQQVNLVLKIRFLSIACC
CWFVIYLFSEAGQSLYEYTEIFHSLISCRWYIWNVKNRRILLVFLANSLEPMTFSLAGITLNY
RFALNMMKTSCSYALILYKLNCDSQIMD
>TcasOR206FIX
MWNNNPFIVIRTIFLDINNYKIVKFCYVSLTVFYSLVHCLQFYYIIKNFNLNLIIRYGFITSLLS
YVLAAGILSLVVEKRIRKTQIFFDEIGWSLNIVGKDAEMKLEKKCKLINISIYAIMLLLIITLLV
NLPFVGSQRDLFLSIQVFEEYFGKWSEILDRLYFALAPFLSYHGARLSFTCIYAIMQVQVQFS
LIGEYLFETYQVDDSKSWKYLQDTRYQHDIGESLRLCVEHHVALKKSIKMMVDIALTCLPF
LVLLGLCTLISCLAFIMNFWDTMDNILKLRIFMWAAWIVLITIMFCRSGQQLIDATSDIFFTLG
GAPWYYWNLDNIKILLTFMANSTKNDSISLAGICLDYPLFVSVANTTVSYALVLYNLRESSL
DSSNKK
>TcasOR207PSE
MVEFNGPFIMLRTILFIDMNTYKILKLFNVLLNVIYSLIHCLLVYHMFKNLNINLIIRYGPAVL
FLILVIVGAVFSVYLEKDILEIVTLFRKTRWSLSMIKKDARIKLEKKCKIINIFILFLVLLIISTITI
NAPYFGDQRELFICIQVFEEYFGEWFFIPYNFFFVAFPFLYYNFFKLWMTFVYGILEAQLQFFI
LEEYLCGTFETDFRKNWEYLQDTRYQQEIGTSLRLCIAHHINLKKLIKMIQNVTLMVMPFFL
VLGVLILISSFSFIINFADTMTTIAKIRMVISAISMVGITILLSWIGQQVIDVTSDIFVTLGGASW
YYWNQKKYXKTLLMFLTNSIKNESIVLAGICADYGLCVALLRLSVSYALVLFNLRKSTLV
>TcasOR208
MEKLDDPFITLRKMVFIEAKNCKIARFCDVLLIVLYSLAQCLHLYYMCQNFNLNLLIRYGPI
LISCLLVIVTAVISVGLDKEIFEVYTVCWKISWPLNFLRKDAQTKLRRKCQIINRGILCSALLF
LTTVISTFPCFGSVRDFFICVEVYEKYFGEWSFIPYYFYFAAAPFLYYHFFRVCYVFAYAFLH
AQLQYFLIEEYLLETYQTNDLKGWKYLQDTRYQQEIGKSLLLCITHHIALKKYVKISQNLVL
IGMPFFLVLGVLLLINSFGFITNFGDTMSNILKIRILIFVACGVSITIVMCWIGQQLIDVTSEIFV
TLGGAPWYFWNRDNNNILLMFLTNCTKNESFILAGICVNYQLFFSIVRLTVSYTLVLYNLRE
SGFI
>TcasOR209
MMPEFSDPFIMLRKMIFIKNHKIAKFCDFLIIAIYSSAFCLQIYYLCKNFSISLLIQYSPTLLCYI
FVIDAAVLFFYVEKNILEAITYYDEIGWSLSMIPKDAQTKLRKKCLIINICVSFILLLILSTLTIN
LPYFGSQRELFICIQIYEEYFGNWAFVPHHFYFGVFPFIYYNSVKMWISFVYTILEAQLQFILV
EEYLLESNIINDFKGWKHLHDIRYQQEIGKSLRLCITQHIALKKLVKMIVNITMAAMPSFLVL
GVLLLISSFAFILNFADTMTNILKIRVLMFVACIVCITTLLCWTGQQVIETTSDLFDSLVGAPW
YLWNRENIQIFLMFLVNCTKNESLVLAGICLDYRLFVSMLRISVSYALVLFNLRKSSIT
>TcasOR210PSE
RYIFSKHFIQHLYFTAALFLYYHFFRVCYSSVYALLHAQLQYLLIEEYLFETYQTDDLKGWR
YLQDTRYQQKIGKSLRLCITHHIAIKKFMKMTLDLVLIGMPFFLILGVLLLISSFAFIINFAHN
MSTILKIRILFFAVSAVAITMTFCWTGQHLINVTRKFLVLGWSSFYFWNRETPKFF
>TcasOR211
MTNKLSYAPFTLLRKLIFIESKHCKLARFCDFLLIVLYSLAQCLNMYYMYQHFNLSLVIRYG
PVLVFSLLVIVTSVISVAWEKEIFELHMVNRKIFWPLNCVGKNAQTKLTRKCQFINHWISCSL

LLFLITVIINFPCFGSQRDFFICVEVFEKYFGEWSFIPYYLYFAASPFLYYHFFSSSFLFVYTILD
AQVQYFLIGAYLFETFQTDDLKGWKYLQDAHYQQGIGKSLRLCIENHIALKKFMKMSLDF
VLIGIPFFLVFGVLLLISSFAFITNFADTMSNILKIRMLIFATSSVCITMVLCWTGQQLINLTSEIF
LSLGGAPWYFWNRDNRKILLMFLTNCTKNESVVLAGICINYAFFLSLVRLTVTYTLVLYKLH
RSGIV
>TcasOR212
MAGFKDPFIVMRTIFLDIINYKIVKVCYVFLFMIYLLVHCLQLYYMIKNFDLNLLIKYGFMTA
LFSYILVVAVLGLVVEEKIRKTLKILDGVGWSLNIVGKDAEMKLEKKCKMINISVYAIMLFLI
ITLLVNLPVFGSQRELFISIQVIEEYFGKWSEMLNRLYFTFAIFLSYHGVRLSFACIYGILEVQL
QFNIIEEYLCEIYETDSSKSWQYLQDTGYQRKTGKSLRLCIEHHVALKKVIEMMLEISVICLP
FLAVVGLANLISCLTFIMNFWDTMDNILKLRIFMWAGWIVLITVLFCRSGQQLIDVTSNIFFT
LGGAPWYYWNLENIKILLTFMTNCTNNDSIALAGFCLNYPQFVSIANTTISYALVLYNIRKSS
DSDY
>TcasOR213
MAKFNDPFKFVRTIIFVDMNSYKVIKTCNVLLNIIYSLIHCLLIYYLCKNLEINLLIRYAPAILL
FILVIFGAVFSIYMDEDILEVRSVFRENRWSLSVLKENSQTKLGRKCQFINIFILLVLLLIVSTL
AINAPCFGNQRELLICIQVFEEYFGEWSFIPYYFFFLGFPLLYYNFFRLWMTFVYGLLEGQLQ
FFILEEYLCGIYETEDSKSWKYLQDSRYQQEIEKSLRLCISHHIGLKKFLKMVENQTLKVMP
FYLVFGVLILICYFSFIINFADTVTTIGKIRMFMTAICMMGVAILLSWIGQQLIDVTSDIYFTLG
GAPWYYWSQKNAKLLLMFLTNCTKNESVTLAGISLDFTLFVSIVHTTLSYALVLYNLRESSL
VSSSQK
>TcasOR214
MAEFIDPFLMLRALVSVKFNDYTSLKLCNILLITIYSLIHCLLIHYMFKNLDINLAVRYVPMI
MFLTLVIVGAIFSVAIEKDILEAQVFLFKANWSLEMIRKDAQLKLERKCRIINICILCVLLLIFA
TITINAPLFGSQRELFICIQVFEEYFGKWSFIPYYFYFAAFPFLYYDFLKLWMSFVYAVLEVQL
QLTLVEEYLFETYQINSSKEWKNLQDTHYQQQIKKSLRLCITHHIALKKFVKMTVDLTIKVM
PFYLTIGVLILISFFSFIINFADSMSNILKIRIFMFSASIVSITVLLSWIGQQLVDVTSGIFWSLVG
APWYFWNLENVKTLLIFLMNCTKNESIVLAGICIDYSLGISILRLSVSYALGLFNLRKSSLD
>TcasOR215
MAEFIDPFRMLRALVSVKFNDYTSLKLCNILLITIYFLIHCLLIHYMFKNLDINLVVRYVPTIM
FLTLVIVGAILSVAIEEDILEAQVFLFKANWSLEMIRKDAQLKLERKCRIINICILCVLLLIFATI
TINAPFFGSQRELFICIQVFEEYFGKWSFIPYYFYFAAFPFLYYDFLKLWMSFVYAVLEVQLQ
LTLVEEYLFETYQINSLKEWKNLQDTHYQQQIKKSLRLCITHHIALKKFVKMTVDLTIKVMP
FYLTIGVLILISFFSFIINFADSMSNILKIRIFMFSASIVCITVLLSWIGQQLVDVTSGIFWSLVGA
PWYFWNLENVKTLLIFLMNCTKNESIVLAGICIDYSLGISILRLSVSYALGLFNLRKSSLD
>TcasOR216
MTKFNDAFPMIRAIVSLNFNANTSFKLCNIMLITIYSLIHCLLIHYMFKNFDINLVVRYTPTIM
FITLVIVGAIFSVAMEKDILEAYAILPKANWALEMIKEDAQLKLERKCRIMNICILCVLLLILST
ITINAPFFGSQRELFICIQVFEEYFSKWSFILYHFYFIAFPFLYYGLLRLWMGFVYAVLEVQLQ
LTLVEEYLFETYQINSLKEWKNLQDTHYQQQIRKSLRLCITHHIALKKFVKMIVDLTIKVMP
FYLTIGVLILISFFSFIINFADSMSNILKIRIFMFSASIVCITVLLSWIGQQLVDVTSGIFWPLVGA
PWYFWNLENVKTLLIFLMNCTKNESIVLAGICIDYSLGISVLRLSVSYALGLYNLRKSLD
>TcasOR217
MVEFKDPFIVLRKIFFIKFNNCKLTLFLNISIIVFFSLVLCLQICYLMKNFNLNLLFRYGPVTVL
FTLVTVTAVLSLTLEREIFMAITFFFKFCWSLNIIRNDAQITLKRKCRCVNIGLLCILLIILIAIVI
GFPCFGSQKDFFICLEVFEEYFGEWSFIPYYFYFAASPFLCYHFLRICFTFVYAILEAQLQYLII
AEYLFEIYQTNPSKRWKYLQDTRYQQQIGKSLRLSIVHHVVLKKFLKRTLHLTKIGMPFFLV

LGILLLTSSFAFIMNLGDTMSNILKIRIFLFTTSVLCITILLCWIGQQLIDVTSQIFVSLSGAPWY
FWNLENIKILLMFLTNCTKNESIILAGICLDYKLFVSVARLTVSYAVVLFKLHKSSLV
>TcasOR218
MMDKLNYAPFITLRKIIFIEAKNCKLARFCDVLLIVLYSLAQCLNMYYMYQHFNLSLVIRYG
PVLVLSLLVIVTSVISVALEKEIFELHMVFRKIFWPLNCVGKNAQTKLTRKCQITNCWISCSL
LLFLVTVISSFPCFGSQREFFICIEVFEKYFGEWSFIPYYFYFAASPFLYYYFFRICYLFVYAILD
AQVQYFLIEEYLFETFQTDDLKGWKYLQDAHYQQGIGKSLRLCIENHTALKKFMKMTLKF
VLIGMSFFLVLGVLLLVSSFAFITNFADTMSNILKMRMLIFATSTVCITMVLCWTGQQLINVS
SEIFLSLGGAPWYFWNRDNSKILLMFLTNCMICLNYELFLALVQLTVSYTLVLYNLHKSGIA
>TcasOR219
MNKFHDPFIMLRKLIFIEARNCKLARFCEILLIVLYSLAHCLQIYYMYQHFNLNLLITCGPIM
GTVLLTIVTAVMSVGLEKKIFEALTVLSNISWPLNFLKKDSHTKLTRKCQVIKWCISCSVLLS
VITLISTFPCFGSQRDFFLYVEVFEEYFGEWSFIPYYFYFAASPFLCYHFLRVTFVFVYAFLHV
QLQYLLIEEFLFETYRTDDLKGWRYLQDIRYQQKIGRSLRLCITHHVALKMFVKKTVDLVM
MAMPFFLVLGVLLLISVFTFIINFAVTMSIISKIRILLFAATGVCITMTFCWSGQQLINVTDEIF
WVLARAPFYFWNRENSVILLTLLTNCTNNDSVVLAGICLDYKLFLSVVKLAVSYSLVLFKL
RKSSLV
>TcasOR220
MADSKDPFIMLRRIFIDVNSYKITKLCDVSVVTFHSLVLCLQLYYMIANFDVNVLIINGPPTI
VFLLMTVSAVLSEAMAKDIFKGITFFQQIRWSLDVIEKNARIKLERKCQTINICITCILLFLSTT
MVINMPFLGNPRQFFISIQIFEEYFGKWSVLLNVLYFTGLPYLGYHAVKLCFAFVYAILEIEL
QFSLIEEYLFQMYEVDYLKSCKYLQDARYQQEIGNSLRRCIIHHIALKKMVKMLVEVVLKC
MPFYLVLGVLLLITCFAFIINFADTTTSTIKIQIFMFVASTLCITVLFCWNGQQLKDVTSNIFFT
LGGAPWYFWNLENIKILLMFITNCTKNDSIVLAGICLDYKMFVSVLRTSVSYALVLFNLRKR
SLV
>TcasOR221
MAEFKDPFIMLRTIVFINMNSYKVLKVCNILLIVLYSLIHCLLIYYMFKNVNINLVIRYSPAML
LSILAIIGAVFSVFKEKDVLEIDKVFHKTRWSLNMITEDAQNMLKRKCLIVNACILFLLLVIV
AMFIINAPCFGNQREIFICIQVFEEYFGKWSSIPYYFYFFGYPFLYYNFFKGWMAFVYAILET
QLQFLMLEAYLCEIYHTDNVKNWKYLHDTHYQEEIGKSLRLCIAHHNVLKKMFKMVVNIT
DTAMPFFLLLGSLILISAFAFIINFVDTMTTILKIRIFISAIVMVSITMLLSWIGQQLINVTSDIFF
TLGGAPWYYWNLKNVKILLIFLTNCTKNESIILAGICLDYQMCISLFQLAVSYALVLLNLRKS
SLV
>TcasOR222
MDKRDDPFIILRKMIFIEAKNCKIAKFCDAFLILFYSLVQLLDIYYMSKNFSISLLIRYSPITIM
YLLIIIAAVISVGLDKEIIEAYTVCCKIRWPMNVVKKQTQIKLKKKCQIINAGLSCTVPLFLVTI
ISTFPYFGSERDLFICVEVFEAYFGEWSFIPYYFCFAASPFFYYHFFRITFVLVYAFLHAQLQY
LLIEEYLFETYETDEAKGWKYLQDTRYQQEIGKSLQLCISQHIALKQFVKKTVDLVLIGMPF
FLVFGVLLLTSLLAFITNFEDITSNILKIRILLAAGCSLCITIVFCWIGQQLINVTSDIFFSLGGAS
WYFWNRDNMKTLLMFLINCTENESVVFAGICLNYELFLSVVRLTVSYTLVLYNLQKQ
>TcasOR223
MTDFKDPFIMLRRIFIDVNSYKITKLCDFTVITFHSLVLCLQLYYMIRHFDVNLSIKYGPITAF
FLFMTVSAVLSGALSQDIFRAVAFFEKISWSLDVIRKEARIKLERKCQVINTCISCILLFSSTTM
VINLPFCENQRYFFISNQVFEEYFGKWSVLLNVFYYSGVPYLGYHSVKPCFVFVYAILEIQL
QFSLIEEYLLQTYETDYLESGEHLEDTQYQREIGEALRRCITHHVQLKKLIDMMVDIVLMY
MPFFLVLGVFLLITCFAFIINFADTTTNTVKVQAFMFVVTALCNTVLFCWNGQQLIDVTNSIF

LTLGGAPWYHWNVENIKILLMFITNCTKNDSIVLAGICLDYKMFVSVFRISVSYALVLFNLR
KRSLV
>TcasOR224FIX
MTEAGDPFIMLRWILLMDVSNNKITKYCNVFLTTIYSVVLCLQIYYIFKNYDTNLLIKYGPIT
ISLLFMITVAVISLIMQKEIFKTVTFIRETCWPLNIIQKSGQIKAERKCRTINFYITSTFLLFLSAII
IHYPCFGSQRDFIICIEMFEEYFGEWSSVLYYLYLIGVHFLYYRLFQTCYMFVYGMLEAHLQ
FFFLGEYLLETYQTDCLKRCKYLQDTRYQQEIGQSLRFCIKHHIALKKLVKMALNLAFIGMP
FFLVFGVLLLISCFTFIINFADTMSTILKIRIFMFASNTVCIAILLCWIGQQLIDVTSDIFVTLCG
APWYNWNLDNIKLLLMFIMNCTKNESIVLAGIRADYQLFVSLLRISASYALVLLKLRKCSLV
>TcasOR225
MKTSGDPFIILRWILLMDVSNNKITKYCNIFLTIIYSLVLCLQIYYMFKNDDINLLIKYGPITIL
LLFMITVAVISLIMQKEFKTVTFIRETCWPLNMIKNNGQIKAERKFRTISFYTLCTVLLYLSVII
INYPCFGSQRDFIVCIEMFEEYFGEWSSVLYYLYLIGAHFLYYRLFQTCYMFVYGMLEAHLQ
FFLIGEYLLGTYQTDCLKRCKYLQDIRYQQEIGKSLRFCIKHHIALKKLVKMVVDLAVIGMP
FFLVLGVLLLISCFTFIINFADTMSNILKIRIFMFVSSSVCNSILLCWIGQQLIDVTSDIFFTLGG
APWYNWNLDNIKLLLIFIMNCTKNESIVLAGIRADFQLFVSLLRVSASYALVLLKLRKCSFV
>TcasOR226
MAKTGDPFIMLRWILLMDVSNNKITKYCNIFLTTVYSLVLCLQIYYILKNYDINLLIKYGPIT
TLLLFMITVAVISLLLQKEIFETDTFIRETCWPLNIIQKSGQIKAERKCRTINFYIGSTFLLFLSV
LILNYPCFGSQRDFFICIEMFEEYFGEWSSVFYYLYFIGSHFLYYRLFQTSYLFVYGMLEANL
QFFLIEEYLLQTYQTDCLKRCKYLQDTRYQQEIGKSLRFCIKHHIALKKLVKMVVNLGVLA
MPFFLVFGVLLLISCFTFITNFADTMSNILKIRIFMFVSSTVAIALLLCWIGQQLIDVTSDIFFTL
GGAPWYNWNLDNIKLLLMFITNCTKNESMVLAGIRADYDLFVSLLRISASYALVLLKLRKC
TFV
>TcasOR227
MPESIKLFSEESSTFRTMAEFHDPFIMMRKILYNKSKNYNLAKLCHVLLIIIYSSVQCLQIYY
VFKNFSINLLLRYAPTILFFIFITGAVLSLIFMENDILEIVTFLDKICWSFNMVRKDAQMKLKR
KCQVINMCILFVLLLLLSTLTINAPCFGSQREVFIFIQIFEEYFGEWSFIPYYFYFTAFPFLYYN
FLKLWMSFVYMVLEAQLQFILVEEFLFETNQVNRLKGWKYLHDTGYQKKIEKSLRLCIIHH
NALKKYVRMTLNVTLKAMPFFLILGILLLISMFAFLINFADTITTMSNILKMRIIMTVTVTMC
IAALLCWLGQQLIDTTSDIFASLVGAPWYFWNLGNIRILLMFLTNCTKNESVVLAGICVDYK
LLVSMLRTSVSYALVLFNLRKSSVV
>TcasOR228
MTDSRDPFIMLRRIFIDINSYKITKLCDFFVVTFHSLVLCLQLYYTIRNFDVNFLSRYGQTTVI
FLFMTVSAFLSGVLEKDIYRILTFSKTFLWSLDVIGKDARMKLERKCQMINMCITCLLLFLST
ALVINMPFLGNPRQFFISIHVFEEYFGEWSSLLNVLYFASMPYLAYHATKPCFLFVYATLHMQ
LQFSLIEEYLFQVYEIDYLKSWKYLQDVRYQREIGNALRRCIIHHIALKKLIKIIVDIVLVYMP
FILILGVLLLITCFAFIINYANTTTNTIKIQMFMFMASALLIAILFCWNGQQLIDVTSNIFFTLG
GAPWYYWNRENIRILLMSIMNCTKNESIVLAGICLDYKMFVSIFRTSVSYALVLFNLRKRSL
V
>TcasOR229
MSARPLHLRNFPYYFLKVLVFDFEQYSAGKVLSYFCAIVHSISIFLQMHYLVKNFTKETMFQ
YGCVLTVLTYCVVALFFAIASGNFVEKLESEISSFVWPLDICGEDVKAAILKRAFYTSLVAYIT
IIAFPIFSVIMFPVLGDQSDMFLCVRVFNEYFTKWSQIPISLYFYSFPVIAFSGIRLPGMLLYAIL
ITHIQMFLLNRRIEQISELSNQRRVFETLCSCIELQAKLKRLIRNVFQLVYIAMPIFILLGAVSS
VFVLFFVVNSLETASYFLVLRMGCFFGANVLVVFIFSQSGQSFSDETGRIFDTLVMCSWYNW
DKRNKKVLLMFLANSLEPMSITIAGITLDYKFALAMLRTSCSYALVLYQMKN

>TcasOR230
MREKPLHLSHFPYYLLKIMLCDTEQYRLGRFLSYSCAVIHSISLLLQMYYLIDNFNKETVSR
YGCVVIVTTYCVVALIYEILYAQPSVSMMSQQISTLWPMDACGEKVKQMILKRAFFTSVVT
YSILFSFPIFGIIMFPLWGDQSDMFLCVRVFNEYFTKWSKVPIYLYFCSFPVLTFSGIRLPGML
LYAILITNIQIILLNQKIAHISDLGDQRLVFGTLCSCVSLQIKLRQMLNKVLQFVYLVMPVFLL
LGALTAISVLFFLFYSLENPSDYLMIRLACFLGGNILVVFTFCESGQALSNDTGRIFDILLTCP
WYKWDKKNKNILLMFLVNSLKPMSITIAGITLDYKLAVTLIRTCCSYALVLYQMKN
>TcasOR275FIX
MVHFPSQKRPLKDDPFQYLRRCLEPWGQKPSLLPLCLLILLIKIFFFTARTVFILKWMKEIDR
FGNFSHMACTTFGKRNRKFWDLGVGGPGLEKELEKRFKLINRFLFGHVSLGVFYVGLYAFF
ADVPIPKGRTRWLPVLASMPFDQDQSPQYEILYVLMYWNLVVSILGHGVFDMVFIYSSQHL
VGQFILLKALLRKLDYGFEGLEIVAKARSRQFQKEIRKRIAICVQHHNLLLAYGNELKKIAS
MIFGVHVLSTSLTLILVGYILSKNLEKILQYSMLLSGVVSEALQFIIFAVQSGEIYHKSVSVAQ
AAYQSNWYVFNAKAKRDLTLLILNSQKGISMYGAGLVTINNEILVSMIQKIFSSITLLRSLGE
QK
>McarOR2
MKRNMSYDNFDYTVFFTHNILMYKIFGFWRPDDDMKREKLYNCYTLICTIIWLLFLASQYI
FIITNIQNVDEVTATSFVTITFSINLIKMLAIYRNMNRIKQLIKDMNLPMFQAKCARHRDIIDY
TRIYTIFFYICLYFGNTDRHYFWTIVPFIGDERATLTHGWFPYNETKSVNYEITYVFQTTVSV
WNTMLCLNLDTFTGSLLILIGLQCDLLCVTLENLGDFHVENGVLCENSEEYQSSLVNDKVK
FSKTMTENLVVCIKHHKEIMRVSKDVEDIHRVSVFILFLGGALIMCCCLFQLSVVPIGSIEFF
MLLFFLISILTEQFIYCWFGNEVIQKSSRILHSAYCTPWLDCDINFQKVLLQLMTQTYRPITLK
AGGLFTISISVYISVIRTSYSYFTLLKK
>McarOR3
MSQKVDPQYFKKHLKWLTWLGIDIIPIEKVWYAIPYKLYAFVLLVYVYLYSLLEIIDIVKSSD
FNSMTFGLSYSVTHILGAAKITILILKKKILRDMLIRLEQGYFVPNKARGGEKEQQLVNASVI
RANLHADIFNTLVYLIIGIRCLYAIFDKGVYVEVLDEKLNVTTLKHIRTLPYKAWLPVDLNKS
PAYEFMFIIQASCLVLYGYYIGFLDSLIYGMMIHMNNQYLILRNILEHYVELAKNIVLNRNPN
SVTDDTSTDYIKLHNGIERQKTLAGPVLDVIENIAYHCAKYHLAIIDYCDDIEKEFSNLMLL
QFLSSLYILCFQLFQLSLVTNYFSFDCISMCLYLILMMYQLFCYCWYGNAVMIQSLDISSVIY
NTDWLVTNESTKKCLLLMMMRAQRPIIFTAGKFAFLSLPTYMAIVRGSASYFMVLQQMQ

>McarOR4
MAPSYVFDLPKAFEFEKKLLLYTGLYPNTGLVNKYIYYLSGLFHIGITILIEISLIIVISIHIDNLS
TITDALMFFVTQIALTWKLTNVCIKRKVFCEIEEILSQPIFYNLSQECENIIHYYVKFSHRFAR
CFRIICIMVCATNGTLPLVGGKLGHAMLLGWNPWDSEDRIKYYLNSTFQLTALCVSACINST
IDILTVILLAIATAQIEILKNNLVNIKYGEKEAKKLFNENVRLHYEILRFVNAVDRSLSSGILSQ
IFGSVLVICVTCFQLIIVSVQSIQGAFLLIYLLCMTFQVGLYCWFGHYLIDSSDTIIQAVYMSD
WYEANNSLRKAVIIFMERCKQPIVLRIGGLFPLSLGTFTSIMRSSYSYFAVLRKWYEPE

>McarOR5
MTDKGYTPHFFRTNEIIEVYTGAWMYNENLVAPGKKWLLYIWSVLIYIGAVFFLFLEFLKLR
DTMKVSNDFIRQCGLISCHSLCVVKFVILVLRHRKIKRLMDTLQDKKYQYEPLGDFSPGQR
FDEARKLTHWCTIGVFCLYSCAAVSAHISAEVLINKDAKRERFDGNITCYEYMTFYFAIPFPS
DTKAQCEMSFIFMHFCIDIYAWFAAGHDSFYAALLNCLRVQVDILCDAFRTIRPRVLKRLELP
QDLSIFHDDDFPKLEEALYRELTHLTEHLMILLRVADDLEEVFNLITLAQTVSSLIVFASCLFIT

STIPLSSPEFFAQVEYFTCMLIELSLFCWFGSAATRASEAISPAIYESDWYGTSKRFKQSVLII
MCRMQNPIYLSIGKFCPLKLDTIVMVFKCSFSYYTVFKAVGE

>McarOR6
MPLSVFFIYFLIILSSMQPFAAIAYQFYVGIEDMNIISEAFIGISDLVGFLFIYICFRKHRGLIKET
IKASAVFLKYCSPNVMEKAEEEVQTYTKGLLIYFSIGLTFNGLIPLYDYENCDQRRLSDYYR
AHDPCGMPIRIWVPFDARKPVIYYLVFFLHANACLNICYGVLCITMTLVGLLIHITAQIKNLR
QNLLQVFDELPEDGDCYSETLVLKLENKLKFCVKYHIIIINYTDQVFAAFNLMLIVHISLTSLI
FGVLGYQIVTVEDFTEKLRYVMHLGGWIALLFLTCYYGQLILDESTTVANAAYQSKWYNG
PTYLRKNLCLIIMRSQKPLKLRAASIGVISLETFLSVIKTAYSYFALLLSIAE

>McarOR7
VLLYCTTEFAFLCKLMNFVLSKKEIIELEAILESRLFTVDTPEEEAIIKTSTRQIRKLANIYKTL
CFLSVTFYALFPLADGGREAQKLPLPGWFPFNVCNHYYEVFIFEAIGIGLCAWFNSALDLLV
VIMMILGKAQFELLRHRLMNIAIYGEDGERRRRVKMCAQHYKSILRFVMLTESIYSNGIFVQ
FMSSGIVICFTGFQMLIISLKSIQFVQRILYLSCMMYQIVMYCWYGQVLTDSSNKITEACYLA
DWINCNVILRKSLLIIMERAKYPAKIRAANIFTVNLETLLTILRSSYSYFALIYSIYDTKNETK

>McarOR8
MTVPYADDFFHTNRWILYIGGLWWPDTYKSIYHKILYMSYCAANFLFCNLYFTPTEVLSLAS
TYKSIYHLIKNFSLSQMHVLGFTKVLFFVFKGYKMKAIISVLEDKKLHYEDCDEVNFHPGM
LTNKYKKIGRVAGIIYLVLPLVVILLAYTLSAIAALRYVEGDSNHQLPERLPFYSWMPFSYDT
PKKHLIALVYQATPLVSYSFSVIGMDFLFANIMNCIAMNFTIIQQAFRTIRERAAIRVKEPLKV
KDELYNSEPLQRELNKEMRKIIQHLQTVYRMCDELEDVHKYLTLAQTLSQLFILCASFYLTS
ITPFGNQLVIEGIFMIMVISPIVFYCWFGDEVTHQGGEISVAIWQSDWLGATKSFKTCMIINMI
RTQKPVYLTTGKFAPLTLATLVSIFKASYSFFTVLKNTSNQ

>McarOR11
MYFNSLLGVWPFVFERPDFRLWQMMYKMYSNLMLVFGTYVICTQYTQLVMLLQEEEIWV
QEIIRNLCLTLLHSMGLAKVYAIRSDNLKELISEALKVEEDIYRRGDEDIMEIYRLYAWHSRV
SNIAFLINIAIETCFYAMHPLYVGELPHFDKATNQTKMIRALPMSAWVPFDIQEQYLEAYLW
QSVEGTVTASFVMYTDIFSFSLIIFPLGQISILSHVLRNFDHYVKKAQEKHGCDRDEASFFIAR
ECVVKHQDIIRYICVFNNAMKYIMVFDFLQSSMQLATIVIQLFGSELKMVEVIFHGEFAFCM
LMRLMVYYWYANEIMLKSSDITLAIWEGVWYEESQRVKHMMLMIIRRSNKPLALDIGPFS
TMTLQALLGILKATYSYMTIMYNR

>McarOR12
MRANALLGVWPFIFEDNPKLQKIYDVYSRCTFIYYLLFIITAIIKLIFLICDEVFVIQEVIANLCI
TLLYSVTIMRVWAIKTPRVKNIIREIIITEERILKSKDETVITIYNSHAMQSKVSNIIFLVNIFLVT
ALYFIHPLYVEDRAKFYESKNITVIEKPLPLSSWFPFNEQEHYLVTYLWHVLDGSIGASFVTY
TDIFTFSLIIFPLGQLKILIHIMSNFEKYVDKIQNQLDCSPEEASFTTLRECVLKHNEIIKYINDF
NTAMRNIMVLDFLQSSIQLASIVLQLLVAEFTILNFAYSGQFALSMFIRLLVYYWYANEIMVH
SSDVAFALCTSNWYEQPEKVKKMLVVILMRCNKFLCLEIGPFTTMTLGTFLGILKATYSYM
MVIYK

>McarOR17

MVKPIETVCCRTTLKILRTCYMYPEEGKEQNPGKLFLLKWLTLMILSSVTFIGSFLHLIISLKD
EDYRHLDVDFSITLSMIATYIFTCFFFARVKFASKFYMHLSNLERLEKPLDFEKKNERLEKFA
LYHYIYMELLVASLLLFSNVIKGAKCKQENLEFDLHEVCGLFTYTWMPFDIDYFPVKQIYLF
LQLFGTHYLYLIAGTMAWTVVEAIQQIVLRLRHAKYLFTEAIKEVDPVLQRQKFNRAVRYH
DAVLGLDDRLNGTFGVFMFTHLGMTAPILGTAFFAILHGGSGSSLFICLGWFIGVSMDCFSG
QHLQNESIDIARALYDTQWYNCSQDIKRDVLFVLMRCTKPMYLKATSFGIMDRVMLLGVL
KATYSYIALLTQTQ

>McarOR18
MDEIKEEEPFTHSLKMLNVMDAFPLEHNFFSNGIFFVRFWILRTMSFCISCVLPTVHMVTSV
KDGIKLIISEDLSVIVGTMVSLITTCIFVFKRNSWSKLLSDIADLKIYGSFSDFDIVKVKLNLFS
RIYFWYCVNATFVYGSVSFIDTSQCEEINKLGKEWREVCGTYLPMRLPFDANIQWMRVSIFF
TQMFFTLLSLAPSALACSIIFQSTGFIIAHIENLKKHLVGAFDSTDVQETSNILRYCISYHNHIL
RLSARLQDLVGTNISHVLLMSAVVFAGIGNQILKTKPVGGTLYFIGYMIALFLLCHSGQRLID
ETASIGSAAYNSKWYKGNTSMIRDTLLIIYRSQKPCTLEVLSLGSLNYPLFLLIIKTSYSYLTL
LQQTA

>McarOR19
MFKIQKGAPFYSTLLALSLFGQIPVEFKETYSKSFLVKCILSRFIGFVLVSVAPILQYVMATKG
SIEVDISENISITISSIGALLTGTFLTCQYKKWLKFFEDITDHKAFGKPPDYEDLVKNFNRFSAF
YTIYCTGSVPVYAVTVYFNSMRCDEAALKAGFFCKSFTPIWLPIDGSSLQLRVIIYIVQMLLG
GTIVCTSAVINFMVWESTEMLISHINSLKIHFNKISEKSTDKERSEQLGFCVRYHNHILRLSSR
LNGLIKWTSGHMSLTAALIFASIGNQISNSKSVGAFLYLIGYVGALFFICHAGQRIKDELMSV
GDAVYNADWYATDVKTIRSLRFIIARCQIPFHYEAIPLGVVDYPLFLMIIKTSYSYVTLLSQT
T

>McarOR20
MIKIRDIVCCTVSIKILQVCFLFPLKGKELEPNYLRGFIFFFLMGFSSLTVIGSFLHFIISIKNHV
YYHIDLDMAIMISMFTTYSFSIVFFFNIKSAVRLYMTLSDFDEHGKPRNFDKRTKLIDKVVTY
YYIYIEFLIIFMLSTSNVSSSGKCKKKNKKYGLNEVCGLFSYTWMPFEIDYYPVKQIYTICQL
VGTHYLIILAGVVSCLMAETMEQIITRIHHARYLFLEAIKEKDYAKQRQMFNTAVRYHIGVL
DLEDPLNETYGFFMLTHLAMTAPIIGTALYSILYGGSGSSTFICLGWFIGVMKDCCCGQRLQS
QSNTVPIAIYDSEWYTCNEEIKKDILFVLMRCRRPMYPKAISFGVLDHVMFLGVVKAAYSYI
ALLSQTT

>McarOR26
MIEMFKKPLGWLKTCGANPLQKRKIGYMLVNISSTICLAALIVLKMIFGPYLEAAELWITFM
LMALKYLVLLQKKEKLQSLFKDFESFWVLESKDDKISQMLIYLKRLTLCWTFILYVGLAVY
LVKPIFLRDASVFFCYIPQHVPFLCVYLVEVYYLFFVAHSFVGFNLLITTFIILTTTQFRQINMR
LQRLDMERIQDQSGWESCHNILRICVKHHNFLIRVVDDLNNTLYTAVGLLIGATTVVMCMH
MYVLITIDLTSVEIARFLISFSAMVFEFMFGYGLPSQMLMDEAGYMTEALYNSCSWYLAPTS
IKKEMLLMLMRSQRIVCISVKKVVIVNNQTFLMMLKTAYSFYTFLRTLA

>McarOR27
MLKELEYPLHLLDTCGVHPFHKINIFFLIINVTSIIFITVLLVLKFIFDADLQAVELTNVFLMMS
LKYIVVIFKRSEIKSLVKEVDGFWTHQSTDDGISHTFYYLKFITTCWRMLYYFGMFFYIVKPI
FLRGSSIFSCYIPQNIPFYVVYAVEVYFIFTGAGAFVGFNLFLITVITLAVAQFREINMKLEQLD

IEGIVDGAGLDKRLSTLKTCVRHHNFLIKVIDNLNEILYIPVGMLIGVTTLLLCMHMYILSSS
HVSIMEFIRITFTSTAVCFEFMFGYGIPAQMLMNEAEDVTYAIYCRCSWYLATSSVKKILLIM
LMRSQMVVCITIKKLIIVNNQTFLMMLKTAYSFYTFLRTITIV

>McarOR33
MDPVDKMSDYFKHNMISFKYTGIWLNLFDVRRTSLLVIFYSVIINSLFMMSPQVCHVIYMY
KARNNVQAFADEFYVSLASLLVVLKSYSLLKNFDIIKKCWKPWTLIFFKQKRLSAKANTAG
YGNMEMIYWLYATFALLYVFLLLISVLLERILQGTKVLPLVVCYPFEVNVSPVYELMFLYQA
IALSWLVIQNFNLDTFITGLLTVAAVQCDLLCNDLENLTPEKLECQKGEENDIMDEKLVDCIK
HYQEIRRFVNDISHCFSMNIFQQFSCSVITICTTLFEFSTKEPLSQEYFAIIIYQSSIFIQLFIFCW
TGSELTEKSKRIPISAYASKWEDASKTFKSNLLIFLHNVQRPLEI

>McarOR34
IVNMIMVLGDIEKMTEASFLALTHLVQVMKLFYVLRYENKLKLLINSINRKSFQPKNLEQY
VILQKYVRESNIISKTFLSAGFVTCCFWGVSPLTQSGDIVLPLAGWYPFDTDRSPAFEIIFAYQ
FVASVTNALSNISLDTLMSGLIMVVCAQLNILNDSLRNIRKYAEAESDDGRAVSREELQRRM
DERLVECVVHHKHILEFSNEVTFLFTNSILGQFIVSVVIICITLFEITLLPWGSLKFFSLILYQFC
MLLEIFLLCYYGNEVILQSMQLTKFAYFSDWTDCSTKFKRNLLFFMTRSQVPLRIYAGGFFT
LSLETFVKILKSSWSYF

>McarOR35
EHYWVAYVWLVSSGGVAALNVAFIDTFMFNLIIFPLGQIDILMHHIRNFNKYALEVQNRVGC
SNEEASFLLMRDFILKHKEIIRYIDDYNIEQRYIMVFEFILSSIQLASIAVQLVLSHLGFFDIVH
WGGFALCMLLRLLVYYWYANEIMIKSSNLGNAIWESDWLEKSYKVKQLMLIFIARTQKPL
GLEIGPFTTMTLERFLGIVKATYSYVMIMYR

>McarOR36
NTADTNITSHVNLCIKYHTTIIDFADETNNAFSVMMLVHITWTSFIISVLGFEIIMETNYSNSF
RFAMHLGGWLVMLFLVCFYGQILMDDSTDISKAVYDTQWYNKAPTLRRSLVLILLRAQKP
LVLRAAGVNIMSLATFLGVLYSAYSYFTLLLKLSHS

>McarOR37
PIPGWLPFDINVDFYYYPTHVLQVTTILISSSINTSLDIINYVLITIVCCQFDIIVDELRRIDLKIS
SGEMQLKMVIKRHKEILKFSKNINTMYSNIVFIQCTSSVLIICLLGLQFLMVPLGSTKFVSSA
SFLFTMFLQIANYCWFGHTVIIRSTEVGDACYDTNWNESNTKIQKLLFIIMERAKMPVTFTA
GGFFTLSLTTLTKIMKSSYSYLAVLQQLYET

>McarOR41
DMMDTDHTYLAFTIKIFKFLNMWPNEKENGDLLRNTTFLLLLHCLCLQLWLILFYNSMMTI
SRLLVLVESIIAISALCSVIYISICFMIKKARDRTFNEEPKDIRGLPSDAEHPETEETAEFLHKDII
IYGIVGNILYGCLPLLSYAECNKNRSHHMIKYGIPCGVIARY

>McarOR44
MQLIYPKEHECTIKHDPIWQVKFFLRLLSFNGIKKIFWFCAIVLKILTLYAETTFAFQNIQEPR
KLIIVLATYPVRIMAVWKLIHMYVDKERVDRFYRTIEKDFWEFHIAGPTLERKIRKRFLATNI
FVFSNIIFNVICVTLFALGNIRLTPEGKRPLPNHNIWAPFNMDSSPVYEVLYVFLLWNLYLTAL
GNAFYDMVFVYCVQHLIVQFIMLKELLKNISLGIMDHKSDVEMFNSEYFQKIVIERLKICTK

HHNKLLIYGKNIEHFCKLVLVPQLIMSFAILVINGYNVTVESDDFSTTLMLTITCFIQLAVYAL
QATELREESLSILNSIIECKWYLFKSPLKKTLVFILMNAEEGIVIDAAGMTNVDNPLLVDIIQK
VFSSITLLQAIINED

>McarOR50
TIPMMGHTTNSIENRTSRKQCKWMNDDPIWFVLMLLGYGFKDHIFLFQAVLLSTAGYLEIF
NIIFVVQALTTKTWCFTQYITGVILQQTNISPPSNFRMIFVILLASVLTITASISSQRLIDESDKL
FTNISNIPWVHWNEKNKKAVLII

>McarOR54
MVLFPKNDHFKVTMYANALLGVWPFIFEHNPFMRKLYHIYANFTFIYFMLFIVTAYMELVV
LLMAKELRVQEIVGNLCITLLYSITIARVYAIKSDSVKNLIREVIEVEEVIYKSDDEEVIGIYKE
YTHHSHISNIIFLVNITIETIFYFTHPLYVGETIVIDEATNATKVVRALPLSSWFPFDEQEYYHL
TYGWQMADGTVGASYVMYTDIFTFSLIIFPLGQIRILMNILRNFDKYVKMTQDQYGYERDE
ASFLTARECILKHKNIIRYINEYNRVMRNIMVFDFLQSSLQLASIVIQLFVSEVRLFNVIFHGE
FALCMLIRLLVYYWYANEIMVQSSNVALAIWDGGWYEEPQKVKHMMMMMIMRSNKPLV
LDIGPFSPMTLSALLGIMKATYSYMMIIYN
>HparOR27
MAFNVKVLKVMKLWLEDDENDKNKWKNIKTYYLSIVLFPSWGPLLLECCFSIFSPSADIVF
KIQELLGTV
CIIGAMYMGICFVQNRKDIKNLLESIDNFALYTDMNSKQVDQKASFYSKLVIAYGLLGTLIY
TTSPLLSR
SSCAVKRTQYEIDHGKPCGIIVALRLPFRYDITPTFQLMILHEMLTAGMTALLVVSITMLICGI
LEHAIS
QLKEVRKCILNLGQASDEEIVQSVGFAVRYHCAVIEFIERVNECFGSQLVLHFTLTSIVISLLG
FEMLMV
NDVKESVMYALHLIGWLIILYNICHYGQRLIDQSIGVAEDAYFSPWYKFSVGMQKDIKLIIIR
AQKPLTL
NAVNLGILSDPTFLGVISSAYSYFTLLLKIKNP
>HparOR47
MVLLTYCLLIYQPAETIVMFKSWNLSLLVKSLRDQLNHFICIYKIFLWFTKRRQILNILKGLQ
SQEFMYE
KYNKYNPGKILYKYKTKSDRWSKLFLYGVNGICFNMTLSVLYVYIFKYRDYSSEDEEGNLI
YTQKLPVTL
ITPFKQNTRLGFILHFIFMILPLDIYGWIIIGLDTLFTSILNCITAHVLILNGAFETIRPRCLTRLK
LNN
RNLSSIDMKTLHNEMLKEQNKCIRHLQKLIRISGKVEEIYNVQTLGQVMISLFEMCFCLFLL
TLSFDSSF
GNELTYLFSTALQLLLYCWFGNGITEASAAIPSALFKGDWLDASLKFKKSMLITMTRMKKPI
SFTIAKFT
PLAFTTFLSMARVAYSFFTVLRSGVFESPE

>HparOR46
MDALPGGQQTSVRLLYLLICGNKLKRLYEMVNETWPNDVLGSVLKKRFEFDSQRFKKFFIA
YVTVVTTIA
MIYMMAPTFCSYRRLVTEWYLPCDLEFDVCYISARIAELVYMLLIFYQVLVFDGMFCAFLF
CLFMEVEKI

KHSFKHLNIGELNSRNEVNRKFCSILKHHGFVLEFLKRFNAVYSVQMLNHFLTLAAALCFG
MFLFSKDGF
PPSSYQSARYGPYVIAYNIQLYTCCYAGELIYNQLLSISDAIYESMWYLNNESNVSNGVRLAI
QVSQIGN
KITIGGLWPLNLRTYMSVFKTSVSLNALLQTVYINNEKSIE

>HparOR43
MPAGKTTKSGLRKLSFEFSKDVYKDGVKMCILPGKVLLQGVCCWPDDEGLRMKIVGWFL
FWNLFVIEIFH
ASYVCLNIKDIGDAVDAGATVTTTMEGLVRLHIMLTKRDVINSTLVKIWKQFWSLDVIEPVK
RRKLKRQA
QMAVMLTSIFLGSSIISNSQITGMPFVRNRGLVLKSVFPFDWRRYYFYEIIYAWQYYSDWFVL
FMINAFD
FFFIALVIICSVQYVIMQEIFKYILTSESKRHRKIIFGERGETMTDREMLFECLEQHKLLIGICN
ELEES
FNIAILIQFFVSTSAICAASLVLKLDYSQFLKMLMYAAAHLSQLFYYCYAGHELSYETGRLS
DAIYHCNW
HLTYDRDFRKAIVLMIQRSQRVQCLTAGGITELDFASFLKIMRLSFSFYTLLNNLLTKHIN

>HparOR41
MAADRLEIFEMMWLTKKALWYFGLYSSKNMRSNILCKMNLTLILFLWLSFLTLMCMEFLE
DVSLFLDIVL
VFYGITWCTGKLLLFLYISSDFNVIENLLEKSSLNMQNAEQDKLVSTTIRNYQNITKLYSCLF
ATAILGI
SAFPFVNKETLIVPIWAPICRNNTVATYFWFAYEAFCMYTAGTVFCGVDFIMLGLMMIIALQ
YKILRDNL
EKSAKRDPRKDYLTQEREIQERLRKCVVHHNAILELTEKVETTFSSIFLYQVLVSVLGICVAA
VQIVLIP
SVAVKHAPPCMVLLALIFQISIPCWFGQNIKTQSLGVTDSCYASEWYTCSTSTKKMFFVIME
KAKNPATL
RAKPFFQLSLETFVMVLRNAYFYFTVVHQVYQQ

>HparOR38
MSDNFFNVNFTMLKMSGIWIPDPKSSLIVKILYWAYNFWWIGYSCLFFCPSELVYFATTLSN
LEDLVKNV
NMGMTHFLANIKVCLWFYYRKEIMGIIETLGAYGRRYESYGDFDNEKILRKAKKFKDIFSV
LFLNFAMFT
SISSCLICFYSTVKLDVPKGGRIELKLPYFSYIPWDYRSSKILFSIAIWYQFFPVFNYAYIIVGF
DTLYT
AILGYVSAQLDIIQGAFSTIRPRCMVRLGLELPESILTDPPELMNEMNKEMNKIVDHLQVLL
DICIRLEE
IYSYVILAQVMISLIVFCTCIFLVSLLPMFSLNFAAEMIYMIAIECQLLIYCVFGNKVTLSSSNI
PNSIY
NGDWYSSNISFKRSMLITMSRMQRPIYFTIGKFTPLTLSTFVTISRASYSFFAVLKNSDL

>HparOR39

MSATTLPMRKLSTEFTKDVSRDGVKSCVLPAKFFLEGICLWPDSETLLKNVVGWFWIVNLSI
MEVFHARY
VLKHFKNISDAVGAGATVSTTFSSIARVYYMLTRKRLINETLVKIWKQFWPLDAIEPMKRKE
IKTKVQIS
LFISFVLLLFTTLTNTVITCAPYIRDRDMPLKSAFPFEWHRLYVYEFLYLWQYIFEWFVLFMI
NAFDFFF
VSLVTICYLQFVILQEVVRLILTEESRKHRVMIFGKRGETMTDREMLFECLEQHKLIIGICNEL
ESSFNI
PILIQFFTSTCGICAATLIMKVDYSQFSKMFTLVGAHISQLFCYCYVGQHLAFESDYLSYAIY
DCGWHID
YDRSFRKALILMMQRSQRTQRLTAAGITELDYASFLGILRLSFSFYTLLNNLFMKNVGQ

>HparOR30
MINDNLKNKKLKDYFYLQKLVLRMTGVSLGENDTDAYKLYSMFCVFLAITYTGVEAYGLV
VHINNIDMFA
NVISLSASHILGVVKVFVLLKNKTSFAKLLNTFEAGIFAPNHQRGGILEDELVEKCISYTTKQ
SIVYWSA
VSMVLFFGILDSVLSKIKSNDYNDWSMPLAPFSLFEVTTNAQFILVCFYQSTCLFIFASIISSID
LLMGS
VIAHMKTQFTILKNTIKSIRSDVDDEDFSEKVTARHLQKTSAGLLQKKLKYVVTYHQSIIDL
TEQFEDAF
NFLLLTLFIGNFLVLCFTMYHASLYPLSNTKAFMDFTYVGAICVQLLLTCYWSNELTLESES
VAYACYEV
NFVGASPAFQKNLALMIQRSQRPVVLTAGKFVNLSLDAYVSILRMSYSYYMVLRRKNEYK

>HparOR29
MKNLEALISEIHVLLMYIFFISNQKLIALILSTKASFRSTTNSPLMKKLTEIKVCVNKFLFALA
VSGYST
VIMLFLALLADRDKFLILESWSPDNYILGGLYSLMQLFMLSMSAQLTLGIDCLLVAFCCHLII
QVRLLKY
DFRRLYVPLDADTIAERRVRKELIKCVKYHKFIMRFFENLRKLCSSLFLCQYTIALVSFCTEL
YAFSNTK
EEFDVTELFKSVFYTSTILLEFGMYCFCSQYVVDEMRSLSDAIYASKWYECKTNVQKDLLFI
IMRTHSQT
GFTAGGLIAIDVQAFASVIKKAFSFYALMKSLF

>HparOR25
MSSFKRFLSLLKSKFLGNDVKLSEDGAKYTIIFSQIITDIINIWPEKFSTSTTVCFSVTFVICILQ
EISL
FVFLITSVEDVDSLTKVLASMSIVLQSMVKGSTFYYKAEEMNEVIKIIRHEFWPSNIMGKDL
DCKIRGNS
KILLSVLLVQYVFAVAFLLFYVILPLIQGTKQLPHTSWYPFDWSATPAYEILYCLQAYITVYFN
ENVVCA
YDSLYCSICANCVAQFRLLSEAVKHIGTGREDEIAGSLLKIPGVNYRATLNVEDYDERRLLVI
CIRHHQK
LIEVTDQLNKIFGNGHLVQVFASVLGSCTSIYRITTKNGLNDLTFLISYYMAHVVQLFEYCA
LSNELSYW

STCLSVTAFQCFWYRKKYSDIRGCLSLVIMRSQRSISMNGLGLFELDYVFFMSVMRFTFSLY
TFLSRFTE

>HparOR22
MAAATVTTTFECLVRFYIIAFKKPEINDIIFKIWRKFWPVMVVSPKKMEQLIRKCYTALILTV
GCYGPAI
LCNSIATLWPYLSNRELILRSVYPFDWNQTYVYEVIYIWQYVTQWYILILVNTFDFFMIPVV
MVCTVQFG
ILQDVFKNILSQKSRRQRLLIFGDVISDRDMILRCLDHQQMLISICNQLEEIFRFAILFQFVTST
AALCS
SALMLQVDSSHFMEMLTFVIAHMFQLFYYCFVGSELISYSENMANAIYKCNWHLSDNREF
MKALTLILQR
CQKPQRLTAVGIVDLNFVSYLTVLRVAFSFYTLLTKVIDRGTSWT

>HparOR19
MLEEESLKILGNKGLNPIISTFASKIKAVVFILLDNMLGISIIMGLIYNTLDTYIIIDSCNAAAVL
YQVV
SKQIVLLIYRKEFSDVIYCVKKFWSSNKFGKKSSTKIHNIQYIVKKLLHIFKLTVLITTILYLLK
PLMEG
ERVLPFTWMNFCNIERGTMCYVSSYILQICGVIDILYGMVGFDSLFFILLSYGYCELEQVKYA
FLDLNVN
LSVNGDEIEVLQKIGILVRQHDRVLIFLRKVNKLFTNLLLCQFLTSVTCLCTGLFLLTAKGFPP
SLTLIL
RYVPYIFTALSQICIYCTAGQIIGDQSESVANAAFETYWWTKRQPLFRRSIILIIERAQRRTQIS
AGGVF
KLDMTTFVSILRGSVSALTLMQTFYNDP

>HparOR18
MFDLNIRFLKLTGLWCILNKSGNSVKSLIHLLCFLLLAQIIYLPAELVKLLSFSENFEQTMEQL
GFMLTH
ILGTVKIISLYISRKKLSKIMDDLRRYSYIYDCDVHNNCHSLEEAFNRKAYRVCAFFFLMGSC
AGLLKFL
ITTIHLIYCKEDTTDKELCITVKPFFIPIPVFLNTIPGRWILGGFQCVCLTLYAWQIVAYDTLFAT
FLIY
IDCSVHILRYFFETITERSMNKLQLRSCITSNPELTRHMNMQMRTGTINLQNLIMTCVEIADV
FKYVILL
QVLFALFILMTCLYVAASVPLFGASFTFQLEFYLTIVTQLSLYCWFGNEITLSFRKIPDAMYN
NNWIPGD
KSFKGSMLLNTLRMNKPIHIMIGMVTPLNLNVLIYILRASYSYFAVIKSKY

>HparOR16
MFRCEEILRNPRWILEVFGCWPVRKPTKTYTIKMLAASAFTLAFPAMLCVEILFAYKNYNTL
MQILNFLI
PYVYVLCKYTIFTFQQNKLFALLNWTNSKIFNQSFVDGDKILRSAYDVVVMVTKVFRFTLA
LALSMIALL
PLSETKLYPVPISHDLGSYSILMYVFQITCLGIGGWLCIGYDCGFIAFTAIGIAQIDILKLRLSS
VIQDL

GLNQDAFSNSSNVEQLKMQTKIKETLKACIIQHLAIQEYCKRLNNLFSTTIFIQYLMSILALAI
TMSRIL
TITELSVKILELAFILMFIFTQLITYSWSGNQIMIKSSDIGEACYMGQWYYYDVSNRKILFFIM
ERAKRL
LYIKASMFAKTTLATCINILRCTYSIFTILRMTYHEN

>HparOR14
MSMSFILTSFKVLIWFYYRQPLLKITRLLEKNSSIFQDYDFDCRGVISKEKRFKDLWTKSFFL
AATLVSV
LAGFLSITETLTSAEKYVEFKNGTHYVYNQKLPYYSWVPFDQTSSKLAFVVAVVYQCLALL
NCGYITVGL
DMMFVALISFINAHFVMSRNAFRRLGTVCLKRLNTESTISNHLSKMLQKEYEVELRKCIKH
LQMLIRICQ
TLEDIYSPLVLMQVLISLVVLCTCLYQVSSIPFGTKLLGNDLAYLLAIEMQVAVYCYVGSKLT
HNALHIP
TAIYESDWLNASLTFKKTMVITMMRMQKPMHITIGKFSPLTLNTFLTIGKMSYSIFTMLKSR
N

>HparOR12
MLACLHFFMTLTGVWQGKRMSVRTHQAIFYTNSFLFTYYTIGFYLHTFKIFTDDKTTIDDVI
DTMFTILS
ATNLLYNYIAIVFLRKEFRALVDNLMNFDKYGIPKNLRAYDERANVLTAFTFSYCFMAGAII
QMGAIFAY
ETCMEHSVRTNKDMTCGYGCKVHYPYKYQSGISQIVHVTVGVYSLSFLASSGGLFVCLFVS
FVEYIIVRL
DHLADMLLKVFDIEDYKDQKEALKLYVEYHILIMGLVEQTNSCLLILSTPALFIYSSIIGLSLF
AILNGN
IIKPSVNALGFIFTTFILSSYGQQLIMKSEAIGTAVYNSKWYHASPYLKKHAVFIITRSQKPFL
MNVGFG
FYFYLPLFTQIMRGAYTYMSLQINTD

>HparOR10
MEVFHAAYVVKNFSNIGDAVGTGATVTTTMEGFVRVYVMVTKRQVINTILVKVWKQFWP
LNVIEPTKRQQ
IQRKARLSVLLTSVLFFCSASSNSLITSTPYVRYHGMLLKSVFPFEWNQPFVYEIIYIWQYYS
DWFVLFM
INAFDFFFVSLVTVCFLQFVIIQDVVKFVLSERSRSQRNVIFGRGGESMSDREMLLKCLEQH
KLINGICN
ELEECFNIAILIQFFVSTSALCAAALIMQVDFSQFFKMLTFAGAHLAQLFCYCYVGHQLSYES
TCLAYAI
YDCDWHTNYDRSIRKALVLMIQRSQKVQSLTAAGVTELNFTSFVRIMRLSFSFYTLLNNLL
MKTESQ

>HparOR7
MRWFKYDREILKDKSKYTMVVSQILLNIINFWPEKYSFATKISFIIMFLTCATMETSLVIYLLT
SIEDVT

SFTKVISSAAVVMQCMTKMCIVFFNSKKLNVIIKTVWYEFWPSNMMGAITEENIRSDSKILL
VGFLIEYG
LGCVFLLLFIIAPAMTGIRQLPYNAWYPFEWSTTPTYEILYVLQVYIDIYIIANTVCGYDFLYG
SICVNC
IAQFRLLNEAIKKIGTGKEAELTSLLLETPGVNYRPIFNKEYEKERRLLVICIQHHQKLINICY
QLNKIF
TYGHFVQLSASVMAICMSCYLITTESSANELSFLTSYYIAHVSQLLVLCAISNEVTHWSSSLS
ITAYECA
WYNNKYTDIRKCLSIVIMRSQKPLSMQALGLFELSYASFLGIMRFTFSLYTFLGKMAN

>HparOR6
MRNFVLNYSLKQLSLLCLYPDIKNLQFQIVGILVCCFYASAWIFNITGVIINFNGLDSIQQAM
VSLPSAH
QLLFKTVFMFFTKSKLAKVVRKINYLQSLKLYTTSENLRRKIEEADEKFNFYFKIYKRIILITS
YIFITK
PLLSRTRHFPIGWYTPCDINDNFCYATWYIWESVYVLFAQYTLASVDSLFFAIIFTMYVEVEK
LKDYFQN
IYVKDKIIKRINYREFCKSIDHHNYILKVLEEINDVFQYQLLNQFVTSIVTICFGIYYLNASYP
PHLELI
PYLLVYHNQLFMYCFAGEMIQRQITSIGTALYTSNWYCCKNKEFTACLKLIIQRSHRPINVNI
GHIWKLD
TRTFIGVMRTSLSIHTFLQTINKQ

>HparOR4
MVARPDQFDAYKFEKAILIFFGFPVGHEWKGLQLVKGIISIIISISLIISMIGNIIEKVDNVPLILE
TLY
FGLTQTTFLCKAYNLIVNKHKVSLLETYLKKPIFNQYTVDQYNFIKKAVRICNMFAKTFRFF
VGITIAFY
AIFPFIEHTLPLPGWFPFDKRKYSYILYIYHLVCLILNGYNHTSLDCINAAMISIASAQFEILKD
NLHNL
RRDEDNDLTIDQQDAVVRTRVKNCVLHHNVIIKFVALIEDMYSNVLLIQFLCSIIIMCVTGFQ
VFVIPPA
RMHYITLVLYFCCNLTQTAIYSWFGHDILAKSSDIGLACYMTEWNVLSPKARKLISIIMERSK
IPIILTA
GKFINLSLNTFMMIVRTSYSYLAVLQHMYKT

>HparOR1
MEYLDEIRMLRVPADLLKQIHETQYLTIRLLIFRIFNFCVIILVGTYIFVNLFYVKNETFIKTMQ
SLIHM
THLLLKYFFLIYYKLDIGKLSVSISENFWNIEAFEKNVKVRTQKVYNIVERIQKCLLFVTIML
GCLYISK
PLLDKNNSFLLETYIPRSNAIDAFLLMSQLYCFFIGIVTIVGFDLIYFSLCVHVIIQIKLLKEKL
KNSFK
SFRKNAAYELSSCIKHHQYLFSMFLRMKEIYSIMLLFHYFVTLLSVCSVVFEILSGHTDLSNY
LLKFMMV
LFFVVQFAYYALPAAEVASEFSDVSQAIYTSEWYNSDIKLQKSMLFIMMKCQQIHYFSGGDL
MDINTDTL

GSVIRKIFSFYTILRNLIDK

>RferOR2
MKPVKYRELFKTDIFALKLSGLWIDIDKKRPIWHYPYAFVILGLFIYPVNLLSLINWVST
STDVKMFASMCYLVMECHMAQYKSIIIMSNREECNKIFAMMDNDYLQPRNSSELTIVNGV
LDKFSVLKKWMSFISMGAVVMITIYPMYNGEHLTPIRSWYPFNINFFPVFEIFYAHQTFA
IWAIGIINIFSEVVITGFLTFIGLQFDLLAFRSQHLENFTTHQIRQIVWNHHQILELFGL
VKGVFGKIYFWQIFTTTVTFCMNMYLLSVADVKSFEFLYLFLYQTAIFLLLLFPCWFASV
MTEKSENVPVALYSCNWFDASEDFKKQLVYVMMRTQTPLMMLANDFFYISVELFMKIVKT
SMSYFAVLTNLDASENQLSI
>RferOR4
MGISELYTKTMKHAKLIELARWHLIFMGLWNFKKSDPSRRRLYLLYDIYKAFIPAYMILF
LISLILNMIQLIANKEEIADIFLSVAFLLSNIDITSKILIFIKNDIPDWLYLVCDMERNL
WEYGTEKMKKYYMGQVNLCNIFVTISTAMIIISMSIFIISKLQLLFVSEIAPGKGLFFMW
LPFDKTKNAVATWTIEIWLCYFNVLLFVTVKITIITLIIFLYTHLRVLHIRIKNIGDKQT
NEEDVITFIKQHQHLISLENRVNDLLKYLLLQEYFLNAANIAASLVQILVLESFLEILYM
CLHGLYASFQIFALGWVANEVKEESMGISDAISNCCFYNCTISTQKSLLITMIRAQRPLM
LTLGPFGSMSLSTSISVVKAAYSYATLTLNRYNAD
>RferOR1
MEKRPFDRNTEKNTRLIGFLKTLMVCTGTWSYHWTDNFILKKLYHYFFFFPLSVFGAFYI
AIIMELFRVLYERDKLDKLLLNLGIVLDASKITIRLFFYFKNNVLAKFKNIMRDEQDIFE
SQEPEVIEYYLQRAKSWRSTLIFLYITTCLCSYGYMIIEISDNILVNRRHKANNESVEPP
FLYQIYYPNPVENRVSLYCVNCFMVTFIILAVNVSFGISTTCMVYGATQLEIFQIRLKRI
HEIAEKSYNGDVALTLKTYIVKHQDLMTFILDLNDCIRSATLGEFLISSINCASVVADIT
KVSDIGLDATYSGCYVVLLISQIFTYASAASEIKIQSAAIADAIYECEWYNFDKNSQKMI
NILQSRAQKPLEMTIGPFGAMTNETAVMMMKACYSYVAFMKDSGVAETTVD
>RferOR3
MGTVLANMFFDNGLFPRCKFMLSMCGMGLYGNNTKLQRFIYKVFSIISQIIYVQCVVFFS
VSAYLLGFDDIGQVLEGFSRMTYVILIVTKMILIQSKRMRDILLMLSREEENCLKAHSNL
KKIYTFHVRYCWIIIFFVLIFLYAAGFINIEMGFMRYFEWKRIPNRNSSIPKPHTIPFWY
PFDRDKHYFIAVSYQICHILQTLLINGSIQALVNSVLVFIRAQLKMLQYEIRWFDWSENE
QKYMHGDYPRKNLRRLVIKHQEIIRWVNMFNDCFKTIFLLEYCITSLQLATTLIQIVQGS
KLIFNLAFFTHCSLQLLALSWNANEILLESSIGFYRALFECPWYLHDKYCQYIISMMLLR
CNIPLTMTIGPFGEMSIDMAISRLKLSYTCLSILQVTTN
>RferOR8
MDSLKQVIYDPKKNEPFYFTLAYLRVLKYYPNSSDYRNFKRHQALAILYRMISTFVCWEC
FLHIFMSIKNGPYVNITDDIVGVTGVANCFYYGAVFQYSNKRWSKFFSDLTDTVQFGTPP
GMDKIVKENNRFSMMFGYFCISGCLLYSAISLIDTENCERSNLQKNAHEICGMITPIWWP
TKEINPLLKGFMLIYQFICVLLIIPGSAMFFFLPLESARLLSVKYKHLRELIHQLFEHED
DGNKMKRLGQCIRYHQELLRLAEELNQLIKVLFGHVSVVGAVALSTIGYYMLEQYYAVIH
IISYTVGLYLLCAGGQMIKDETSNLCENVYMANWYKDLKLAKNLKFIMQRSYKAVVLESY
MFGDLDMILFVAIVKTTYSYLTLLSSINFD
>RferOR5
MNLFIYKPNDFFARCIPLCYVMGAIPREATDPNLKARIGYRVYSVILLGSALFFNSATFY
KLFEMIIRRDYVEKIITNYMLASLHLAGIIKSLMTKSKKGGWLLSRMIEFEKKVYKSNDK
HILTLYRRNIQAFRSLSGWYFVGIVIVVACYASSSIFRPKEFMIHGNETIEVKQVPMMII
SPFDQHDQHMAAFFWSTCTGGFLAIYFVTGDLMCFGFTIFAFCQIDILKYFIENFSKKSQ

EIQNYHRCEPGHAFRIVQRECIIMHQDIIRYVEELNASMKYLMLVDFLPGSIQIASLLYQ
LMNNLNAIQIVLLGIFIITLIIRLHIYCNNANKITLESEMIGDIWYQSDWTEMPLDVQRN
MMICIARTQKPLTIAVADFQNASLATFVAIMKATYTYMMFLTTF
>RferOR20
MNGDEFTYERTKNTKFVGFLKFLMICSGTWPYQWTDNIILKKLYSFFYFIPTAAFALFCI
SLPLEIFINSTVSTENIYLNSGFAVDVTKIVIRVIIITKNKLFDIIKDIMSNEKILYKTN
DWDAIECYLGYVRSLKFKLITLFITTMKTCITFILIEVYNNIETNKIHVAKNETIESPLL
YPLYIPYYSMTEQNVEVLYILNTFVALLVILGVNVTFGFSITFMTYAAARLGVLCIRLRR
LKDIAITSYGDDINLALKILIMEHQHLIGFIRILNDKISMITFAEFLFSSINCALVIVNA
IKADTLTISSMYSLGYMTLLIEQIFFYASSANEIKLQSIAIIDACYESDWNTFDEETKKM
LLIVMMKACKPIEISIGPFSSMTNETALMMMKACYSYVAFIKDSTI
>RferOR22
MYDVEFTEETKKHTKFIRVLRILMISTCTWPYRWTDNMILHKLQSIFRIVPIFAFYFFCT
ALPVGVVKNAIDQEQFDENTFMHSGIILDIMKIFTRSIIILKNKLFDFYKHIMLYEQNLL
KSGESDVIECYMECARSLQIKLIILFLTTGECCFGYVTVEIVGNVNANRIHVANNESIEF
PLLYPFYIPYYGYTEERIGIFYFINLIVVLFVVLGVQVTFGFTITSMTYASTRLRIFQIR
LKNIKNIADTSYNGDVYLTLKIYIKEHQELISFIKNINEKIKTITLGEFLLSSMNCALAV
VNTTRANGLTVDAVYSLGYTVLLIEQIFCYAAAANEIKLQSTAVIDACYACEWYTFDEKT
KQLLLILMMRAYIPMEINIGPFSSMTNETALMMMKACYSYAAFIKDSME
>RferOR41
MILLSRKRLIFISLLKQYDEQLFQPRNENEMELVKINLKKFESIKRSLTYFCYSSLTMSV
IAPYFHQEPFGLPFEAWYPIDMSYCLYVAAYIHQTIAITLLAFGFIFGEILVIQSMTFVG
LQCDLLCYRLKSIKVKQNSDKENKIILTEYVRNHLMILKFLDITGEMYGMTYLLQLFACT
TTFCTGLFLLTVTENSFEYFYLLSYIMAQSFLLLTPCWSATQMTRKSEKIPTAAYSADWI
HGTNSFKQMLILIILRSQTPMKFYAGNFFELSLDTYVSICRASYSYFTLLSKMTNEK
>RferOR28
MSFYEIGLLGCVFLYSFIPILEHKSLPSDFPFFNEGLFHYPFYIFEVSAISISAFINMSL
DLLALGIIYIAAIQLSILNKKLQDIDKNIKRRSQSCNDVENLTLIYLNNCCQHYVDIEKY
IYLTTELLSIHIFANIGGYVIALFNSALQLMELTPFSLQALGIFTYAVMVLFQSAAYCWF
GNEVYIESMEINNSCYNSEWYNHGQDVRRTMLILTERAKKPFEIKGCRYVVLSFDTLIAN
ANHRNVNIITYEQGTNVLNGISLPLKMM
>HeleOR1(a)
MCIVKLKINFMAKLSCLQPLKMSSYVLSKCGLLFTREEITNLDILKIILN
TSFAVVGLILVILNVGNGMITHNMTLINWSMCVLIPLVYYLAKQFTLLIN
RKYLLSLLSDLSSDTFNSHAEELNSHVQNINTITNVMLKCSVFAGVVLVI
VTCILPVAINMRMMIPSPFHTGRFDILYKFFHLFAVTYLGMNAIIFDIFY
LTLMASAVAQLNILQERLLNVFEGAQNMKARIYNYENVSAITGCILKECF
VQHQMIISFIAKLSILVSFPLFAQYTCGCFTICNTIVRLLIMGETDTTSV
ISNLSYSSVIFTEITAYHWLANEIIYKSNKIVESCYLSNWYEMDVKSQKS
LLPLMERAQRPLVVRLYNFVDVSLESLGMIVRWSYSIFALVKARYS
>HeleOR2(a)
MTKTKEKELLEVSLRIMRFFQIFPKDNAFSRWSNLQNIFIFFNPMMIGSV
LHFLHNRSMNSLHIDEGVEDFVFIMANVGMTLGILHFKYNHRNILLFLGK
LQNFKDFGYPTGLFPLNKKINQFTVYWIMFAILGYFTNAFCRIFLDDSCQ
KYNTKYAKSDICSVATPLWLPFELSFSQKLLVIGMQLSCVLYLIITCTLV
AFTTGCIEFIILRIEHLNDLFGKITKPGSYFQSKHSLIQCVKYHTHITLL
AQEFNECFDKFTMLFYFQIGPTISLLSFIIILEPKIRHALHLAGWLMTLC

ILSLAGQRLTDKSLSVGEALYDTDWYLLDVTLRKYIILIMLQAQRPLKMK
IWLHSEASYISLSKVIKITYSMLTLLNGTLKQNS
>HeleOR3(a)
MTVEIVPEQDIYRIGIYVLQFIGEDLQYTSKKIWIFRIFNCTLFALLLFL
VVLNVQNVRSEMFTKSAESAMVALHPLLKYILIICHKNDFRNLLKTQLER
FWRHKGFTEEVIQSNIRLHNITKRTQVILVAVAYTAAFGLFMRPAWSGKM
IHPMQIWTSEDYLTLNALLLASQYYLLYFTVPTIFGYDIFYLSLCVDMIG
QVRLLKSKLQYLSMIPHKNRMEEVKRYIEHHQLLLSVFNHMRGIFSVMLL
FHYFVSLTLTCVAVYECLRGKSSTIHAVEELFNAFVIMFEFAFYTFPVEE
LAFEFHDIPNKIYMAAWYDNDIAVQNQLLIMMIQCQRKRYFTGGGIIEIN
VQTFATVVRRAVSFYAILRNLLNR
>HeleOR4(a)
MLDVLYRYETFSGVWPEKNRIKDIIKLIINIGLMFYLITGMLLYLIAMLR
EPAATVMNITDAMYTVQTSGNFCFNYIGCVVLRKSSLKFFRNVRNFDLFG
VPKNLHERDAQINKNVALIVGYCVTGCIGGNAYYVFTKNWCIKRHNVGDI
HFCGYPCGLYYHYDYDHGVLAIIHKILITYTYTLLCATGILFICAGYCAI
EYMVIKLEHLKEMLAEVQTLKERPMILQLLNTCVKYHIHIISLAEELNSC
YSVINSPAMFFYSVTLGICFYSLSNEYTIKALLLTIGYIMAIFLLAMSGQ
NLMDASESIAFAVYDMEWYNMDVDTRKYISFIMLRGQRPLTFKGGPFGVF
SLPVFMNVIRGSYSYMTMQSNMK
>HeleOR5(a)
MSKVNCLETLKPSSDILSKNGMLFLTKITKKDVLKIALNFAFEIVVQILI
ILNIRNALNVNNIRLANWMTCLLLPMTNYTAKRLTLLVNRECVLSILKDL
QSDVFNKHSKGLNKHIKLIKTVSRLMIRYFLFTMSSSITIYCILPFVINI
NMMIPAPFKTGHFDIIYKCFHFLLSFYLAVNTTCFDIFYLSLLGIEIAQL
NILQERLWNLIEDAREMKKSSTGDTYKNLNLRAEDILKDCVVLHDMIIEF
ADKVSILLSFPLFMQYICGCFIICNTILQFTIMGETDTSHLIGISGYTSI
VFVEMAAYHWLASEIIFKSSKIADSCYLSNWYELNRTCQKYLLLLMERSR
RPLIIQLYGFVDFSLDSLGVIIRWSYSLFALIKARYN
>HeleOR6(a)
MSTKEEPLRKLSLEFTRDIYKDGARRTLLPAKIMLQSMCAWPDNDRILIR
FMNWFCFVNLLAVEIFHAAYAFKHFNDISDAVAVGATVTTTMEAIVRLYI
LIVRKHTINNVLVKVWKQFWSLDVVEPIKRNKMKNQALTGAILTLSFFFF
SFISNTQITGVPYVKDHELILRSVFPFAWNQSYVYEGLYIWQYYCDWFVL
FMVNAFDFLFIPLVMVCAVQFVLMQEVLRNILTTESKNHRVAIFGKSGED
MSDRDMLLACLEQHKLLIGICNELESSFNITILAQFFISTSAICAAMLVL
KVDYSQFLKMLMYAAAHLTQLFYYCFAGHQLTYESSKLADAIYECHWHLS
YDRDFRKALVLMIQRSQKVQCLTAAGITELDFTSFLRIMRLSFSFYTLLN
NLLMKNTDEN
>HeleOR7(a)
MSKNHPHDIIDVERYVLWLCGIYSGTKCSNGRMRKTSFIINYFLAFCLIV
TMLVKLVQEKNDFEVFFEIVHIFITQLACVIKSLYFYRNMAVFNELEERL
MLPVFNQHISTQKHFISRSFISFKIAAHTFRSLAWISALAYTLCPILDGE
ILAIPIWSPFGKNTPKIYLQIFESICFTWLASVHPTMDLIPVGLMSLTVA
QLEILNANLRRAADRDEGEDYDTQEKKIQERLRNCIKHHLAIKKFSLDLE
TIFSFGIFLQIFSSGVVICMTGLQFLRISPNSASFVSVVIYFATMLVQIG
TVCTFGQNMITKSSEIREACYVSNWYQCTTSTKKMLFIIMEMSKREIVFK

AGNFFAMSLATFLLIIRNAYSYFAVLMQVFK
>HeleOR8(a)
MMLKWNPFEFGFSAMKIVGACGDRVTNRTRLFRILNAIVFAQAFACGLIN
MYVTTENLSDVIDSTTSIFHCLAKYYWFLIFQPKYANFCKGDIYTYWNEA
DVSERIKEDIRLIRKNIKNLQLVVTTAVLCLATLYSALPLISSTRTYIFE
INFTVNSYALRYVILLSQYYLVFLGICVVSGYDNIYISLCFYTLIQLKLL
RGIMRNFGATVSRSTFTTCIRHHYFILSLFSRMKNLYSIIFFVQYSTTII
SICNKLRLCSDSEITYCQYIIAVIYLVYIFLQFVAYCVLAEQVGYEILQL
SDVVYSSKWYELPRYYRNEILLIMKATQNCGYFTIGGLKDMNLQACVTVF
KSAFSFHAFVNNILND
>HeleOR9(a)
MGKINCIEPLLRVLCVLTKQGLLPEKNMRLVTLILHALLFVLALFCMILN
GVNAVRTKNDTALIQATGMLIPTLNLLVKLIVIVRKKEYLTSLAYRLKSD
IFNNHSDELNKYVKLVYRISKFALICYLIICALYMSITSVLPLAVDTKTM
MIPTPFEMGKYEIIYKLLYLVIQTYFGINSVCIDFLFMSLLGICIAQLYI
LEKRLMYVFEEAEDMSKGGDSGEVDVYVESILRFCIELHKSLNEYINTLS
IILSFPLFVQYFGGCFVICNLVLQLTIIGERSTGNILNSIAYAGITFTQM
AVYHWLGNEIMFKSGNIMDACYLSQWYRLNNKSKKGLILLMERTKRPLAV
QVYKLIFISLETLGVIIRWSYSLFALIKARYN
>HeleOR10(a)
MASKQPYQYLAISVNILKFYCLWPLEGQVDALKWRQLRTYWIFISMAFSW
IPLITEIYYTFHEAEDAIIQIQVLLANICIWGMFYMAVCFIQNQNRIKEI
ISRISTFEQYPGINTLHVDKKITLYSKCFVAYAVSGMLLYCIGPWMDLSY
CEQHRSASMDAWGVPCLIIVPYRMPFRYDVSPYQQLCFIHQSYVGCSTAI
LVVTLTMLICGILMHVVSQLQNLRRYIWELRNVPIDQLDEAVRFVVKYHV
FIIDYFEEVNNSFDTVMVLHMTLTSFVISVLCFEIVMLGAVHRFITSIRF
ALHLFGWLVILFLVCYYGQKIIDESIGVAEDMYATPWYNWPVHIEKDVLF
IVKRAQKPLTLKALNIGTMSAETFLAVLRGAYTYFTLLIQFI
>HeleOR11(a)
MAKNHIYDIIYTERKVLWVFGIYSGQQRATGTIHKISFVTNLLLACCLVL
CMSVKIIEVKNDFESFFEIVHVLITELACVIKISYFYHYLPKFNTLEEQL
KLPIFNQYTSEQNRFVSQAKTNFNIVAFPFRGLAWLSVVTYSLCPILDGK
ILAIPIWSPFGEFVPKLYLQMFESACFAWLGSVDAAVDLIPVGMMSVLVA
QLEILNENLKHAAHRNEEEKYDLQEQRIQERLKRCIQHHLAIRRFGVDLQ
SIFSFGIFLQIFSSVVVICMTGLQFVIISLQSAAFVSTIFYFLTMVLQIG
MMCCFGQNIITKSGEIRDACYMSDWCQCNASTRKILFIIMEITKKEIVFK
AGNFFNMSLNTFVMIIRNAYSYFAVLVQFFK
>HeleOR12(a)
MTVVGEKLGYTSKTLKLFRFLNCGILLFLSVLVFLNVFYAERRMLVQAVE
SNVIVLHTLLKYVAFMYYKSDIEELLDISERKFWKIQHFSKEALSLKYDI
YKRARFAQLLIVGGGYAVITLFLLRPLFNHRIVFFVEPWQFKDSIVLDST
ILMLEYYISYIMVSTVFGYDAFYLAFCVDVVVQVQLLKYKLYNISEQCRG
EELEIEANRCIEHHQLLLSVFSRLKVIFSMMLFFHYFVTLVASCIELYEF
ILIQSTSFYALVKLFSVFMAFLEFGFYTFPAEQITLEFSAISNRIYMSKW
YQTQRNIQNKFLLIMTQCQRPQYFSAGAIININIETFGSVIRKSVSFYAV
LRNILSK
>HeleOR13(a)

METQNTLSLHLNVNWLKNDAKVFEDDGKHVVIFSQVIAKIINLWPCDRGI
SKTVFFGFMLATGLLQELSLIVHLLTSDLNVDVLIKAIASMMIIMQSMIK
GCVFYYKSHDLQNLIQTVWNEFWPADVMGDTIRNDIKKNSKILLWVFAVQ
YVSAVAFLFFYVILPITKPGRHLPHTSWFPFDWTASPAYEIVYVWQAYLT
AYINENIVCAYDTLFCSICGNCVSQFRLLCAAIKEIGTGNENQITQKLLK
IPGSTYRPVFSNEDKQERRLLVICIKHHQKLIGIASQLNEIFGPGHLAQL
CASALGTCTACYRITTETSFEDLVFLVAFYVAHVSQLLEYCALSHELSYW
STTLSTSAFQSLWYEKKYTDIRKCLSIVILRSQKAISMNALGLFELNYAS
FITIMRFTFSLYTFLSSMAK
>HeleOR14(a)
MPNEISYQFMSFNVNVLKFMNLWQRDKGTKDKWRYLKIFIAFISLSPTLI
PLFWEFITVFIDPTPDIVYRIQVILAEVCVLGAIYMLVCFIRNKSGIKDL
VNSIEGFKSFSNLDLKVVDQRAALFSKIVMGYSLSGMVIYSLSPLLSKQS
CENRKSQYRIDHGVPCGIIVPIRLPFPTDISPVFEFWVIDEVINGVVIVL
VVVNITMMICGLLEHAISQLKELRKFILTINEDVDIETSIKFTVAYHSAI
IQFVEGLNEYFGSQVILHFTLTSVVVSLLGFEILIVDDNRESIMYGLHLI
GWLIMLYNICYYGQLLIDESMGVAEDAYCTPWYTSPVHIQKEINLIILRA
QKPLTLKALDLGVMSHAAFLGVISSSYSYFTLLLNAKKY
>HeleOR15(a)
MSGTSKSVEAATYARRNLWILGLYHGRLLPKDAYFEARRIGMIGICTLFP
VLVLMKIFFCNMEFATLVTALSFLALLVWIMFRIPLHIGTLSKLRLLEDL
LEDSIFNIQNTRQERFIAMAKTKQEYIVTTYGWGSVSFILLFVLCPIATK
KFANVPMWTPFKSNKENIVAYIFQIFYLIVQGVAYPGIDCILSGFSNVIA
AQIGILKDNLEHSTERDPEDDYMKQERQIQKRLRMCIIHHNAILEFRNRV
EDYFFSILFFQMIFAVLQVCFSGMQLVVETNRAQQVYWSIYFAGIVGTIM
VNCWCGQQIFTESSGITEACYMSEWYTCTPSTRKMFFIIMESAKRPITLH
GGYIYTLSFDTFVNIIRSAYSYYAVFRRIYQK
>HeleOR16(a)
MLEAALRILRIISLDPVFLTSTTKLKATVFLLIDIFLETMIFASIYNDPD
FSSSIECLTAAGPILQIIVKETSLLFYRKNYAEAINIIKQFWPVNKFGET
SKKNLSNIHKITKQFIQTYYYFIFAAGTLYVFKPVFEQKRTLVVPWITFC
ELDSSLVCYAGNYLIQVTWMYKLLHGLVGFDVLFVALLSHGYCEFEQVKY
ALVHLKMNEQIDGDEPEVLNHLADLIKQHNVILGLIERIDALTANIMLYK
FIGMLVGTVSCMFLIAAEGTPQLSMVTSLGPYVGCAFLEIYIISFCGQII
SAQSESLAFAAYSSNWWLKRQPKLQKTLLIIILRAQTPAVITLGGLFNLD
LQRFVAVARASVSGFTLMQTMYLSK
>HeleOR17(a)
MKIKSFSCAKLIDVNPKIFHDKSKYTMIIGKALLYVINLWPEKYGTVSKI
SFGVMFATCATMEISLIIYLLTSIEDVNSLTKVISSAAVCLQSMTKMCII
LFNHKDLTTIIETIWYQFWPSNTMGFITETQIRIDSKRLLIGFLVVYGTG
CMFLLLLIIAPAITGIRKLPYSSWYPFDWSTSPAYEILYTIQVYIAIYII
ANTVCGYDFLYCSVCTNCIAQFRLLNEAIKLIGTGKESKLTGVLFKIPGV
SYRPTFNMEYENERRLLIICVQHHQKLVKICYQLNEIFGYGHFVQLAASV
MAICVSCYLITIEDDPNERSFLLSYYIAHVTQLFVYCAVSNELSYWSTCL
SKTAFESLWYSDKYSDIRRCLSIVIMRSQRSVSMSALGLFQLTYASFLTV
MRFTFSLYTFLGKMAKK
>HeleOR18(a)

MIITSVVIGYDTIYLSLCTHIVLQVRLLKRRLASISKCRPSDAKSEINCC
IVYHQFLISVFRRMKRVYSLMLLFHYFVTLLTGCSDLYELLIRNSDTSYM
VVIIISVLFIYAQFGYYVIPADIVACELSEVSNSIYMSNWYENSAEIQKL
LLLIMIKSERMEYFVGGGMIEVSIYTYGSVIRKTFSFYAILRTLF
>HeleOR19(a)
MSGTDRTLLNTSDFILTVWGLNPFKDGKLPKRGLILVCFMVVWTIPILIP
LVRALTDTKADIWITLMSCLQCCIKGIVAIIKRASFFKTRKIMDSFWCHD
RFGRAFREKLRTIEKAVLKVHMVYYVGGIASFLALMTKPLLARQRILPGE
RQNFCDIQASIICYATCYSIQAAVAVYVISIMATFDCMFLSFLAYGYLEM
EQIKEGLLRLSINRNRIGDEPKVLEEISSLVEHHNRALTFISKIKEVYAE
VLLCQFVISLVTVCMSEFCLIANGFPPPAHIAATYTPFLLFSNYQIFIYC
TAGEIIAGQVEFIQVRKLIFTVPILFKSESIAEATYASDWWVKNQPKTRK
ALGLIMVRAQKTYRISVGGMWELNMATLAAILKATTTMLAFMQAIYGR
>HeleOR20(a)
MANFWDFHGVADHPLKKNIENIYKYLKLLQNWYLATSIFVVTALFLRPAF
GNNTKYIFYCWVPDSITIETIALFCQYYFMAALISMTFSCDCLYISYSTH
VIIQLKLLRNRLKHVVVNSNLAEIYACIRHHQFLLSIFDRMNQVYFWLFL
FHYFVTLITGCTQLYVILLGNSDFVDLVGTIIYVIALIVQFGCWSFPVEE
IVFELSNISSAIYLSKWYKENKTVKRILLIMMMRSQNQKYMSAGGLMNMN
IDAFGSVVRKIFSFYAIIRNILN
>HeleOR21(a)
MIDAKCRDVISVKKWMFSKSRVWPVQPMGWLDLFTLAINSFFTLMVIEIT
KFWKDFAKLADMLDIALPYALYCYKLFSLAYSKKYFFSMLKVVNSITFDS
HPKYLQEPLAKTIWISKTAESKLKIAMFVLLLSCTFDLIINHNILIVALS
YGFGNYYYLMDGYEVIALILTVWNINCTDLLFISSARLGAAQIATLRRKF
AICRYSENEDVTDVKFHQKIAVIHDCIKLHISILRFLENVNGLFSDMKLV
QYVILIVPICHFVAELYFRSNRIREAIYLWEWYRFDSYNKKCIFIIMEKL
VQCSYSMLAMMKSIYTNYV
>HeleOR22(a)
METLPKIEMIYTSIQLFQFIGETKRHTSQGQLIFRIFNLCLMLFALVDVV
TNFFFIEGKLYVQTLQSFVYLFHILFKYLLIIGHKRSIEELLNDILNRFW
DHRKFNKHITQMIERIHATMTHMQNAMLIFWVLTCYIYFLKSLFVASDGF
IVESFMPRSTILGAIILISQMYSFSIGILIIFGYDFLYFALCIHVVLQIH
LLKQKIKDTIGGYSGNWSDIKICIRHHQFLFAMFLRLKHINSIMLLFHYF
STLLSTCCILVDLVLRESDFIESAASCLVIVVFIVQFSIYTFPAEQVSFE
FLGVSDAIYSSQWYSTSVVNQKILLHIIAASQKVHYFTGAGLVDININTF
VSVFKASFSFCAVLRNLIRK
>HeleOR23(a)
MTRINCIQAMSMVNRILSKEGLISSDKQQYIPMVKLILHAVVFILTFFCL
VSNGINAIKTHNDTAFVDAACTFIPIMNLLVKVIVILRKQEYLISIFGYL
KSDVFNNHSDKLNYYVRFVSRNTQAIWKLYVAVVVLFVFISTLLPFVMNV
KMVMAPPFDMGQYITLYKVVLVVLVSYAGVNSTSFDVLFMYLIAICIAQL
YILEKKLMFIYQEATQMYANRCSARSFLYEEKIIKECVVLYESLSQYVEK
LNVILSFPLFVQYFCGCFIVCSVILQITIMGEKNINNILYLALYACIVLV
QMAIYHWLGNEVSFKSAAIVNACYSSKWYRLNRRSKQSLVILMERAKRPL
TIQLFKLVFVSLESLSVILRWAYSLFALIKARYN
>HeleOR24(a)

MEEEYVMSSGLYSLDLVGLHPFKSLIRKHLVAFVFISFTLTCYGLNLAGV
ILRYDGIKSLADSIDAVPAGQQVMVKLLSVLFLKKKMRHLYNTVERKWPD
NIYGEEIETKIKDLSNKFKKLYNMYRTTIWITAVLYVSKPLILFSRILLT
EMYIPCDLSENYCYLPFVAIQTIYITDLAFAVYTFDGIFYAFLFHVYCEL
EKIKYGFATLKATTESEGDDEDVYKEFCAIVKHHNFMIKFLVDVNNVYYL
QLLNHFATISATIVFGIFFMNMDGFPPSLGQISRYVPYLMSYHFQLYIYC
TWGQEVFNQVCSVGDMLYQSKWYMKNQPKLTKGMLLVMNMSQISNKLKIG
DLWQLNLATFMKVIKTSMSFHAFMQTVYKGGEEAIMSANNESQ
>HeleOR25(a)
MLAVALIYSTTIWRMVTCNSKGFQKLIKQLRAMEREIISHKNEAIQKLYH
QYARRNYICSVGFIWVGAITVFQYHIRPIMEKLTSEPVYMSIVVNNVSTE
YKKRSLPINSWFPFDRYKYHSLSYIYQIIALTIGGSMVVATDLFFIAIMI
FIIGQLRILQYNFKCAIQFAKTLGRNIGAPYEKSVQYTIRYCVRKHQIII
T
>HeleOR26(a)
MRSIGSGIPIINLFVKLLMIVRRKEYYASLMNHLRSDIFNGHSVVLNAYV
KFTHRIIELALKCYVIIAALYLVVAVALPLVIDTRTTMIIPPPFERETYI
IIYKLLHAVIFIYMGINSTCIDFLFVLLLALCIAQLYILENRLIYVFEEA
KAICKSGSYEEIEAIEENILKFCITLHKCLNEYVNKLSVILSFPLFVQCS
CGCFLICNLILQYTIAL
>HeleOR27(a)
MDNYRRDLRQSHVLNFNLKILEMTGYWPSENYSKMFKKIYKIYTYILTSL
LALHVFSEGASLIVVAIEGNVEDLIQTSYVFFINLCCIIKIHLFTRDIKR
IKHLLTRMDQEIFKIKNYEENLIVADHEKKVALFCRLFILSGISTCCLFA
IFPFTDVNELKLPFKGRYPYQSKVFEAIFIYQILEEVFVALCNISIDCII
ICFLSHMCMQLDLLKNKIKHMERICKMELANKTKQIFHGDHRKQLQELMD
AMLVKCIVHYQTIVEMKIDFERIFSPTIFVVFVFDCLTISMTMMQLIIID
IASIQFISVVTYIFCVTMELLAYCWMGNELMIKSAMISISAFESNWIDAS
LFFQKTLLLFTTLSMQPIQLVCLHLAVSVETFKAIMHTAWSYFAFLRQKY
VQ
>HeleOR28(a)
MVGFPIWLLKLSGLWCILSEHEYKWLHLVHTILVIFICQGICLPAELIEL
VANWDFKTTMEQLSFVFTHVLNLFKIINLYINRKIISKVISDLYNFDDDN
SEECKNLKRKFHRKISILSVFFLILGVSAGLLRILVSLIYLKQCKDEYSH
MELCSSVRPFLIASPGFLRTSYFQWLLCGFQAVCWSLCACQIVAYDILFV
SLLIKTDCNVYILRYLFETIQVRSNKKVNKEYGSDCKMQLEMNTEMNSAT
KKMEMLISSTKQVSNVFQYLILLQSIFALFVLMACLYMAASVPVFSGRFA
FQAAYYFTIATQLCLYCWFANNITISFSAVPQAIFNNDWIAADQRFKTSM
IINMMRVKKPIYVKIGDVTPLNLNVLVYVLRASYSYFAVIKNK
>HeleOR29(a)
MQKHLFYSTACFLTMLALAPFPIMIITKIIMDKQDLKAVVETHYILLHFW
IVIKILLFLDKFKDVRRLENCLESETFNSHNILQDRFVYSAMQKQTRFYK
LLWSSTSTFSSMFTIFLHIEIWTPIELNHFTCHFYEVICYIISALVYPGI
DCTLTGFIANMTAQFQILRNNLENAARRDPQDEFLVQERKIRDRLRNCVI
HHNAILELVSETERIFSISLFCQILFSVVGICLSGFQFLLVSTNVEYVAT
FAYLLIMLFQIYFTCWFVQDVIMESTSIIESCYISDWYTCSTSTKRIFFI
IMERAKKPLRFDAGYFFTLSLTTFVMILRNSYSYFAVLRQVYQD

>HeleOR30(a)
MACVIISSITLMSKGITAVTNRNCNVSLLDDLKSDAFNRHSNEQNRNIQA
IDKISKHALRYYIFLAGTYFIITSFLPFVSEMKTTLPAPFFMERFEFSYK
ILHSLLILYLTSNAVCFDVYYLSILGLCIAQLLILEDKLVSLVESNTGNL
NITFNREKSLEECVILHETINQFAKKLSSLLAFPLFIQYMTGCFIICNTI
LQLTLREQTDVTDALSIIIYSVGVSSQLFLFHWLGNEITFKSNKIIEWCY
LSGWYRLNRNSQTSLLLLMERAKRPLIIELYGLVQISLDSLLAIVKWAYS
LFTLMKATYK
>HeleOR31(a)
MHPVPREQFINYFTLQKFALRIAGVSLQQTETSIYKIYSALCILLVITFT
IQESYGLIVFMNNVDLLASVISLATSHILGVLKIFVLLTNKTSFAKILNT
LEERNLERSDDTVEERLIMKCVSYTTTQSRVYWTSVSAVLGFGMAASLLT
RIKSNDWRKWEMPLAPFALFEVKSSSCFVLIYCYQSISIAVFAIIISSID
LLIVSALAHVKTKFTILKNIIRNIKTDATILDGKATKTDQSESLENLLQG
KTKYAAQYHQSILNLTLEFENYFNFLILTVFIGNSIVLCFTMYHASLNPL
SDVKTVGDLSYVAAICMQLFLYCYWGNEVTLESQSVASACYDVDFYDIPN
RLQKQLIYMIHRCQRPVIFTAGKFINLSLTAYVSILRVSYSYYMVLRSKQ
DVE
>HeleOR32(a)
MMVPLLKYPNKQAENYFHLQRTVLKLLGVSFSPNESFLYRIYSIVWLSSV
VITFSIVELYEIYVVYRDNMDVLVNNLSYLGTDLLGIAKLTLVLYHRIAIG
NMLDNMETSSLLSNRLKDDGNSEDNSIKHCILLSNRQTCLYYLSVIMVTG
IGALGTMLKRIFHSNAKTWEMPFMTFSWFDTNSSPSFELIWFYQFWWRTF
YALIVSSMDCLIAGVLAHISVQCEILQNRVRNVVADTRKQITLELAEPYA
TIPRNILAKKTKELVSYHMAIINLADTFEELFNFLVLTIFVTTLFILCFV
MYHASLFDLFSIRAAQDFSYVALIALQVFLYCYWGNEVRLESEKVADACW
QMDFLQTDIQFQKSLVMMIRRSQKPIILTAGKFTNLSLETYVWIIRLSYS
YYMVLTNYHTES
>HeleOR33(a)
MFPKYLIKTQVERMPELRKIIDFLSIGGWKSYAWLSFATFKIMCVSSQTK
FGIDVSRYDFLLFSYAASTYFIAILGLSKLYSWSKYYGKIQEMLLVIKQK
FFDINIVNERLSRRLNLRLWTISKIVVVQVCISNFFCLVVAIFPLVNFPD
GRKKLPTAVWIPFDVDSTPTYQIVYIVLTISNHVSVLINMTFDSLYVYLT
QTLSVQFVLLKYLLKNITEVPDIVGPYRYESVKYQKILEDRLKICIDHHD
LLMRFSNDIEQIYSFCLGVQLALSILSLVFTSYILAMDHCDVIKILKVLV
YASALTIQVLLYNLNGTEIESEHDNVAIAAAQCDWYYGRKSFRDGLLFVM
RRSQHGFVINALGLAAVNNACIVMIYRTVFSTLTVIKKVTQ
>HeleOR34(a)
MENNDVHLRINMKCMFYFGAFRFRFDNWLADGAYRSYSYFLKSYFVMFVI
SQYTELITMSDKDLFNVISILAVSLLYTTTVWKMAICNGKRFQRLVTQLR
RIETKLLSYKNKDIEDIYYEHVKKNYFCSWFLLYGTITSSLYYIRPIIEE
RSRDPIYMNVTKNNITLQYRVRPLPLGSWFPFNRYKYYYFCYIHQIFATM
IGGTMVVLTGLLFVALMIFIIGQLKILQYSFKNATKLAQNLTIRSHISYR
KALRCAIQHCINEHKMIIQYVKDLDKSMRRLMLIDFAV
>HeleOR35(a)
MFIILNIRKGIKNRDVKLTNWMTCVLIPIFICATKQFAIVANKKCLHSIL
EDLDSVTFNAHSEKLNRHIQFINRLSKLISKFFVIAACLFMVAFCVLPIV

INIKLMIPPPFDMGRYGTIYKLMHLLLSFYLSLNMICLDSLYMTLMAIAT
AQLIIMRERLITAFESVNNVETRKMECGLCETNITAEQILKECAILHVTI
IKFVKKLNVLIAFPLFVQYASGCITICNTILNLTIMGGMDTLSIIGMVGY
ANVVFMEIAIYHWLANEIISNSDKIGESCYTSKWYESSAFCKKSLILLMQ
GSQNPLVIRLYNVIHISLDSLVVVIRWSYSMFALMKATFN
>HeleOR36(a)
MIVLIVSMVKSSFVFMGSISTAFDVLYMSFMALCGAQLDILKERLKNVLE
DARELYEDKSYRRRFISVNEVVQNILKECIILHEVINRFTKKLDVLLSFP
LIIQYVIGCFIICNTILQLTILGNPNSTNIIGLCGYSGVVFAQIAAYHWL
GNEIFFKSDDIIEACYLSNWYKLDLSFQKCLIILMEKAKRPLVITLYKFV
FISLASLGVVVLSNICGAHVLTLLFLDCSM
>HeleOR37(a)
MVGIWIPDSKSTFPIKLAYTVYNTIWVIYSCLIFCPSEIIYFAQSFSKLN
DLVKNMNMAMTHFLGDVKVCMWFYYRQEIMNIIKTLGAYEKRYESYRNFD
TKKIIRKAKRFKDVFGLSFLSFGIFTAISSCLLSLLPIINAEVSDNNPTS
LKLPYYSYIPFEYRSSRRRFIMALFYQIFPTMNYGFIIIGFDTLYTAILG
YVSCQLDI
>HeleOR38(a)
MDHFFDVNFTMLKMSGIWIPDMNSKLSIKLRYLTYNVLWITYSCLIFCPS
ELAYFANTFTNLQDLVKNVNMGMTHFLANSKVFLWFYHRKEIMSIIETLG
VYGRRYENYGDFNTERIVQKAKKFKDIFSVLFLTFAMFTSISSCLICSIN
VVAMDLPKGGEIDLKLPYFSYIPFGYKRSKVLFLIAIWYQFFPVFNYAYI
IVGFDTLYTAILGYVSCQLDIIKGAFETIRPRCMIRLRLKLPEDILKDPP
PLMDEMHKEMNKVVNHLQVLLNICRRLEEIYTNIILVQVMISLIVFCTCI
FLVSLLPMGSFNFAAEMIYLIAIECQLLIYCVFGNKVTVSSNNVSSSIYK
GDWYSASTSFKRSMIITMSRMQKPIYFTIGKFTPLTLSTFVTISRASYSF
FAVLKNRDVST
>HeleOR39(a)
MSCAMVVILFTIYPLFDNKSLPTPIPINLGKYTFVMYIIQSLALLIAAWN
NFCLDTLCISLMGLAAGQFDILKEKMLHFTKYAENDIPGGTSNDLISKSM
KLENTANERTKNSLNNCIAHHIALIIFIRQIENVFSFGLLAQLIGSIIVI
CNTGFFLLLVSPASLQFGMLSSYFITMMAQLILYCWYGNEIMLKSTHIGE
CCYLSEWYMCNLAVKRSLFVVMERSKRPLAITALKFSKLSLTTFTSIIQW
SYSYFALLQRLASKIENQA
>HeleOR40(a)
MEQEFVTAFGLRSLNLIGLHPFKPSVIGHVKSFIFATITITCYGFNIAGV
ILKYEGIRSLADTIDSVPSGQQAVVKLLSTIFLIKKMKRLYTMIEQKWPD
NIYGEKLENTLNLLSQGFKKFFSAYKMTYFITGMLYVSKPLVLFSKILIT
EWYIPCDISNNYCYAFFLIAQNLYIGILAFVVFTFDAIFYAFLFHAYCEL
EKIKHGLKHLKIANTKENEDSVYEEFCNIVRYHNFILKFIERLNNVYYLQ
LLNHFTTFVATIVFGIFFMNMDGFPPSANKLSRYIPYLFTHHFQLYMYCI
LGEMVYDQVYNVGDMIYHSKWYVKHQPKLTTGMLMVMVVSRAKNKLTIGN
IWKLNLATFMQVIKTSASFHAFMQTVYLADDNV
>HeleOR41(a)
MVKINCTEPVSRVYGILSKEGLLSNDTLQNTPIIKLMLHAVFTLIPFCYL
VTNGVTAIRAGNDTALVDATCILLPVANLLVKVVVAVRKRNHFASLLHCL
RSDIFNNHSEKLNYYVRFVYRITELIMKNYAAAITIYVLIAAILPVIFDT

KMMIPSPFEMGKYLILYKFLHIAICVYLAVNTACFDFLFMYFLGICIAQL
RILEKRLKYVFEEANQMHGKSSLDEVYLYEENILKHCIRLHESLSQYVDK
LNIILSYPLFIQYFFGCFIICNVILQLTIMGVRNTNTIFFFGTYGFVVFS
QMSLYHWLGNEITFKSAGIVNACYSSKWYKLNKRTKQSLVILMERAKRPL
TIQLFKLVYVSLESLGVIIRWAYSLFALIKARYR
>HeleOR42(a)
MSALPQEQIYETGVTLMKLMGEHMHYISKRLICFRVANCGVMTLLLMLNL
TNFFYVDSGRYTKTAETSMVVIHPLFKYVLFLCYKADIDQLLCDKREKFW
KYQDFNNGIVATYNKLFAVIKLIQVTVVGGAYAAVFLYALRPIFGGNAPI
LETSVFKDSVAWESVILMSQYYLFCFIVSMTFGYDALYLAFCIDIVVQLR
LLKFKLKQLTENTTNSITAEIRECVEHHQLLLLVFHRMKNIYSVMLLFHY
FVHLIAGCIDLYELILGNLTFSQAMGELLN
>HeleOR43(a)
MSNFFDLNFMILKLSGLWVPNYENRNYKAVMAYNSFWIIYSMIYFTIAEL
IALRESAENLSDLIKNLNMLLSFVLTLVKVIIWFWNRKDILGIIKILGRR
ENIFRDDNLDCDSIILKEKMFKNIWTRSFFIVSSLVPLSAGILSITEAIT
SGIKYVEIHTDKGYVYTQKLPYYSWIPFDPTSSKFAYVIAVVSQCLALLN
CGYITVGLDMLFVALISLVTAHFTLTKRALNGIRNTCTERLKRQTNNSQI
GNEMLTSECDNEIKKCVKHLQVLISLCQKLEVIYSPLVLTQVLISLLVLC
TCLYLVSSIPMGIRLLGNELAYLLAIEIQVAIYCYVADKLTHCALKVPTA
IYESNWLSTSSGFKQIMLITMMRMQKPICITIGKFSPLSLGTFVMIAKTS
YSIYTMLKSRN
>HeleOR44(a)
MLVKYFGAAVGLFVLIFSVLPFVTDIMMMIPASFNAGKYIAVYKTAHLFF
TIYFGTNSVGYDILYMSLIALCGAQLNILKERLINVYIDAKQHQRNGSQK
SIQSLLEDILRECVILHDTINRFVKKLDILLSLPFFVQYVVGCFIICNTI
LQLTILGERNSTNIIGLCGYSAIVFAQMTAYHWLGNEITFKSDKIIESCY
MSKWYKLHLKFQKSQFLLMERAKRPLSITIYNLLFLSLASLVVIVRSSYS
VFAIIKARYK
>HeleOR45(a)
MEELPKIEMINASVHLYQFLGETKRNTSRKQLIFRIWNTCIIVLVLSFII
TNFFFIQGELYVQTFQSFIFIFHLLLKYLLIIHYKPRIEELLYGMLQSFW
NVTNFNANVARGIKRIYRIIKYLQIIIVTIAVATAHAYLLKSLVVSNSGF
IVESFMPRSIILDAIVLSSQFYCFCIGVPVAFGYDFTYFALCIHVVLQLR
LLKVRIQHILSACSQEWSEMKICIRHHQFLFSMFLQMQDIYSLVLLFHYF
GTLIATCTALLELFLRESNLANYTANCPIKSITDNIFAA
>HeleOR46(a)
MEMYKAKFFRINYIFLKYGGLWTPQKQTFDYKMYKIYQSMVLLVVLVFCS
YSTGRGVIDNLSRFSILIEVSSVAITIFLSETKIIFWLKNSDRLKNIMNT
LEYNEFCYDKIDDFDPKLILKKSKRIGTIYVLTLWIFAEFTIGFESIPSC
IASFWYHFNDIPISNVSTFETLPYNTHIPFAHDTAIKYLLACLSQYFMYQ
YVIMGFVCVDGLFQNLLNLIGEQMIILGGAFRTLRRRCLRKISGPPLTED
GLHNTEELEELMMAEMKKCIKHLQMLFRCCEEIEEIFKFLTLLQTLGSLF
ILCSTLLLLSTTSILAKQFGKNIFYLVGAAIQLGLYCWSGNELTLKTSSV
LPALGEVEWLEARKPFKSCVLITMMRLQKPMRLTAGDFAPLTVDTQISVL
KGSYSYYTVLKTRNASTN
>HeleOR47(a)

MVFGPGKKRGIFNFSLFKGDEMFKDHAKYTLLVSKFVLNLVNFWPENYNT
STKISFYTISAIYVLIQTSLIVFLATGIDGVVTLTRTMSTLSLGMQNTAK
MNILYLRSKELEKIIKQIRFEFWPSNILGEDMEKKIRSESKKLLITYLIP
YVGAAIFLTQIILYPILNGVRVLPYNSWYPFNWSQTPTYEIMYVCQAYIT
IFVNENVVCGTDFLYCSICANCTVQFRLLCDAIKQVGKGKEDELTDRLMQ
FPGVTYEAVLNKEYAKERRLLVLCIKHHQKLLNICSKLNSLFNFGHFYQL
TASMTGLCAVCYLITTENSANGQSLLASYFIAHVTQLLLYCGVSNELSYW
SEILSVEAFQCAWYNQKYSDIRQCLSILIKRSQKAVSMRALGLFELSYAS
FILVMRYTFSLYTFLDNFAET
>HeleOR48(a)
MFLATGVEDVKALTDAIASLSCAMQLLIRTTVALYKSHRLNTVIKKIRYE
FWPSNVAGSNIDRDIRKYSKKLLNISITEFCFGGMFLLLNTVLPFTKPTR
QLPHSAWYPFDWRKSPTYELLYGFHIYFNLYLNDSGCVWYDSLYYAICSN
CIAQFRLLCASIRYIGTGKENEIIEKLSKTFGANYGVRTGRKERKLLMVC
IKHHQKLIHVCKELNRIYSNGHLGQLLTSVTGLCTACYRITVETSFNTIV
FLLFVNLAFLLQLFLYCAISNELSYWSVELSTAAYESLWYKENCAEIRGY
LPMIMVRSQKEISMSALGLFSLNYVLFVSVMRFTFSLYTFLNEMSK
>HeleOR49(a)
MVGECTPHPSVYLRVFRSINIIVLCVLFMFVTRALFEREHKSITRIMEGF
VAVIHPLLRYLMLIHNMARVKRLIGDGSGGFWDTQYFNKSFIAHEKRFLK
IVKSVQLLLSVTLASSAVVVMLRPLYDENRVFIFETCSVDSVIINSVILA
LQYYLVLIAIPVLVGFDCLYLTLNVQVIVNIRRLKYKLQNLPVRSSDYPR
ETLIYCIKHHQFLISAFSRMKETFSLTLLFYYFVTLVSSCSELYELTGNG
SYDIGQIINIFLLFYQFGYYTLTADQVTTELGDLPTSLYLSKWYKNSPAF
QKLVLLMIARSQVNKPFSGGGIITMNTDTFGSVIRKSFSFYAILKNVLNK
>HeleOR50(a)
MADFRPKLVVLDSFTPYLRVLTAFCFFAHELDRYKIIYKIYSSLILIIVQ
LLPLVLEVIYTIGSVKNFADIVKIPLYLPTDIIANLKLFYLLRNKKEIKD
ILAYTNLEVFQPRNARHEGMLDSAIAIARRNIRLFLCVSILSTTALSLTF
LTNLSGKTAPLSMWLPLNISKSPGFEMGHTYQGIVNLLHATSHVSMDSIP
LLLMSFICGELDVLADTLSNLRLYAREESNDPESIASVHEKMDKLLTECV
DKHRHIRSR
>HeleOR51(a)
MTNLREKFASLRTKFLKDNSQILDDGAKYTLLVSQIYGNIVNMWPGNVNK
TKEVYATVMLLICLVYQISLLLNLAINSDNIESFTEMLIMTVGVLQTTAK
VLVLLYKSKKLKHLIEIIWYEFWPSNLLGEDFEKILKRESKILLIVFLFE
YCTGLSYLVLLVLISITQPIRQLPHSAWYPFDSTKTPAFEIVYAFQVFFL
VYLLPHVIMCYDTLYYSICGNCVIQFRLLCETIEYIGTGREDEIVDKLMR
IPGVSYRPIFSRKYEKERKLLVICIKHHQKLLSVADQLNDNFGKGHLVQL
CVNILCACAACCGIATQHSINDLSLLLACYFSQISAILLYCAVSNELTYW
STCLADAAFQSAWCNKKYKDIRNCLSIIIMRSQRAISMNALSLYVLNYTT
FLAVMRFTFSLYTFLRQMAIEE
>HeleOR52(a)
MQSERFVRYVTLMFRILAFSPLKDSDSFYYRCWYYFYRTVSITSGSTIIA
LTLIEIQLSISAGMRFSDYSKAVVVLAVYISLASKTNYFYIFQKKHVELT
VRVKHLFRTETNTKLVERHFSDYARNADVLSAFWVIATMIGTQIFMLIPV
ILTGEWLLPLKIWYPFDEDSSSLYVSMYSFQMFVHNLMVFNFIITDMFFI

TINVITSSQFRLIAWECEYMCYAALLNCDISKEDALAFKSNSEERSKVAA
PIYQIMQSTTYTAHLNKKFIDVIAFHRDIAKFCIDFENFISPVMLTTTLA
SIFHTTIIAFTFVTATTLSDLVSSVSYVVLTLIHIFTINFVGQQIINHSS
QLEHILYDCPWYLCNQKFLKLFKFVHLRSHECAFITIGKFAPINLQTFMW
IIKSCVSYLTILRSMDQKKV
>HeleOR53(a)
MIRFSILRLKTFIMQQLHKRESIQIAITLLRFIGENIQHTSWKTVLYRCI
NSAILILSGIFVAGNFIDANGSLVVNTLESLISVVHVQFKYLLWFYYKND
FKDILVERLTRFWDYRNFKGSVVIQIEHIYRSISAAQLIVLVVWLICAEL
FFLRPLFSERFVYIFDAKVFIDSVTLEVVVLICQYYFPCIGVPIIFAYDS
IYLSLCVDIIVQVKLLKYRLKCISSLTNNETTIEEVVACIEHHLYLIT
>HeleOR54(a)
MSKLPERDILQFSLDLVKIIAENINHTSKKVYVYRTVNVAILLIVLFFTI
ENCTKEEGVSLVKNLESAITVTHILAKYVGFLYGKTRIKTIVDDTLKFWH
PNSINDSGAKHVNKLSRFTKILQKTLLVSLIICAYLHMLKPLFNKDDVFP
FNAWIWYDSLVLEVVVLVMQYYCICIATPMIVTYDTTYFGICMHIIIQLR
LLKCKILQVSSKRQIPLSACIKHQQLLSSIFMRMEEIYSWMLLLQYLISL
GTACLQLYILSTGKTNAADTIELIVYLTTLYCQLGYYSISIEVISSEFSD
IANAVYMSSWYETDIRTQRTLLGIMISCQTPKYLSGGGLIPINIDTFGSV
FRKSFSLYLILKNVM
>HeleOR55(a)
MEELPKIEMINASVHLYQFIGETKRHTSLWHLIFRTFNTCVLVLVLSFTA
TNFFYIQGELYVQTFQSFLFMFHILLKYLLLIYYKPRVEELLRDITKRFW
NYRRFNRQVIDATQKIYRMIKYMQITMLISVVSTVYTYFLKSLLVTSNGF
IIESFMAQSSALNTIVLMSQFYCFYIGVPTVLGCDFIFFALCVHIVLQLR
LLKQKIRDTVNKYSQELSDIKNCVQHHQFLFSLFVEMADIYSPLLLFHYF
ATLVSACAVLVEIFLRQSNLANYTAKILSILIFTGQFALYAFPAQQVTFE
FLDISDAIYSSRWYSINVLNQKMLLCIMGAGQRVHYFSGAGLVNVNIEAF
VSVFKASFSFCLVFTNLITE
>HeleOR56(a)
MEKHRLHRTNSIVVIFVLVLFPLLILIEFALHEQDAEIYLEILHYLLVHI
WMIIKLFIYLSYRSKLADLEDLLDSQELNVQTKQQDCLIAKAIQHQKLFA
GSLLCMGWMFTITFALLPIFSDQILLVPIWMPLDLGELFWHMYESFYYVI
LVCIYSAIDAKVTGYVAIVTAQLEILSDNLEKFTERNADEDFAVQERKIQ
SRLKNCVIHHNAIIKFIRKVEQVFSLIVFYQVLFSVLGICLTGIQFTTIL
TGSIKFASECVYLLGMLFQIFSPCWFAQNIIIQSAGLVQCCFMSDWHICT
PTTRKIIIVIMERAKKPIVLRAGPFLTLSVNTFVMILRTGYSYYAVLNNL
YPK
>HeleOR57(a)
MQSDIIYYERRMLWIFGLYGLKQGKMSSILLEARRILTVGFAVTMLLLMF
VYMFQNKHDLFTVLDTSYYFMVETGFVIKLHVYFYYLPILRELEEQMKSQ
IFNCYSKYQQHYISDALKFHRTIAGLYQLCCVSTAAFYSLFPALDGQSLA
VAIYSPLDVERYRTLLYFYEVCTFFLTAISNSALDSITVGLMMITAAQLD
ILKDNLQHSTHRNPEDSFPIQERRIWRRLRHCVIHHNAILEFTNTTQKIF
SAGVFLQIVISVIGICMTGFTSLTVPIGSMKFASLLLFLVTEILQIGMFC
WFGENIFTKSAEIGQSCYLSEWHRCGATNQKMFFIIMECTKTPITFKAKD
LFVLSLNTFVMVLKSGYSYFAVLRHLSEE

>HeleOR58(a)
MTKINCIKPLSRVSRVLVKEGFLPNKAARKATQTIKLILHSVVWANIFIL
IVMNGINAVKAGNGSALVDITCIFIPATNLCVKIVAVILRREYFFLLVDY
LRSDVFNNHSEKLNRYIMFVYSISEMLWKYYVFSIAVYAAIVAGVPLVID
TKLMMIPPPFEMGRFLLFYKCLHAVLTIYLAINSICFDVLYMTLLSLCIA
QLYILEQRLINVYKEAKKMCKNGGSGQMYLCEERIIRICVVLHENLTQYV
DKLSTILSFPLFVQYFCGCFLICSLVLQLTIMGERNTSSTVSIIVYGVIT
FSQMAVYHYLGNEIIFKSGNIIDACYLSQWYKLNNKTKKILMILMERAKR
PLAVQLYQLIYISLESLGVIVRWAYSLFALIKARYN
>HeleOR59(a)
MATETKRLLIVPDAILSILGINPFKGGTVTKRGIVFLSCEILFLINVAFR
IASALYEADADIWDNCMSISQGVIKATTILIKRDLFFQTRKIMIDFWPYH
KLGKKIGEKLEKIETGVYELLQVYYVVSVFAYLAFATKALFARQRILPCD
WYNICNIQESILCYAICYSFQVAFATYSMLIMSAFDCLFLNFLTYGYMEM
EHIKEALLHLSINKKREGDDTEVLHEISSIVKQHNRTLKLISNIQKTYAD
VLLCQFAMTLVTVCMAEFCLIIDGFPPPPKTLVNYIPYLFWGIGQLFVYC
KTGEIITEQSGSIAYAAYESNWWVKNQPRTKKALRLIMVRAQKTCKVTVS
GIWELNMATFASIIKATTTMLAFMKMVYGR
>HeleOR60(a)
MASQDVTNNYFKVQIFCFKIMGIAIEDYDDSIIGRLYTAYSFLILSTCVY
LFLITEAIDLFLKWGDLDNMTFNMCYLVTHLAGLIKIVVIMYLRPKIRKF
YKSLESGYFLPNYDRGGVAEYRIISEAVRQTNMQTYIFYTLVTVIVANRA
LYAAFDKGTFEAVENASNNETTIIHYQLMPYTTWVPVNTNISPYYEIVFA
YQVIGALIYGLLIGTSDSSIAGFMVHIKAQLLILNNSLRKYVQRAKQNAE
VKMKETLEDRIQNNGIYIGLRKVDKIEKLNPAIQVLVMHYLRECIIHHQE
IIGMTEVLELEFNYLMLVQFLGSLMILCFSLFQLSTNDIKSIRFFSMVCF
ACLMLFQLLIFCWNGNEISVESLETAHAAYDSDWILSDTETQKALVLIIQ
RAQRSLKISAGKFAYLTLETYMSILRASGSYYMVLRSVNE
>HeleOR61(a)
MAMRLNKFKFHKENAEFLKDGAKRTLIASQVLVNLLNLWPVNVGKYKNVS
FAILIAVCFIHELSLFVSVLTSIEDVDAFIKSISSLTIGLQVMTKAITFF
CKSNELKTVIETISHQFWPSNLMGTRMDKNIRQYSTKLLYIFLTQYVTGI
GFLLMYIIIPITKSTRQLPHPSWYPFDIYKSPAYEILFLLQSFLTIYVIQ
NIVCSYDYLYCAICGNIVAQFRLLCEIVKYIGTGRENEIIAVLMKIPGVG
YRPVFNKEYDQERKLLVICVDHHQKLIDVGNQLNEIFGIGHLMQLFASVL
GICTTCYRLTTETNLNNLVFSISFHVAHVAQLLTYCALSSEVSHWSTCLS
VEAFQSIWYAQKYTEIRKCLSIMILRSQKAMSMNSLGLFELNYVSFIAIM
RFTLSLYTYLGKIAN
>HeleOR62(a)
MKFLAKSDIYEVGISLMQFLGENLNCTTPGIVLFRIVNCAIIAFVLIFIH
ANFIHAQGEMYIDTLESWISINHILIKYVLFIYYKADLGNIMNIRLNYYW
SYRKYGNNLIETVERTYNFVKSTETFLLILTVFLMYMIFLQPVLYKNTVF
LVKTWVFFNSIPFEFVVLICQYYIYVIVIPIVVGYDIWYMTICIDLIIQM
RLVKHSLQHVSDKSVEDGVKQITATVQHHLILLSVFSNIKRIYSITMLFH
YFVTLITACTDMYELLVGESSFVNCLLKIPTLLFIFGEFAYYAVLAEQVS
FEFSDLSNAIYMSQWYEKDIKIQKLLLLVMVKSQRVEYFTGAGLMDINID
AFGSLVRKTFSFYTIIKNLLNK

>HeleOR63(a)
MSESNLQTIDKDYFKMHFLVMRLFGVSIRPVQNPIARMFYQIYSVIMFLI
VYVYFPIAEILYIAYNANLENATIGITYICSHTLGTLKTVIMLLYRKKIS
SFCEHIETKPFTPDQNRGGTVEFNYVKDAIKVTNYQAYLFYGFVVGVIIP
KIYNILSDSGFDNQLLESFENATLVKYTRTGPFNCVMPFRIIDSPYFEIA
ACYQATAAAILGFVIGAEDAIICGIMSHIKAQLLILKNCLNTYVQRGIYL
MKEDNINDSNSGGEVGNFPKFNEDSSGAQKISFVLQKYVDISVNDIIKHH
QKILDLAEDAEETFSLLMLVQFLFSLSILCCQLFQISIMKPGSAQFYSMC
FYILLMLFQIFLFCYRGNEVMLQSYDLIDAVYESNWPAIHCKTQKSLLLM
MIRACRPIKLTAGKFVFLSLEAFVSIVRGSGSYFMVLRSVNTPQE
>HeleOR64(a)
MKEYVPDFFYTNALFIKYGALWPPTGVNKIARKLYTLFKIVVLFLTLGFS
TFSVLMGVLVHIDDLSIVIEVLNVGVTILLGGVKTSFWLNNEDRIKRIME
TLETAPFHYEEIDNFDPRVMIQEAKTIGIVFAWLLMVFSHLVLGIAYIPI
ILISLWYYFNNLPITGIKNFETLPYYIYVPIEHNTPMKYICACLMQVIPC
YLYVNAFVGIDSLFMNMMNLIATHILVLQGAFRTLRRRCLKKLNGPELTS
DGLYNSDELEEYMMDEIKKCVQHLQLLFGSCKDIEEVFKYVTLAQALGTI
YILCTTLLLLTTTPVMTKEFGKNFFYLIGVVVQLGLYCWFGNQLTLKATN
LPLAVWESEWLQTRKPYKICMFLTMLRLKKPLYINAGQFVPLTLETQIAV
LRGSYSYYSVLKGMSK
>HeleOR65(a)
MHVQQKGLEKLSLEFTRSLRKDEIKRYLLPAKILLQCVCNWPDSEAVCMK
IAGWFLFSALFLTEIFHAAYVLKNLDNISAAVSAGTTVTTTLEALVRLYI
MLSKKHAINGILVKVWKEFWPTSIIDRKLQNRMKTKVKISFILTSIFFIT
AVSSNSFITSVPFIQDHQLILRSVFPFEWNRDYVYEIMYVWQYHADWYVL
FMINAFDFFFVSLLTICVVQYVILQQVMCCILGKETRGHRLVIFGDIGGC
MTHKEMLYRCVEQHKMLISICTELEDSFNIAILFQFVVSTCANCAAFLLL
KIDASQFIKMLLYAICHVTQLFYYCYVGQQLLNESGNLADAVYRCDWHLA
YDRDFRKALTMMIQRSQRSQGLTAMGLTELSFPSFIMILRLSFSFYTLLD
NVFMKNGGQ
>HeleOrco(a)
MMQFKPQGLVADLMPNVRLMKFSGHFMLNYYADNSGAVHTLRLGFCFGHL
FLLLLQFGFTFGNLVKESDDVNDLAANTITVLFFTHCIIKFIYFGVRQKL
FYRTLGLWNQSNSHPLFLESNNRYHQLALTKMRRLLIIVMIGTIGSWIAW
TTITFFGESVHTRKDPSNENETITEEVPRLLVRSWYPWDAMSGVPYYITL
VYQVYYVGFSMLHSNLLDSLFCSWLIFACEQLQHLKEIMKPLMELSATLD
TYVPKSADLFRAPSATSQDNLIDSDYNQRNDEANLKGMYTTHQEMGVTYR
SSHLQEFSSGGIGPNGLTKKQELMVRSAIKYWVERHKHVVRLVTAIGDAY
GIALLLHMLTSTITLTLLAYQATKIDGVNKYALTVLGYLFYALAQVFHFC
IFGNRLIEESSSVMEAAYSCHWYDGSEEAKTFVQIVCQQCQKAMSISGAK
FFTISLDLFASVLGATVTYFMVLVQLK

IRs sequences

>HeleIR8a(a)
MGCISFLLSISAIVHCSAAESTTISFILLHEQDQFSFVTWFEDALKFIED
LKKGTIALDMFAIEADDNNSTIDKVCQGLSNGGLMIVDVTWIGNEHVLAL

AKDMNLPYVRVDLSISPFLKLLDKYLDERNCTDVALIFDDPIQVDQALYY
WIETERGRMVITDTLDRTSARRLKALRPIPNNYAIIGTTANVLKLFQLAL
EEHLFQLPDRWNFVFLDFEYKQTFSQFLRYESISLLTLDEEICCLLLQTP
NCVCPKDFLLQKHFLTNVLTILVNTIDILIHESKMLEPVLCNNTKNDKEV
LVRFTDILSSNIDQDKTIEETNNSLRLQVGGNVDMIESSDISRLGTYNDA
FGFSLENGKQIRPIRAFYRVGLTQAIPWSYKVNDASTNVTQWNGYCVDFI
AKLAERMDFDYELIEPVRGTFGEKDKSGEFDGVVGDLQKGETDIAITALV
MTADREEVIDFVAPYFEQSGISIVMRKPVRKTSLFKFMTVLKLEVWLSIV
GALIVTGFMIWFLDKYSPYSAQNNKKAYPYPCREFTLKESFWFALTSFTP
QGGGEAPKSLSGRTLVAAYWLFVVLMLATFTANLAAFLTVEMMQTPVQSL
EQLAKQSRINYTVVESSDAHQYFINMKNAEDTLYRLWKELTLNASTDETQ
YRVWDYPIREQYGHILLAINDSIPVMNASGGFEKVNERMDADFAFIHDSS
EIKYEISRNCNLTEVGEVFAEKPYAVAIQQGSQLNDEISKKILDLQKDRF
FEYLQSKYWNHSQKGECDNSDNNEGITLESLGGVFIATLFGLALAMITLV
GEVIYYKRKRANKVNPPNKTGQTIKTISVKPTIPDSITFGTTFKSTDFNK
DNRRISHLTLYPRPRSRIN
>HeleIR21a(a)
MHERRALQKSHQKPQIDVFLETFLLTNQDEESITVADLIIKIFEKYLSRC
VPIILYDQQNLRESHIILENIFKRINASFLHGTVCEYNADIKAKLIKDKL
ETGCYSYILLLYDIFLSKHIGRQNTNNVLIVSKASQWRVNEFLATDASRS
LINLLVIAPSTNTIIRGDELCYVLYTHELYVDGLGASAPKVLTSWRNGSL
TRSEAKLFSQKMKNGFAGHRFITSVAHQPPFVIKRGLDENDEVIWDGMEI
RLLKIFSELYNFTVDIRVIKDESNKSPADKVTDNVSDGIVNVGVSGIYLT
QDRLAKVDVSYPHSYDCAAFISLTSTALPRYRAIMGPFHWTVWLSVTIAY
LFVILPLALADRLTPKRIWKNPEELENIFWYVFGTFTNCFTFGRETWTKS
PKATPRLLMGFYWIFTTIITACYTGSIIAFITIPLYPATVDTVKQLLQGR
YRIGTLDKGGWEYWFKNSTDQASQKLLKNLELLPDVQSGLRNITRAIFWP
YAFLGSKSQLDYIAQTNFTTNSKRSLLHISSECFVPFGVSIVFAKNSVYK
DIIDKGISRISQSGFMIKFERDIRWELVRTATGKLLQAYSPTLKMMTLED
RSLTLDDTQGMFLLLGAGFLLGFISVIFEAAGGGFKCYKKPTNTPHSIAS
NPRIHDTPTLRERYDSIEADHDMIVANGHEIEVGIYRNKSGRPYLRKRNR
ETKFGTKHHFENFFGEDTSIVRNDLSLIIP
>HeleIR25a(a)
MLGLLLLTVLILDFCSKLNSQTIQNINVLYTNEEDNDVADKAVDVALDYV
RRNSRLGLKVDLRRVVGNRTDTKGMLDALCKTYGTMLENKALPHIILDNT
MTGLVSETVKSFAKALQLPTVSGSFGQEGDLRQWRDIDDSQQDYLIQIMP
PADIIPEIIRTIVINQNISNAAILFDQSYVMDHKFKSLLQNVATRHVIRP
IQEPTLIRDQLQNLRKLDIVNFFVLGSLISIKKVLDAADSINYFNRKFAW
HCITQDKGDVKCACTNATVLFAKPLVDNKYQDRLGLIKTSYQLSSQPEIS
AAFYFDLALQAFVAVRNMINDGAWHNNLTYITCDNYDGKNSPNREGFNLK
KYFAKDTTESPTYGAMHIASNGQSFMEFSMQLTAVSVRSSSSDKSVNLGT
WRAGFNNNISLVNPESMGNYTADIVYRVATIVQAPFIMIDEEAPKGFSGY
CIDLIDEIANILKFDYDIFLVPDGKFGNMDLKGNWNGVVKELMEKKADIG
LGSMSVMAERENVIDFTVPYYDLVGISILMKLPKTPTSLFKFLTVLENDV
WLCILAAYFFTSFLMWIFDRWSPYSYQNNRDKYKDDEEKREFNLKECLWF
CMTSLTPQGGGEAPKNLSGRLVAATWWLFGFIIIASYTANLAAFLTVSRL
DTPVESLDDLSKQYKIQYAPLNGSASMTYFERMANIEAKFYDIWKDMSLN

DSLSDVERAQLAVWDYPVSDKYTKMWQAMKEAGLPNTLEEAVDRVLSSRS
SSEGFAFLGDAADIRYFELTHCDLQMVGEEFSRKPYAIAVQQGSPLKDQF
NTAILQLLNRRQLERLKEKWWSNNPKAMKCEKQDDQSDGISIQNIGGVFI
VIFVGIGLASITLAFEYWWYKYRKTAKVITIQDTTLRTVPKQKAIPSTSK
TLAPSKLYLRSRF
>HeleIR40a(a)
MMLVYYFMCCISAVICCFNTGKSNYIPSKNDSQLAAAIVDIAISLPTYEL
AIVFDSTADKDFLNQLCNTLSAKGISLLVFNASTVSKLDKYLDFLASRVQ
NYLSSYALYFVNDKLAEHLLLEISDSNFIRRNIYYIFYCGENRIQRDFLR
NVHEAMNIVIITHPRTDAYRVYFNRATSYRRHHFTMVNWWTNEKRLFNHP
TLPSSLEIFKDFKRKLIYVPVIHKPPWHFVLYKNDSFEVLGGRDDKLLRL
LAEKLNFRYEYIDPPERIQGSSFSINGSFEGVLGSIWRREVEFFLGDVAL
TWERSNVVEFSFLTLVDSGAFLTHAPDTLSEAFALIRPFHWKVWPVIIFT
FLISGPTLFVLITIPNLWHPKFLIRSYSKLFCDCIWFTTSLFLRQSEKEP
SSSHKSRFFIVLLTIAATYVIGDTYSANLTSLLAKPGREKAINNLIQLEE
AMKFNGLKLFVEKYSSTQSLLENGTSIYGRLWDIMNTAQTSYLINSVEEG
VMMVKDSSDVAVIAGRETLFFDTQRFGPTNFHLSEKIITAYSAIAFQLGC
PYIENVNKILMAIFEAGILTKMTESEYEKLGKEQMKEIENIDKNLDTSTK
KEIRNTIKVANSKDKLKAINIKMLQGAFYLLFIGYFVAGVSLLEEIFYRK
HQKKCLRLRRKFTDNIQTLTTWLRRRLQRFLNEAILSTFDYTE
>HeleIR41a1(a)
MERYNYRKYLFIPMDNANENYLDVFESVALEFVADLDVIVKTNISEEDDI
FHVITHRYGREINHKTTMILDKWFFNNQSFLFGNNLYPNKLKNQYGRTLK
LGTIDYVPYSVIDSLDGLDISIVMELAKKLNMTISLVLDTEVQWGEIYPN
FTGNGILGNLVMDRTDIGFASMFIWEEEYHYLDISKQTIKSSISCLVPAP
KLLGGWSTPFVAFNKPIWMVFGLTVVVSVIALYFVLAWKHIMQFGLTKTK
FSKYLFNSILLTEAMYLNQSVTYIPNHFSTRLVFSMILMMSLVLGSIYDS
GLASIMTIPRYHSVIR
>HeleIR41a2(a)
MSLALSGRMFLMQSFQKIPNLDQSRLTFGLALILSLLLNTIYSSGLSSTM
TLPRFYGTIHDVNDLVAAQIHWGATSEAWISSIESDSRDVYKKAVDAFII
TNEENLKDQNDKEFAYAIERLQAGNLAIGNYISLQGAQNRRLLDEDVYWE
YCVLMLRKNSIHLHSLNDLIFAATESGLVNYWEYQAVVKYMDWNVQKAVK
STFKSTSTDSDSVVKLTLDHVMGAFTIWGVGIMISICVLIFELFKERRVN
LQHSA
>HeleIR68a(a)
MTIPWFELSTVPFPLPIQTIFVPKRLDIWRKSKFRTGVNLFQDKTRDLRN
QTMRVVVMKHTPATTKLTISEDFTVRAVINAGSASFSGLEVEILSAVSKA
MNFKCRLYEAPNSEDLWGRKQIGGKYTGLIGEMVKNGADIALGDLYYTSY
ILELMDLTVPYNTECLTFLTPESLSDNSWKTLILPFKLVMWLMVLLCLFV
CGIIFHYLALFHSIINQTRKIKAKGMEFYTKKKCIVSIPIININQLDTNS
KYILMKKQFAENQNLQPNGLYVFSDFFNSILYTYSMLLMIALPKIPSGWS
LRMLTGWYWLYCTLVAVSYRASMTAILANPAPRVTINTLQELVDSELYLG
GWGEVNRELFKTSLDPITQIIGAKFEYINDTKEAIVKVSEGNFALYENTY
FLKEISVLRQSQQTLSVEDQTNKNDRNLHIMKDCVITMPISIGLQKNSPI
KPRVDMYIRRVLEAGLIRKWLDDVMQPTLNAEIPGISGTTKAIMSMRKFI
GAIVALGIGYFVSLVALCVELVYFHFAVRKHPNFNKYSRKICK

>HeleIR75a1(a)
MAFSAVCQQGSNSIPHTIAGRIITILLFTSLMFLYTSYSANIVALLQSSS
TSIQTLSDLLHSRLQVGVDDTVFNRFYFPNATEPIRRAIYLQKVIPPGQP
ARYFPIEEGVKMMRQGLFAFHMETGAGYKLVGETFLESEKCDLEEIQFLQ
VPDPWLAIQKNSSYKEMLKIGFRKIQESGIQPREVALLYTKKPNCVFRAS
TFIGVSIVDCYPAAEILIIGYLIAVCIFLIEILHYYRMKIYLGIIARGSK
VPYTH
>HeleIR75a2(a)
MDLMELVKDFLLIQNSPTKISLHLCWKTYEQILLAKYLYNYPLSFHYSNK
SEPCFDKQLAEELFLVDLRCPENFNVLDVANRRMLFQQPYRWLVITDNYI
IPRSLNILIDSRFYVINKSSSGIYTLHSPYKISKHAKSFITNDVATWSLE
FGFMYFNQLHAVRNRTNLMGLTVNISYVITDIDSLNHLWDYRDKHIDGIS
KLNYILIHHVTDFMNASRNFIMRNTWGYKNVTTNSYDGLIGDLQSDRAEF
AGTASFFTQDRISVVDFIASTTPTRAKFIFKAPPLSYVTNVFTLPFDRMV
WYASFLTIGIISVVVYVIVKWEWGNSEFQHVLAKKQNAMRPKYIDVLLME
LGAICQQGSEAEPKSGAGQIATMFIFSALMFMYTSYSANIVALLQSTSES
IKTLDDLLNSRIKLGVEDKPYSYYYFKMQTEKTRRAIYTQKVAPLGQKPN
FMNVEDGIRKVKDEFFAFHVECASGYKIVADIFQESEKCGLREIEYWQII
DPWVAVKKNSSYKELAKIAYRKLQENGVQNREFARLYAKKPVCQSRGSNF
ISVGLVDCYYAFLVFAVGCFIAVGILLIEIIYKRRISYISSIRHS
>HeleIR75a3(a)
MLEIDITLTSNSSEIINLIEDFILILHKPVKISFDVCWPQNDKLLLLQHL
NQHLISFQHFDPRKYGEDTVPTVTELFLLDLRCEGYNESLHDVDQKNLFK
QPYKWVVIVNNFDDIPHNLNILVDSQFYLINETLSNEYVIHSIYKITNNT
NEFVVHDIGLWTQAAGFKYFNYLTIGKNRTNLMKTHLNFSIVITNPDSLN
HLWDYRYRHIDSMTKLNYFFCHHIVDYLNISHTFIVRKTWGYRNETNNRY
DGMIGDLQSGLAEIGGSPAVLMAERLAVADYASPTTQTNAKFIFRAPSLS
HVTNILTSPFDATVWYCYFILIGIIGIVLYLIVVCEWKNPARMNQPTGLQ
PNFIDVLLMQLGAICQQGSEAEPRSNSGRIATIFIFINVLFMYTFYSANI
VALLQSTSETIQTVEDLLYSRIKLGAEDNAYTKYYFKIQTEPIRKAIYTQ
KVQPEGHASNFFDAKIGIQKVKDDFFAFHVERAVGYKIIADIFEENEKCR
LREISYTSLLSDPWIPIRKNSSYKEVTKIAFRKLHENGLQSREFRKIYYN
KKPICESKGSSVTSVGLMECYYALSIFAVGWLLSVCLLLLEILIKKKCSQ
NSLYRAAST
>HeleIR75a4(a)
MKFLFFITTLVLHCTESFQDNSIRLLHDFVNSKNIMLTLNSCWNKDKLLE
FLQIASYGKDNAKFVSVHKQFSANMYFPNNLVIMDINNCEKAWSLLLKLN
QTNSFISPTKYFIMVDNVTSIVPVLQNFTIYPGSDVVIAEKYGYDFKLHG
VYRINGISQLIWEKYGYWSANHGLLEYLKRYPPSQRRHNLRKENLKVMIR
MTDNDTWNHLDDFRHASVDSSTKLSMGSTICLLDYCNANVTYVSSEYFGY
ADETGKFNGIVAALINEEIDTSGTAMYPTRERVDVISFMTMQSSYKIKFI
LKKPPLSYVRNIFAAAFAANVWIVMVAALTAFGVVCYFIYNLEAKHERMG
FQDKLSSTDVILISLEVLSQQGTSTDPQRISGRILAWLYFLGFFFIFVVY
SGNIVAMLQAQVKLMSRKELLTTRMEIGAEDNNYMRIYVSRDTDEVGRQI
YQKIGANGYYKFDEGMKKVQNGYFAYQVELGEAYDYILANFTNDDMCSLQ
EIDGYFHRHRGHSVTRKKSPYKEIFKTGFSKIDEYGLKLRNYNRWCVKPV
CHAKRANYESVGLTESRMAFLLLIYGTLVALLFLLLEKIAGHAKKKYGMV

RIKRMF
>HeleIR75a5(a)
MAFFMLGVLLLQILGSLQNHSIIELINDFADSKGLTISLHTCWNKKEQFE
FMKGAIHANGRLKYTSISKNISDAQLLHNNLIVLTLENCESAWTTLLQIN
QSNLLISPAKYFLLVDNIDGIEPILQKSTAYPASDIFIVTKNGTTYDIQQ
AYRINAFSDLIWEKYGRWSPEFGLAEYLRKYPASQRRRNFRRETLKVMIR
LLDNDTWNHLDDLRDPAIDTFTKLSYGATSCLLEYCNANASYSGSEYFGY
YDNNTGKYSGIVGALHNKEVDVSGSSMFPTHERLERIEYLGRQSPYYIRF
LLKKPPLSYIRDIFTLALERNVWISIGAILTIISTALFVILNLEAKEQKI
GHQQKFSTSDVILISLEAVCQQGTTTEPRRVSGRILALITFFAFMFVYVA
YSANIVAMLQSQVELKSTKELLESRIEVGAEDNNYMRIYISQDKDKVRQQ
MYQKKIGKDGFYSFNEGMEKLRKGFFAYQVELGSAYNYIIEKFTGYEMCC
LQEIEGYFYHLRGYVATQKKSPYKEILKTGTLKVDEYGLRKRSYIRYFIK
PSCQVKGTNFESIGLIETRMPFVVLISGILLALLFLFLEKSTQYLKNTD
>HeleIR76b(a)
MGLIDLVVLTLCANTSCPNDTVYDISSMQQQLLRHQAHLLRNYTLKIATV
HNPPLSSIVETNGTWQAGGIAFDYIEILRKKFGFKYEIIKPPEDSLNAED
NGIIGMLSRNEVDIGAAFLPAFPFLSNSIRFSVSLSKEEWVVLMKRPPVS
ATGSGLLAPFTFQVWLLILVSLFAVGPIIYLLILLQHYLCKDETSRIYPL
PSCVWFVYGALLKQGSTLSPQTDSSRILFATWWIFITILTAFYTANLTAF
LTLSRFTLPITTITDIGTKKYTWVSHKGNAIEAALNNDINFKLSLQGSHG
KFLDEEATSILDNWVKGHNYMYIGEKPIVDHLMYRDYLSKIDPDVAEADR
CTFVVTTWIVKDNLRSFGYSPNFPFVELFDNILEHLVESGIVMYSLREGL
PDTQICPLDLGSKERQLQNTDLLMTYYIVVGGFIVSTIAFLTEFLCSKSI
KTSQPDDLFTKTQKLRAQKLDDQRLVPPPPPYHALFKPPFPHSENAQTKT
VNGRDYWVVKSIDGSTMLIPIRAPSAFLFQYDN
>HeleIR93a(a)
MDLVTYVMQAAQSLHLTDINNQWLYVISDYKMDDESTAEIIYNLKEGDNV
AFLYNTSKSGVSCKEGMDCYIDEMLDAFAKSLDTAIVEEFELASQVSDEE
WDAIRPTKFERRNYMLNNMKDYLLKNGVCGNCTRWQIKACDTWGKKFRSS
SNSDFSLIEVGYWKPSDGPSMTDVLFPHVIHGFRGRSFPLISLHNPPWQI
IESNSSGGLSCKGLVFDIINELAKALNFTYTVVVINDGKNELKNSSYYGK
EISYRVTSAIPDRVVKMIQNKEAFLAAFAYTITEESKLLVNFTIPISTQT
YTLLSARPRELSRALLFMSPFSNATWLCLCAAVIAMGPVLYYIYKYSPVN
EYYGFRVKGGLSSVYNCIWYIYGALLQQGGQYLPYADSARLLIGSWWLVV
LVISTTYCGNLVAFLTFPTYNKPITTIAELLNQKGSITWSIAPATLFEYE
IQVSNEPKYQALYHGSIKTVTNMEEMIRNIESGTHVHIDWKLRLQYMIKK
QFLLQDTCSLSLGIEDFFDERLGLIVSQDNPYLKRINREIKRLHQVGLIE
KWLKDYMPKRDKCFKTRSSNGANNHTVNLNDMQGCFFVLFFGSVVSLLLI
AVEKLYCRHQKRRQKKIVHPFVS
>DmelIR8a
MELPLLVLLLALRFAGSEVLKITFWIEPVQRAEFDTDIAMVLKELDALRLDVKVDDTTLT
LTRSEDGLDMQRFCEILSTVGASAVIDLTYSHWEEGYNLVRSLGIGYVRLERIMRPFLDM
FGDFMRQKRANNVAMVFMNARDAVEAMQQMLVGYPFRTLIMDASQTDPGQHFLERIRSL
R
PAPTYIALFARAAAMNGIFEKVQKADLFQRPLEWHFVFLDTRDRVFKYRRQAELCTRFTL
NPRAICRSMPMPDLYCGSGFTMQRAMLLNVLRSLINAAQVSPGYPLAIYQDCNATASSSE

VSDPLEKDDYNWLDMVHWSNFLAYAPPLPHIQDQFQSPVPGLTFAVNISAGYYSSEHEAK
TDLAAWSSVGEMRLLNETISPARRFFRIGTAESIPWSYLRREEGTGELIRDRSGLPIWEG
YCIDFIIRLSQKLNFEFEIVAPEVGHMGELNELGEWDGVVGDLVRGETDFAIAALKMYSE
REEVIDFLPPYYEQTGISIAIRKPVRRTSLFKFMTVLRLEVWLSIVAALVGTAIMIWFMD
KYSPYSSRNNRQAYPYACREFTLRESFWFALTSFTPQGGGEAPKAISGRMLVAAYWLFVV
LMLATFTANLAAFLTVERMQTPVQSLEQLARQSRINYTVVKDSDTHQYFVNMKFAEDTLY
RMWKELALNASKDFKKFRIWDYPIKEQYGHILLAINSSQPVADAKEGFANVDAHENADYA
FIHDSAEIKYEITRNCNLTEVGEVFAEQPYAVAVQQGSHLGDELSYAILELQKDRFFEEL
KAKYWNQSNLPNCPLSEDQEGITLESLGGVFIATLFGLVLAMMTLGMEVLYYKKKQNALE
ITQVRPVNDSSGSGGNSSTAPPTATSTTKQAWHIPVLEAEEKPAKVSPPPSFETATFRGK
KLPARITLGDGKFKPRHGLYARRNLGASDSHSGYME
>DmelIR25a
MILMNPKTSKILWLLGFLSLLSSFSLEIAAQTTQNINVLFINEVDNEPAAKAVEVVLTYL
KKNIRYGLSVQLDSIEANKSDAKVLLEAICNKYATSIEKKQTPHLILDTTKSGIASETVK
SFTQALGLPTISASYGQQGDLRQWRDLDEAKQKYLLQVMPPADIIPEAIRSIVIHMNITN
AAILYDDSFVMDHKYKSLLQNIQTRHVITAIAKDGKREREEQIEKLRNLDINNFFILGTL
QSIRMVLESVKPAYFERNFAWHAITQNEGEISSQRDNATIMFMKPMAYTQYRDRLGLLRT
TYNLNEEPQLSSAFYFDLALRSFLTIKEMLQSGAWPKDMEYLNCDDFQGGNTPQRNLDLR
DYFTKITEPTSYGTFDLVTQSTQPFNGHSFMKFEMDINVLQIRGGSSVNSKSIGKWISGL
NSELIVKDEEQMKNLTADTVYRIFTVVQAPFIMRDETAPKGYKGYCIDLINEIAAIVHFD
YTIQEVEDGKFGNMDENGQWNGIVKKLMDKQADIGLGSMSVMAEREIVIDFTVPYYDLV
G
ITIMMQRPSSPSSLFKFLTVLETNVWLCILAAYFFTSFLMWIFDRWSPYSYQNNREKYKD
DEEKREFNLKECLWFCMTSLTPQGGGEAPKNLSGRLVAATWWLFGFIIIASYTANLAAFL
TVSRLDTPVESLDDLAKQYKILYAPLNGSSAMTYFERMSNIEQMFYEIWKDLSLNDSLTA
VERSKLAVWDYPVSDKYTKMWQAMQEAKLPATLDEAVARVRNSTAATGFAFLGDATDIRY
LQLTNCDLQVVGEEFSRKPYAIAVQQGSHLKDQFNNAILTLLNKRQLEKLKEKWWKNDEA
LAKCDKPEDQSDGISIQNIGGVFIVIFVGIGMACITLVFEYWWYRYRKNPRIIDVAEANA
ERSNAADHPGKLVDGVILGHSGEKFEKSKAALRPRFNQYPATFKPRF
>DmelIR21a
MSYYWVALVLFTAQAFSIEGDRSASYQEKCISRRLINHYQLNKEIFGVGMCDGNNENEFR
QKRRIVPTFQGNPRPRGELLASKFHVNSYNFEQTNSLVGLVNKIAQEYLNKCPPVIYYDS
FVEKSDGLILENLFKTIPITFYHGEINADYEAKNKRFTSHIDCNCKSYILFLSDPLMTRK
ILGPQTESRVVLVSRSTQWRLRDFLSSELSSNIVNLLVIGESLMADPMRERPYVLYTHKL
YADGLGSNTPVVLTSWIKGALSRPHINLFPSKFQFGFAGHRFQISAANQPPFIFRIRTLD
SSGMGQLRWDGVEFRLLTMISKRLNFSIDITETPTRSNTRGVVDTIQEQIIERTVDIGMS
GIYITQERLMDSAMSVGHSPDCAAFITLASKALPKYRAIMGPFQWPVWVALICVYLGGIF
PIVFTDRLTLSHLMGNWGEVENMFWYVFGMFTNAFSFTGKYSWSNTRKNSTRLLIGAYWL
FTIIITSCYTGSIIAFVTLPAFPDTVDSVLDLLGLFFRVGTLNNGGWETWFQNSTHIPTS
RLYKKMEFVGSVDEGIGNVTQSFFWNYAFLGSKAQLEYLVQSNFSDENISRRSALHLSEE
CFALFQIGFLFPRESVYKIKIDSMILLAQQSGLIAKINNEVSWVMQRSSSGRLLQASSSN
SLREIIQEERQLTTADTEGMFLLMALGYFLGATALVSEIVGGITNKCRQIIKRSRKSAAS
SWSSASSGSMLRTNAEQLSHDKRKANRREAAEVAQKMSFGMRELNLTRATLREIYGSYGA
PETDHGQLDIVHTEFPNSSAKLNNIEDEESREALESLQRLDEFMDQMDNDGNPSSHTFRI
DN
>DmelIR31a
MNLLISMFILILAAGEGEIIPSMEESVVTNFVKSLVKTKQAIVFSCLFKDFKEISLALMR

INQFVSVVNLNQSYSLTSILTRENYARTSVMVNARCSGSSELLFEASENRYFNKTYQWFL
WGVDLEVQSLFPLNLNYVGPNAQITYVNETADGYAYWDIHSKGRHLKSNLEINLIATLIN
DTLNIARDIFHLQSIDFRGQFNGLTLRGASVIDKEDIISNEQIESILSRPTKDAGVAAFI
KYHYELLGLLRERFNFTVNFRNSRGWAGRLGNTTFRLGLLGIVMRNEADIAASGAFNRIN
RFAEFDTIHQSWKFETAFLYRYTSDLDTHGKSGNFLSPFSDRVWLFCLLTLGAFSIIWVL
FEIIDYKILRIRVNSQKLEHLNQKSSVICIKTTCIERILQTFGACCQQGLDPNPVDRSVR
FLVMTLFLFSLVMYNYYTSSVVGGLLSSSDQGPSTVDEITASPLKISFEDIGYYKVLFRE
SQNRSITRLIEKKLSSSRSLNELPIFSHIEDAVPYLKAGGFAFHCEVVDAYPVISEYFDA
NEICDLREVSGLMEVEILNWILHKNSQYTEIFKTAMCNAQEKGFVERILRRRQIKKPACQ
SLYTVYPVSLSGVLPGFVILICKSINKFS
>DmelIR40a
MHKFLALGLLPYLLGLLNSTRLTFIGNDESDTAIALTQIVRGLQQSSLAILALPSLALSD
GVCQKERNVYLDDFLQRLHRSNYKSVVFSQTELFFQHIEENLQGANECISLILDEPNQLL
NSLHDRHLGHRLSLFIFYWGARWPPSSRVIRFREPLRVVVVTRPRKKAFRIYYNQARPCS
DSQLQLVNWYDGDNLGLQRIPLLPTALSVYANFKGRTFRVPVFHSPPWFWVTYCNNSFEE
DEEFNSLDSIEKRKVRVTGGRDHRLLMLLSKHMNFRFKYIEAPGRTQGSMRSEDGKDSND
SFTGGIGLLQSGQQADFFLGDVGLSWERRKAIEFSFFTLADSGAFATHAPRRLNEALAIM
RPFKQDIWPHLILTIIFSGPIFYGIIALPYIWRRRWANSDVEHLGELYIHMTYLKEITPR
LLKLKPRTVLSAHQMPHQLFQKCIWFTLRLFLKQSCNELHNGYRAKFLTIVYWIAATYVL
ADVYSAQLTSQFARPAREPPINTLQRLQAAMIHDGYRLYVEKESSSLEMLENGTELFRQL
YALMRQQVINDPQGFFIDSVEAGIKLIAEGGEDKAVLGGRETLFFNVQQYGSNNFQLSQK
LYTRYSAVAVQIGCPFLGSLNNVLMQLFESGILDKMTAAEYAKQYQEVEATRIYKGSVQA
KNSEAYSRTESYDSTVISPLNLRMLQGAFIALGVGSLAAAALNNTINVRSLNSRDKFICG
GPVKIWYYLVLLLWYYFNRGLVGIYQLWHKTSIRNTGKGMPFLGE
>DmelIR64a
MHWWLLVFLPLSCQGLPEHELLELELDYGLAEPQRTSLLQSSLILQFSQDYKHIPRITYF
TCQKPHLQTPNQIPNAAEHRDAFAAKNFQLIKSLYESELFVRIVLLDVLAQSPTSGRPNR
PGNGPTGGFSQTPSQAQSNSEWLEGVLRMEALRQIAVVDLACGAVSRRFLELASAKMLYS
EKFHWLLIEDFAWHGRTQTAEGSGKRDDGEMEEEEPPGQQIQATDDEDLPSIESFLGGMN
LYMNTELTLAKRMSEAAHYTLFDVWNPGLNYGGHVNLTEIGSFTPTEGIQLHTWFRTTST
VRRRMDMQHARVRCMVVVTNKNMTGTLMYYLTHTMSGHIDTMNRFNFNLLMAVRDMF
NWT
FVLSRTTSWGYVKNGRFDGMIGALIRNETDIGGAPIFYWLERHKWIDVAGRSWSSRPCFI
FRHPRSTQKDRIVFLQPFTNDVWILIVGCGVLTVFILWFLTTIEWKLVPHDGSALIKPKG
GAPPRHHYQQQQQEQVEAPVRPITAVSVVVSKEKVEEKQEEYEDSTPIDAGTLWQRCYQ
KLNKYIKDRKAKQKKAPERVGLFLESVLFFVGIICQQGLGFSTSFVSGRCIVITSLLFSF
CIYQFYSASIVGTLLMEKPKTIKTLSDLVHSSLKVGMEDILYNRDYFLHTKDPVSMELYA
KKITSVPTTKENEADEDEPVDPNPVSTDPAKSYRDIVHSHETGAHAKDNAASNWLDPETG
LLRVKHERFAFHVDVAAAYKIIAETFSEQDICDLTEVSMFPPQKTVSIMQKNSPMRKVIS
YGLRRVTETGILTYHFNVWHSRKPPCVKKIETSDLHVDMDTVSSALLILLFSYAITLMIL
GTEILYSKWHNRIQLKWVGAT
>DmelIR75a
MQLVQLANFVLDNLVQSRIGFIVLFHCWQSDESLKFAQQFMKPIHPILVYHQFVQMRGVL
NWSHLELSYMGHTQPTLAIYVDIKCDQTQDLLEEASREQIYNQHYHWLLVGNQSKLEFYD
LFGLFNISIDADVSYVKEQIQDNNDSVAYAVHDVYNNGKIIGGQLNVTGSHEMSCDPFVC
RRTRHLSSLQKRSKYGNREQLTDVVLRVATVVTQRPLTLSDDELIRFLSQENDTHIDSLA
RFGFHLTLILRDLLHCKMKFIFSDSWSKSDVVGGSVGAVVDQTADLTATPSLATEGRLKY

LSAIIETGFFRSVCIFRTPHNAGLRGDVFLQPFSPLVWYLFGGVLSLIGVLLWITFYMEC
KRMQKRWRLDYLPSLLSTFLISFGAACIQSSSLIPRSAGGRLIYFALFLISFIMYNYYTS
VVVSSLLSSPVKSKIKTMRQLAESSLTVGLEPLPFTKSYLNYSRLPEIHLFIKRKIESQT
QNPELWLPAEQGVLRVRDNPGYVYVFETSSGYAYVERYFTAQEICDLNEVLFRPEQLFYT
HLHRNSTYKELFRLRFLRILETGVYRKQRSYWVHMKLHCVAQNFVITVGMEYVAPLLLML
ICADILVVVILLVELAWKRFFTRHLTFHP
>DmelIR76a
MENLLVESYYFSTVLSFFAQQFFADSHATCIFWHPAFDFRLETVHPMPLIIMDWHRWANR
SDQDVYDYKIKEDEFEGKGIPYNDWTLRLTVAIERSHCETFIAFQEQIPEFARYFYHASI
YSIWRSLRNRFMFVYTKEFEDKKDSYLSGYIFQDQPNILVITSQYLNSSTFEIKTNRFVG
PRNFNKNPEPVEFYILQRFDAKGTKATWETQSAMSSKMRNLKGREVVIGIFDYKPFMLLD
YEKPPLYYDRFMNTTDVTIDGTDIQLMLIFCELYNCTIQVDTSEPYDWGDIYLNASGYGL
VGMILDRRNDYGVGGMYLWYEAYEYMDMTHFLGRSGVTCLVPAPNRLISWTLLLRPFQF
V
LWMCVMLCLLLESLALGITRRWEHSSVAAGNSWISSLRFGCISTLKLFVNQSTNYVTSSY
ALRTVLVASYMIDIILTTVYSGGLAAILTLPTLEEAADSRQRLFDHKLIWTGTSQAWITT
IDERSADPVLLGLMEHYRVYDANLISAFSHTEQMGFVVERLQFGHLGNTELIENDALKRL
KLMVDDIYFAFTVAFVPRLWPHLNAYNDFILAWHSSGFDKFWEWKIAAEYMNAHRQNRIV
ASEKTNLDIGPVKLGIDNFIGLILLWCFGMICSLLTFLGELWRGQG
>DmelIR84a
MIKLQVKVISWPLIILTAFLRVLQIESINTNFLELAAFEDFLRSEHLSHVLVVRGDDADG
DWKIECHQKLLANYRVQFYRPEMSANFEDLMFYGSPRTAVLVLNSEHVLVRRQVFGVASE
AGYFNNSLAWFILGSGRESLPVEQLIDQLLSGYRMGIDADITVALRGPDNASMLFYDVYR
ISRQANTPLIIEKKGLWTHSGGYQKFGNFKNTWVIRRRNFLNVTLIGSTVLTEKPPGFGD
MEYLADDKQLQQLDPMQRKTYQLFQLVERMFNLSLAISLTDKWGELLDNGSWSGVMGQ
VT
SREADFAVCPIRFVLDRQPYVQYSAVLHTQNIHFLFRHPRRSHIKNIFFEPLSNQVWWCV
LALVTGSTILLLFHVRLERMLSNMENRFSFVWFTMLETYLQQGPANEIFRLFSTRLLISL
SCIFSFMLMQFYGAFIVGSLLSESARSIVNLQALYDSNLAIGMENISYNFPIFTNTSNQL
VRDVYVKKICKSGEHNIMSLQQGAERIIQGRFAFHTAIDRMYRLLLELQMDEAEFCDLQE
VMFNLPYDSGSVMPKGSPWREHLAHALLHFRATGLLQYNDKKWMVRRPDCSLFKTSQAE
V
DLEHFAPALFALALAMVASALVFLLELFLHWLPDFRRRLGTMST
>DmelIR92a
MLLQPLVMHLSQLLRIIVGQYFAEFPSILIVYNNSASTTPLQLEYLSALELVLRELSKPI
RLQWINVAFLKDLNDLEDQVMGALNSSVTEGFITILSQTHHFIHARYYATRNANVRLKDK
RYLFLCEDESPAELLCMDILQFYPHHLMVRPGTETAPTGPTGPHPDPRRGGGASVSTKNK
DDGEGGAGNKTTSPYRDINFELWTQKFVGAVGNLDALLLDAFLPNETFANRVELYPNKLL
NLQRRSLLVGSITYVPYTITNYVPAGQGDVDPIHPQWPNRSLTFDGAEANVMKTFCQVHN
CHLRVEAYGADNWGGIYDNESSDGMLGDIYEQRVEMAIGCIYNWYDGITETSHTIARSSV
TILGPAPAPLPSWRTNIMPFNNRAWLVLISTLVICGTFLYFMKYVSYRLRYSGTQVKFHH
SRKLEKSMLDIFALFIQQPSAPLSFDRFAPRFFLATILCATITLENIYSGQLKSMLTFPF
YSAPVDTIEKWAQSGWKWSAPSIIWVHTVQSSDLETEQILARNFEVHDYSYLSNVSFMPN
YGFGIERLSSGSLSVGDYVSTEALENRIVLHDDLYFDYTRAVSIRGWILMPELNKHIRTC
QETGLYFHWELEFIDKYMDKKKQEVLMDLANGHKVKGAPQALDVRNIAGALFVLAFGVA
F
AGCALVAELLIHRMDLSK

>DmelIR93a
MNPGEMRPSACLLLLAGLQLSILVPTEANDFSSFLSANASLAVVVDHEYMTVHGENILAH
FEKILSDVIRENLRNGGINVKYFSWNAVRLKKDFLAAITVTDCENTWNFYKNTQETSILL
IAITDSDCPRLPLNRALMTVECRINAVVFVDQTILEENALLVKSIVHESITNHITPISLI
LYEINDSLRGQQKRVALRQALSQFAPKKHEEMRQQFLVISAFHEDIIEIAETLNMFHVGN
QWMIFVLDMVARDFDAGTVTINLDEGANIAFALNETDPNCQDSLNCTISEISLALVNAIS
KITVEEESIYGEISDEEWEAIRFTKQEKQAEILEYMKEFLKTNAKCSSCARWRVETAITW
GKSQENRKFRSTPQRDAKNRNFEFINIGYWTPVLGFVCQELAFPHIEHHFRNITMDILTV
HNPPWQILTKNSNGVIVEHKGIVMEIVKELSRALNFSYYLHEASAWKEEDSLSTSAGGNE
SDELVGSMTFRIPYRVVEMVQGNQFFIAAVAATVEDPDQKPFNYTQPISVQKYSFITRKP
DEVSRIYLFTAPFTVETWFCLMGIILLTAPTLYAINRLAPLKEMRIVGLSTVKSCFWYIF
GALLQQGGMYLPTADSGRLVVGFWWIVVIVLVTTYCGNLVAFLTFPKFQPGVDYLNQLED
HKDIVQYGLRNGTFFERYVQSTTREDFKHYLERAKIYGSAQEEDIEAVKRGERINIDWRI
NLQLIVQRHFEREKECHFALGRESFVDEQIAMIVPAQSAYLHLVNRHIKSMFRMGFIERW
HQMNLPSAGKCNGKSAQRQVTNHKVNMDDMQGCFLVLLLGFTLALLIVCGEFWYRRFRA
S
RKRRQFTN
>DmelIR7a
MFHHLWLLMGLRSLAMGALHPPQPEAMTPLVAAALEILAEQVSPSQSTLAVMDLTQDAEH
RDERQEQLMTIILRSVGSEMALRTFQKPPAEVPASFVVFLVNSAQAFNTLGFHFTDIHST
REFNFLILLTHRMSSRAERLQVLRDISRTCVRFHTSNVILLTEKRDGVVLVYAYRLLNMD
CDLSVNLELIDIYKNGLFRHGHEARSFNRVLSLSGCPLQVSWYPLPPFVSFIGNSSDPEE
RAQIWRLTGIDGELIKLLASIFDFRILLEEPCNKCLSPDIKDDCSGCFDQVIISNSSILI
GAMSGSHQHRSHFSFTSSYHQSSLVFIMHMSSQFGAVAQLAVPFTVIVWLALVVSSLLLV
LVLWMRNRLVCGRSDLASHALQVLTTLMGNPLEARSLPRSSRLRILYAGWLLLVLVLRVV
YQGKLFDSFRLPYHKPLPTEISELIRSNYTLINQEYLDYYPRELTVLTRNGSKDRFDYIQ
GLGKEGKFTTTSLIATMEYYNMMHWSTSRLTHIKEHIFLYQMVIYLRRHSLLKFAFDRKI
KQLLSAGIIGYFVREFDACQYRKPFEEDYEVTPIPLDSFCGLYYISLIWLSAAVVAFILE
LLSQRIVWLRRIFE
>DmelIR11a
MRFAILWLFSGCLLPGIQVGIWVVVRAQPTGRDVLLSRLGNQQNELNTRRLANASSYLTR
NYIANRINTLVVREICVECPYELSERQRQLVDQILASLAPELSVLLHKGTAEETTWEYTL
FVVNDHTAFTGQVFIFPDELLEREFFCIVVVSEIQSRQFVRQTVGSIVKSNLQMHFVNVV
VVAQLEDGTVGTYSYKLFKANCTPGITVRQINHFDRITGKPQQSMPDLYPVRNGHLGDCP
FNVGAAHMPPHLIYKRHKDPPPASNVSIPAEDLAGIDWDLLQLLAKALKFRIQLYMPQEP
SQIFGEGNVSGCFRQLADGTVSIAIGGLSGSDKRRSLFSKSTVYHQSNFVMVVRRDRYLG
RLGPLILPFRGKLWGVIIVILLLAVLSTCWLRSRLGLSHPIEDLLTVIVGNPIPDHRLPG
KGFLRYLLASWMLLTLVLRCAYQARLFDVLRLSRHRPLPKDLSGLIKDNYTMVANGYHDF
YPLELTCRQPLDFSARFERVQRAAPDERLTTIALISNLAYWNHKHPNISRLTFVRQPIYM
YHLVIYFPRRFFLRPAIDRKIKQLLSAGVMAHIERRYMQYENKRKVASNDPVLLRRITKS
IMNGAYRIHGLVIVLATGMFILELLAGRSNGRLRRWMEWVHQ
>DmelIR20a
MLASLNRSTGLSAELLDLYGLVVHFLLSGEHTTLVYFNPAGLDCSWGVLWQRNLTAHPQI
VWQRNYSYPDLYYQFNAKLLVLACLPMDSRAAIQLEILANSLSHLRTVVRLLIEVAGPDQ
VTLARQYLSFCLRRSMLHVELYFRDYHHSLILYSFRAFPSFELVMRWISVGQGVKLFLHK
LDDLRGHRLRVIPDLSPPNTFFYRDARGDNQVTGYLWDFLATFAGRLNAGLEVVRPSWRA
GSASDSSYMLEYSAKGLIDVGLTTTLITKWNLWAIHQYTYPLLVSSWCTMLPVEKPLATP

DLFGRIVCPTLAMTLLLIILVTWLVFRQLRCLTRLKNSRPARIVPHLLTLLLLTTCSAQL
LSLLIFPPYHVRIASFEDLLRGDQKILGMRNEFYNFDGAFRARYAGVFYLIDDPNELYDL
RNHFNTTWAYTMPYIKWLVIKTQQRHFSKPLFRWSKDLCFFDFMPTSVIVAPDSIYWESI
KDFTFRIHQAGLMKHWIRKSFYDMIKAGKMSIKDYSDLETLKPLNIGDLEIVWRVCGAAI
AVASAIFIMELLYFYINVFFNSL
>DmelIR41a
MFIDLSWSLVLSAIVGKYLNESTICIFWNDKFEFQLLHKSDYISFVGINIKSFDDNGGHY
IIDTGLKKKELQNKHLFLDELVIKIIISIEVTHCETFVVFDKDIDRFVNAFNKASVYSIW
RSLHNKFVFAHIANESPESRNHFFEDQPNILFVVRDHSSASSFDIKTNKFVGRKAENPSQ
MILVDRYLASEQRFQFGKSLFADKLNNLQGREVIIAGFDYPPYTVIKHNMSTNAQDMGVS
GESDFKNVYIDGTETRIVLNFCEQFNCTIQIDSSAANDWGKVYPNMSGDGALGMLINRKA
DICIGAMYSWYEDYTYLDLSMYLVRSGITCLVPAPLRLTSWYLPLEPFKETLWAAILLCL
CAEATGLVLAYKSEQALYVLPGYREGWWTCTSFGVCTTFKLFISQSGNSKAYSLTVRVLL
FACFLNDLIITSIYGGGLASILTIPSMDEAADTVTRLRFHRLQWAANSEAWVSAIRASDE
ALVKDILYNFHIYSDDELLRLAQDQHMRIGFTVERLPFGHFAIGNYLGPQAIDQLVIMKD
DIYFQYTVAFVPRLWPLLDKLNTLIYSWHSSGFDKYWEYRVVADNLNLKIQQQVQETMTG
TKDIGPVPLGMSNFAGFIIVWILGSAIATLTFLLELSLTYILKQSNLK
>DmelIR47a
MRQIKLLVWLLVVGVVSSTEQLQFLKNFLEAVHKERSISTILLIQRKVHKNDFLHGLYPI
FWPIICLDETKRVELVNNFNKDFLALVYMESEADTLLLSALAADLNHIRDARIMIWLQMS
PSENFLDRIVFQASKQKFLNLVVIENTLKTRRFYPFPQPKVQVIDKPFEEKEIYPALWRN
FMGKNAIAVPDLVPPRSFNSFDPKTGHRRESGSIYNVFKAFTQRYNITMLLKWPLIRNTT
QEEIIGKSVRGEIDLPITGQLISFRHPNGSRSQPLLGMTALSIAVPCGPELPMFDRFFLF
YGLATPITITGYYVLLNTIEIILGTLSDRIKRHPRRKKILNLVLNLRVFSCILSLPTPQG
NRLRSVKGQLTMVMSITGLILSCIVAAQTSTILTMKPQYRHIKNFQELSDSNITVVCNHL
NYLTIKQQMDPKFMAKFMQNIWIVNSIEQMKMIFDLNTSYAYQTFSYKKDPFTLLQMHTT
RKAFCRTPGLDLVSGLAYTAVLEKNSIYALALQDYTLKAFSAGLVYYWAEESIRDLISTV
GRTQFEKLPIVIGYQSLKLQDYNVCWKILLIGGALAFCVFIVEVVVGLINRRI
>DmelIR52a
MALGWSVIILGFIGQLSAQILNYTQSRDLELLEGSLFRVLSRLNLEEEYNTLLIYGKECV
FHSLLRKLEISAVTVPSGSTDYDWSFSTAILILSCGYDAENEENSYTLMKLQRTRRLIYL
EDNSEPESVCMRYSLKEQHNIAMVKSDFDQSDTFYSCRLFQTPNYVEGHFFKDQPIYIEN
FQNMRGATIRTVADSLVPRTILYRDEKSGETKMMGYLGHMINTYAQKLNAKLHFIDTSKL
GAKKPSVLDIMNWVNEDIVDIGTALASSLQFKNMDSVWYPYLLTGYCLMVPVPAKMPYN
L
VYSMIVDPLVLSIIFVMLCLFSVLIIYTQHLSWKNLTLANILLNDKSLRGLLGQSFPFPP
NPSKHLKLIIFVLCFASVMITTMYEAYLQSYFTQPPSEPYIRSFRDIGNSSLKMAISRLE
VNVLTSLNNSHFREISEDHLLIFDDLSEYLVLRDSFNTSFIFPVSVDRWNGYEEQQKLFA
EPAFYLATNLCFNQFMLFSPPLRRYLPHRHLFEDHMMRQHEFGLVTFWKSQSFIEMVRLG
LASMEDLSRKRNEEVSLLLDDISWILKLYLGAMFISSFCFILEILRCGERCKRLWRCRW
>DmelIR54a
MWTVITGIVLWAPVLVAGSAVDFIFRAAAEHSLSVIMIRIDYCPYNWAKDIFENQTIPVV
VLSDSETFINIRMFSRPLHVACLPGHELQKDLALLENFTSSLMDFPSQKKIVYISNNFSD
PTRMDYIFETCYHRRIWNIVGLLASDEHRYFYRYHLYPSFRTEYRSLESSTIFDKDFPNM
HGHPLTVMPDQWLPRSVLYVDRRTGKQILAGSVGRFFHVLSWKLNATLQLSKKVTTGRFL
NATALKELSESFSVDVPASLTIMERVEQLASTSYPMEVTHVCLMVPVARRIPIKDIYFIL
SSASNMFLAIVIVSSYGLALNLLRNMTHRDVRLVDFVLNDKALRGILGQSFNLPLSRSFS

TRLIFLMLGIVGLNVSSIFGAGLDTLMAHPPRQFQARSFAGLRRTKIPLVTTEEDFPTWM
KLRVPMLVVNVSEYNHLRNGRNTSNAYFASRLYWNLFSEQQKRFTRELFIYSTDDCLWSL
ALLSFQWPQNSLFTEPVSQLILEVNANGLYDFWVGMHYYDMTAAGLSGLEDPSLQLKERE
HPTSLRIVDFQWMWQAYGTFMVIAILVFLLEVSWHRITSLFVSLVY
>DmelIR56a
MGSRFFIRNLILFGLLASSNMQIPFGELEKKFELDVDFLLGVTELVGHIQGLYSITVYAD
CIDIHPSIQQRIMDKFMVPVNTIGSNLSRPNYHKLDNSRIRIVLFTGLNDTILVNLNKTD
VPYSDNFYMLAYASAIKNKCIELDFIEEVFTLLWKMSIQNAILLIRGEFMMEMWSYLYMG
KIHKIKLTKPNSYLESLRKYNYRFSLEVINDPPAIFWYNSSEQADVTGGGNLSVSGPLGL
IIINFLRHLNVTIDIVPIPGKQTSQYELFQQPDNLRAENGVNMVGSALLKYSPMVTQSRM
CLLVSNRRMIPFSRFLDRLVSPGVHKLTFVSSIGIFVIKYFSHRPRSFVDAIFCTIRFFF
AIPLPSIILNRLPVVDRFIEVFIIIFVQILLSSNISITTSALTTGFWEPPIINVETMRAS
GLHILTEDPTILQAFKENILPSSLADLVILVDEDTYFHHVTTLNNSYVYVVQAHNWQIFR
LYQQQMTNEPFEIASEELCSKWRILGIPLNPKSPLRFMFKDYFYRILESGLREQWVHSGF
KKFCEFNNLKKLPVDSVDSWQPLSIEFYSNVIRAYIIGLVIATLAFVAELLHNGYRRKNV
KKT
>DmelIR60a
MWCNNPGLIIIIFLGQILNLCQGIVNLSNETANTVIFMLPEKDLGPDVWKAGVGCLDSFA
QIFFFRNPKERFTRAYNLMLVHAFHLSSPADQIQEGFSKLINEAVTNPGPPDREELFQMR
VASDYNITNGTEDKGELILADNYVIVVDSVDRLKELMKKKIVEMRSWNPGARFLVLFHNA
TCRNRPLGVASNIFKDLMEMFYVHRVALLYANSTMNYNLLVNDYYSNVNCRILNVQSVGQ
CHDGKLYPNNAVVKASMQDYVSGFSPRNCTFFACSSISAPFVEADCILGLEMRILGFMKN
RLKFDVNQTCSLESRGEMDGPANWTGLLGKVQNNECDFVFGGYYPDNEVADHFWGSDTY
L
QDAHTWYIKMADRRPAWQALVGIFEAYTWIGFILILIISWLFWFTLVMILPEPKYYQQLS
LTAINALAVTISIAVQERPICETTRLFFMALTLYGLNVVATYTSKMIATFQDPGYLHQLD
ELTEVVAAGIPFGGHEESRDWFENDDDMWIFNGYNISPEFIPQSKNLEAVKWGQRCILSN
RMYTMQSPLADVIYAFPNNVFSSPVQMIMKAGFPFLFEMNSIIRLMRDVGIFQKIDADFR
YNNTYLNRINKMRPQFPETAIVLTTEHLKGPFFILVVGSCWAALTFIGELIIHRWRTQLV
STSEQQDRRSDKRRRRRRRRKPEKDNRWQRQVQVAPVVRFTPVKRRKVFQGQTSQK
>DmelIR62a
MYLQFLFALFLSRYQIVATENFDRAFELALFLDRIGRVHRLHAITIVNSLGSVDPSYLDD
LHRGLMCNSSNHFYMLPQMTATDKDSSHVHFSSLQDEETIYLVFARDSKDAVIYLQAERA
RGRRYTRTMFLLRKQESQKDIKYFFELLWKLQFRSALVVVAARNFYQMDPYPTVRVIRMR
RLSSYDPHHVFPPANRKNFRGYRMRLPVQQDVPNTFWYKNRRTKAWELAGLGGILINQLM
MHLNVTMDLFRFEVNGSSLLNMAALTDLIVKGKVELSPHLYDTLQSNTSVDYSYPTQVAP
RCFMIPLDNEISRSLYVFLPFSLTMWLCLLFVLLVVHFVYVRRLIPDGHFWAILGVPGAG
QVRYGNRKPVRRFSTFLILFGIFILGQTYSTKLTSSLTVTLIRRPDNSLEELFLLPYRIL
VLPTDVYAIVDSLGHAEQFSTKFSCTDAENFSQKRISMHPEYIYPISTIRWRFFDMQQRF
LRKKRFYFSKICHGSFPYQYQLRVDSHLKDALHRFLLHVQQAGLHDLWLDTCYRKAHRM
G
YLKDFSTLAELEEKLRLRPLALNLLVPAFSLFLCGMLGSGIAFLVEIRHSFGCRQKPPSI
NRNPGD
>DmelIR68a
MRCLWILIVAFISLAMATSIPIPIANPAPLSGYEMQLKILLQKILWVANVKRCFAVITDD
LHYPIYDRIFFESVGRRVIPFFVMRTNESDDLQRPSRQVELFVKAIKSSDCELNVITILN
GWQVQRFLGYIYDNRSLNMQKKFVLLHDLRLFESDMIHLWSVFIDAIFLKRQLDNKYTIS

TIAFPGILSGVLVMKNIANWELGKGLNGRILFADKTSNLFGTSLPVAISEHVPMVLWANA
TKSFQGVEVEIMNALGKALNFKPVYYKPNQTENMDWTELDGGASVAYGSGNPDGYAQNG
T
HIDSMLVDEVAAHSARFAIGDLHLFQVYLKLVELSAPHNFECLTFLTPESSTDNSWQTFI
LPFSAGMWVGVLLSLFVVGTVFYAISFLNAIINGNVSSEFFRCLRPNRNVPMDPKIYRRI
SFRIAISRYRSSKGDRMPRDLFDGYTNCILLTYSMLLYVALPRMPRNWPLRVLTGWYWIY
CILLVATYRASFTAILANPAARVTIDTLEDLLRSHIPPSTGATENRQFFLEANDEVARKV
GEKMEVFGYSDDLTSRIAKGQCAYYDNEFYLRYLRVADESGSALHIMKECVLYMPVVLAM
EKNSALKPRVDASIQHLAEGGLIAKWLKDAIEHLPAEALAQQEALMNIQKFWSSFVALLI
GYVISMLTLLAERWHFKHIVMKHPMYDVYNPSLYYNFKRIYPQH
>DmelIR85a
MSIQWLKHILLLAILVNLAGTRENHIPLDLKKSSIVMVKMSQILCKARIKVLFVYFENQT
SHEHTGQILKEVTKCDISNQNTPLEAVKDDGILMYMVMITTNISQPLELSLIRKKSAAKH
RSHVFLLVRDADTVSDAWMRASFRQFWKIWLLNIVILYWRDGRLNAYRYNPFMDNYLIPV
DNKPNEVPTLEQLFPKTIPNMQRKPLRMCIYKDDVRAIFWRQGTILGTDGLLAAYVAERL
NATMMITRPHSYNNHNLSSDICFLEVAKEYVDVAMNIRFLVPDTFRKQAESTVSHTRDDL
CVIVPKAKTAPTFWNIFRSFGSLVWALILVSVLVANVFCYILKSEVGRVPMQLFAGALTM
PMTQIPPNHSIRLFLIFWLYFGLLICSAFKGNLTSMMVFQPYLPDINQLGALARSHYHII
IRPRHVKHIQHFLTLGHKHESRIREQMLEVSDTQMYEMMRNNDIRFAYLEKYHIARFQVN
SRVHMHLGRPLFHLMNSCLVPFHAVYIVPYGSPYLGFLDSLIRSSHEFGFERYWDRIMNS
AFIKSGVKVVNRRRGSGNDEPVVLKLQHFHAVFALWLVGIGMACIVLAWEHLTHNYNLAV
TKRRD
>DmelIR87a
MSTPEQRFWLAALLFLLSQHSEVRGFGINLMKVQTEDKGQEACILALLRKYFDSGDGLSG
SVLCINRNYQLPNIEEQLLRGVNNYENYPWSLLITNSREGPSPAKFLMNEKPQCYFLIVD
NLEDEDLDEVFEHWKGMVNWNPLAQFVVYLASLEETDEEMNDLMVELLLTFINKKIFNV
N
VIGQSEENQFYYGKTVFPYHPDNNCGNRVISVELLDACDYPSEETDSEDENDEDEGDGAQ
EEDDGPQEEGDGEQEEEDGPQEQEDGDQAKGDEGQENDDGGLENKVENEFRIGASDDDE
L
ENDLSSNSSEPEAIIEEFFRAKFEDKFPRDLSGCPLTASFRPWEPYIFRNSEEQPVDDYY
YGLQGDEDDYNDTSPNYGESDDESYADPGEDGDGAIPDTETQSGGKLKLSGIEYEMVQTI
AERLHVSIEMQGENSNLYHLFQQLIDGEIEMIVGGIDEDPSISQFVSSSIPYHQDELTWC
VARAKRRHGFFNFVATFNADAGFLIGIFVVTCSLVVWLAQRVSGFQLRNLNGYFPTCLRV
LGILLNQAIPAQDFPITLRQLFALSFLMGFFFSNTYQSFLISTLTTPRSSYQIHTLQEIY
SNKMTVMGTSEHVRHLNKDGEIFKYIREKFQMCYNLVDCLNDAAQNEHIAVAVSRQHSFY
NPRIQRDRLYCFDRRESLYVYLVTMLLPKKYHLLHQINPVIQHIIESGHMQKWARDLDMR
RMIHEEITRVREDPFKALTFDQFRGAIAFSGGLLLVASCVFAFELCYVKYVYRTEKRERK
TKKITKKVHNIKIQHD
>DmelIR94a
MALPKQLKFINIFLVLLIIYGSSDGTENQHEIFLNRLLQAVHNERSVETLFLLHHSNLAN
CSLQDWNPPRIPTIRSNELTVFNVEKTFNHNALALVCLMKNSYREILNTLAKSFDCMRQE
RIILMIHRKSDSKFIEDITHEVKNLQFLHLIVLIVQEKYNGQVFASTLRLQSFPEPHFKR
IRNVFAIQRIFYRPINFHGKVLNAIPNDIPILFVALNEMFTEYARRYNSTLRIQNRTIKE
DIEITEDNYDIDMKIQLHNSQNFLHHMNIAMDIGSNSLIILVPCATELRGLDIFKELGVR
TLTWLALLFYIIFVLVEMLFVFISNRFNGRNFTMRYTNPLINLRAVRAILGQTSPISNRY
SLSIQHFFVFMSLFGTLFGGFFDCKLRSFLTKRPYYSQIENFSELRKSGVTVVVDHTTRQ

FIEQEINANFFRDEVPNVRTTTIQELINHVYSYDRKFAFVANSIPWRTFREEMKSINQKI
LCDSKNLTILENVPLTFSIRRNAIFSHHLRNFIINAADSGMITCWFKMAGKVIRKHIKTT
LRESEQQPSHLPLSFDHFKWLWAVLCIAYYVMSFMVFVMEILWSKYQRRTRSVSIV
>DmelIR100a
MATTLQLIMLALVGGTLGQANNTDHKQVLTSIVKQLEGGLELHLRTSEDGGNDLVQFLMQ
EKSSIIISAKQEEVPSRAKIMRHHFFIFDGVHQMQEIRTSLFNTDGFYILALENNTIEDD
VLLMEFAADVWLQHGHSRIYYVQLSKKSVLLFNPFLQRLVVVQDSKTYSRIYKDLEGYHL
RIYIFDSVYSSVIGDGENKVLSVTGADAKLAKTVARQLNFTADFVWPDDEFFGGRLANGE
YSGGVGRAHRGEVDIIFAGFFIKDYLTTHIQFSAAVYMDELCLYVKKAQRIPQSILPLFA
VHMDVWLCFLLVGLLGALVWLILRAVNLILGIEGVPDGSRATRISYFGAARRIFVDTWVI
WVRVNVGRFPPFHSERIFVASLCLVSVIFGALLESSLATVYIRPLYYRDVNTLRELDESG
QPIYIKHPAFKDDLFYGHNSEVYRRLDAKMMLVAEGEERLIEMVSKRGGFAGVTRSASLQ
LSDIRYVMTKKVHKIPECPKNYHIAYVLPRPSPYLEEVNRIVLRLVAGGIVGLWTGEAKE
RAKWSIQRFPEYLAELDVGRWKVLTLSDVQLAFYALTIGCLLSAIVCMAEILLGRQRRLH
SPK
>HoblIR25
MLNLFYLVLFIASCDRLRTQTIQNINVLYANEEDNDVADKAVDVALDYVKRNSKLGVKVDI
RKVVGNRTDSKGMLDSLCKTYGSMLDNKGPPHLVLDTTMTGLVSETVKSFVKALQLPTIS
GSFGQEGDLRQWRDIDDAQQNYLIQIMPPADIIPELIRSIVINQNISNAAILFDNSYVMDHKF
KSLLQNVATRHVIRPIQDDSAIRDQLQNLRKLDIVNFFILGSIVNIRKVLDVADSISYFNRKFA
WHSITQDKGDLKCMCKNATVLFAKPVMDTKYQDRLGLIKTSYQLTSDPEISAAFYFDLALQ
AFLSIKNMINDGGWPSNQSYITCDDYDGKNSPDRKGFNLKKYFGKDPTETPSYGPMNIARN
GESFMQFNMQLTAVSVRAGSSDKAVNLGTWKAGFNNNMSLIDPESMGNYTADIVYRVATV
LQAPFVMVDSEAVKGYSGYCIDLIDEIANILKFDYEIFVVPDGKFGNMDLKGNWNGIVKEL
MEKKADIGLGSISVMAERENVIDFTVPYYDLVGITILMKLPKTPTSLFKFLTVLENDVWLCIL
AAYFFTSFLMWIFDRYSPYSYQNNRDKYKDDEEKREFNLKECLWFCMTSLTPQGGGEAPK
NLSGRLVAATWWLFGFIIIASYTANLAAFLTVSRLDTPVESLDDLSKQYKIQYAPLNGSASMT
YFERMANIEAKFYDIWKDMSLNDSLSDVERAQLAVWDYPVSDKYTKMWQAMKEAGLPN
TLDEAVERVRSSRSSSEGFAFLGDATDIRYLELTNCDLQMVGEEFSRKPYAIAVQQGSPLKDQ
FNTAILQLLNRRQLERLKEKWWTNNPKALKCEKQDDQSDGISIQNIGGVFIVIFVGIGLASIT
LAFEYWWYKYRKTSKVITIQDNPQQSVQKHKSKPDNSKTMALGKLYPRSRF
>HoblIR76
MALIDIILIGLCANLTCPGDKIPDLSKRTRSLLREQEELIRNYTLKIATVHNPPLSIIETVDGVL
RARGIAFEYIEILQNKLGFKYELVKPPDNSLRPEDNGIIGMLSRNEVDIGAAFLPPFPYLSNYI
RFSTNLDKGEWVVLMRRPPVSATGSGLLAPFTFQVWLLILVSLFAVGPIIYFLILLQSRLCKE
DDNIIYPLPSCVWFVYGALLKQGSTLSPHTDSSRILFATWWIFITILTAFYTANLTAFLTLSRFT
LPISSITDIGTKKYSWVSPKGSAIEAALDIDDTFKQSLDGSHGQFSEEDASIILDNWVTRRDY
MYIGERPIVEHLMYRDYLAKIDMNIAEGDRCTFVITKWVVRDNMRAFGYSPEFPFQKLFDN
LLEHLVESGIVMYSLRKGLPDTQICPLDLGSKERQLQNTDLLMTYYIVAGGFIVSTVAFAGE
LLSKKCSGRVKSRKATASKDLFTISKKRKKGVLNEDHLFPPPPPYHALFKPPFPHSENAKTK
TINGRDYWVVKSTDGDTRLIPIRAPSAFLFQYDN
>HoblIR93
MLPVQLLIGLGFVNFVFSNNFPSLLSANATLAIVIDKEYLGDNYESIKSTIEDYIYITKRDRLK
HGGINVFYYSWTSITIRKEISAILTIASCADTWKLFKRANVENILHMAISESDCPRLPPNEGFT
IPTIVRGEELPQILLDLRSIKAYDWQSVVILYDDSLGRDFVTRVLTSLTIDTKEVTGGGTAVSLI
KLDKHNTGLDKTSIKNTLSTIAARLSGTNFLVIVSMELVTYVMETATALRLVDTNNQWLYVI
SNHKLNNKSISGILKNLKEGNNVAFLYNISTSGAKCKEGMECHIREMLDAFSNALDVAILDE

FELAGQVSDEEWDAIRPTKLERRNFLLDTMKAYLIKNSVCGNCTAWQMQACDTWGKEFK
TSSSSEFSLIQVGYWRLGSGPSMTDVLFPHVAHGFRGKSFPLISFHNPPWQIIKINKTGGANC
RGLVFDIINELAKSLNFTYTVIVLDNGQNVSGNNSNYNNEISYGVTYTIPERVIEMVQNKFV
FLAAAAYTVTEQSKTMVNFTMPVSTQTYSLLAARPKELSRALLFMSPFTYNTWLCLLLSIF
VVGPLLYIVHKYSPANEYNGMNVRGGLSSVYNCIWYIYGALLQQGGLYLPYADSARIVVG
AWWLVVLVLSTTYCGNLVAFLTFPNSDKPITTINELLNRRDTITWSITPASFFEYEIKMSNEPK
HQILYQGSTKGIKNFEEMIKNIELGKHVHIDWKIRLQYIMKKQFLSKSTCDLSMSKEDFFEE
KLGIIVAQDNPYLGRINREIKRLHQVGLIEKWLRDYLPKRDRCFKTKGGSGVNNHTVNLDD
MQGCFFVLFLGCSISFLLICFERLFYKYQKKREKKVVQPFIS
>HoblIR68
MCINVLLFYFLQIFVQESEDLLSPHNKTQKSLLQIKHDDCQTYIILISNGQQVSRLLRYGDRY
RILDTRAKFIMLHDHRLFHKSLHYLWKRIVNIIFIKEYVGMKKSGDTTIAIPWFEISTVPFPSPI
HNVFVPKRLDIWRKSKFRSGVNLFQDKTSDLRNQTLNIVVMKHTPATTNTLLVQDDTVRA
VMGDGSKGFAGLEVEILAAVSKAMNFKCKLYEAPNADVEFWGKKHPGGQYTGLIGEMIG
GKADVALGDLYYTSYILELMDLTVPYNAECLTFLTPESLSDNSWKTLILPFKLFMWLAVIFC
LFFCGIVFHYLAQFHCNINRLKRNAQNSDEIFPKKNQTLTLPTISIDQMNYNSKYFLMKQQF
VTKRETNDPEGLYLFSDFSNSMLYTYSMLVMVSLPKLPTGWSLRMLVGWYWLYCTLVVVS
YRASMTAILANPAPRVTIDTLQELVNSKLSYGSWSEVNKDLFKTSSDPISQTIGNNFQIIRNSS
MAVEKVSEGKFALYENVYFLKEAFVIRQLQVQSATNSTENQVTEQVSNKPGERNLHIMKNC
AINMPISIGLQKNSPIKPRADKYIRKVLEGGLIKKWLDDVMQPTLNAERPSTLQTTKALMS
MQKFIGAIVALFIGYLIGFAALCTEIIYFHYIVKRHPNFNKYSRRIN
>HoblIR75
RQKIKNIKFRVQQYGLLLCRSSKSFHFVKEKLKNLNLRIDSNLYVALKSTVGEYEVHEFYNP
GYKNGGTLMSYNSFNYTQRGAFASNYLSGYQMRKDMRSVHLRVAFVVTKKVSAPLEYLK
DQRTKQFDTSSKVNFGIFGHMIDIHRYRWDVVITPAWFGSKRHGVEIGLANVLHQNLADIS
GSSGLMTPGRVEAFDFGVGTYLFRTSFLFRNPYTYDDDVGEALVLRPFSRGAWLCAGLFAL
ILGLSLKLVFQFETRVLKRTSQTYSLCSIVVITIAAFCQQGEFYFRIDLQKLNDETTFSNIGSGL
HYNSVCGRVLLLQLLVFAYVLYNYYTSSIVSSVINSKKKTLDTLEELVKSDLDIGIERIAYARV
LNATNNPILFHLNNEANANNTRISFFPQSEGIQKIREGNFAYLAETITAYNDMDRVFSNEEICE
TVELDGARPTLIYIVGKKNGQYNELFSISYRQIVHSGLLQRQQKIFTNQKPHCLNAIQFVQV
GFGQLTSAFVLLTAGYGITLMILLIEIVHFRIKSGSYKNIQ
>HoblIR21
LGSSAPRILTSWRNGSLTRPQVKLFSKKMHTGFSGHRFITSVAHQPPFVIKRGLDENDDIVW
DGIEIRLLKMLSRMYNFTLDIKAAKDDISKSPADKVIDYVNDGTVNVGLSGIYLTKERLENL
DVGYPHSYDCAAFISLTSTALPRYRAIMGPFQWTVWLALTITYLFAIFPLAFSDKHTLKHLLT
KPEEMENMFWYVFGTFTNCFTFGKDTWTKSPKITPRLLMGFYWIFTTIITACYTGSIIAFITIP
IFPTTVDTIQQLLSGRFQIGTLDKGGWEFWFRNSSDKNSQKLLKNLEFLPNIESGLRNITKAF
FWPYAFLGSKAQLDYIVQTNFTTTNKKSLFHISSECFVPFGVSVVFGKYSIYKDIIDRGISYIS
QSGLIIKLEHDIRWDFMRSPTGKLLQANSMNTLKLISVEDRSLTLDDTQGTFLLLFAGFLGG
MLSLIFERVGGCFKCYKKRRFSTSSSISSKPRSYDEPTPREKLDSIQYSYDEFQLLNNVQENN
RNKTDVDAEFENMHHINKELQKTSTNLIVRTFNFDKLFGEENLNSHHETVEDH
>AplaIR75
MLKFLLFLMFCPTTYCNVLLCSKSFNLIKYFIKERHLTHIYIFDCNIFFKGLDRVYLVRLLSNL
RYLFFYLERVKLFNVLGEDRYRLFEYSFKSKTDHSLHTILANIKRDNSLQGNPGFVLDGDCA
SSGDFLIKAGEFELFNDSYHWLIITKKFIKKVFSNVNTNINSNLLVAECEYGNGVNVNVTLID
VYNAAYKKGGKLNYNVLVKKSNNFSFNLKSNNYNKYWNRKNMSGVVFKTIAVLVNKFEG
ELEEYILKENNYRVDSRTKFHSVLMNHCKEIYNFSLNFSLTDSWGFKLANGSFDGMTGALQ

RKEVDFGGSVLFLKKDRVYVISHGRSTWLLRTAFIFRNTNKKSFCEIFIYPLNYSVWYLAIFC
AIFVTIVLKAAVSTEKRILNDNFKDSNHTETSWSILFLFTLGAFCQQGTSYSSQLISGKFAALS
LFVFCVLFYQYYSGSMVSFLLTKQPSKIKSTEDILKSPLKVAVQDIVYMRAYFAETVDPVAK
RLYDTKIANKNSINFLSPENGLKLVQEKGLAFYVETSTGYPIIEDTFTDRTVCELEEVVMVPS
QTSHIATVKGSPFLDLLNFCMQYLTEVGITYRQRKIWDARKPNCVRASAATISVVDLSEIYP
VIALLFLGILSSLFILFVEIVHCKFMN
>AplaIR104
MRSNCFTLGCLLLLSAYRSNASLFIGEHSWKCFTDENKLFSNTITKLIEKSPNKALVILTSNK
MEVPDYLHDLMKVEIPHFIRSIIQEEVNKDVFTEMLSCSTEQNVGIEHLRCTVPETYHAYED
YLIIGRSYEDFRKILRAIYVKVSWNPRGTFILIWDTKLVDDMNSGKIDIVGGFLHNIGLTDRR
LEATFMASILFCYWLLPQRKILPEETLPSFSLSVKILLFLVIIMLILTLRFICRVTKHTSYKDLSC
SALIVFQVLLNGGGVTRMSRRLSFRCVIIAAIFLGVHFQVLFYSGKSSSLTQPTLTPKIKDISD
LATKTQLKFFFMNKAFYTILLRIENENVAKHLRERTQLELAGKTFFEYGVLDLYSQLEENLV
AATIPVDFVRQIKNYKELESFRYSTIMESFDVFVVKKGNPFLDDINKIMIRIFEANLFTKWYD
ESAKKVVINIKKYKIRPLNMEAIRPAVNIILHGYAIAFGVLVLEIMWFKYISKLSFK
>AplaIR105
MMVNVVIVLTFLINYAIYINAEESIIFKRFTVPSAESNSENNVRTVSDFINKHLNEKFTFITYTE
LSPFGQKILMKVNISQKLVCIKNNDKNVNSNSNDIGSNANIKNSNYMYDYLIFPNNFMDLLF
IMKFIMKSPTWNPTNRFLIIFECSLGSIINDLNNHMLDIGVITCEHGIKRELVEKTVFYYFAENI
WLFPRRKILKEWNIFFPIRYISYFCLSIITIIIVLVPLLTLHSRFLNDAIEMSLLNRTIFAFSLLTTA
SGKLLSKSISLRLLMFIAIIHGFYLNLVYTSLISSIVTNPPREAKIRTFEDFLKSHYQMNFYEGL
FGHLEQKGFKGLQEAMKQKWIKPSSDAFYSEVVNVIKSQKYGIVVNSRATKVITNYNEGE
KLKLFHDFYYECFLVRKGFPYLDKINEILMWTFEAGIFGKWLNMDMATLPHVAIPSATYEAT
KLKLQDFTGAFIILGVGIVISVGIFIAEIIIMYVVKR
>AplaIR107
MTLNVVIVLIFLIHNVIYINAKQNIIFKRLIDFTDQNTRENDKTISDFINKHLNEKFTFTTYTEL
SPFGQKILTKVNISQKLVCIKNNNENDDSNSNDIGPNANIKNSNYMYDYLIFPNNFMDLLFI
MKFIMKSPTWNPTNRFLIIFECSLGSIINDLNNHTLDIGLMSEQEVKTELVEKTVFYFFAENI
WLFPRRKILKEWNIFFPIRYISYFFLSIITIIIVLVPLLTLFSKFLNDTTEMSLLNRTIFAFSLLTTA
SGKLLSKSASLRLLMFVAIIHGFYLNLVYTTVISSIITNPPREAKIRTLEDFLNSHYQMNFYEG
MFGHLERRGYKGVQQALRQKWIKPTNEILYGEVVNVIKSQKYGIIVNSRAAKLIKNYNEGE
SLKLFHDFYYESLVVRKGFPHLDKINKILRWIFEGGIFEKWLDMDMVTFPHVAIPTATYEAT
KLKLQDFTGAFIILGVGIVISVGIFIAEIITMYVANR
>AplaIR110
MVYSESYTKMILRNTFATIVIIFTLIGNLSASNGLMDALVKTDYYNSDSDIKTLSRIIAKQLNT
KYRMLLYTKFTSTIDGILKNSKMPYKMVCIGKEDSCFRSFENKFQNVESNYLYDYLIFSNNI
QELSATIKFITSSKTWNPSNRFLIISESNMIVKDLTTNRIDISPNVLFSYKVPTNSVSVTFFYNI
MEGYYLLPQRKYATYWATITQFSCLTYFWIGAIFITSIIVPLLLLRLKLLETSFSVPLIHFTTDIF
SIVVTGSGNVSHKSLTLRIMLLTAILYGFYLNSIYSATVSSVITQPPLEKKVKTFKELYETKYDI
HFYEGHFQFLRETGHPKMAESLMKKWKKPQSKRGITEMASVIKSQNYSIVTNSYYLKFIKN
IDKVEKIEFFPIQYAYVGFVVRRGFPVFEKMNQILQWVFEAGIFGHWQFQEMSAYKHVAVPE
LQNETRPLKLKQILGVFIALAIGLAISTLIFIGELVVHLLITKKIISK
>AplaIR111
MFPTIVMIVLVSQNYLTINGEMIIEYPKSEQYRQHLFLTFEKYLEPSKVNFCMDCEEFIHLIPR
SVALVMISRKDKIINWPAYNKVDSYILDLTRETKERLEFLERTPGWNPRAKFFIVYRKKARS
YLLKVLFSKYIVNVAIFSNTDNTTLTIRTYDPFKNGELRPKILKTTIIATIKETNREIDLGSDLFP
NKYPQRWNDITVNVLPVNVAPYMMIFSGQLVGLDFRILISVGRRMGIKFNFSSNDMLHYW

GEKDNDGKYSFAFGALQAQKFDISVGAFHVGYPECVDFDMAYPVMEDYLVMAVPTAPVK
ERWKCIEPVWISLLISVFSMTIVFFLVSITKTNYEPSTSFLICLGCFLKVCLPKMPKSKSQRFLL
MFWIVFCFLIHVYFESHLQGAFNYVHREKQIDSFDEIKQLGMKVAMKKKYLQLFANEETEI
DRFIARNHYETSSTLQALNESLKYKKTVTIALERFIKFNIAHNYVDSKDGSPTVHIVTEKIMF
YPLTVYFTKGSPLYDRIQTLFEYYRAIGCINRVYLYGEHLDQIFYHKYATTWKIEPLNLGALK
YIFWMLLGGLAFSSVYYFLRYYGTNSSPTGIEF
>AplaIR112
MFKLIVAVIFVSQNCLKINGATFFNHPTPEPYQKHLFRVFEKYLEPDQVNFCVDCDKLIHLIP
RTVSLVTISQNDKTINWPAYHKVDTYILNLTRETKERLEFMEKTPGWNPRAKFFIIYSKNTIN
YLLKILFSHYIVNVVIFSSPDSISLTIRTYDPFRNGELRPRVLKAITIGTINETNREIDLKKDLFP
NKYPQRWNDITINVLPVYSPPYIMIISDIGLVGLDVLNLHAVGTRMGIKFNFSSDHILSYWGE
RGNDGKYSFAFGALQEHKFGISVGAFHVGFPEYADFDMAYPVMEDYLVMVVRTAPIKNRW
NCLIDIFEKPVWISLLISVVLMAVVFYIRSINKREYELSVSFLICLACLLKLTISKMPKSNSQRL
LLVFWLVYSFIMDAYFESHLQGALNVAHREKQINSFKEIEEIGMRVAINRRDLKLFSNDVTQI
DRFVSRNHYESNSPIEVLNKSIISDCNNGVIVLERFIKYSTARFYLDSEDGSPTVHILTKKLMF
CPLTVYFTKGFPPFDGIQRLLHDFKAMGYIEKLYRYDRHMVQLLSYRKGIKWKVEALNLA
DLKYIYWLLLGGFAFSSSILLLEMLWHKFGKLKRLNLV
>AplaIR113
MFRLAILLHFVLLFAENDAKMALKSGEFDVYSKYMKIIYEKHLNPQDITFCMDCHHRLTDY
IPKEIPLIMINSKSKQLQWYQNIKIDSYILDISTETENVLDLLEEMPGWNPRAKFFIMFEGGSV
SHLLKQLFAKYITNVVILTPVNISTLKVLTYFPYKNGQLRPKSIKSTTIAIFGDEYLNDTVDLF
PDKIPRKWKNATINVMPVYAPPYMMCPTCPSMRGLEFLLLDNVGRCLGIKFKYPNNVSNA
WGRKTINGSYTLGMGAVQERKFDLAVGGYHANSPDNVDFDMIYPHMEDNVVVVVHSAN
VEARWKSTIDIFQRPVWILLLIVLFTMPIIFLTFSLFRKKGEFCSSILISLSILLKMTITRMPHTK
AERVLLMFWLFYNLLTDIYFESHLLGALNKAHREGMVDSLEKMVDMKFTVMVHSTLSKFF
PNNDLEIDRYIQRNSKKTNFNLEALNASAADHRTAAVAMERIVKFYIARYYVNPEDGTPMV
HLIKKKFMFVPMTMYLSKGFPLYDQINDMIHELKSKGLIIKLYHYSQHHNLINFYAFKRVTW
KSDSLKLTELKYLFWMLFIGLFCGNVAFICEILWYRFKLRNDYRNYG
>AplaIR115
MNLFLQLILQFHFIFTVNTIGLLPVEDGEFYLNECVNKISAKYFTNTGPVLMINVTLSNFPLQ
TILQTDFVNFNKTLFYYGNACIIDQRGQKIEDFLKYVATTLHWKPQAKYIIISDENPQEIFKCL
SPHFVINVIVLYPKSSKMVEIYSYNLFGENGEILYPVSKLLDVCIEGVLQDNIDLFPNKLPKN
WSKMVVKADEVTFLIKIFKDHNVFGEVMSAIAKKMKYTLKYIDLRPNRSYFFHRELLNNC
QIHMAFTDNLQFLDKIYPQAPDRLVWVIPKANEISGWMRLTYIMDRTQILSILFLFCFIALAK
FLSDIVNKKPHDFISSLLFSYRFFVSSSARSKNQATSRNVLDLFWAIFCFIYTTIFMTHLTVYM
TTRSFDRQLDTAEDLVKSRLKIGIRERFADIDLFQNNNSVDNYIRMNHYNCDDFQKWLHWA
ANERNVTCIFSENSLKAIYNKVPSEMIPIHIGSETIIATYYSFVFCKGFPVQDDIRRHAASIFES
GILSNYLNMFLTRKKRVSHKRTDSNVFLTASDLSCGIKVITLGLGISLIIFAAEFCYYYGLKFY
KERGPFFQQRFEFRNRPW
>DponIR108
MLGKILCLVSCFCCSQSANYSEDASLLSCLVKGLDKNFVNFKPILVSLPVDSRSRVHGSEILI
GLRLWKKWPIILANINREALEAFKDHRTDKHENYLLYFNGSQEALKGFQALDAMLLLNPH
ARFLIVLHADVQPEDWAGGKIIHLLYQYYIFNAVIYIGKNLYKPQPFFEKSCKFNAEARLKLV
DTCKSGKWLKDVHWYGESVPNKFESCDLKISYIEGPPHVINMENVSILDKNEFLHHGFEIGI
LANIFAHMNATLRFTKSEIIGDMYLNGTGTGSFLHLLRKDVDIVVGDYGATAPRMTFFDCS
VPYSADGLVWVAPHYLVYSTGFISIVKTGVWVLIGLFIFMVSSIITVLGKYAENEHALYKQPF
LVFSNVLTVVCNLPVATLPRNHRIRIFYPMILLLGFVFSTCYMTYHMSLIARDGLRKDKFKSP

LDIANYKLKVYIVPNTKRFFQDKSSTVESFLVENAQICALKDFDTCLREIALLRSAALVASRP
YIEYVQDNYIVEADYPPFVFFETGIKGSIFFYMAKGFWGVDRLNGLLYKAFEAGLIDKWTNI
AISSKYMGRTDKSKLSFSGKPLNLRSCAQFCYGLLLAYLIATVVFIGELVVYRKTHSF
>DponIR113
MAGLAISLLLANAALLASSKLNSIPKRVPQVNLDRFLKKYYAESYTNVVIADYHDDLTSASE
ELLFRTNGAAVKSFLDNLEQTPRTFVIFPFGSSGLTEIFGNISESSMGLPGASKYIILTREEELV
EDFAQILWKFKLPFSFILAYKEAVADAKVFNLNTWNCGSNISSSFISSLNGGSDLSTIHEWNF
KSQFAGCPIYALWTVMPPYVNEVNTSRQGIFVEFLDAITSIGNRPIVYRPYDPVYLQELSEGY
NFATLLEDLDGEFGQLFVGPVNIHVTLIFDISPILIDNSLLFLVPMPYSSLTEQMLRPQLITLAI
GYITLIVTMVVMLYYMSKNTVDHELFSSIMKTYFLFSGPILGISSMHRLPKTRSLQIYVGAFL
LQSMTISTCIQGSIVSELSGTIYGDPIENNDDLLNSNLPIKTISGVAFLFRIGFHERDGEIYKRIQ
ALEGAPYPFQAGNMAVEKKDFATIVDTGYIIMRPNILRSFSVFEVYGMKQNLAMPKNSYFT
EQYNEWIGHLMEIGVLSKFIKLYQYFYALKFYVEEDKRSFVVTLEQLLPVLSIVGAGYFLAL
LIFVMELAVDRWQTIEFKPARKSRKISI
>DponIR114
MLLGAVVFLLALLGVEGKIRIQLPTSSQCLREVLAHVGPTRSIQILSGDYADLKGQTLIGAPS
LAEAPQVYWIASQSSEQLNVTLKALVEAPEYKSATFVIVADPTTEQSAVDILWKFRLLDSLV
VSSQGIFTVALIKCGATVRAKKVGSCTSPDLQEFDFKQAFLGCPLKVLWVRYPPYMYEPNQ
TSGWKGIFVDFLEAVGHGLQRPIQLAPSDADYLDEVYQGYSYDSVLRDLQEADLFIGTADT
RTAELFYLSPIIVSDYVTMLAPRALVDYWSHLRRALSLTLLILFGAMFFSMAAVLFGLSQLR
DGASRVADIVMLLYGVSMGLSGHARLSDYSPLRLFLGLFLVVLMVLSFYLQATLTSVLSGPT
YEPSISSIEELVESGLPLKLTELFKLVFEFGTGSYQKPHMSELAKRIEVLDEPLLLNVLDAMIR
DRNFATLMINAFMFMRPNLEGTFGSFPVGNIDLCFAMGKNSYASPPLNAWIARALETGFFEK
FKRLHHFELALKWNVEAAGRTFVVSWGQIAPALKVLGYGYGSAFLVLVLELLLAQLHSRL
K
>AcorIR1
KNHRLPFTIKDAAIRHDDRNGLQLSHKTCNLLARGVHTIIGPNSYPMSRHVGVICSGKDIP
QVLTRSYSSDEDYNNFAINLHPHPPILEKLFVELLNKLQWTKFMIIYQSNQDLVKVHQLLS
YKSNIYDIKLSQLILDDRMSYRLMLNAIKKSGECHFVVVCDLLTLKRFLQQAQQVGLLTE
KHHYVIYNFDMSNIDVEPYQYGGCEIISVRFFDPYSTEIQDAFNAVDEELYSNYGIETDGQT
LNLETALIMDAVKLIHTTLKEHMLPEHIDNQPLHCNDSEAWWHGPSLRNYLNVANVKGY
TGLIKFDPKGYRSDFEADIIELKSEGLTKIGTWNTTDGLIIERPEKKDPLPDDLSDVRGRTLN
VVSALTRPYGLMKQATTRLYGNDQYEGYGIDLIHELSKELGFQYKIIPQEDGVNGSKDNK
TGKWDGMIGKVMSGEADLAIGDLTITSERENAVDFTLPFMTLGITILYKKAEPVPPSLFMF
TSPFSPQVWLLLIVAWIFVSLSLFVMGRLSPSEWQNPYPCIEEPEYLINQFTFKNSFWFTVG
SLMQQGTELAPVGISTRMLAGVWWFFTLIMVSSYTANLAAFLTVTTLNTPFSSIDELAKQ
DEIKYGAKANGATAFFFKDSDKPVYQKIWKYMNNNPDLMVKDNMLGVNRVLKENYAFL
MESTTIEYITERYCTLAKIGELLDEKGYGIAMKKGSAYRQRFNTAILKLQETGMLTTLRMR
WWKEKLNGGACDERSSTATVTALDLQNVGGVFLVLGLGAFFGVVMAVLELSMDIMRYV
KHQKAKYKQQMREEMKFFIEFKRNVKPSRKTGQENEDAVEFPFNINYMENYINENNQNE
>AcorIR21
QADITDSEMKLRSVIITIFTINIAMAKLEKRALQKAHERSRIDKLMDKFLPRQDYDKTTSL
AELFIQIFHDYLSECVPIIIYDDKINQYYPLLDIVFQKVNISFIHSMVVVKNGKNVDRNFTYT
PDTHCFNYILLVDDIFSSKYLIGKQSTPKIVLITNSSQWRVNEFLMGDFARNLVNLLVIAPST
SPSITETDICYILYTHDLFVDGLGSSVPKVLTSWRDGELKRRHVDLFASKMKKGFSGHRFIT
SVTHEPPYVIQRGFDENDGIVWDGIEIRLLTLLSQLYNFTIDIKNFKDENFKSPTDKIIDNINN
GIVNVGLAGLYLTVDRLEVGDISYPHSYDCAAFISLTSTALPRYRAIMGPFNWTVWLCLTI

VYLMVIFPLALADKLTIKHLLKNPEEMENMFWYVFGTFTNCFTFGKDTWTKSRKLTPRVL
IGFYWLFTTIITACYTGSIIAFITLPLYPQTVDTVAQLLAGRFRIGTLDKGGWEYWFQNVSD
PQSQKLVKYIEYLPDIESGLKNITRAFFWPYAFLGSRARLNYIVQTNFTTTSKRSLFHISTEC
FAPFGVGIIFAKKSIYKNTIDKGISYLQQAGIVSKFESDIRWDYMRSPTGKLLQASGSTLKM
LTVDDRSLALDDTQGMFLLLGAGFLLGFFALISETLGGCFRCLKRKRCDSSISSIPSNPRLYS
LPTPRESIDSIQFSNNIQWDFAGEKMKTNINDNSRIDLHNFEKFFGEHINLIHYRRVSDRVNI
AR
>AcorIR41
MYLQINILANFIINKYFNNNNCLLIITDRNNNFEYNGNLSYVYVKLNGIEIPYNLVFRSYGC
QGIVITCQSPVSIFENLELGMKLGSDRFNYRKYLFLTVTNQLETSLDVLKSKAAEFVADILI
VAVDSDESIFDLYTHKFAGPKEKSTDITWLDRWYSINNSFLFDSNLYPDKLENLEGRPFRIIC
FTYKPYCIIDPPDGTDMLVALEYARKHNMTPELVVDEAGEWGNLYDNWTGNGVVGNLA
QDMGDIGLGALYTWEREYSFFDYSKPTMRSGITCIAPAPRLASGLATPFVSFSMELWIMTL
SSYFLASVALLIVLSVTISDENDKKRNVNNIMLSLSLAGRIFLLQSFQKVPNLEQSRITFGLA
LILSLMLNTIYSSGLSSTMTIPRYYGTIHDADDLAASEIKWGATSTAWIESIDSDSRKVFVHI
VKNFQILTEKELAAFNDEDLAYAVEHLQGGNLALGSYISLDGIQRRRLLDEDLYWEYCVL
MLRKNSIFLSSLNDVILAVTESGLLYYWEHQTVYKYMDMNMQKAVKMSLRANNPGGSN
SIVKLNLDHVIGAFTIWGVGILISIIVFICELIKNKYNRGDNKV
>AcorIR75
MLLTCLDFGLSITKHNSIINFLSSYAKSKNVMLTLHTCWNKDMFLDKLLDFSKIANYGNDY
ATFVSTHTHIFQKYSFNNLLIIIDLNNCPKAWSSLLQMNQTDSFAAPAKYLIFIDNIADIESTI
RNFTIYPSSDILIAEKHASDYKLHGIYRINAISDLIWENYGYWSCEHGLVEYLKKYPPSQRR
HDLKQQNLKVMIRITNNDTWNHLEDFRYTSVDGFTKLSYGASKCLFEYCNANVTYLGTE
FFGYKDKTGNYNGIVGALMRGEIDTSGSPMFTRIERVPLITYMTMQSPYYIKFILKKPPLSY
VKNIFFSAFDYKVWAAVIVSFVVLVIVSLFIYNTEAKQAKINFQSKLTISDVILVSLEVLSQQ
GTYMDFQRMSSRILILTFLVAFFFIFIAYSGNIVAMLQAQVELKSTKELVDSRLDLGAEDINF
MQLFLSQDTDAVGKQVYQKIGENGYYPLAVGMEKVRKGFFAYHAELAEAYYYMREKYT
NNEMCCLQEVEGYFQYLRGYSVTRKRSPYKEIFKTGLLKVDEYGLKLRHYNLWYIKPICH
TKGSNVGSVGLIECRMAFFLLIYGTLISMLFLLAERIIHYTQTTL
>ItypIR25a
RGNREKYKDDEEKREFNLKECLWFCMTSLTPQGGGEAPKNLSGRLVAATWWLFGFIIIASY
TANLAAFLTVSRLDTPIESLDDLSKQYKIQYAPVNGSSTMTYFQRMADIEARFYEIWKDMSL
NDSLTDVERAKLAVWDYPVSDKYTKMWQAMKEATLPPDLETAVERVRKSKSSSEGFAYLG
DATDIKYIHMTSCDFVVVGEEVSRKPYAIAVQQGSPLKDQFNSAILQLLNRRELERLKEQW
WNRNEESKQCETSDDQQDGISIQNIGGVFIVIFVGIGLACVTLAFEYWWYKYRKNSNITNVI
VSDPKHRRVAGFPKDVGGKANEGELALRPGKLYVKPKY
>ItypIR64a
LVKLGLNLVKQGHYAFHVELVTGYPFIRKHYSESMVCELKSVSLFPSMFMHANYQKWSPF
KDLLDVCLHRLGENGVINRELIFWHPKKPECIRSSSTININTGLESFYPALVVLLLGILASLNI
LLLEILWFKYQKRQILPYTE
>ItypIR68a
VRGDTTQFKDEGNQSVSNKIRANIMLSNATELFSGLEIEILDTLSKVMNFHCELYEPDRADT
ELWGRKQYGGVFTGLLGELFTSKADMALGDLYYIPFILDVMDLSIPYNTECLTFLTPESLTDI
SWKTLVLPFS
>ItypIR75p
QSRFIAEREMYEENAPDFLDIVLMQIGVVCQISYSFKPRSTAAKIATLSLLVGFVYIYNAFCA
RIVILLQSTANNLNNYKDLYYSKIDMGVEEAPYNKYYFSNPNNRANEAWRKLIYETKIMSK

NKHPVFYSTAEGLKLVKQSYFALHVEYTTATDVILATFTNEEMCAVRVIESIYKEDVPYISCP
VNSTFTEYLLIGFHRLFETGLHSREARRKFSKLPKCIGRNSIFVSVGIIGCYFAVEVFLVGVILS
LVIFAVELV
>ItypIR76b
QSTGVSYTKLFDFTIQHLRQAGIIQFKHRELLPDAKICPLDLGSKERRLRNSDLAMTYQIVG
GGLIISS
>AplaIR8aNTE
CSSILSGKIPIIIDFTWNGNDVAQKMSIEAQIYYLRIDISIAPILKFLDMYLERKKANDVTIFFD
DDFYVEQSIFFWLNSPRLRLFITTKDGKNLKQKLSKIIPIPSTIAIIANTKNMINIMTNAVEQN
AFKIPDRVIILFSDLNYDRFSLTILNNTSISMFKISKSLCCFLQEQAVCTSPENFDIQKTFLKLSL
QKFIIIFKSNYWGNYTFPNMHSCNETNQERHLLKLKIQNDVSMMIGEGANIFETNDNVLEM
KLSGTIEKFDEDKGDVNIGSYANKQITTNFTRTIEPIRKFYRIGIAESIPWTYTEIDPLTGNKYL
TGFCIDFIKKLSEVMDFDYELVIPKVGFYGNKNEFGEWDGVVGDLVRGETDIVVTNLIMTA
AREEIIDFVAPYFDEGGISIVMRKPVRKTSLFKFMTVLKLEVWLSILAALTLTAIMIWFLDKY
SPYSAKNNKKAYNYPCRDFTLKESFWFALTSFTPQGGGEAPKSLSGRTLVAAYWLFVVLVL
ATFTANLAAFLTVERMQTPVQSLDQLAKQSRINYTVVESSQTHKYFINMKYAEDTLYRMW
KELTLNSSPDDSRYRVWDYPIREQYGHILIAINDSIPLKSASEGFRKVNERTDADFAFIHDSSE
IKYEITKNCNLTEVGEVFAVKPYAVAIQQGSHLQDDLSLAILDLQKIRYLEYLQSKYWNNSIQ
ENCPNTSENEGITLESLGGVFIATLFGLALAMVTLVGEVLYYKLKPKQCYVHPINKNRNAK
KCKLSNKKTITIGNTLELIPDRNLTPQISYINNYPRSRFAKFEHQQKY
>AplaIR21a
MKVVVVLVVTFLISASVCLKISPNSTRYVRALQKEHEKSQEDKWIDAFFGKKPASNIQNSLA
ALLLMISKTYLYKCATILLYDQLSMKTYPFLLDKLLREFSFDFVMGQISEDYKLLNADLLQS
FPNKCVNYVLFVYDIMKCKHLIGIQHTNKVVIVSSSSQWRVHEFLHSDISHSFVNVLVVTQS
DKIVSENEELPFILYTHHLYNDGLGASAPIVVTSWMNSRLTRPQQKLFPAKFKKGFSGHRFII
SLANQPPFAIKRQSEAVIKQIIQRKANLGIGGIYVTDETISKVEMSQPYTQDCAAFISLTSTAM
PRWRAIMGPFHWTVWLILTLVYLLAIFPLSFSDRHSLKYLFRTPEEMENMFWYVFGTFTNCF
TFKGKRSWTQSHKYATRILIGFYWIFTIIITSCYTGSIIAFVTLPTIPETTDTAQQFLSGHYQIGT
LDKGGWQHWFNDTTDPVTQKLLKNLDLVPTVEDGLKNTTKAFFWPYAFLGSKAQLNYIV
RTSFTTKNKRALLHIAKECFVPFGVSIIFNKNENYREIVNKGLLRAVESGFLVKFTHDVEWD
MIRSSTGRLLQANSGGSLKILIEDRALTLKDTEGMFLLLGFGIILGGISLLTEWLGGCFNLCR
RRRLSTSSLSSNPFSFDDLTPREKIDSIASLHYREENLTDKKHEKLNTEEMEDKITDYDIKECF
DKILNFSEKLDGFNDKEKSNEEGSKCDTKVIKIGIK
>AplaIR25a
MCFFRSVFIIFVTCSCFTKYCLAQTTQNIVVLFVNEEGNVVADKAVDVALNYIIKNNKLGLK
VDMQKVVGNRTDAKGLLASLCRAYQGMLDVKQPPHLVLDTTVTGMASETVKYFTAALAL
PTVTASFGQEGDLRQWRNLDEDQENYLIQIMPPADLIPELIRSIVEYQNISNAAIIFDDSFVMD
HKYKSLLQNVATRHIITQIDDRSTKEQLQNLRKLDMMNFFILGNLLNIQRVLDSANSLEYFS
RKYAWHALTQDRGDMKCNCKNATVIFARPFVDNRFQDRLGLLRTSYQLNAEPQISSAFYFD
LALHSFIAVKSMIADAAWPKDMNYISCDDYNEKNSPVRKDLNLKEYFGKDSSESPTYGPLT
VTRNGESYMEFQMMLSAVGVRDGSSDKSAYLGTWKAGFGNSLQLKEPEIMKNYSADTVY
RIVTVVQEPFIFEDPDAPKKFNGYCIDLIDEIAKQLNFDYEIYLAEDKKFGNMDDNGNWNG
AIKELIEKRADIVLGSLSVMAERENVIDFTVPFYDLVGITILMKLPETPTSLFKFLTVLENDV
WLCILAAYFFTSFLMWIFDRWSPYSYQNNREKYKDDEEKREFDLKECLWFCMTSLTPQGG
GEAPKNLSGRFVAATWWLFGFIIIASYTANLAAFLTVSRLDTPIESLDDLSKQYKIQYAPTKD
SSTMTYFERMANIEDRFFEIWKDMSLNDSLSDVERAKLAVWDYPVSDKYTKMWQAIKEA
GMPATLEEAVKRVLQSKSSSEGFAYLGDATDIRFLELTNCNLQMVGEEFSRKPYAIAVQQGSP

LKDQFNTAILTLLNKRQLERLKEKWWTNNPKAKKCKKQDDQSDGISIQNIGGVFIVIFVGIG
LACITLAFEYWWYKYRKNAKIINVQETNPKKPHQQTKIKVKEHADTSKLNNSEDNKFNSS
HLRSRF
>AplaIR40aNI
YLNFLKEKYIRYEELATLFLCNNRLSEHILVEIHFNNFIRKNFYFIFNWGKDTISPSFLEYIHEA
MKIIVITNPRPNAILYNQIKKNGNTEEKSYLEMVNWWNQMDGLSKHPTLPSVYDIYKDFRG
RELIVPVIHXVKYCDPPERTQGVTFSENGTFDGALGLIWKREADLFLGDVALTLDRFQVAEF
SFITLADSGAFITHAPSKLNEALALIRPFHWQVWPVIFAILMLSGPVLYALVILPNTWHSTFLV
KSKGKLFFECYWYSISLFLKQTIKVPFDNDKVRFFTIMVSISATYIIADMYSANLTSLLAKPG
RERAINDLYELEEAMIKRFYRLYVEKHSSSYSLLENGTGVYRRLWHLINQQRSFVVDSVEE
GVMLVKESNSNNVLLGGRETLFFDIQRYGAQNFHLSQKLNTAYSAIAFQVGCPYLENVNSI
LMALFEGGILTKMTENEYAKLGRRRSVVNEGTAGAITPVGKKEIRSVTKAAQDNEKLQPIN
LKMLQGAFYLLFFGCMLSGLILGLEIFTHKLNVRSKRHENYRICAKSKCWQMIVRITCSKR
RKLANYIRMRYRQFISEAAIETLPYLD
>AplaIR41aINT
MEVNPGLNLLVSTVINTYFAHLYCLFIFNDNVSTFAYKGSILPSVNYKIDDTTNKTDFKISDN
RCEGIIVSTSNPKWVFRKIEYKIKMSNVHRYNRRMYLILLTQIESEEFVNDLFNTTELFYVNN
FLVIRAANIKAEKCDNFTMKCKENVEYELLTNKYVGNKNNMNTILLDKWFSNNNSYLFNS
DLYPDKLVNQEGRPFRLASLHYPPFHIVEDPANESVGAETNFAKEYAKRHNMSVEYVIDEV
GQWGNIYDNWTGDGLVGNTAMDNADIGFGAVYLEWFPLYSFLDFSHPCIRSGVTLLVPEPI
LAGGWTVPLFSFSTEMWIATSISFVCTIVALFVVTLYTRKYFRLEGXHFSVPVFIKHDAIQHL
RPMTEDIFWAYCIFMLKKSTPLLPSFNMLVLRVLEAGINYAWETKVVLFHTNSRTQQIIRYH
YYHGEDTETVSLQWMHVQGAFGILAFGYAIAFLCFLIEQVVHKYKTPT
>AplaIR60a
MPLIYVLLFSLVSAKTDIYSKLEGRLELQEGIEDATACAIFTIEQYLSNRINAVVSVWTTRNAS
NDVSNFIEDFVELIFKKDWSAEIFKVPVAIENDDVINVKANVHAVILDDILPLAEVVTNILED
KFFNFESRLLLILKTPSEFSDIMGAVCKNNRCIVPIFVVMLPSKTNPKETDIYFPKYRYSKNKT
CHQTPSLQLVNRCKHGKSISHRRTIKQIYSTNTFNKCYMEVITFKYPPYVTSEKEGLCINIILK
LGDYFMIDFRIELLENNYSSDNRSLIIIKNIYDHNKFAIGDMPFHDDTSPNYSLSKPYFYEHLV
WVVPKATRVQKWEMLHAMFPLQLWLLILFVTFSSSVLLYLFSKITKEATPGDDLASCILTGF
GILVSVVVYLQPRHVLSRTVFMSLVFFALIVTTSYQSELFIYLTRPVHHKQISTLQEVIDSKLK
FGLENKYHKPLVSRLHLHERDLVSYGQNYGEDDWLLQVAKYRNVCTVSNEIYVKTLMAT
NKAFQNEPVEAQRQTNPKLYVFKSNLGITAFHIVMPKGYFLKEIVDDQLFAMIESGITTKW
MNDYLFPIKLMEAMDETNLRDIQISMVLGLDRLQTAFIFFGLGTLLSIICLFAEIAIYNIFDRY
NENKKKYHDGKN
>AplaIR68a
MYIFMFFTFTLRYVISFRLITDKHLTKISKGNDKNLTQLISNIFEKYMESYRCALVVMDDFYY
ETIQSDFFKKLQQRVSMITIKVDDYEDLFDPCNETLSSLQLGKTTGCQFYLILILDTIQVTNLL
KFGDSRRIINTRANFIINYDNQLFYEDMKYIWKRIVNVIFIREYGGIKRMGSKRKRIPWFEITT
VPFPAPIGNSLVPRRLDIWRDGKFRSGIDLFRDKTNNLNKQKLQVVTFDHMPAVIKVPSGKG
VFSTKLSSTVSSDNFEFAGIEMKILEIVSKAMNFEPELHEPDITNEYKLGEKQSDGKYNGLLG
EMVASQADLALGDLHYSPQFLDLMDLSVPYNTECLAFLTPESLTNNSWKTLLLPFAPTLWA
AIFICLFFIGFVFYVLSRYHNKVNEMIFGVKKDRKMLFRNVLGISKDTNGKLDVNAKYALM
KKQYYVEQKEIEPVGLYLFGDIPNSILYTYGMLLVVSLPKLPTGWSLRVLTGWYWLYCILVV
VAYRASMTAILAKPAPRVTIDTLTQLVDSGLPTGGWGEVNKNFFATSLDPLTKYVGDQFETI
QNLEETIERIVQGKFAFYENVHFLKQAAVKRQLRFQNNRTKTGNATIHKEDLERMGNKRDE

YRINTKHNLHIMSDCIIHMPISLGLQKNSPIKPRVDEYVKRAFEAGLIKKWLDDVMAPYITA
ELHFQEKGDVKALMNLEKFFGAIVALFVGLSISIFLLITEIAYFTFVTKKNPNYDKYLRIVVIK
>AplaIR76b1FIX
MLLEDVFLATILLNITNDSYVTQKSVNVYSNAYDIAVDAIKNKTFNVATVHYPPISFLDGDK
GSGIAFHIFNAMQERFQFKYEIVQKKEDTMVSITEISDKYGNETDIAVAVFPVNGKINKNMIY
SVTIGSIQWHILMKRPKLSANGSGLLQPFTWEVWILVVITLLVVGAAMYFIAFIQSRFEKRV
DKKMFSLQSCIWFVYGAILKQGSTLNPVTDSLRIIFATWWFFIMILTAFYTANLTAFLTLNTFT
LPINNIRDIGRKGYTWCTQVNAVDNITNDPTEELSKALVGSTKKVVNTSDTINFMNNYVIGK
NYLFIAEKPLIDIWLYKAYVKKVAFNPNDVLSDCPFAVTDWSLLTTSIAFAYSKKFTYTFLFD
KALQRLVEAGLINFWFRNMLPRAQICPLNPKVNERRLRNSDLQLTYYIVAVGFAIAALFFCA
ELVNNIIKKRRHTSIVTIPQNMLFTKSPPPPYSSLFGPPFPKEHNYKVKRKEINGRDYWCVTS
VNSKTTKLIPVRAPSAVLFNYTKY
>AplaIR76b2NTE
NYPLSYAELVNDTWIGKGIAFDIIKYLQNKLGFTYTVYLPNDKTVDSDEGVVGMLNKNKV
DVMAAFLHNEFFRNLGFPSSKVLDMEKWSVMMKRPKESATGSGLTAPFTWDVWLVILLFL
SFIGPIMHYIIRYRDHLVNDHKESSIYTKFKCSWFAYGALLKQGTTLDPKSDTTRILFATWWI
FITILTAFYTANLTAFLTLSQFKLPINAPADIGQKRYGWLTQKGDPIVDLLKDKRQALHHALK
GSKSLSLDQERSEILQNYVFLKNFLYISEEHTIQYLMYQSYINNARKGIAEEGRCIFVMTNW
EVVRLNRVFVYSKNFSYEEVFNRQLLLLTEGGIILQKINGGLPGVDICPLNLGSKERQLRNT
DLLLTYYLVAGGYTLSMLILIVEIFSGSVHPKKTTMNEIKEEIDEKPIPELPPPPTYQALFGEPY
KNGKLTVFNGREYWVVKSTGGRRKLIPARSPSAVLYHYKF
>AplaIR93a
MSGYGFVFLLLLVISVSGDNFPSLLDVNATLAIIIDREYMDEKYDEVKDAIVEHIATVQREIL
KHGGVNVQYYSWTTINIKDITAVLSIASCDDTWKIFELAYRENALHMAITESDCPRLPSDRA
VVVPLVDNGREVPQMILDLRVVKSYKWQSITMIYDRSLGRDLVEKVVKSLTPQSTSKFQDN
GASVSLVSLFSNTSLLNIRPYLQNVLSTIPVKSSGMNFLVVIKRDYVEIMMEMARRLKLTHP
RNQWLYVISDQLRSNISSVKTLFSEGDNISFIYNTTKSGSSCKEGLLCHVKEMINGFVKSLDL
AVMDEAELFSQVSDEEWEAIRQTKADRSDFLLRGLEVSHLRHYGECGNCTAWKIEAGETW
GVEYTGSYALDYKLIDVGVWSPSKGAILTDVLFPHVEHGFRRKNLPVVSFHNPPWQILTLN
DTGAVLAYEGLVFDILEEIANNLNFTFTVQVPSELVLKNLSAISRNSSDGHNHRRELSNVLTN
VLPRPIVKLIKSKSVIMAACGFTIKQEHKRVVNFTIPINIQSYTFLVARPKELSRALLFMSPFTE
NTWLCLGLAIISMGPVLFAVNRLSPVYEYKGIEMRGGLYSIQNCVWYMYGALLQQGGMHL
PFADSARIVVGTWWIVVLIVATTYCGSLVAFLTFPKMDIPMNSIDDVIARKDTITWSVVRDSY
MDEQLKASYENNYKILSRGMKYSRYDHNMVVDVAKGKHVHFDWQTRLHFHMQNYFEIT
GRCDLTLGTEVYFEEQIGIAVPPGTPYLSMLNFQIKRLHQMGLIEKWFKSYLPKKNRCFKTR
RFTEVTNHTVSLDDMQGSFFVLLLGFTLACFWLFGEKLWHRRKRTTQLSTTTPQYLS
>AplaIR100a
MKLSVKFIILIIYTEYITLHCLTNDDFVKDAIPYWLFRNVSDIVIIYKTNNEIEVDQVMEALPD
NKPFMLFDRSDMFNIKLTKRSFFLMIETNPNDATLTFHKIEKNLSVTTPILIVLSSNSCKENKR
LLMKWTLRFKRLIIVMTSKSCSNHYMNMTTYNPFTKTLATFNRTQEQQMVEFLQINNFRRH
KIKTTVFENGPTVVFKKGRYIGEDISLMGLIEEYLNVTIISHEPKEGKTFGSSKYGSIKELLTN
EADMTTVQMFIKIYDTDDVDMTNYLTFDYVCIVVPKAEKMPIWMAFFTNFNVTLWLILILI
PTLLYFGLLVTQRLRAEHFKRNKPTVTDVLFKIIRIFWNVSRLEPAGVFSSRFFTGLTLIYVFFI
YTVFQSRFVTFLTTPKYYPEINTLEDLSNSGLVVSVKSESISDTLNGTTIPNGFINVKLIPTESP
NYKAFLTRQRTVTYYMEICGPNYHIMPNCPRYYALGYMTKKNFYFLEDINRLVARVREFDL
KHKWDVDAIYQDTMKRRNGTIEQKTETVTLVEFTSALVVLVFGLSFSIATFIFELVNFHLLH
WNFF

>DponIR8aJF
MHVLGLFGAVACVLSVRGQQFKIVTLHQPDQAAEVKYFENAFLKVNKDEEIAFLDVLLNE
DESGHYKQICDALSTGFSLILDFAWSGTEVAQDLTSNMSLPYLHVDVSVAPFLVLLDSYLDS
RNSTDVVVVFDKEEYIDQSLYYWLDSVRLRLVMADALNRSTASKIESIRPIPHSFAIVASAKN
MNKLVSQALNEDLMSLSDRWNLVFTDFETGIFDKSLFQNQTPSLMYLKPELCLDLSIQSRCP
SNFVLNEQFLYWLAWGLSRLAKMAAEESLEFPEKEFQCGKTTFSEDTKERLGDMLDSIIAD
NSNVLSLTGRSVKVSVRGNVEKMINGSFQTIAQYTNGKLTPEPGKQIDPIRAFYRIGITHAIP
WSFKVQNPQTGEFYWTGYCADFAQKISEVMNFDYVFVEPATGTFGEKVNGTWDGIVGDL
AVGETDIAITAVIMTADKEEVIDFVAPYYEQTGITIVMRKPVRKTSLFKFMTVLKLEVWLSIV
GALIVTGFMIWFLDKYSPYSARNNRKAYPYPCREFTLKESFWFALTSFTPQGGGEAPKALSG
RTLVAAYWLFVVLMLATFTANLAAFLTVERMQAPVQSLEQLARQSRINYTVVQGSETHMY
FINMKFAEDTLYRMWKELTLNASTDDTRYRVWDYPIREQYGHILLAINDSNPVANASEGFRI
TNEHLDADFAFIHDSSEIKYEISKNCNLTEVGEVFAEKPYAVAVQQGSHLQDDLSKVILDLQK
DRFFEQLQAKYWNDSAKGDCPSTDDNEGITLESLGGVFIATLFGLALAMITLAGEVLYYRR
KGKKLSPKSGRQSPRTCHSIRLD
>DponIR21a
MLLYALVALLAPIHCTVLESTDDIYTSVTKRALQKSHEKPRIQKLSEFFLETDYYHEDPDKSL
IGLLNSIANQYLSECTTVILYDNYTENTELVFLKKFFRTYPLTYVHGSIPSDYHIQIGELVNKN
DKKCVHFIIFIKDVMRCQDVVNKRNERVVVVAKSSQWRVQEYLSSEFSQEIANLLVIVKSD
KYGPQKAETPFIIYTHRLFVDALGSSQPIVIASWSRGKFSSNASLFQTKLNHGFSGHRFIVAT
AHQPPYVIKKQRSDKDEFEYSGIEVKLVELLAKMYNFSTDYKETADIKVLGSGEAVVKAIK
AGNVNLGIGGLYITENRYNAGIFHWHSEDCASFISLASTALPRYRAIMGPFHWTVWLGLIAV
YLAAILLFSYSDKLTLKHLIRNPIEIENMFWYVFGTFTNCFTFSAGTSWTRAERDITKLLVGI
YWLFTIIITACYTGSIIAFVTLPIYPAVIDSIDQLTGKRYQIGMLNKGGWPSWFQNVSDETSER
LLRKVDYVPDVESGLRNVTKAFFWPYALLGSREELQFIVKTNFSLGSKKSMLHISQQCFVPF
KVGIALPHHLVYSEILAGGIQMILQSGLNIKMKNDIEWEMLRSSTGKLLAANSRSGTLTILSR
DDRALTLDDTQGMFLLLAIGFLAGGGVLISEIFGGCFNLCKKIDNSRATSSNSSIPSNPRFHER
QTIRERNRSISLASFQQRHNSIQSEIAFEKAQAEEHHQGGLVECQIHGTIPDQNSQGVVLEET
NADIDYNEQISKLFEQALGEETCGSSHSGHNQKLT
>DponIR25a
MKNNNIVAGGFFSIFLLNVADICGQTTQNINVIFANEEGNFVADKAVTVALNYIKKTSKLGLS
VDLRRVVGNKTDSQNVLDSLCAAYQQMLDDNNPPHLVLDATRAGLASETVKSFTAALGIP
TVSASYGQQGDLRQWRNLQPNEEEYLVQISPPGDIIPEMVRTLVLNQNITNAAILFDDSFVM
DHKYKALLQNVATRHLIDEINEDVNKIPDHLESLVKLDLKNFFVLGSLQTIKNVLEAAEKKS
LFNRMFAWHVLTKDPDDLKASIKNATIIFAKPIVNNLYQDRLRNIQTTYQLSSVTPEIEAAFY
FDVALKGFLAVKEMLLDGSWKKNNVTNYVTCDDYEPKYSPKRFNLNLRSYLQKESSEPPT
YGPFAIESNGMSFMEFSMALSAVYYVRSGASDKSLPLGTWHGGFNNNMTLLTPKDMKNYTA
DVVYKVVTVVQKPFIYRDDTAPKGFKGYCIDLIDEIAKILHFDYEIDAVADGMFGNMDENG
KWNGIIKDLIEKRADIGLGSLSVMAERENVIDFTVPYYDLVGITILMKMPETPTSLFKFLTVL
ENEVWLCILAAYFFTSFLMWVFDRWSPYSYQNNREKYKDDEEKREFNLKECLWFCMTSLT
PQGGGEAPKNLSGRLVAATWWLFGFIIIASYTANLAAFLTVSRLDTPIESLDDLSKQYKIQYA
PVNGSSTMTYFQRMADIEAQFYEIWKDMSLNDSLSDVERAKLAVWDYPVSDKYTKMWQ
AMKEAGLPPDLDTAIERVKKSKSSSEGFAYLGDATDIKYLEITNCDMAIVGEEFSRKPYAIAV
QQGSPLKDQFNTAILQLLNRRELERLKERWWNKNPEKKQCEKADDQADGISIQNIGGVFIVI
FVGIGLACITLAFEYWWYKYRKNTRVTNVAEAPNSRHHKVGGVQKGFPRQFEGESDMKIT
KLYPKTKF
>DponIR40aNJ

RLRITYGGNLVEAVFDIIHDFPETKLAIGFHKTDPEFTTVLLRKLESGHISVDIYNLSTVDSQD
KYFENLFYHKTNYLPLITIFFGSPKLYEHILLQIYDQNCIRRNLIYIFSWGQTPIKKYFLKNIHY
AMRVFAITNPRLDTFRLYYSQATSYRKHQLELINWWNQDKGLFHHPTLPSKKSVYKDFHG
KYLHVPVLHKPPWHFVRYNASPSKSFEVIGGRDHRILHLLSKKLNFRFRYSDPPERSQGSSD
SESGGFNGVLGLLSEREADLFIGDMGITYERATVVEFSFITLADSGAFVTHAPSRLNEALALL
RPFQWQVWPAIGLTCIVVGPFLYALIALPNIWQPRFPVRSHARLFFDCTWFTITILLKQTGKE
PSATHKARAFIMLLSISATYVITDMYSANLTSLLARPGREKAINNLYQLKGVMQSKDFKLFV
EKQSPSYGLLENGSGIYGEIWDEMERRQNRYAVESVEEGVKLVRDNKNVAVMAGRETLYF
DIQRFGPKNFHLSEKLNTAYSAIALQSGCPFIEEINKILMAIFEAGIITKMTENEYENLGKQQK
IQVLEVPEGDNPASDPETKRSIKSSEDDKLKPINLKMLQGVFYLLFIGNIFAGLVLLSEISLSK
FYKNSKRRPKVQLAQHRKICKKVRHYLRCVRRRFVRMYRNFRHDAFTLTLEYLE
>DponIR41a1FIX
MSSLMALTKNIVNTYFLNYHCLVFFSQSPANFDLNLTQVPILAINLDNRDNIKNIFNYAGCQ
GFFLQVNQPVAVFRAIEQQIKVHPDRFNQRKYVFFATENWSKEVFQIFETLEANFVSDLTVIF
ANSFLNESSQVFDIWTHKYTGRTGNNKPFLLDKWFAENASFLHNNNLFPNKLGNQGGRML
KLAVFPYEPYVVIGIDSYSYMGSEMKILETFSQYVNASISPVINQADYWGEIWNNWSGSGL
MGNLVEDKADIGAAALYTWEFAYEYLDLSKPTVRTGITCLVPAPKLSAGWLTPFRVYSLEA
WMALIGTLALSFLALYALNKLQISVKPQLKSKHHINQLKGKLLSKTLMSVSKPFVMQSITN
KEMAQGNLAKYLMGLVFLSTLVLSTTFDSGLATIMTVPRYDNPINTIEELAESGLSWGGTQ
DAWILSINNSLEPNLMKLVARFVAHSEANLRKYSLGDQFAFGVERLPNDNYAIGAYIKEDVI
QNYHLMTQDIYWEMCVVMMRKSSVLLTEVDKFVLRVFEAGLISYWQNEAATQYMDRTV
QTAVRYYSYNKRKDIVKLKMFHVQGAFAILVVGSIISIAFFCAELAYYKLISSAHSPVIHLGD
YVLYTRDHKF
>DponIR41a2
MLHLNILLSTIVQQYLTPKCVILITDGLNPSPNVPLGTNLLNVNVNANLSNIFGNFGCQNFIL
NANDLSKAFGAVEWALSRNQESFNERKYFVINHKNQEVNDFFENEAVNFVANLVVANPDEE
EIEVEFWTHPYSGTSGNNKPVLIDRWFSCNQSFLQGNGIFYDKTRDLQGRVVKAGVFDLWP
YVILGNIRNISGADIQLLQTLINYINATVEFHIVRDLWGKIFSNGSSFGVKGKVFKGHVDIAF
AGIYLAEDVVHNVDYTLPYSRADVTCLVPKPRVAPPWLSPYYSFASDTWMVLLSTYATLDG
VIYVITYYQLYERAKFKNFRRQKPSKVFVTYKTLVIVSQLTVFQTSTDRKYSTLKKLMFPIM
LASIALSIAYTAGYTSIMTKPRYEGTINTVEDFLRSDLKWGGQQAEELIQKWRNGEEGSYKQ
LGERYLSLTPERLRLLSKRYDMAFKVAQTFGLLHFPTYLSNDVLENYRLMKECLFSQYIIAIL
RKNSVLLTVTNTVVSRMKESGIITYWVLRSESTKSATQRKLRQSLDGQYPENLKLLHVLGA
FVVLLIGLFLASILFCIEICKSKEVKKMSKF
>DponIR60a
MPISETYCVSRKRFTCGCCWTAMFLSVFIFNYFIAFSDFVVIQSKGPHHKNMVVKCLEQITK
HAYNFTKINKNYHTDFLLTYLINQDLSMPGAEIQDELLKLLHKSDKYTIEVLQYGRTDFQSY
LSNCPTDAFEMLSHSSDCIRKHVKQMKTTVYLIVMDDVEMMKSKLENIAETSSFNRDALCI
IYYSKIGNKNNFVTGEIISYLYDVMVITKIVIMMPINLTTFETYIFKIDNQSGMRCFSKSAFKI
QKSYQCNQGTLATNGSNIFQTKELKNYHQCYTRVHALPYEPFVISNLSGIETDVINEIGKVL
NISIHIQLYPSAPMDLGEKCDNGNWTGFLAPVYDSWHLGIGNVPPESEYMDDFTFTLDYIR
AKIVYVVPNANLVPSWRTFMVIFNIEIWGICLLAIISFAVAFRVLKTKSDDRSFRTLQKCFAVA
FQILIAHPIEKQPKSDFTRVYFIALAMLSIVLNSVYTSSMIYFLQNPIREHQISTDKEIANHLPL
GSSPKYKEMFNGSSNQFAKVMHDSYITVNESLDTNNYWIAKVGNERNICTLGIEINLNYLL
SKKNRLVTDKFGNDKVFVLEKPLRSQPVGIIMRKGNFFLDNFNSVIQKLIIEAGIIEKIKSKYL
QSTVHSMGDHEMSENNNNMHSSSNLGQSALSTHHLEGAFAILALGEVCGFLVFAFEIVYCR
VLAKMNT

>DponIR68aJOI
MPGWVLFSILLLYENPVLISASGDSTIKESVSASVRDVQGLQLLVQEILLKTFESGKCILILAD
SMYRPLFQGPWFRRYKGSASFIMIHTDDAEDLLAPCKATQASLDLARNNSCQLYIILVANGQ
QVGRLLKFGDRYRLLNTRANYLMLFDSRLFSEELLFLWKRIINVVFIRQFSGKAQKNDWFE
LSTVPFPIAFRDVLAPRRLDVWNRSQFRKGSVLFKDKTWDLKNETLRVAVFAHVPGTLKAN
TSSLRTIDIGPAFAGSEIEILITIAKQMNFRCELYEPEGAGVELWGRKHVRPTYTGLLSEMSTS
RADIALGDLYYIPFVLGIMDLSIPYNTECLTFLTPEALTDISWKTLLLPFSPIMWACVLLCLLL
TSATFYILARFHRGIDGKKQLTSNRAIKLQLHNAPEDLKYSLLMRQYTRTKQAGEPEGLFQF
AETGNSLLYTFSMLLLVSLPKLPTGWSLRLLTGWYWLYCLLVVVSYRASMTAILSRPAPKV
TIDSLPELLASHLTLGGWGEINSEFFKSSSDDLINTIRERFEIVNSSDEALNRVIEGHLAFYEN
AYFLKHIVWLHRGKTAKNSNSLRHLHIMRDCIINMPVSIGLQKNSPIKPRVDQLLRRMLEAG
LVKKWLYDVMKGAVIPDSDTDGTKALMNLKKMYGALVALAIGYGAGLLALLGELLHFNF
TVKKNWGLTSLIRMGR
>DponIR75a
MSWTILCFLKMFSVCLSSSPAHADTDISFLFDFLQQTNRPNYAVLENVCWSKEETQKLHRNL
TMQNFKCKSVDGNSTIMTERYYEHSYIILVHVDCDFDTIWKQAAKGSLTFYPHIWILLGDFE
KIKKKNVHIPMNSLLLSLVRTANYTKLETAYKIKKTVDWYIERTVGNWSPKVAKFDLEKVN
IFKDRGNFMKVPLRVTYIITDNSTVNHFIDYRNKHVDNLTKINYVLYILIFDILNATQIRLFSR
GWGFESRENNGSFASGMFQDLANDRSDIAGTLAFTPSSRLKYFRYIYPPAKDMDICLVFRAP
SLAYYTNVFGLPFNNWVWVALGLQLLCGCVLIFIIFKWEWKVAQGRKENGPSFLDTAMMQ
IAIACQQDFFHEPKSISGKMATLSILIFFTFIYTAFSAKIVLFLQLSTNKINDVGSVYAAGFDFA
VEDQPFNQYYFKGPSDRAEEQLRKQIYEKKIRRSHGDQFVSAEKGIQLVRDTFCAFHVERT
VAHYLVDKTFSNNQKCSLRFVRSIFKSDMPYLSIPWNSSYIKYFIISFRRLAETGLQDRECKR
CYAKKPSCEGKANSFVSVGLIEAYFPLLIFGVGISLCISILILEKLVHKYIKLH
>DponIR76b
MGLMEVVLTTLATLCFNSTCVDQDLINASKQRLAHLKEELKHETLTVTTLKNGPLSGYEIV
NNTVIGTGVAFEILNIVQREYGFKYNVIVPDHDSFEPVNGGEGGVRNMLLNETIDVAVAFLP
QQYTDVVSYSRSLDTAQWVVLMKRPKESASGSGLLAPFTATVWSLIIISLLGVGPILWLTILL
RARMCKEDHDIVFSLPSCMWFVYGALLKQGSTLNPRTDSSRILFSTWWIFITILTAFYTANLT
AFLTLSKFTLPISEPKDISRKHNKWITNRGNGIVEQLYLSKKYANGDGNSLFEEIGMPQWEP
DVDEDTMLSTYVIKQNMMYIREKTVLESIMYEDYKVKTKADVEESKRCTYVITKFAVCVF
PRAFAFRPGFKYKELFDFTIQHLSESGITDFQQRKSLPDTTICPLDLGSKERRLRNSDLAMTY
MIVGGGLIISTIIFAVELIIYYAKMHCFNKKSHVNNNNTLVTQSNNGLFVKNHQHQGNFRAS
KQFVSPPPSYHTLFHPPNLTNGEYKNKTINGRQYWVFNDKQGMTSLIPQRTPSALLFQFTN
>DponIR93a
MWVRLVICLGVFCKVTNSDIFPSLLTTNASIAIVIDRNYVVEEYEPIKSKIEDYLVYAKREILK
HGGVNTHLFAWSAINLKRDLTFLLSITSCTETWKLFESADTESLLHIAISEQDCPRLPQHSAIT
IPIIDRGQDTPQLLLDLRTVGIYKWKQVVIIYDNTITNDLLTRVIKSMTKQVNRIDASGVSLV
QLAKKVSTTRDVIIANLRTELSKIDPRIMGNNFLVIVSYELAATIMECAKELNMVNTQTQWL
YVISDTNSSTKSMNRFKTFLNEGDNIAFIYNTTDVKNVCLGGTICHTEESITGLMKALDSAI
MEEFQMASQISEEEWEAIRPTKNERRKYLLEKIQKYLSDYGTCDNCTKWTIEAGETWGRE
YQMLDEATNAELLAVGTWRPSDGPNMIDALFPHVAHGFRRKLLPLVTFHNPPWQILKTNST
GDVVEYGGIVFNIIKELSKNLNFTFNVATVKPQSLLNASTLQSPKGDTDSSANFNGNSYITTY
RVPHSILEMVHNKSAALGACAFTVTEENQRVINFTDPISIQAYTFLAARPRELSRALLFISPFR
GDTWLCLSATIISMGPVLFYIHKLSPVYEYKGVRCKGGLATIQNCIWYMYGALLQQGGMH
LPYADSARIIVGSWWLVVLVIGTTYCGNLVAYLTFPKIEVPMTTIDDVLAHKEMVSWSYAKN
TLFEARLHNSVDKSFNIIFKDAKNIWDRKAMMGEIKSGKHVYIDWKIKLQYMIKEHFIDSG

ECSFALGVEEFCEEQIALIVAPDTPYLHKINEEIKKLHQVGLIQKWLSDYLPKKDKCWKKKR
TIEVNNHTVNLDDMQGSFFVLFIGFLIAVIVISLEMLWSRKVTNNRKRKVVHQFVT
>DponIR100a
MPQRAKYFGVYTKVTTMIQATDFVFFLVFPSVIFNRLITRDQQECSYFHNYKMYMIHFNLS
RDVKITQMVCDNSSDYNTVDLVGEFFEMNQVSLKKATLLENQIVTYDRNEISGASFSKYGI
VYDAVRNIKIIQKDVSSMYKRMAYIDSYLVRIFIGVDPEMFFKYFADMDVSTFTKTARSLNV
LLFSTRDVDMPMVHQLLVFLWERFSILNIVVQFPCSADYREYVSTYRPFMKTKSEYGQVHI
YHYEQVAKYPQLLLNDVTDMKGYPLKVSLFERHPTATRFVPKFIRECKIYKTIPYTSKFYGC
DGEAMATLSAYMNFTIHLHDGSASYGRLQADGTLTGSFKDIVERTIDLQGNSRFMMPYGVE
GYEFTYIYHFDQLCVIVPKAKKLSNWLATLKILTGRFVLVSVGIVAICGIANKFFRHEACLLS
EAVLELYSSFLGQSVAEIVGTQNRLSRRIFIASFLLYSIVISTAFSAALLSVYTTETYLPDINTLE
EFDKIGMTIRSSINPFKDGESALYQSIAKKVRSKYQKENHLSSIEIAAASNVGGIERYLDSKLL
IQTHFSDKDSKPLLHIVRDCPNSYFLSYIVPVGSPYLKIINHFLVLINEAGLKSKWLEDFSDAF
VYQRRIEKLKQQASGSAEVYQAFDLDNIKGILIILLIGYCIAFIVFISEVCSMPLPECI
>DponIR100b
MSRSASKLFLLFNLCFLGQSKMRVPNVQEDFLPKPFRNFYRYCSSRAGEVSIIRLEYRGKTD
LQTGEIVNSFYRNKSNILCKNYLVQLKPMELTSSASKIEEFNKLHMVETRELMELKPDFYNC
DSSSIQVIIAKDASIVYDFIKSHKSQTPFETVRGLKILIFPNSNSDIKSIKAILAWLWTNCGVLN
VIVLLPSSVKYRNFYITYKPFTATAFGHGQIQIQHLKGLRINTPGRINTKIPDLQGYPLIVSFFE
RISTAFRTVPNFLVNSKIYRHFTRIHGVDGLTIEYLAQTMNFSMKLCNEIECQYFGLVLDNKT
VIGSLGKVVERKVDFQANSRFLMDYGTDDIEFTTSYMHDKICVLVPKAKLIPTWIKVLRIFK
LDTKIVFLLAFIAAAITSKLTETTMSLADSVLEIYILMLQQPVDMDVCNKHVARRCFMGSSII
FFMIVSNIFIGGLFAVFGVKAYYSDINTLEEVDASGMDIKTSVDPFKGSSLPLYKRLSQKLKM
ELKSKNTIEFVLSGHGASLERSEDTQFRIIYQYVTHDGTPLLHMVPDCPTEFNLAYIVPKGSP
HLPAINHILSIFLESGLTLKWYVDVLEAIRLNAHWNIHEKQRSRKALSLNDVQAMSWILIWG
FGFALLAFLLELSIHKISNA
>DponIR100c
MLPFSHILVCSWSGTNKPNHFLNFRQFIQSKTSKMQVNVLKFLFVVNQFDFSSEKLQNEDP
DNCLVFDSRKVFTNERFPPTFMHVTRLRYPEESTYESGFHDLHRSFQRPPPYNLFEITDQEVA
SQSVFIQNEIQDVEVVNITLNRKWHRHEQTSVYDKHVVQIFIGRDFRVFFDYFIPANLIKVSR
LARTWHVFYFTSKQTNMVKVAKFLFYIWKQFGILNVIAQIPCSPKYKEAVAVYKPFMVNSK
QQYGQVEIYSIASALKNPYTLSNTASKLNGYPLKVSIFKRYPTAIKHLPYVLHDYKIYKNIGK
TSTFYGLDGVALSEISAALNFEIKIAIGPENQYYGYVSSNGSIIGSLGQTVQKVIDLQANARY
FMNNDILSVEYTSFFMFDYMCILVSKSETIPKWLEIVYVFLTKANIVAMVAWMVCCCFNVM
TRTSIVVSKQEAIMEMYCASIGQPQKALIVFHPKMSRRIFITTCLIFSMISIPLLTAKLFNTLTS
VNSSPDINSLEELLQSGLYIKSSINPFSSKFNAVYEKLSQRVDTNWLNTTAIELVAQSKQWAA
LERLKDAKLQIASRYIDGKGIPLLHIIPEYVAKHYMGYIVPAGSPYLLLINKVVTRFSEAGLL
EKWYKDFEDAFVAEARMHKEGQLLEDSRYKPFKMIDLQSVFYMLCCGYLTSSVMFLVEIIF
SAKNPRIDKLLNI
>DponIR101FIX
MGSTPAHMSGSGAFKHPFSGRTGRRDRPGAPLKLTRRGKIADPPTLVNSGQSIICPQGLVQS
VPNQRVAEMLAFLLLASVQAQLEALVGAIFAPCSSLCYLRAPARGLDGLLSHIRRPVYVQPL
SQLSAHPLANATLHCDCFLLDTADLSLVARHLESRRNNYLIRPHSTLWIIQPQLEAAAPPQMF
QPYALNVLQLQLAPNRSEAQIYSAWRGRVVPARQLDAGPWRARDLIAFRKRPLSISLFDCK
PFMYLADGQARGVEVQWVNELLSGIAQRHRPVPLSENPWKDSLSSVQARESDLAGCSQYA
LNLLKWNVDAILSPLQACYTFLVPKAGYQPEVTFLAQAFSWPVWAGLLLSLVGLGLLGVA
LGVWRSVQDALIHVLQACAAGSAYSARCRPLRTSKRLFLIGALFYGLLFDSYYTAGCSSILT

QPRIVRVIDSFADMVHFNVGYQLQSSTIQSDFWALNSSTFDGLAALYRRADRRQTRLDGRS
ALTVKSIGDAYITDLAGFPREQLRHYRVLKQCISNNYIGLALMKNSPLTRVFGAVSQRLTEA
GLIPKWLRDEIQAERESQREFFKAYESEMGYGMVTSGKLAGALAVLLLGHLAAAGSFALEL
FVGSKQQ
>DponIR102
MAVSCCLLLFGLLLHGADGWNISASEPLPSELFKRLFSLLFPNASSLCYIFQSDSLQLAHFLS
LPVTMVQYQNASILLKMERSCQGFVLSVKDVGVVQRYLRLRTVRYGVLKPHRRIAVVYER
AAQIHLQELLGLYAMDVVEVEMPGDALRLRRLSANRVIFQWNLTETEFFLDEAGFAQEEWE
PTAFARQFNYTFQFAVFGCEPFILAQNKRIRGGPEINLALEMTRGLPLRFKDQKPFFSAENPW
AEALNLVKRNEVDMAGCSQYVANQWRHSIDFTVNQNQICQTFLVPKARPSSDVHFLIEAFS
GRVWLCLLLGAVGFGAVGRLLLVAWPAGGGASAWITPMLDVLRLYSAGGVFVTGGNLASC
PARIYFLFLLFHGFLISTYYSAGLSSTLTVPKLIRQINTLQDLVDYGITFQEQSGIVAAFDLVNS
SLFRGLARLHSNQPRSSTPLDGLTAMTVKTLDNSYVTDLDGFEPDKLKDYKALKECIGNHY
MGFALQRESPFKRRFDTVAMRIMESGLLRKWLHEQIYQKSALQAQFFSSYAQNAHYRTITA
RRLYGAFVLLLVGYGSGALALLVEMVFNKNV
>DponIR103
MRCSAVQSNPDHSRNVMYLVIIFIACKFATVFGKISPVSEARHEELLVDLIRMVLGKAENVC
YFEDDRYGGVLPRAFATKTSNSFVILAQSKCDFYILHVKLENLQSALKHLIPHTKVIVINSKR
NDDSSQWTLDSKLLYERGLFPLAVSSPEGSKVVQVTHLQTNTTLQVHRGENFSFPLVTWSP
KDFLRFTGRNIVITTFHCPPFVETTENGLEGLEQKIIHELVQDWPIEYKIIPDEKGVLMNKFLL
AIESVQTEQSDIAFCFLWQRVLMERNVDFSTAMFPTCVTFLVHKPKLLQSYTFLFQAFQDIN
TFITCLLTIAVLEIIYKVFITDLWQAKALFERSAGSHLKLQLTSSRICLVIIVTFHFLFFSYYSAK
LTVLSSFPRFSQNYIRSFTDMVEQQTQWVEPKNDIQKWLKKTNDSICMGIAQNFRIGDNRTE
INKKLKSGQYGFLVKRFAQDFVSGSEELDDYAKIYLRPIPGCLATFYSSIAFQKNSPFPLYLN
RKISSMLESGIVNYWRQLTLRKPAYGYLKSFKSLYVDQMVTSKFDIDKLSGILYFLVFGFGLS
IVCFVNELFNGRKKPVKCSSIKRVKT
>TcasIR40a
MRRDHGGDLVSASFDIVAGFLFEEICICFDKNTNINFLQHLLVRFVSNNIAIKLFNITTVEVQD
KYFAFLNYQVTNHLGANTIFFSSHKFYEHVLLEINERDFIRRNLIYIFNWGRRPFSRYFVRNII
NVMKVFVITNPRNDTFRIFYNQAVPYKKHHLEMVNWWQHGVGLFNHPTLPAKYNNVFKD
FKENVFKIPVIHKPPWHFVQYGNDSIKVTGGRDDRILSLLSKKLNFRYDYFDPPERIQGSSAS
ENGTFKGVLGLIWKRQAEFFIGDVALSHERANYVEFSFITLADSGAFITHAPSKLNEALALL
RPFQWQVWPAIGVTFVVVGPVLYAIIALPNAWRPRFRVRSHARLFFDCTWFTTTVLLKQTG
KEPSSSHKARFFIIILSISSTYVINDMYSANLTSLLAKPGREKAINNLNQLEKAMATRGYDLY
VERHSSSYSLFENGTGIYSRLWQMMNRRQTHFLLESVEEGVQLVRDSTNKAVIAGRETLFF
DIQRFGASNFHLSEKLNTAYSAIALQLGCPYIEEINKILMAIFEAGIITKMTENEYEQLGKKK
QTTSETEKELIPGVKKENRRVAKVSEDNEKLQPISIKMLQGTFYLLCIGNIFSGFILLAEILVY
KHRKTYKHKKRRHRFVYLRKIRHSVASKFGAVVDAVRRVYRRAMHDAFVATLEYLE
>TcasIR21a
MQRGLIVLKLCLTALALKSLDKRALQKSHEKSQLEKWEDKFLNRDPSFDQTASLVNLISKV
ALDELSGCSATILYDKFTETSSDLLLEKLFRTFPIPYLHGQITDKYHMKVPKLQTSQDTCTGY
ILFLKDVMRSKDVVGPQTNNKVVLVSRSSQWRVYEFLASEQSQSFMNLLVIAKSEKIVSSSI
ARLICLALHLKFGTALAIYAPNGGKSAVYPSVIANVPKLGFRSAESVTSVITQNGANLGIGGL
YITDTRLKATDMSHIHSQDCAAFISLASTALPRYRAIMGPFHWTVWLSLTLVYLFAIFPLAFS
DKHTLRHLLDKPEEVENMFWYVFGTFTNAFSFFGKDSWSKTDKFATRLLIGFYWIFTIIVTA
CYTGSIIAFVTLPVFPATVDTPEQLVRGKYTVGTLDKGGWQYWFENSTDPITQKLLTRIDFV
PDIESGLKNTTKAFFWPYAFLGSRAQLDYIVRTNFTTINKRSLLHISSECFVPFGVSIIYNKNA

LYSKIIDQGVLQAVQSGIVDKIKNDVEWETMRSASGKLLAANSYGKSLKALTVDDRALTLD
DTQGMFLLLGIGFLLGGASLLSEWMGGCLHLCKGNRNQSATSIQSNYRSHEVPTPREKLDS
MQFNSFENHKIEEEIVEERNCIIHRQDDDDIEEHINRLFDFEGVFGEANPDSRTGPEEELSFKN
TTKAFFSLYAFLDSRAQLDYIVRTYFTSMNKRSLLHISSECFVPFGVSIIYNKNALYSKIIDQG
VLQAVQSGIVDKIKNDVEWETMRSASGKLLAANSYGKSLKALTVDDRALTLDDTQGMFLL
LGIGFLLGGASLLSEWMGGCLHLCKGKRNQSATSIQSNYRSHEVPTPREKLDSMQFNSFEN
HKIEEEIVEERNCIIHRQDDDDIEEHINRLFDFEGVFGEANPDSRTGPEEELSEENGKK
>TcasIR76b
MGLFEIALAALCLNATCPGEEEPPEFPEVQYLAPDSNDRKTLFAQLTEQLKNENLIITTLKND
RLSGTEKRNNTILGKGIAFDLLNILQDKFQFNYTLIEPKANVWGAEKFGVLDLLKDKKANL
SAAFLPVLTQYSNHISYSPSLDTGEWVVLMKRPKESATGSGLLAPFNLPVWLLILLSLVVVG
PVIYFIIYLQAKLCKDDNNKVFPLPACIWFVYGALLKQGTTLNPMTDSSRLLFATWWIFITIL
TAFYTANLTAFLTLSKFTLPITEPKDIGEKRYKWVTTKGNALEDTVTVNESLTELGKILGQPQ
RYLYVSDSDILRNYVHKRNWMFIREKPIVEYVMYDDYKEKTRNQIEEAKRCTYVITKFSVV
SFSRAFAYSKDFKYKPLFDSTLVQIVKCHKCFSLLSRIQYLVESGIIKFKLREELPDTEICPHNL
GNKERQLRNSDLLMTYEIVGGGFIISAIVFIIEVIIRRQKKPKTKSLPLQNPNKHTFEINLNNN
YEKFGHFPYSSKFVTPPPPYHTLFNPPHKSDNMKKRNFNGREYWVYDSISGETKMIPMRTP
SALLFQYTN
>TcasIR64a
NKISLILVILSKTETYIIKSCLSNAIVDFAILANVAFSLRISCYKLFMHIKLIANVFYNQLDQVL
NRNHYHLAVIIDSGCIDYADFAIQDKKYFYETYHWLVPTTPQNLNNSLNFLQKSPLNINSDV
NVAILNGEGTKWSILDVYNPASSHHGQFTVTKLGLCDETNGYQAKIAGNKYWSRKNMTG
VQFKSAVVVPDPSIKLNDYLTSDKNRQLHSMHRFQSVTVNYCREMYNFSLEIQRTNSWGYL
TPNGHFDGLVGLLERRLVDFGSSPLIYKLDRMPVIDYSYGNWVLRSTFIYRRPKIIEASYKIF
LRPLSRTVWICIVLMMVLLMLFLKVVFSREKRLLQKRNLVDSSWSFLFLFTLGAFCQQGAT
CHPQLLSSRTLSIFVFLFCILTYQFYSASIVSYLLIDPPRKINNLKDLSDSNLRAGIEDILIDRNY
FVQTTDPVAIELFNKKIKFSNNNSGFYEPWDGLDLVKQGGFAFHVETSTAYPIIEETFTNEEIC
ELEEVQMYRTQPMHTNLQKNSPFREMMNYCMLHLVENGLMYRLRKYWDARKPMCIESA
KKFTFNVGLKEFSSGLIVLSYGILISLGLLLREVIVHKK
>TcasIR75q
SFLGTILTVYKQLAEKKIVLNVLTNHWKINQTKLSQHTFLVGDTLCPQFNSLLSHVSKFFCY
QNSQQTLGQIITSSXKWLVFDQNSTVNTNDLLLDSNFAVASQISNGRFHLKLCYKRAPNETI
KFNEIGVFSNGFEYYNHFIPTRNRSDLSGVNITVSYVVTKPDYPFDVEDYRFRHLEAFSKLS
YAMVYPMLEMLNCTKKFIQRSSWGYKGANETQFVGGMFGDIQNGTAEIGGTVSFYTVDR
MSVVDYLSVTTPSDLKFILRAPPLSYVNNLFTLPFDTKVWYCLYFIVGVTVLILYVIVRCEST
YENALERRNNIDNIKPKFFDVVMLQIEAITQQGSENEPKTMSGRIAVFIVFLVLMFLYTSYSA
NIVVLLQSTSANINTLQDLLNSKITLGVEDVVYSHHYFETQTEFTRKSIYEKKVAPKNQKSN
FMTTEMGIEKMKDEFFAFHVETTAGYKQIMDTFQEHEKCGLIEIDYLNVLYPSITIRKNSPY
KEIVKVNFRKIYESGIRHRQLNRIYYKKPHCVGKGGSFKSVGIVDIYFSVEIFAIGCFMALWL
LLLEVLFKKKIKFLVQ
>TcasIR75q2
MKILIVFICLLINETTQNNFTDNLIVNTFNFIKILNVPVKISAHICWTRGKFDSLLMKLYXTVL
ANTIHFIKSISDKYNTNLIKNVSPKYANPEHQLFIIDLKCNDSLSVLQQAEKFKLFKSPFKWL
LLGNSESLPNLYFGTDSQIFVTEPRSQLDDIKTIYKYSPMVPRFVQHSFDRFYTNTKRTNLM
GTTIKISYVITNLDSLNHLWDYRLQELKKKLYHFLICRNSHIDAINKLNYILVHNLMDFLNAS
RQFTMQPTWGYKNSTTGLYSGMAGDLQKGLADLGGTPLFFTPDRIDIIDYIAATTPTYMKFI
FRAPPLSYVTNVFTLPFDSAVWHYCFVMVAVVVVCIYVIVVWEWKETKFEEKDTHSHIDTL

RPNIFDVVMFEIGAITQQGTNAEPKSNSGRIITIFSFLTLMFLYTSYSANIVALLQSTSDSIKNL
EDLLNSRIKLGVEDIVYAHYYFENAQEPVRKAIYQQKVAPKGQKPNFMTAEEGIRKVQQGF
FAFHVELSTGYKIIGEVFQEGEKCGLKEIEYVNLIEPWLATQKKSPYKEVMKIGMRKMHET
GVQNREIRKIYTRKPQCHSGGSNFGSVGLIDCYSAFLTFGVGIAFAFLLFVMELIVRRYFIRR
EKERLK
>TcasIR8a
MVISENLDKTTANRLKAIRPIPNNFAIVATSSNMEELLQTALDENLVTLPERWNLVFLDFQYQ
QFDKKRLKNMPINLLHMDEEICCRFLQSEKCECPHDFNLQENFLSLATNTLAKILKTLTMEN
LLRADLNCDDSRYSEATRTRFYELLQQEVDSNDLVFKENFGLHVNINGVIETGDEKVAEYN
YKTGVTVLDGKKVEPITPFFRIGITHALPWSYKETDSSGNTYWTGYCVDFTEELSKLMGFG
YEFVEPKSGTFGKKRDGVWDGVVGDLATGETDLAITALIMTADREEVIDYVAPYFEQTGITI
VMRKPVRKTSLFKFMTVLKLEVWLSIVGALIVTGFMVWFLDKYSPYSARNNKKAYPYPTR
EFTLKESFWFALTSFTPQGGGEAPKALSGRTLVAAYWLFVVLMLATFTANLAAFLTVERMQ
TPVQSLEQLAKQSRINYTVVKDSDTHKYFINMKHAEDTLYRMWKELTLNASTDDTQYRV
WDYPIREQYGHILLAINDSNPVANASEGFRIVNEHTDADFAFIHDSSEIKYEISKNCNLTEVG
EVFAERPYAVAVQQGSHLQDEISKTILNLQKDRFFEQLQAKYWNHSGKGSCPTTDDNEGITL
ESLGGVFIATLFGLALAMITLVGEVLYYRRKSKIQNSETKKPKTVQTSENWKTDTLMPVSLI
NKDKQSVTIGTEFKPVNRNRDLSEFGHITLYPRARNRITQTSNE
>TcasIR25a
MASSSAIIYRIAIYSRIATAHLNYSDFLNNVLTETHKMLKLVAFILYCTNLANGQTTQNINVLF
VNEEGNLVAEKAVDVATNYIKKNNKLGVNADPVKVVGNRTDASGLLDSLCSSYNEMIANS
MNPHLVLDTTMTGLASETVKSFTAALGLPTISASFGQEGDLRQWRNIDENEKEYLVQISPPA
DVIPEIIRSLVLSKNVTNAAILFDDSFVMDHKYKSLLQNVATRHVIAPIKEADKIGDQLRQLR
KLDIVNFFILGSFENIKRVLDAADSVGFFNRKFSWHAITQDKGELKCNCRNATITLAKPLIDA
QYQDRLGLIKTSYQLNAEPEIAAAFYFDLALYSFLAVKEMIADGVWKRNNATNYITCDDFD
GKNTPRRAGLNLKKYFSKEVSETPTYGPISIVSNGYSFMEFTMQISAVGVRESSSDKSVPLGS
WKAGYDNNLTLVDPQIMKNYTADVVYRVVTVEQKPFIIKDETAPKGYKGYCIDLIQRISEIL
NFDYEITPVGDQKFGNMDENGKWNGVVRELMEKRADIGLGSMSVMAERENVIDFTVPYY
DLVGITILMKLPKTPTSLFKFLTVLENEVWLCILAAYFFTSFLMWVFDRWSPYSYQNNREKY
KDDEEKREFNLKECLWFCMTSLTPQGGGEAPKNLSGRLVAATWWLFGFIIIASYTANLAAFL
TVSRLDTPIESLDDLSKQYKIQYAPLNGSSTMTYFERMANIEAKFYEIWKDMSLNDSLSEVE
RAKLAVWDYPVSDKYTKMWQAMKEAGLPNTLDEAVKRVKDSRSSSEGFAYLGDATDIRY
LEITSCDLQMVGEEFSRKPYAIAVQQGSPLKDQFNTAILQLLNRRELERLKEKWWSKNPEA
KKCDKQEDQSDGISIQNIGGVFIVIFVGIGLACITLAFEYWWYKYRKGGKVVDVQAKHSDV
ATKINDGFHAKINKLYPRSRF
>TcasIR68a
MIKNLLPYKCVVLISDDIYGGTFTKSWYRRFGPFITFVVIRVDEYEDLLSPFEETQACLDTAK
NEGCQMYLILLSNALQVSRLLRFGDKYRVINTRAKFVLLYDNRLFDKPLFYLWKRIINVIFIR
RYSGQKSDTKKNMPWYEITTVPFPTQITSILIPRRLDIWTKSKFRKGIDLFRDKTSDLRNQTL
KVAAFSHIPGTTKSLQEKTARTVIGNFSGTEVEILQTVSAAMNFHCELYEPVNVDVDLWGG
KQSSGKYTGLVGEMVSTNADIALGDLYYTPYILDLMDLSIPYNTECLTFLTPESLTDNSWKT
LILPFKYFRPAMWAAVLVCLLICGAVFHALARFHETISQNKSQVLEIHTKRKKIIILSICPEIEK
LDSNLKYTKMREQYKPPRFEGQSIGLYQFSEPFNSVLYTYSMLLLVSLPKLPTGWSLRMLTG
WYWLYCLLLVVAYRASMTAILARPTPRVTIDTLQELVNSRLKCGGWGEINRQFFKSSLDPIT
KLIGENFELVNDSNEAVDRVAQGVFAFYENSYYLKEALVKRQLRFQIARTTQNQSEREMRDI
AREDRNLHIMTDCVIKMPISIGLQKNSPIKPRVDKYIRRVLEAGLIKKWLQDVMASILNAEV

QSTQEEMKAIMNMKKFFGAIVALFIGYFISVVVLIVENVYFHFFVKRNPHY
NKYTRSIHHVKKAE
>TcasIR100l
MPRKLFLWIFFLLVSCYGNLSETHLQFLKRYFVSANSVAISMLQTHHQEVKIRDLAEVISRK
LNSIGTPVVVHENHKSGSLNIIMIVWSLKILRQFLDSLVVPEEKGTYYIIILEQDCATVHSDFA
QILEQFWCEHNVLNVVVQNPCSGGTFYLFLPFEHRDNFWGSCKSWDFNEQMPNKLRNLN
QFPLKISLFLYNPTLIAKLPKGLKTNPRYHNLSASKGYGGLDGFLLRELVDYFNFDPVIVENL
EEYGRVLPNGTAFGSLGDVVNQRVHFSINSRFLMDYGTKEIEYTFPYISDEICMLVPKSLKVP
TWKTLLKCFNTLSWVLIFVSCLCSTFAWYFVGPSKNLHKLIWQIYCFIVGIPQKIEPSFSQFV
FLLSCFFFNVTIFGIIQGSYFTEFATTSFYPDIDTLEELYESNLPVATHFWFLLDGDTSDLMTKL
KTHKIEATGDCLEQTARQRNIATLGRKSESDLIIRTKYTSRDGTPLVHIVEECHTSLYLCGIVP
KGSHFLAPFNQIITRLFEGGFTTKWYRDVFDGIISEEKPQLDETVSFNSLNMNDLQTAFHILTI
GHLFSIMVLIGEVVIKGKHNKKLLT
>TcasIR100
KVTIIILIMMCLSLPKIQTCPIKINHLKEHFKQVKSARIMILQNEIIVTDWLIMELIKDNKITVT
VQKAIRNFEPFNTSNLTRFEALEFNDTIPTLQTDSTCGHLIIVKNEERLYQYLKSDPGFLILNP
RHFYAIVAMELFKTNVLREFWSLQVSNILLLDCDTSYTVLPFNGTTIRINAYTQRKLLRNFH
NYFLQVSMQPKPPTAIVKFPKPLRENPIYKDLVPFKDYAGLDGCLLKVLTQRLNMKYVIVG
NGQKYGTVLKNGTTTGTLAWIASNKVQISTNGRFLMTYGTNKLEFTVPYSSDQVCAVVPK
ALKIPKIIMLAKSLTPSSWFMIFLIYVICVLIYTLMGSTGSTWTLYAIFHGFPVKIVPTSRQSFF
LTSCMLFSIIIMTIIEGSFFKTFTTTTYYKDINTLEELDESELPIAETFFSFTNDKSRIMTSLKRK
KLVINRDDILEQVARKRNIAKLERKRDIKVRLKTEFLDEEGESRLHVVEECFTTFYIGFIVPK
NSIFLPTFNNVIRRIFESGLTQKWYGDVEFSIFLEKIFKLENNIKHHSFSFDNIVSALCVLFIGL
SLALLVFFWEVTKXKQITLIYVSLIYCIISRH

>DmelIR7c
MLHSAVHNVSLVYALVWAIDNYYGMATSTPLAVVQFPTSRESRRLHNDLIDAALGRSSGTG
RIQFLLEDD
RVEMTETDTDPPPPSGLTGRPIAIWFLDSLRSYFRLEMYLNQLGSPYKRNGFFLVIYTGLEDQ
PMESLKI
MFRRLLNMYVLNVNVFLQRDGTVHLYTYYPYGPHHCQSSLPVYYTAFQDLAAPANGFGLT
KPLFPRKLTN
MHGCEMVVATFEHRPYVIIEDDPKTPGGRSIHGIEGLIFRSLAERMNFTIKLVEQKDKNRGEI
LPDGNFT
GILKMMVDGEVNLTFVCFMYSKARSDLMLPSTSYTSFPIVLVVPSGGSISPMGRLTRPFRYII
WSCILVS
LIFGFVLICLLKITALPGLRNLVLGRRNRLPFMGMWASLLGGLALYNPQRNFARYILVMWLL
QTLILRAA
YTGQLYLLLQDVEMRSPIKSLSEVLAKDYEFRILPALRTIFKDSMPTTNFHAVLSLEESLYRL
RDEDDPG
ITVALLQPTVNQFDFRSGPNKRHLTVLPDPLMTAPLTFYMRPHSYFKRRIDRLIMAMMSSGI
VARYRKMY
MDRIKRVSKRRNLEPKPLSIWRLSGIFVCCAGLYLVALIVFILEILTTNHRRLRRAFNVINRYA
A

>DmelIR67a

MLPILVPVLLLFNETSWINPILTSIYKDRHHETVLLLQHSQHGNASGLERFPWPVFSFNEQM
DFYVRGKY
NSEMLVLIWQTGNSDWDLDLWQALDRSLLNMRKVRVLLLRKWEKIPTADVAATAEHLLFL
HVAVIGQGNR
IYRLQPYAPQSWLQVDPIESPIFIKIRNYFGRYIVTLPDQFPPRSIVYRNPKTDEIQMTGYVYK
FLLEFI
RIYNFTFRWQRPIVQGERMNLILLRNMTLNGTINLAISLCGFETPSELGVFSDVYDMEEWYI
MVPRAQEI
SIADVYVVMVSGNFLIVLIIFYFIFTILDTCFGPLLLKERVDWSNLMLNERMISGIMGQSFNM
SARNTIS
SKVTNATLFLLGLVLSTLYAAHLKTLLTKRPTSQQISNFKQLRDSPVTVFFEEAERFYLKHA
WDRPIRYI
KDQLNFRETIEYNALRMGLNRSNAFSALTSEWMIVAKRQELFKQPIFTVQPELRVIQTSVLL
SLVMQSNS
IYEDHINDLIHRVQSAGIVEYWKHQTLREMITMGMISQKDPFPYVAFREFKVGDLFWIWLL
WVSFLFMSF
VIFLCELLVDCFISKTLIRNKRPH

>DmelIR94c
MSKVFKLLVLPLIYLSLTKGSKNPQLKFLRELINVIEEGREIRTIMVIKHSRDEYCHLDQWNP
RGSPILR
TNEMGSIRISGYFNDQAVILACMGENSDYGLLKSLANAMDNMRQERIILWSEREPTKMLM
DYISQQADRY
NFAQIIIVTMNEDVDAVPSLHQLNPYPTPRFRQITNISNIRRTSFFGCGLSFQGKTAILKESVVS
NIRFK
VWSPSGPIPLSELKDYEIVQFAVKYNLSLKLYDQNESKSDHFDIQLGPLFITKDFPTQMAFVS
PNTACSL
IVIVPCSPKWRFMDVLHKLGVLKLIGCLLIAYAVFVLIETLILWLTHRISGREVRLTSLNQLLN
PRAFRG
ILGLPFPEFRRSSISLRQLFLVISVFGLVYSNFVSCTLSALLTKPAQNPQVRNFKELRDSGLITI
MDKYT
HSFIEKHIDPEFFDHVLPHYLILQKKEALRMIWNFNDSYSYVMYTTTWKSLNTVQKSFDER
VFCESESLT
IAWNLPRMYVLGNNSVLKWMLSRYITYMPQTGIPDSWTEQLPKVLKLLYNVTSPRRIKEGA
VPLSIQHLS
WIWHLLFIGESIATLVFIVEILLQKSNQHTSNMRERSSEDDDFV

>DmelIR10a
MAVLGTVFLLFMLDLKTLNLTRLNGLLVEPTRDLPQLELWLRAGSDHQDAENPYVQWFLL
RTEIPLSIVT
YQENRYWMDDPFGRRNLVLVMSLDQLLTNRGAAAPIQKASTFFYILADQDKDLSADEQLR
LEGSCRQLWT
QHKVYNRFFLTRDGVWIYDPFKRRDSAFGRLVRYYGSETLDKLLFRDMAGYPLRIQMFRS
VYTRPEFDKE
TGLLTRVTGVDFLVAQMLRERLNFTMLLQQPEKKYFGERSANGSYNGAIGSIIKDGLDICLT
GFFVKDYL

VQQYMDFTVAVYDDELCIYVPKASRIPQSILPIFAVGYDIWLGFVLTAFACALIWLTLRVINL
KLRIVSL
GNQHIVGQALGIMVDTWVVWVRLNLSHLPASYAERMFIGTLCLVSVIFGAIFESSLATVYIH
PLYYKDIN
TMQELDESGLKVVYKYSSMADDLFFSETSPLFASLNKKLSWNRDLRADVIDEVARFRNKA
GVSRYTSLIL
ESSHFTLLRKIWVVPECPKYYTISYVMPRDSPWEDAVNALLLRFLNAGLIVKWIQDEKSWV
DIKMRSNIL
EADAESELVRVLTIGDLQLAFYVVIGGNLLAFLGFLAEHFRWKLQKKGV

>DmelNMDA
MAMAEFVFCRPLFGLAIVLLVAPIDAAQRHTASDNPSTYNIGGVLSNSDSEEHFSTTIKHLNF
DQQYVPR
KVTYYDKTIRMDKNPIKTVFNVCDKLIENRVYAVVVSHEQTSGDLSPAAVSYTSGFYSIPVI
GISSRDAA
FSDKNIHVSFLRTVPPYYHQADVWLEMLSHFAYTKVIIIHSSDTDGRAILGRFQTTSQTYYD
DVDVRATV
ELIVEFEPKLESFTEHLIDMKTAQSRVYLMYASTEDAQVIFRDAGEYNMTGEGHVWIVTEQ
ALFSNNTPD
GVLGLQLEHAHSDKGHIRDSVYVLASAIKEMISNETIAEAPKDCGDSAVNWESGKRLFQYL
KSRNITGET
GQVAFDDNGDRIYAGYDVINIREQQKKHVVGKFSYDSMRAKMRMRINDSEIIWPGKQRRK
PEGIMIPTHL
RLLTIEEKPFVYVRRMGDDEFRCEPDERPCPLFNNSDATANEFCCRGYCIDLLIELSKRINFT
YDLALSP
DGQFGHYILRNNTGAMTLRKEWTGLIGELVNERADMIVAPLTINPERAEYIEFSKPFKYQGI
TILEKKPS
RSSTLVSFLQPFSNTLWILVMVSVHVVALVLYLLDRFSPFGRFKLSHSDSNEEKALNLSSAV
WFAWGVLL
NSGIGEGTPRSFSARVLGMVWAGFAMIIVASYTANLAAFLVLERPKTKLSGINDARLRNTME
NLTCATVK
GSSVDMYFRRQVELSNMYRTMEANNYATAEQAIQDVKKGKLMAFIWDSSRLEYEASKDC
ELVTAGELFGR
SGYGIGLQKGSPWTDAVTLAILEFHESGFMEKLDKQWIFHGHVQQNCELFEKTPNTLGLKN
MAGVFILVG
VGIAGGVGLIIIEVIYKKHQVKKQKRLDIARHAADKWRGTIEKRKTIRASLAMQRQYNVGL
NSTHAPGTI
SLAVDKRRYPRLGQRLGPERAWPGDAADVLRIRRPYELGNPGQSPKVMAANQPGMPMPM
LGKTRPQQSVL
PPRYSPGYTSDVSHLVV

>TcasNMDA
MNVVVFLLNVLIVVVVSGGPAWREVQRGGGLRVGSGAGKNSSSSRGNGIRVGGSTRGVRI
TTTMMPEGDQ
VSQPTATATSNHQHTPLNVGMVLPSKSFGVREYTKAVTSAKYNLQRKLRLFKHHDIKVHIV
MKELTPSPT

GNSMAGDHKDSLNVGLILPYTNFGVREYTRAINNAVSGLHRSRGQRLNWLKKYNFTPKNV
HYVLITLTPS
PTAILKSLCKEFLSVNVSAILYLMNYEKYGRSTASAQYFLQLAGYLGIPVIAWNADNSGLER
RASQSSLQ
LQLAPSLEHQTAAMLSILERYKWHQFSVVTSPIAGHDDFIQAVRERVSAMQDRFKFTILNAV
LVSHHRDL
AALVDSEARVMLLYCTSQEAIDILTAAKDFHLTGENYVWVVTQSVIANPLEAPGQFPVGML
GVHFDTSSS
SLVNEITTAIKVYAYGVEDFTNDLANAGRSLNTQLSCEGEGAARWNTGDRFFRVLRNVSVE
GEAGKPNLE
FTQDGVLKAAELKIMNLRPGVSKQLVWEEIGVWKSWQKEGLDIKDIVWPGNSHTPPQGVP
EKFHLKITFL
EEPPYISLAPPDPVTGKCSMDRGVLCRIASDADITEVDTTLAHRNGSFYQCCSGFCIDLLQKF
SEELGFT
YELVRVEDGRWGTNENGKWNGLIADLVNRKTDMVLTSLMINAEREAVVDFSVPFMETGIA
IVVAKRTGII
SPTAFLEPFDTASWMLVGVVAIQAATFTIFLFEWLSPSGFNMRLSLNQSTPSASHRFSLFRTY
WLVWAVL
FQAAVHVDSPRGFTARFMTNVWAMFAVVFLAIYTANLAAFMITREEFFEFSGLDDHRLSRP
YSQKPLIKF
GTIPWSHTDSTIAKYFKEMHAYMRQFNKSTVHEGVDAVLSAEMDAFIYDGTVLDYLTSQD
EDCRLLTVGS
WYAMTGYGLAFPRNSKYLKMFNKRLLDFRENGDLERLRRYWMTGVCKPGKQEHKSSDPL
ALEQFLSAFLL
LMAGILLAALLLFLEHLYFKYVRKHLAKTDRGGCCALISLSMGKSLTFRGAVYEAQDILRH
HRCRDPICD
THLWKVKRELDISQMRCKQLEKELEAHGIKPPPPCKRVIVSGEQARSRLRSLNPDKSSNSDL
FGQKTEIA
EMETVL

>AplaNMDA
MYLTNLLLTVALALLVVSGGPAWNKSNAVTRAPALKIGSGSSRNASLGRGNGLRIGSSRSGR
TTTTTPAP
VINPTTSTNQKILQLGLVVPQKSFGVREYTKAVNNAKLSLSRKLPSLFKKYDLQVRIVMKEL
TPSPKAIL
KSLCQDFLSVNVSAILYLMNYEQYGRSTASAQYFLQLAGYLGIPVIAWNADNSGLERRASQ
SSLQLQLAP
SLEHQTAAMLSILERYKWHQFSVVTSPIAGHDDFIQAVRERVSAMQDRFKFTILNAVLVAKN
GDLAALVD
SEARVMLLYCTREEAKEILKAAREFHLTGENYVWVVTQSVIENPQSASGQFPVGMLGVHF
DTTSASLVNE
ITTAIKVYAYGVEGFINDQKTDNWSLNTQLSCDGPGVARWDTGDRFFRHLRNVTIESELMG
KPPIEFTPD
GVLKAAELKIMNLRPGVSKQLVWEEIGVWKSWESEGLDIKDIVWPGNSHTPPQGVPEKFH
LKITFLEEPP
YILLTPPDPVSGKCSMDRGVLCRVATDAVMAEVNISQVHRNGSFYQCCSGFCIDLLEKFSEE
LGFTYELV

RVEDGKWGVNENGKWNGLIADLANRKTDMVMTSLTINAEREAAVDFSVPFMETGIAILVA
KRTGIISPTA
FLEPFDTASWMLVGVVAIQAATFTIFLFEWLSPSGFNMRLSLNQGSTTPHRFSLFRTYWLVW
AVLFQAAV
HVDSPRGFTARFMTNVWALFAVVFLAIYTANLAAFMITREEYFEFKGLDDPRLSRPFSNKPM
FKFGTIPW
SHSDSTIAKYFPIMHHYMQEFNKTSVEEGVEAVLSSDLDAFIYDGTVLDYLTVQDEDCRLLT
VGSWYAMT
GYGLAFPRNSKYLKMFNKKLLDFRENGDLERLRRYWMTGTCKPGKQEHKSSDPLALEQFL
SAFLLLMAGI
LLAALLLLLEHLYFKYVRKHLAKTDKGGCCALISLSMGKSLTFRGAVFEAQDILRLHRCRD
PICDTHLWK
VKRDLDISQMRCKQLEKELESHGIKPPPPERQHRRRNRNNTCLSSCLSPSSEANEVILAGEGI
KARLKSL
NFNHQTGSNSDIFSPKTEIAEMETVL
>DponNMDA
MKLTMFALNLLVVSVVSGGPAWKEVQRGGGLKIGEKGGRNASSLRGNGIKIGSGGRGLRS
TTTTTTSVPE
EEFIHPPTSPSDRNGGIKHLNIGIVLPYKSFGVRDYTKAITTTKSLIARKLKLFKSHDIQVHIV
MKALTP
SPTAILKALCKEFLNFNVSAILYLMNYEQYGPSTASAQYFLQLAGYLGIPVIAWNADNSGLE
RRASQSSL
QLQLAPSLEHQTAAMLSILERYKWHQFSVVTSLIAGHDDFIQAVRERVSAMQDRFKFTILNA
VLVAHRGD
LAALVDSEARVMLLYCTKQEAVDILTAASDLHLTGENYVWVVTQSVIETADQAPNQFPVG
MLGVHFDTSS
QSLVNEITAAIKVYAYGVEDFLGDPANWNRSLTTHLSCDGEGVVRWDTGDRFFTYLRNVSV
EAEQGRPNL
EFTPDGLLKAAELKIMNLRPGVSKQLVWEEIGVWKSWQNEGLDIKDIVWPGNSHTPPQGV
PEKFHLKITF
LEEPPYIKLAPPDPVIGKCSMDRGVLCRVASDEAITEVDMAQAHRNGSYYQCCSGFCVDLL
QKFSEELGF
TYELVRVEDGKWGTNQNGRWNGLIADLVNRKTDMVLTSLTINAEREAVVDFSVPFMETGI
AIVVAKRTGI
ISPTAFLEPFDAASWMLVGLVAIHAATFTIFLFEWLSPSGFNMKLAFNNGVPSTAHRFSLFRT
YWLVWAV
LFQAAVHVDSPRGFTSRFMTNVWAMFAVVFLAIYTANLAAFMITREEFFEFTGIDDIRLSRP
YSHKPSIK
FGTIPFSHTDSTLAKYFKDMHAYMKQFNKTTVLNGVATILSGEMDAFIYDGTVLEYLTSQD
EDCRLLTVG
SWHAMTGYGLAFPRNSKYLKMFNKRLLDFRENGDLERLRRYWMTGVCKPGKQEHKSSD
PLALEQFLSAFL
LLMSGILLAALLLLLEHLYFKYIRKHLAKTDRGGCCALISLSMGKSLTFRGAVFEAQDILRN
HRCRDPIC
DTHLWKVKRELDVAQLRIKQLEKEMEVHGIKPPPRKRRRRNASCITSCLSSSKSPDIVVSGE
QAKARMRS
LEPADSGSCSDINGPSPTEIAEMETVL
